# Floristic inventory and distribution characteristics of algific talus slopes in a specific area of forest biodiversity in South Korea

**DOI:** 10.3897/BDJ.11.e113952

**Published:** 2023-12-18

**Authors:** Jong-Won Lee, Ho-Geun Yun, Tae Young Hwang, Kyungmin Kim, Se-Hoon Jung, Jong Bin An

**Affiliations:** 1 Korea National Arboretum, Yanggu, Republic of Korea Korea National Arboretum Yanggu Republic of Korea; 2 Korea National Arboretum, DMZ Forest Biological Conservation, Yanggu-gun, Republic of Korea Korea National Arboretum, DMZ Forest Biological Conservation Yanggu-gun Republic of Korea; 3 Daoneco, Sejong-si, Republic of Korea Daoneco Sejong-si Republic of Korea

**Keywords:** Algific talus slope, Korea endemic plants, rare plants, floristic target species, alien plants, phytogeographic plants

## Abstract

**Background:**

This study conducted a survey for establishing in-situ and ex-situ conservation measures for northern lineage plants that are vulnerable to climate change and for designating Forest Genetic Resource Reserve for 25 algific talus slope sites, which are specific areas of forest biodiversity. The survey was conducted in South Korea within a distance of 50 m to the east, west, north and south from the core area where wind blows to the algific talus slopes. The study was conducted once or twice per season from April 2016 to November 2021.

**New information:**

Vascular plants of 25 algific talus slope sites in South Korea included a total of 1,052 taxa of 125 families, 486 genera, 947 species, 23 subspecies, 75 variety and 7 forma. The maximum surveyed area was 0.09 km^2^, accounting for only 0.00014% of the 62,860 km^2^ forest area in Korea, but comprise 22.27% of the 4,724 species of vascular plants in Korea. The algific talus slopes are areas rich in forest biodiversity. Six taxa were categorized as endangered, including *Paeoniaobovata* Maxim. Sixty-seven taxa, including *Astilboidestabularis* (Hemsl.) Engl.; 58 taxa endemic to the Korean Peninsula, including *Weigelasubsessilis* (Nakai) L.H. Bailey; and 317 taxa of floristic target plants were categorized as rare plants in the Red list. Further, 181 taxa were identified as northern lineage plants and 32 taxa, including *Sillaphytonpodagraria* (H. Boissieu) Pimenov, were limestone area plants. Regarding alien plants, 75 taxa, including *Oenotherabiennis* L., were identified and the naturalization and urbanization rates were 7.13% and 12.12%, respectively. Plants specific to the phytogeography of the 25 algific talus slope sites in this study were *Vacciniumvitis-idaea* L., *Rosakoreana* Kom., SyringavillosaVahlsubsp.wolfii (C.K. Schneid.) Jin Y.Chen & D.Y. Hong, *Lonicerachrysantha* Turcz. ex Ledeb., *Tephroserisflammea* (Turcz. ex DC.) Holub, among others.

## Introduction

Biodiversity refers to the diversity of the biological species on the Earth as well as the diversity of the ecosystems the species inhabit and diversity of the genes they exhibit (United Nations 1992). Advancement in the economy in the 20^th^ century led to sudden urbanization and indiscrete exploitation of natural resources, resulting in rapid increase in the temperature of the Earth due to climate change and global warming (Boo et al. 2006). Acceleration of climate change led to disturbance or alteration of habitats of plants growing in alpine and subalpine zones (Kim and Lee 2011) and the disappearance of the species that failed to adapt to the changing environment (Lee et al. 2022a). Additionally, species population and biodiversity reduced as invasive alien plants dominated the habitats of endemic species, resulting in the elimination of these species from their natural habitats (Kong et al. 2011). Moreover, the reduction of biodiversity is occurring at an unprecedented rate corresponding to the everincreasing causal activities such as overexploitation. Particularly, the COVID-19 pandemic experience has demonstrated the importance of the relationship between humans and nature. The well-being and survival of humans have been predicted to be severely affected by the continuous loss of biodiversity and destruction of the ecosystem (Locke et al. 2021). As biodiversity provides various ecosystem services and has a substantial role in the sustainability of the Earth, ecosystem and humans, there is an urgent need to develop measures that support systematic conservation and sustainable utilization of nature with respect to biodiversity at a national level.

Algific talus slopes are characterized by locally detected unique micrometeorological phenomena such as cold air blowing or water getting frozen during summer and warm wind blowing during winter in a hole or crack on the rock (Kong et al. 2012). In a situation where the habitat of polar and alpine plants that grow at low temperatures is gradually decreasing due to global warming or climate change, the micrometeorological phenomenon of wind holes that maintain a constant temperature regardless of the season is very important as a refuge for plants sensitive to high temperatures. It is thus known that rare plants and plants that are uncommon in the environments at close proximity to humans are widely distributed on the algific talus slopes. Notably, as the habitats of polar and alpine plants that naturally grow in low temperature regions are being continuously reduced due to global warming or climate change, the micrometeorological phenomena on algific talus slopes, where low temperature is maintained even in summer, are critical for providing a refuge for plants sensitive to high temperatures and for the conservation of rare and endangered species (Kong et al. 2017).

Various studies have been conducted in South Korea and other countries on algific talus slopes. In advanced countries such as the U.S. and Japan, the importance of algific talus slopes has been recognized, which has led to their designation as reserve areas for protection. Since 1989, algific talus slopes in the U.S. have been designated as natural wildlife reserves and are managed as such; furthermore, in Japan, continuous studies are conducted on the algific talus slopes in central and northern regions of Honshu (Iokawa and Ishizawa 2003). In South Korea, data on algific talus slopes have been compiled since 1926 with the investigation of forests during the Japanese colonial era. A previous study reported a total of 149 algific talus slopes distributed across the nation (Park 2017). The Rio Convention has emphasized the importance of promoting biodiversity in response to climate change through continuous management, such as topographic and species distribution monitoring. Despite such efforts, all algific talus slopes other than that in Bangnae-ri, Hongcheon, designated as the Forest Gene Resource Reserve (FGRR), are yet to be designated and managed as a reserve and concerns have been raised regarding their destruction (Kim et al. 2016).

The Korea Forest Service designates and manages the FGRRs as forests that require protection and management to conserve the plants within forest ecosystems. The designation of FGRRs is performed by the mayor, governor or local branch of the Korea Forest Service. There are seven types of forests designated as reserve areas: virgin forest, rare and valuable forest, rare plant habitat, alpine plant habitat, forest marsh and valley, natural ecosystem reserve and useful plant habitat. However, most algific talus slopes are currently not designated as reserve areas despite the urgent requirement of active forest management. Hence, algific talus slopes should be designated as FGRRs or there is a need for Other Effective Area-Based Conservation Measures (OECMs). OECMs are regional measures to conserve the biodiversity in and around a reserve area. An area of OECMs is not a reserve area but a geographically defined area that is managed in ways to achieve positive and continuous long-term results towards the conservation of biodiversity and the relevant ecosystem functions and services. The goal is to conserve an area with cultural, spiritual, social and economic values (Hong et al. 2017, Convention on Biological Diversity 2018).

This study was conducted to rediscover the phytogeographic values of algific talus slopes by investigating the distribution of five types across 25 algific talus slopes as the specific areas of forest biodiversity. This will aid in developing optimal conservation measures for algific talus slopes that serve as a refuge for northern lineage plants among alpine plants and those vulnerable to climate change, as well as providing basic data for the systematic management of algific talus slopes and facilitating the designation of algific talus slopes as FGRRs.

## Materials and methods

### Study site

**Figure 1.**
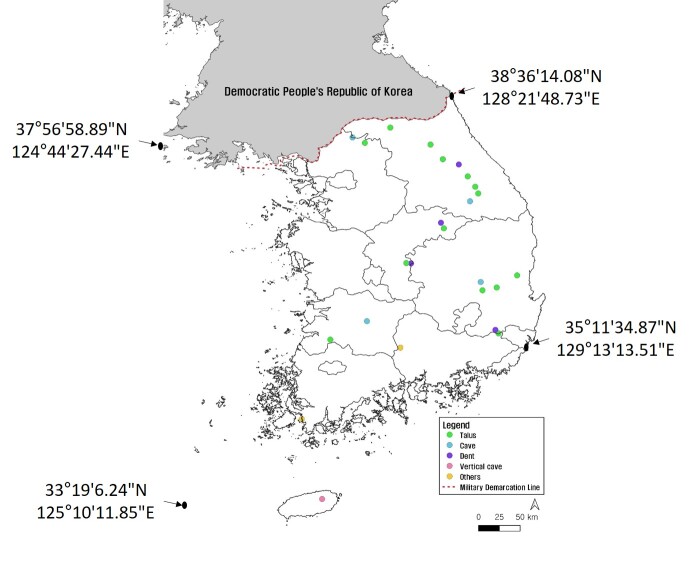
Map showing location of survey areas.

Arboretum of Korea Forest Service (Korea National Arboretum 2013, Suppl. material 1, Fig. 1). Algific talus slopes are classified into five types: talus, cave, dent, vertical cave and others. According to the current status of algific talus slopes by type, 14 algific talus slopes are type talus (e.g. Bangnae-ri, Nae-myeon, Hongcheon-gun), 4 algific talus slopes are type cave (Dongmak-ri, Yeoncheon-eup, Yeoncheon-gun, Gyeonggi-do), 4 algific talus slopes are type dent (Milyang Ice Valley of Samyang-ri, Sannae-myeon, Milyang-si, Gyeongsangnam-do), 1 algific talus slope is type vertical cave (Geom-eun Oreum of Seonheul-ri, Jocheon-eup, Jeju-si, Jeju-do) and 2 algific talus slopes are type others (one of Gwandong-ri, Hwasan-myeon, Haenam-gun, Jeollanam-do and the other of Gwnagjeom-dong, Macheon-myeon, Hamyang-gun, Gyeongsangnam-do) (Fig. 8).

### Method

Vascular plants were investigated in each season during April 2016 to November 2021. In the investigation, we aimed to cover up to 50 m to the east, west, south and north from the centre of the core area from where the wind blows to the algific talus slopes. The detected plants were mainly identified on-site. The plants that posed a difficulty in identification were made into a specimen to be identified at the laboratory in reference to Korea National Arboretum 2008a, Korea National Arboretum 2011, Korea National Arboretum 2012, Lee 2014a, Lee 2014b, Korea National Arboretum 2016, Korea National Arboretum 2019bVscular plants were listed in order from pteridophytes to gymnosperms to angiosperms according to the Engler’s classification system (Melchior 1964). The scientific names were recorded according to the National Standard Floristic Inventory (Korea National Arboretum 2023). The names of rare plants and Red list species were recorded in reference to Korea National Arboretum (2008b), Korea National Arboretum (2021), National Institute of Biological Resources (2021)and those for endangered wildlife were recorded in reference to Korea Ministry of Envrionment (2023). Chung et al. (2017) and Korea National Arboretum (2022) were referred for the endemic plants of the Korean Peninsula. For the endemic species, references were made to National Institute of Biological Resources (2013), National Institute of Biological Resources (2020). The floristic target species were recorded according to the class defined by National Institute of Ecology (2018). The northern lineage plants of the Korean Peninsula were recorded according to Gantsetseg et al. (2020) and the 300 species threatened by climate change were recorded according to Korea National Arboretum (2010). The limestone area plants were listed according to Kim et al. (2021). The alien plants and invasive alien plants were listed in reference to the national floristic inventory of alien plants Korea National Arboretum (2019a) and Kang et al. (2020), respectively. The calculation of the Naturalization Index (NI) and Urbanization Index (UI) was based on Numata and Kotaki (1975) and Kim et al. (2000), respectively.

The set of all the records collected for the present work was included in the dataset of occurrences of vascular plant on algific talus slopes in South Korea, which was published through GBIF (An and Lee 2023).

The distribution of the species listed below is based on The Royal Botanic Garden, Kew (2023).

## Checklists

### Checklist of vascular plant on algific talus slopes in South Korea

#### Huperzia miyoshiana

(Makino) Ching, 1981

43347B93-6B52-5CCC-B1BC-4DD82C556FA6

##### Distribution

Alaska to NW. U.S.A., Russian Far East to Korea, Japan

#### Huperzia serrata

(Thunb.) Trevis., 1874

44092BB2-BF2A-5F19-8B6E-610AF1A61332

##### Distribution

Russian Far East to NE. China and Japan, Hawaiian Islands, Mexico, Cuba to Hispaniola

#### Lycopodium annotinum

L., 1753

D1BBA7C9-11C8-5979-ADF0-7BF55C4E8016

##### Distribution

Temperate Northern Hemisphere

#### Lycopodium obscurum

L., 1753

B2C675A1-0043-5A02-A02B-3AD9435F5D78

##### Distribution

Russian Far East, Subarctic America to U.S.A.

#### Selaginella helvetica

(L.) Spring, 1838

2A570FE0-FF62-5571-B496-B503A3E40BE6

##### Distribution

Europe to Japan and Himalaya

#### Selaginella involvens

(Sw.) Spring, 1843

BC44BFBA-6D0F-5607-B357-E7FEA7FAA804

##### Distribution

Tropical & Subtropical Asia to NW. Pacific

#### Selaginella rossii

(Baker) Warb., 1900

EBF69D36-33B5-5EAE-8728-FA8BFA04EEB0

##### Distribution

South Russian Far East to North China and Korea

#### Selaginella tamariscina

(P.Beauv.) Spring, 1843

E6528388-1CF9-5D88-B06D-832A06779C8C

##### Distribution

Russian Far East to Central & South Malesia

#### Equisetum arvense

L., 1753

EF5DB4C4-AFA1-5203-ACE8-B0FE47D33B29

##### Distribution

Subarctic & Temperate Northern Hemisphere

#### Osmunda cinnamomea

L., 1753

5066ADBF-5EBB-5A9E-B82F-5F406EBCBDCE

##### Distribution

New World, Arunachal Pradesh to Russian Far East and North Indo-China

#### Osmunda japonica

Thunb., 1780

E07B3CAA-5408-5B53-8652-A0834DFA12A5

##### Distribution

Pakistan to Sakhalin and Indo-China

#### Crepidomanes minutum

(Blume) K.Iwats., 1985

E6417977-799A-5C4A-9F97-64BC620A92A1

##### Distribution

India to Ogasawara-shoto and Pacific

#### Dennstaedtia hirsuta

(Sw.) Mett. ex Miq., 1867

4B826784-FCAA-5F65-9462-C9FDFEAAD99C

##### Distribution

China to South Russian Far East and Temperate East Asia

#### Dennstaedtia wilfordii

(T.Moore) Christ, 1910

FA4C551B-10E8-5873-A93C-6A1E0323B299

##### Distribution

Pakistan to West Himalaya, South Russian Far East to China and Japan

#### Microlepia strigosa

(Thunb.) C.Presl, 1851

741AA0DE-512F-561E-9CC3-23CBD048BB7E

##### Distribution

Tropical & Subtropical Asia to Pacific

#### Pteridium aquilinum var. latiusculum

(Desv.) Underw. ex A.Heller, 1909

E6799F54-58A7-5F46-A30A-58D3048F73D1

##### Distribution

India to South Russian Far East and Temperate East Asia, Canada to North Mexico

#### Cystopteris fragilis

(L.) Bernh., 1806

7E051ADF-FA2E-5AAB-9C27-1C7F3156248F

##### Distribution

Cosmopolitan

#### Gymnocarpium dryopteris

(L.) Newman, 1851

3CC6527F-5D55-5970-9132-84292EFB808B

##### Distribution

Temperate Northern Hemisphere

#### Gymnocarpium jessoense

(Koidz.) Koidz. 1936

5D39E1CA-5A32-536D-800B-2DC8862A55B2

##### Distribution

Himalaya to Siberia and Japan

#### Adiantum pedatum

L., 1753

888B276B-DA08-5A52-94F1-6C7B558A6026

##### Distribution

Himalaya to Russian Far East and Japan

#### Cheilanthes argentea

(S.G.Gmel.) Kunze, 1850

0A4651E9-2E40-5F8D-8AA0-00A324414A67

##### Distribution

Siberia to Japan and North Indo-China

#### Coniogramme intermedia

Hieron., 1916

1C43196C-43D7-5FFC-8B76-A3DF0158071B

##### Distribution

Pakistan to Russian Far East and Indo-China, Taiwan

#### Coniogramme japonica

(Thunb.) Diels, 1899

385847AB-7D47-5BC1-AD70-BE3B55E111A1

##### Distribution

Central & S. China to North Vietnam and Temperate East Asia

#### Asplenium incisum

Thunb., 1794

A44A9B7D-5669-58F2-A43D-D2275471FEAD

##### Distribution

Russian Far East to China and Temperate East Asia

#### Asplenium pekinense

Hance, 1867

23A0F01E-1ACD-506C-8426-2919DE9B5D71

##### Distribution

Pakistan to South Russian Far East and Temperate East Asia

#### Asplenium ruprechtii

Sa.Kurata, 1961

A5C827DA-576A-50FF-9FAD-F32334915071

##### Distribution

South Siberia to Japan and China

#### Asplenium tenuicaule

Hayata, 1914

26990ED1-218C-5EC6-BFF5-AE2D6C32AFB3

##### Distribution

Pakistan to South Russian Far East and Philippines

#### Asplenium trichomanes quadrivalens

D.E.Mey., 1962

CE07F8F9-BF33-57C6-AE51-698EEB2BF915

##### Distribution

North America, Temperate & Subtropical Old World to NorthEast & East Tropical Africa

#### Parathelypteris japonica

(Baker) Ching, 1963

A60125A6-B57A-5A26-B68D-B671C4A50172

##### Distribution

South China to Temperate East Asia

#### Phegopteris connectilis

(Michx.) D.Watt, 1867

9E2F7620-C7FA-5673-9CDC-853FBF3DECBB

##### Distribution

Subarctic & Temperate Northern Hemisphere

#### Thelypteris palustris

(A.Gray) Schott, 1834

4C2BB448-56C5-5B99-B880-000CD5F88F4A

##### Distribution

Central Canada to Mexico, Bermuda, Cuba, Temperate Eurasia, Morocco

#### Woodsia macrochlaena

Mett. ex Kuhn, 1868

71A3B3A3-D060-51C9-AA3D-14BAD9B060B8

##### Distribution

South Russian Far East to Japan and North China

#### Woodsia manchuriensis

Hook., 1861

9D4CD52E-C71C-593F-ABD2-08999BBB2C72

##### Distribution

China to South Russian Far East and Japan

#### Woodsia microsora

Kodama, 1917

81384239-D5E8-5686-92A2-C5C4C7A6C7DB

##### Distribution

North Korea

#### Woodsia polystichoides

D.C.Eaton, 1858

D790AC0B-0586-50C7-B385-46E5A2C68D88

##### Distribution

Mongolia to Japan and China

#### Woodsia subcordata

Turcz., 1832

739B6573-CFB5-5BEF-8BCB-26C92E1B00FD

##### Distribution

SouthEast Siberia to North & Central Japan and North China

#### Onoclea interrupta

(Maxim.) Ching & P.C.Chiu, 1974

793CB55C-CB62-50E5-8DBB-3AF33BC3AA85

##### Distribution

SouthEast Siberia to Japan and China

#### Pentarhizidium orientale

(Hook.) Hayata, 1927

BB7948BF-85BA-5B2A-844C-EB3489592BCE

##### Distribution

East Himalaya to South Russian Far East and Japan

#### Athyrium iseanum

Rosenst., 1913

61C7219C-2D81-5A10-AEDB-895E4EB4D79B

##### Distribution

China to Japan

#### Athyrium monomachii

(Kom.) Kom., 1931

CFFE7FDA-909C-5412-9025-DE6C5636AEE9

##### Distribution

Siberia to North & Central Japan and North China

#### Athyrium niponicum

(Mett.) Hance, 1872

5ED429E0-ECEC-583B-A55F-78F521F33DAA

##### Distribution

East Himalaya to Temperate East Asia and North Indo-China

#### Athyrium spinulosum

(Blume) Milde, 1870

8E478681-B2E4-54EE-B978-0A0B45E18F90

##### Distribution

Sulawesi to New Guinea

#### Athyrium yokoscense

(Franch. & Sav.) Christ, 1896

287BBA48-5394-5C40-A8CC-01336DA9BB09

##### Distribution

China to South Russian Far East and Japan

#### Cornopteris crenulatoserrulatum

(Makino) Nakai, 1931

6D0FE8C1-CEF4-5C57-9CBE-8B935EFAFD27

##### Distribution

South Russian Far East to North & Central Japan and North & East China

#### Deparia conilii

(Franch. & Sav.) M.Kato, 1977

7B45704C-D130-5093-AFD2-01E525088514

##### Distribution

East China to Japan

#### Deparia coreana

(Christ) M.Kato, 1984

13FE7416-2421-583A-BF82-F57F5ED9477C

##### Distribution

China to South Russian Far East and Japan

#### Deparia japonica

(Thunb.) M.Kato, 1977

14DC1953-F84A-57C3-A0CD-D2527618A61E

##### Distribution

Pakistan to South Kuril Islands and Lesser Sunda Islands

#### Deparia pycnosora

(Christ) M.Kato, 1977

BCECC432-AA96-58CD-9CAD-88AAE1D55603

##### Distribution

Russian Far East to Japan and North China

#### Diplazium sibiricum

(Turcz. ex Kunze) Sa.Kurata, 1961

9545438F-456D-567F-8996-DBAE8551F217

##### Distribution

North & NorthEast Europe to North & Central Japan

#### Arachniodes borealis

Seriz., 1986

4D900BDD-4A26-5C58-99C1-CFBDD9275938

##### Distribution

East Himalaya to South Russian Far East and Japan

#### Arachniodes standishii

(T.Moore) Ohwi, 1962

6F060373-0653-56B1-8F02-48C4DCFDF4AF

##### Distribution

Korea (Jeju-do), Japan

#### Cyrtomium fortunei

J.Sm., 1866

52F5EF94-ED6C-5CFD-9893-A7961D0BD691

##### Distribution

East Himalaya to Korea and Indo-China, Japan, Taiwan

#### Dryopteris bissetiana

(Baker) C.Chr., 1905

8C654749-B236-50F6-B3CC-370EE9B4A019

##### Distribution

China, South Korea, Japan

#### Dryopteris chinensis

(Baker) Koidz., 1930

A65BBE11-9CDB-5174-94F3-7A9BCDAB086D

##### Distribution

South Russian Far East to China and Japan

#### Dryopteris crassirhizoma

Nakai, 1920

3C1F25CD-7BB5-515D-A604-3A207DE13F6C

##### Distribution

Central East & Central North China to Russian Far East and Japan

#### Dryopteris erythrosora

(D.C.Eaton) Kuntze, 1891

321FBE7D-E69C-500D-998E-A4BCE0D11BB1

##### Distribution

China to Temperate East Asia

#### Dryopteris expansa

(C.Presl) Fraser-Jenk. & Jermy, 1977

02FF5D9D-1CA1-5ED2-B58A-460B1FCA0402

##### Distribution

Subarctic & Temperate Northern Hemisphere

#### Dryopteris fragrans

(L.) Schott, 1834

FD1A3DEB-7780-5FAD-8E65-27D21C5FD597

##### Distribution

North Finland to Russian Far East and Korea, North & Central Japan, Subarctic America to North U.S.A.

#### Dryopteris gymnophylla

(Baker) C.Chr., 1905

1B9B0348-1139-5E99-9238-55AFE4CD4366

##### Distribution

China to Japan and North Thailand

#### Dryopteris lacera

(Thunb.) Kuntze, 1891

269FE0FF-37E1-5184-957A-0D28BFAC8547

##### Distribution

China to Temperate East Asia

#### Dryopteris sacrosancta

Koidz., 1924

FA666C8D-F93C-5D38-BBEB-37AAB168B298

##### Distribution

Central & South Japan

#### Dryopteris saxifraga

H.Itô, 1936

B703D20D-C4BA-5001-99E4-986E69773324

##### Distribution

China (Jilin, Liaoning), South Korea, Japan

#### Dryopteris uniformis

(Makino) Makino, 1909

93BE2227-3522-58E8-8B0F-4EB7214A5BEE

##### Distribution

SouthEast China, South Korea, Japan

#### Polystichum braunii

(Spenn.) Fée, 1852

5CC7E9B2-8BA0-530E-AFB6-A7E46056D565

##### Distribution

Temperate Northern Hemisphere

#### Polystichum craspedosorum

(Maxim.) Diels, 1899

8CCFC80A-0CCF-5FE2-81AD-8E14F8E9367B

##### Distribution

South Russian Far East to Japan and China

#### Polystichum ovato-paleaceum var. coraiense

(Christ) Sa.Kurata, 1964

831A0B5D-9512-50C4-B08A-AB88A1A953D7

##### Distribution

Central Korea, North & Central Japan

#### Polystichum polyblepharum

Nakai, 1925

89FFDE68-EF84-5344-8BBA-564D88AAFEE3

##### Distribution

Zimbabwe to South Africa, West Indian Ocean, Himalaya to Japan and Vietnam

#### Polystichum tripteron

(Kunze) C.Presl, 1851

ADF71ADB-BD3E-51F5-95A1-DDC277490E0A

##### Distribution

China to South Russian Far East and Japan

#### Davallia mariesii

H.J.Veitch, 1880

1AE4219A-FF77-5FD3-8094-3CE1D89F59C7

##### Distribution

North & East China to Temperate East Asia

#### Lemmaphyllum microphyllum

C.Presl, 1851

7F8C5947-ACA0-5C29-8CE1-4A0FAE6B71EF

##### Distribution

Arunachal Pradesh to Temperate East Asia

#### Lepisorus onoei

(Franch. & Sav.) Ching, 1933

F4077317-8523-5EF7-8FAF-684FCFAB7648

##### Distribution

South Korea, Japan to North Nansei-shoto

#### Lepisorus thunbergianus

(Kaulf.) Ching, 1933

6B083EB2-F53B-5262-A01C-66B742BCD62A

##### Distribution

Himalaya to Temperate East Asia, Philippines (North Luzon), Hawaiian Islands

#### Lepisorus ussuriensis

(Regel & Maack) Ching, 1933

3956B48D-B693-5AE6-90D3-136FA8ADDA4E

##### Distribution

South Russian Far East to North & East China and Korea, Japan

#### Pleurosoriopsis makinoi

(Maxim. ex Makino) Fomin, 1939

46A7548A-5B15-559C-B1FA-89B7D08E6071

##### Distribution

South Russian Far East to China and Japan

#### Polypodium sibiricum

Sipliv., 1974

261DDD4C-5AEF-5EF1-9C90-9EA25E05DAC3

##### Distribution

Siberia to North & Central Japan and North China, Subarctic America to West & Central Canada

#### Pyrrosia linearifolia

(Hook.) Ching, 1935

C8A9E560-FD29-5DAB-94D0-25C0832E4A93

##### Distribution

China (Jilin) to Temperate East Asia

#### Pyrrosia petiolosa

(Christ) Ching, 1935

874DD141-65AF-5C7B-8DC2-A38BA4D96554

##### Distribution

Mongolia to Korea and China

#### Ginkgo biloba

L., 1771

B9E06624-AED3-5AD4-A603-713B16BFB4BF

##### Distribution

China (Zhejiang)

#### Abies holophylla

Maxim., 1866

9A88B1FD-8C1F-5948-8168-87DE7213389E

##### Distribution

Primorye to Korea

#### Abies nephrolepis

(Trautv. ex Maxim.) Maxim., 1866

894DD425-4555-5206-BFC7-5F6355353D0F

##### Distribution

Russian Far East to Korea

#### Larix kaempferi

(Lamb.) Carrière, 1856

57423B95-5514-5872-ACFF-D434B1C6160D

##### Distribution

Central Japan

#### Picea abies

(L.) H.Karst., 1881

E448445C-BB39-5BFC-959F-1F94D4A0413B

##### Distribution

Europe

#### Picea jezoensis

(Siebold & Zucc.) Carrière, 1855

F673C7DD-C0F8-54BA-9CC2-2ECAB31DB479

##### Distribution

Russian Far East to Korea, North & Central Japan

#### Pinus densiflora

Siebold & Zucc., 1842

A7B419E5-FBEC-58FD-810C-3FAF71675C23

##### Distribution

South Russian Far East to Korea and Central & South Japan

#### Pinus koraiensis

Siebold & Zucc., 1842

B4152113-A6B4-55DA-B8C6-E8DA3204B6EF

##### Distribution

Russian Far East to Korea, Japan

#### Pinus rigida

Mill., 1768

1E49D1F1-88CE-5A4E-9D9A-6CF738580362

##### Distribution

SouthEast Canada to East U.S.A.

#### Pinus thunbergii

Parl., 1868

970FBDFB-282A-586D-AC9D-418A7FDD44A4

##### Distribution

Korea, Central & South Japan

#### Juniperus rigida

Siebold & Zucc., 1846

9BF1F0E8-ACBD-525B-B60A-B38FD78EC935

##### Distribution

NorthEast China, Korea, Japan

#### Thuja koraiensis

Nakai, 1919

7F3A392B-8144-5AAF-BB43-A7C806ED6E36

##### Distribution

China (Jilin) to Korea

#### Cephalotaxus harringtonia

(Knight ex J.Forbes) K.Koch, 1873

3282C306-2FC3-5E25-9C70-E2BF8DDCE967

##### Distribution

China to Korea, Central & S. Japan, Taiwan

#### Taxus cuspidata

Siebold & Zucc., 1846

5F5663F6-6D46-5D0F-A68C-693643C6ABB1

##### Distribution

South Russian Far East to North China and Japan

#### Juglans mandshurica

Maxim., 1856

FAD3F927-C746-55E7-ACFD-74EB417E336B

##### Distribution

Russian Far East to China and Temperate East Asia

#### Platycarya strobilacea

Siebold & Zucc., 1843

1C8D96DF-205D-5A27-9ABA-D61F44950383

##### Distribution

Central & South China to Vietnam, Korea, Central & S. Japan

#### Populus tremula var. davidiana

(Dode) C.K.Schneid., 1916

859C1ED1-F101-574B-8079-BEF08B1C72F6

##### Distribution

Temperate Eurasia, Algeria

#### Populus tomentiglandulosa

T.B.Lee, 1955

AD480970-1B0C-5CEF-9423-664E719E39C3

##### Distribution

Korea (artificial hybrid)

#### Salix caprea

L., 1753

10947084-5185-5846-8F3B-83FFBE48066A

##### Distribution

Europe to North Asia, North China to Japan

#### Salix gracilistyla

Miq., 1867

50B9B419-6837-53E8-BCCF-B87EBF021A05

##### Distribution

Russian Far East to North China and Korea, Japan

#### Salix koriyanagi

Kimura ex Goerz, 1931

C47CC68E-6049-5EE4-8F92-5D85C187E063

##### Distribution

Korea

#### Salix pierotii

Miq., 1867

8A34EF35-6B73-53E7-8314-7BC7F3790516

##### Distribution

Russian Far East to North China and Japan

#### Salix rorida

Laksch., 1911

E0149A74-9FA4-5F86-9A7F-71A200FE3BAF

##### Distribution

Central Asia to Russian Far East and Japan

#### Salix xerophila

Flod., 1930

1B2F247E-DA48-5B22-9A99-5B4DC16492DE

##### Distribution

Subarctic to U.S.A.

#### Alnus incana hirsuta

(Spach) Á.Löve & D.Löve, 1976

DE765E6D-944D-5953-B134-AD9AB734E134

##### Distribution

South Siberia to Korea, Japan

#### Alnus japonica

(Thunb.) Steud., 1840

9EF20323-1EF2-5F49-BBD2-3040EBA201B4

##### Distribution

South Russian Far East to East China, Korea, Japan, Taiwan

#### Betula chinensis

Maxim., 1879

78B3D402-52AC-55B1-BA48-8DADBA05E1C6

##### Distribution

North & East China to Korea

#### Betula costata

Trautv., 1859

8E40ADA0-257B-59AE-ACBC-0344DEE5EFEB

##### Distribution

Russian Far East to Korea

#### Betula davurica

Pall., 1784

889CF90A-4228-5CA9-9538-3DC8CF3C250D

##### Distribution

SouthEast Siberia to North & Central Japan

#### Betula ermanii

Cham., 1831

F130CCF5-7A2E-5A1E-B8EF-98A628E2FBB0

##### Distribution

Siberia to Japan

#### Betula pendula

Roth, 1788

5EEB1FA3-B7D8-59DF-9590-CEB1CF69E63D

##### Distribution

Temperate Eurasia, NorthWest Africa, Alaska to Canada

#### Betula schmidtii

Regel, 1865

B83F5DD6-6D33-56AD-8D37-B12D01215E8A

##### Distribution

Russian Far East to Korea, Japan (North & Central Honshu)

#### Carpinus cordata

Blume, 1851

89FFC28F-12AE-519E-A174-6196C128B8D2

##### Distribution

SouthWest Primorye, China, Korea, Japan

#### Carpinus laxiflora

(Siebold & Zucc.) Blume, 1851

4020BA0D-B2D2-58CC-A6F7-69E02B0325A0

##### Distribution

Japan, Korea

#### Carpinus tschonoskii

Maxim., 1882

EAAAFF4E-1227-591D-9959-D08BFC189F19

##### Distribution

South China, South Korea, Central & South Japan

#### Carpinus turczaninovii

Hance, 1869

53B60F74-B257-5468-920D-75DC4C0B7E61

##### Distribution

China, Korea, South Central & South Japan

#### Corylus heterophylla

Fisch. ex Trautv., 1844

1BE6B847-25C6-5F0A-8173-4ACF7EF2207D

##### Distribution

SouthEast Siberia to China and Japan

#### Corylus sieboldiana

Blume, 1851

0D4F6577-F69C-5469-8717-AC81485A0EBE

##### Distribution

South Siberia to North China and Japan

#### Corylus sieboldiana var. mandshurica

(Maxim.) C.K.Schneid., 1916

6DE33CC8-D3FA-5E76-AC75-B0DD4F11310B

##### Distribution

Southeast Siberia to North China and North & Central Japan

#### Castanea crenata

Siebold & Zucc., 1846

D743BDEF-0DEF-5226-80E7-0DBE82A0BB93

##### Distribution

Korea, Japan

#### Quercus acutissima

Carruth., 1861

32DC8436-EDD4-53BF-856E-CA6E9F576437

##### Distribution

Himalaya to China and Indo-China, Korea, Central & S. Japan

#### Quercus aliena

Blume, 1851

AEEA98B6-9A96-59FE-91AF-1170F98FBE76

##### Distribution

South Russian Far East to Indo-China, Central & South Japan, Taiwan

#### Quercus dentata

Thunb., 1784

99A84C40-E022-5647-9127-F36007807769

##### Distribution

Mongolia to South Russian Far East and China, Temperate East Asia

#### Quercus mongolica

Fisch. ex Ledeb., 1850

9A5CE6D5-3E84-597E-A0B6-23615692AF41

##### Distribution

SouthEast Siberia to Japan and North China

#### Quercus serrata

Murray, 1784

8CDB0EA6-3D52-5243-81D0-01576D9D9B27

##### Distribution

East Himalaya to China, Taiwan, Korea (including Chenju Do), Japan

#### Quercus variabilis

Blume, 1851

16EB64D5-DB6C-5189-92DA-0B8B900C9E62

##### Distribution

Central & South Japan, Korea, Taiwan, Central, East & South China, Tibet, Vietnam

#### Eucommia ulmoides

Oliv., 1890

6E7BB570-5C6E-50D1-80EC-90CC27113CBC

##### Distribution

Central & S. China

#### Celtis aurantiaca

Nakai, 1930

ED10F4A4-4EC4-5C04-A61B-1FA023481475

##### Distribution

East China to Korea

#### Celtis biondii

Pamp., 1910

AEF0087B-726D-514C-90B2-3B2E63D54C0B

##### Distribution

Central & South China to Temperate East Asia

#### Celtis bungeana

Blume, 1856

666A03F5-5589-5862-A722-FA49B6932680

##### Distribution

China to Korea

#### Celtis choseniana

Nakai, 1930

CDAF4173-1872-5E49-9FD1-CF7F9191FA73

##### Distribution

Korea, Japan

#### Celtis jessoensis

Koidz., 1913

1634659A-8EE3-5F29-A679-80AAB3753224

##### Distribution

Korea, Japan

#### Celtis koraiensis

Nakai, 1909

DAA01185-9891-56BF-ABF6-323C9D6D09F5

##### Distribution

East China to Korea

#### Celtis sinensis

Pers., 1805

8976F263-F5A4-5027-9E04-C1394C3139AB

##### Distribution

Central & S. China to Indo-China and Temperate East Asia

#### Hemiptelea davidii

(Hance) Planch., 1872

4A2519B4-62C3-5437-B054-5C597D6B2852

##### Distribution

China to Korea

#### Ulmus davidiana var. japonica

(Rehder) Nakai, 1932

2CC65440-29D6-5ECA-AA22-27C6CD35B0E5

##### Distribution

South Siberia to North Myanmar and Japan

#### Ulmus laciniata

(Herder) Mayr ex Schwapp., 1895

F58F77B8-D66F-5A1C-B4EE-EEE09FCCB3F2

##### Distribution

Russian Far East to China and Japan

#### Ulmus macrocarpa

Hance, 1868

19ABEF49-05AE-5656-9AC2-180BC5870B9D

##### Distribution

SouthEast Siberia to Korea and East Qingai

#### Ulmus parvifolia

Jacq., 1798

420AF4CB-9FF8-5993-A574-F759067AC484

##### Distribution

Central & South China to Vietnam, S. Korea, Japan (Honshu, Kyushu) to Taiwan

#### Zelkova serrata

(Thunb.) Makino, 1903

08BCE2F2-0B26-586F-8DA7-752E5A645120

##### Distribution

China to South Kuril Islands and Temperate East Asia

#### Broussonetia hanjiana

M.Kim, 2009

C91CDE1E-B178-5906-A80D-5B5687C9778A

##### Distribution

Korea and Japan

#### Fatoua villosa

(Thunb.) Nakai, 1927

6A2FA4B4-5354-5448-B124-D6C73A31A1CA

##### Distribution

Central & South Japan to Jawa and North Australia

#### Ficus erecta

Thunb., 1786

DE84D747-B5AA-5162-994C-6967DB16E25C

##### Distribution

East Himalaya to South China and Vietnam, Temperate East Asia

#### Morus alba

L., 1753

4112C9A4-6DE3-54D5-896F-1CB780D6CD22

##### Distribution

Central China

#### Morus bombycis

Koidz., 1915

1A697E73-06C9-5F0B-9B0E-AC286B76409D

##### Distribution

Sakhalin and Indo-China

#### Morus mongolica

(Bureau) C.K.Schneid., 1916

E939B78A-7154-58A4-9C66-6CD9F4F9CA68

##### Distribution

Mongolia to China and Japan

#### Humulus scandens

(Lour.) Merr., 1935

DC84CBE6-FB65-51E5-B281-0F2F0A6C9F09

##### Distribution

Russian Far East to North Vietnam and Temperate East Asia

#### Boehmeria japonica

(L.f.) Miq., 1867

373623D8-7BDF-58C2-A6DE-2EBF46F8A841

##### Distribution

China, Kuril Islands to Temperate East Asia

#### Boehmeria nivea

(L.) Gaudich., 1830

8C81BA2B-6732-59C8-99EC-BBB3328A8EED

##### Distribution

Indian Subcontinent to Temperate East Asia and Indo-China

#### Boehmeria paraspicata

Nakai, 1930

C0394AA2-1388-5F35-9422-ED088CB040B9

##### Distribution

Central & East China to Japan, North Taiwan

#### Boehmeria platanifolia

(Franch. & Sav.) C.H.Wright, 1899

62B9084B-2BA1-598F-997E-67AADAB67D0C

##### Distribution

China, South Korea, Japan

#### Boehmeria spicata

(Thunb.) Thunb., 1794

3D910D01-A4E4-5A00-8E6B-D37D2ACC0E4E

##### Distribution

Central & East China to Japan, North Taiwan

#### Boehmeria tricuspis

(Hance) Makino, 1912

407A371D-C3F1-5D02-A7E1-C5E984A57FAF

##### Distribution

Central & South China, Kuril Islands to Temperate East Asia

#### Laportea cuspidata

(Wedd.) Friis, 1981

F8921034-63C3-5481-B727-2714D08F9868

##### Distribution

SouthEast Tibet to Central & South China and Myanmar, South Korea, Japan

#### Nanocnide japonica

Blume, 1856

0056AF2F-24C3-5E18-9337-50213695D237

##### Distribution

Central & South China, South Korea, Central & South Japan, Taiwan

#### Pilea japonica

(Maxim.) Hand.-Mazz., 1929

A0DA2455-C052-5151-BCE5-8785878F7B40

##### Distribution

South Russian Far East to China and Temperate East Asia

#### Pilea peploides

(Gaudich.) Hook. & Arn., 1832

4DE20D1E-8CBD-5F56-9985-CDFB0BF2F036

##### Distribution

South Russian Far East and Malesia, Hawaiian Islands, Galápagos

#### Pilea pumila

(L.) A.Gray, 1848

90BC0179-21FE-5EF1-A331-6D2574984448

##### Distribution

East Canada to Central & East U.S.A.

#### Urtica angustifolia

Fisch. ex Hornem., 1819

E4B72538-FD67-5A3C-A4F1-23AFBC3827F3

##### Distribution

Temperate Eurasia

#### Urtica laetevirens

Maxim., 1876

57701B0B-D051-5039-BF7E-50FBC5937FC7

##### Distribution

Russian Far East to North & Central China, South Korea, Japan

#### Urtica thunbergiana

Siebold & Zucc., 1846

62BB6235-2B90-56E5-8392-C8E673E8F017

##### Distribution

Russian Far East to North & Central China, South Korea, Japan

#### Thesium chinense

Turcz., 1837

571E8E75-258E-5C43-B25D-CA6286348DF0

##### Distribution

SouthEast Siberia to China and Temperate East Asia

#### Viscum album var. lutescens

(Makino) Kitag., 1979

BB81D9AC-EC9B-5B8B-BF7E-6DDB51142D8A

##### Distribution

Russian Far East to China and Temperate East Asia

#### Aconogonon alpinum

(All.) Schur, 1853

96E35263-614A-598B-AF51-897287F3985C

##### Distribution

Temperate Eurasia

#### Fagopyrum esculentum

Moench, 1794

458020DE-AFB2-5EF8-AFB1-4B1FD5864256

##### Distribution

East Tibet to China (Sichuan, Yunnan)

#### Fallopia dentatoalata

(F.Schmidt) Holub, 1971

A0B8FEBF-806D-58DD-99A7-E0D2E5D531D7

##### Distribution

Russian Far East to North & Central Japan and West Himalaya

#### Fallopia dumetorum

(L.) Holub, 1971

A9B96775-F65A-5962-8DE9-D1D8CE3BC8A9

##### Distribution

Temperate Eurasia

#### Persicaria filiformis

(Thunb.) Nakai, 1914

49BE215D-4EC4-5A4A-BA26-D3CBB82D9F72

##### Distribution

NortheEast Pakistan to Kuril Islands and Philippines

#### Persicaria hydropiper

(L.) Delarbre, 1800

5C473C30-33D8-57FA-B4AB-CF20B6827DD0

##### Distribution

NorthWest Africa, Temperate Eurasia to West & Central Malesia, Australia

#### Persicaria lapathifolia

(L.) Delarbre, 1800

96C67CF2-9869-532C-9ABB-DBA8D672C4CF

##### Distribution

Subarctic & Temperate Northern Hemisphere to West & Central Malesia, North Africa to Ethiopia

#### Persicaria longiseta

(Bruijn) Kitag., 1937

524033EE-134B-5BEC-A8EA-206C0D5AC56E

##### Distribution

Pakistan to Russian Far East and Philippines

#### Persicaria nepalensis

(Meisn.) H.Gross, 1913

0B6F1DCC-1B0A-5EFC-9F8B-4546976DB061

##### Distribution

Eritrea to KwaZulu-Natal, Madagascar, Tropical & Subtropical Asia to Russian Far East

#### Persicaria perfoliata

(L.) H.Gross, 1919

667F2DC9-A1EF-5710-8F32-C8FF224BBA9C

##### Distribution

NorthEast Türkiye to Russian Far East and New Guinea

#### Persicaria posumbu

(Buch.-Ham. ex D.Don) H.Gross, 1913

06402B58-6572-5D86-A239-5B3CBAC51E0C

##### Distribution

Himalaya to Russian Far East and Philippines

#### Persicaria sagittata

(L.) H.Gross, 1919

32D38D16-CD14-5BF8-AB00-F3ECCC033CE4

##### Distribution

Siberia to Himalaya and Temperate East Asia, Central & East Canada to Central & East U.S.A., Hispaniola

#### Persicaria senticosa

(Meisn.) H.Gross, 1919

A88CEAA7-B154-5086-8B46-756474D0D386

##### Distribution

China to Vietnam, Russian Far East to Temperate East Asia

#### Persicaria thunbergii

(Siebold & Zucc.) H.Gross, 1913

37BDF435-CC25-5957-A421-D2A1E5DC3EE1

##### Distribution

Northeast. Türkiye to West Caucasus, Himalaya to Russian Far East and Temperate East Asia

#### Polygonum aviculare

L., 1753

47D3A0BD-2758-55D5-A374-4943F931F1A0

##### Distribution

Temperate Northern Hemisphere, Macaronesia to Eritrea

#### Reynoutria ciliinervis

(Nakai) Moldenke, 1941

35EF3BB6-1653-5350-AC70-FBD7CF094654

##### Distribution

China to Korea

#### Reynoutria japonica

Houtt., 1777

BB6B8A4E-7748-5D72-AD9B-28E52BF9216E

##### Distribution

Russian Far East to China and Temperate East Asia The Royal Botanic Garden, Kew (2023)

#### Rumex acetosa

L., 1753

96E8493B-F7FB-5E17-B60F-136DCDD5C63E

##### Distribution

Temperate Eurasia, NorthWest Africa The Royal Botanic Garden, Kew (2023)

#### Rumex acetosella

L., 1753

2ACB8EA8-1AA7-5138-9C49-06FDC496D446

##### Distribution

Temperate Eurasia

#### Rumex crispus

L., 1753

D0010EF9-2F33-5CFA-8F91-F4A4AFFB37A1

##### Distribution

Macaronesia, North Africa, Temperate Eurasia

#### Rumex japonicus

Houtt., 1777

434D9A56-340F-58E6-9EB9-9D89462C479B

##### Distribution

Russian Far East to Vietnam and Temperate East Asia

#### Rumex obtusifolius

L., 1753

22AC08D7-4929-5131-8571-08CA32815AB3

##### Distribution

Europe to Central Siberia and Iran, NorthWest Africa

#### Phytolacca americana

L., 1753

3C0906E2-97B7-5A73-B1F1-E94A40535417

##### Distribution

East Canada to Mexico

#### Mollugo pentaphylla

L., 1753

F4BF879C-7158-5532-9431-8876DFB83DD2

##### Distribution

India, Sri Lanka, Korea

#### Portulaca oleracea

L., 1753

9382F1C9-4B1D-5E31-8EE5-5622F9A98AD5

##### Distribution

Macaronesia, Tropical Africa, Mediterranean to Pakistan and Arabian Peninsula

#### Arenaria serpyllifolia

L., 1753

89DF1263-A4F4-5298-8FEB-D4DC4004A757

##### Distribution

Temperate Eurasia to Philippines (Luzon), North Africa to Ethiopia

#### Cerastium glomeratum

Thuill., 1799

F2FD0932-3CC1-58C5-BCBA-FBFF3961BED3

##### Distribution

Europe, Macaronesia to Assam

#### Cerastium holosteoides vulgare

(Hartm.) Buttler, 1997

7ABAFF6F-CBE7-5815-9AD1-80FF3FE6C3FB

##### Distribution

Temperate & Subarctic Eurasia to New Guinea (Mt. Kaindi)

#### Dianthus chinensis

L., 1753

C20ACFB3-8873-5257-B93E-60F3A98AAD33

##### Distribution

East Europe to Temperate Asia and North India

#### Lychnis cognata

Maxim., 1859

CBEFEA43-0BEE-5C6F-A9A7-C67F715AF33A

##### Distribution

South Russian Far East to North & East China and Korea

#### Pseudostellaria coreana

(Nakai) Ohwi, 1935

2AA80113-E4A2-5E66-8C07-DD2FC6C8462C

##### Distribution

Korea, Japan (Honshu)

#### Pseudostellaria heterophylla

(Miq.) Pax, 1934

189E8EC2-FE38-568A-86E2-40382BA9CF35

##### Distribution

South Russian Far East to China and Central & South Japan

#### Pseudostellaria palibiniana

(Takeda) Ohwi, 1935

6F9B322F-3BBC-562E-9529-9588CB84B6D1

##### Distribution

Korea, Japan (Honshu)

#### Sagina japonica

(Sw.) Ohwi, 1937

4ADC88DB-4702-5EBD-8886-1D79599ADE26

##### Distribution

Indian Subcontinent to Russian Far East and Temperate East Asia

#### Silene baccifera

(L.) Durande, 1782

90730BAE-E01A-5D3B-BDCB-E13968E58536

##### Distribution

Temperate Eurasia

#### Silene firma

Siebold & Zucc., 1845

4732CA07-F848-56C1-8480-147635B56684

##### Distribution

SouthEast Siberia to China and Temperate East Asia

#### Silene seoulensis

Nakai, 1909

6CBB9EE0-1715-5B4F-A973-6F23719213E7

##### Distribution

NorthEast China to Korea, Japan (Shikoku)

#### Stellaria alsine

Grimm, 1767

62E52EEB-532E-593F-BB71-094C18688638

##### Distribution

Temperate Northern Hemisphere to West Malaysia

#### Stellaria aquatica

(L.) Scop., 1771

46268EAA-487E-539C-9C83-C36CE2BD6CD9

##### Distribution

Temperate Eurasia

#### Stellaria media

(L.) Vill., 1789

19A8804B-9060-5CC0-ACB8-61D825920117

##### Distribution

Temperate Eurasia, North & Northeast Tropical Africa

#### Chenopodium album

L., 1753

EB21D658-09E4-5735-87F5-5DB8A27F1DB4

##### Distribution

Temperate to Indian Subcontinent

#### Chenopodium album var. centrorubrum

Makino, 1910

454EA371-94AD-577C-A0F3-C9A59332E8BA

##### Distribution

Temperate to Indian Subcontinent

#### Chenopodium bryoniifolium

Bunge, 1876

840E8D39-69B4-52BF-B26E-F8AADE61FBD6

##### Distribution

SouthEast Siberia to North & Central Japan

#### Chenopodium ficifolium

Sm., 1800

3CEE6F62-2A61-51C8-B5E0-4532AFA734D7

##### Distribution

Europe to Korea and North Indo-China

#### Achyranthes bidentata var. japonica

Miq., 1865

91A73259-E2C2-5655-A21D-3935896F92F2

##### Distribution

Tropical & Subtropical Asia to Northwest Pacific

#### Amaranthus blitum oleraceus

(L.) Costea, (2001)

7D8D2CD1-31FF-559D-B60D-82D85020F1E5

##### Distribution

Paraguay to South Brazil and NorthEast Argentina

#### Amaranthus patulus

Bertol., 1837

474F32C5-B8E9-5E88-81B2-258BD95BCF53

##### Distribution

South Ontario to West South America

#### Amaranthus retroflexus

L., 1753

6C44A0B8-764F-56E3-80FB-C60E064C321F

##### Distribution

Mexico

#### Magnolia sieboldii

K.Koch, 1853

420DAB59-7AAF-5E94-9B7E-5F694F9CE98A

##### Distribution

Central China to Japan

#### Kadsura japonica

(L.) Dunal, 1817

008F761E-D7D1-5DDF-B2C9-0C2508802011

##### Distribution

South Korea, South Japan to Taiwan

#### Schisandra chinensis

(Turcz.) Baill., 1868

D25D7AA3-BBF7-5385-91F7-C896A07008ED

##### Distribution

Russian Far East to North China and Central Japan

#### Illicium anisatum

L., 1759

A5D29A9D-EB3D-527B-B552-563E49FBD0C1

##### Distribution

South Korea, Japan to Taiwan

#### Actinodaphne lancifolia

(Blume) Meisn., 1864

FCDF0505-3CDF-551C-BD15-E2B7FDA314CB

##### Distribution

South Korea, South Central & South Japan to Central Taiwan

#### Lindera erythrocarpa

Makino, 1897

F348C790-BA18-5A84-AA5C-14EB67B25758

##### Distribution

Central & South China to Korea, South Central & South Japan, Taiwan

#### Lindera glauca

(Siebold & Zucc.) Blume, 1851

42DF0EAB-6E49-5488-973A-9C9460E62073

##### Distribution

Central & South China to Indo-China and Korea, South Central & South Japan, Taiwan

#### Lindera obtusiloba

Blume, 1851

E69F8084-4533-5E51-96ED-46009BF85CE4

##### Distribution

Bhutan to China and Myanmar, Korea, South Central & S. Japan

#### Machilus japonica

Siebold & Zucc, 1846

E31251B3-54B3-5549-AF35-158295D1FDA1

##### Distribution

South Korea, South Central Japan to Taiwan

#### Neolitsea aciculata

(Blume) Koidz., 1918

91FDAB17-8ECC-5F8F-BA28-0BA87E813805

##### Distribution

South Korea, South Central & South Japan to Taiwan

#### Neolitsea sericea

(Blume) Koidz., 1926

FC3407E6-8E1D-5B9C-84A6-41FB084D8FC0

##### Distribution

China (Zhejiang), South Korea, Central & South Japan to Taiwan

#### Aconitum alboviolaceum

Kom., 1901

8C1CB3D8-EF0D-56DB-8EBB-05745C822CCC

##### Distribution

South Russian Far East to North & Central Korea

#### Aconitum austrokoreense

Koidz., 1934

02EC9299-8666-5830-A692-6E21D950B6AD

##### Distribution

North Korea

#### Aconitum barbatum

Patrin ex Pers., 1806

1AFE672E-EBB2-5B93-B15D-7CE08550C402

##### Distribution

Siberia to Korea

#### Aconitum coreanum

(H.Lév.) Rapaics, 1907

1934F538-AF1B-5FF1-9345-0281293BBD05

##### Distribution

Mongolia to South Russian Far East and North & Central Korea

#### Aconitum jaluense

Kom., 1901

A3EB42D0-725A-5F2C-A22F-D65A2FAB0B37

##### Distribution

South Russian Far East to North & Central Korea, Central & South Japan

#### Aconitum longecassidatum

Nakai, 1909

7E813D40-6017-5E84-8184-74D0037AF152

##### Distribution

East China to Korea

#### Aconitum pseudolaeve

Nakai, 1935

B989F66E-EAAA-5262-9E71-7F073CDF8575

##### Distribution

Korea

#### Actaea asiatica

H.Hara, 1939

7D435E14-3D5E-5AC8-92A0-C8681A5282FC

##### Distribution

Tibet to Russian Far East and Japan

#### Actaea bifida

(Nakai) J.Compton, 1998

7A7513AD-9B8F-5913-B6E6-DBD5361BEF20

##### Distribution

Korea

#### Actaea cimicifuga

L., 1753

CA93F7C9-06E9-588F-A0E1-0E5AC7A8FDFD

##### Distribution

Siberia to Korea

#### Actaea dahurica

(Turcz. ex Fisch. & C.A.Mey.) Franch., 1883

861AB384-0340-587B-8823-B2428C6627E0

##### Distribution

SouthEast Siberia to Korea

#### Anemone reflexa

Steph. ex Willd., 1799

0CFF0240-42C4-5B29-91CB-84C96033C17C

##### Distribution

East European Russia to Korea

#### Caltha palustris

L., 1753

FCEE96C6-8C43-5775-9D9B-774FCED0BAF2

##### Distribution

Temperate & Subarctic Northern Hemisphere

#### Clematis apiifolia

DC., 1817

1ABB5BBA-E284-5B8C-83ED-8FD16AB38B34

##### Distribution

Central & Southeast China, Korea, Central & South Japan

#### Clematis brachyura

Maxim., 1876

EC542BED-D0DF-5EB4-9BE4-ABBA5BD56756

##### Distribution

Korea

#### Clematis brevicaudata

DC., 1817

D74F06E3-BDA7-5BD7-ADB8-044413C6AE3B

##### Distribution

Mongolia to North Vietnam and East Korea, Japan

#### Clematis fusca

Turcz., 1840

A4508250-5370-5161-83D0-60A299A242E2

##### Distribution

East Siberia to Russian Far East and North & North Central Japan

#### Clematis fusca var. violacea

Maxim., 1859

29B0B8C6-C0D8-5F7D-AB51-67BFAB405D8B

##### Distribution

Russian Far East to North China and Central Japan

#### Clematis patens

C.Morren & Decne., 1836

6394A031-08B0-507A-A3F1-3F673550F957

##### Distribution

East China to Central & South Japan

#### Clematis serratifolia

Rehder, 1911

72DCF93B-7333-5349-B03E-324E3D570D4B

##### Distribution

Russian Far East to Korea

#### Clematis terniflora

DC., 1817

2729616A-3E1A-5542-8AA3-0B15CA32F158

##### Distribution

Central & South China to Temperate East Asia

#### Clematis terniflora var. mandshurica

(Rupr.) Ohwi, 1938

61E08BB5-90C8-5A8F-A90E-2FEB70F59805

##### Distribution

South Russian Far East to Korea

#### Clematis trichotoma

Nakai, 1912

4B311B87-CF51-54D8-ABF4-191BF61C1EAA

##### Distribution

Korea

#### Clematis urticifolia

Nakai ex Kitag., 1937

F7436D96-8CC8-5677-BA4D-5F68D579E63C

##### Distribution

China to Korea

#### Enemion raddeanum

Regel, 1861

1569BF22-6BB1-5836-AA7D-C24CC4110537

##### Distribution

Russian Far East to North & Central Korea, Central Japan

#### Eranthis stellata

Maxim., 1859

D441EE30-D051-5DE3-8AC4-D28F084B8A23

##### Distribution

Russian Far East to Korea

#### Hepatica asiatica

Nakai, 1937

09AD2E71-9CDD-5E6A-ACAC-52111D15C388

##### Distribution

South Russian Far East to North & East China to Korea, Central & South Japan

#### Pulsatilla koreana

(Y.Yabe ex Nakai) Nakai ex T.Mori, 1922

C3D97DEB-C4A9-53D7-B316-0C74802425A3

##### Distribution

Russian Far East to Korea

#### Ranunculus chinensis

Bunge, 1833

41B0C006-DC69-54C1-8739-AB88E3DB8466

##### Distribution

Siberia to Temperate East Asia and Peninsula Malaysia

#### Ranunculus japonicus

Thunb., 1794

E4359905-CAA0-5EED-B5C0-8CDEAFA6B67F

##### Distribution

Siberia to Russian Far East and Temperate East Asia

#### Ranunculus sceleratus

L., 1753

AA199F5D-F4AA-560A-9663-6E2A8D53CDE2

##### Distribution

Temperate Eurasia, North Africa, Ethiopia to Rwanda, Central & East Canada to Central & East U.S.A.

#### Ranunculus tachiroei

Franch. & Sav., 1878

9EC515B6-F1A2-5886-BAB9-8530AD0FE69B

##### Distribution

East China, Korea & Japan

#### Semiaquilegia adoxoides

(DC.) Makino, 1902

A1111CDF-D751-515B-886A-1886A46E9C29

##### Distribution

China to West Central & S. Japan

#### Thalictrum actaeifolium var. brevistylum

Nakai, 1937

6414154C-F4CD-54A6-A8EE-8E680C3122E8

##### Distribution

South Korea

#### Thalictrum aquilegiifolium var. sibiricum

Regel & Tiling, 1858

175E11DC-6A36-5252-B172-323FC12B27BD

##### Distribution

Russian Far East to North & Central China, South Korea, Japan

#### Thalictrum ichangense

Lecoy. ex Oliv., 1888

CC654270-F373-5C66-851D-A6E51241193F

##### Distribution

Central & S. China to Vietnam and Korea

#### Thalictrum kemense

Fr., 1817

37D71057-6C8B-518C-A4C7-20F390E9BDE5

##### Distribution

Subarctic to the Caucasus, North & North Central Japan

#### Thalictrum minus var. hypoleucum

(Siebold & Zucc.) Miq., 1867

ED040897-4AA6-5B73-BFE5-612103C550F0

##### Distribution

Temperate Eurasia, North West Africa

#### Thalictrum tuberiferum

Maxim., 1876

AED13ACC-40B3-5BB5-BDB6-C8F7ABBD0246

##### Distribution

South Russian Far East to Korea, Central & South Japan, Temperate Asia & Europe

#### Berberis amurensis

Rupr., 1857

5A5C102D-EBAF-54FE-8312-C63752851D10

##### Distribution

Mongolia to North China and Japan

#### Berberis koreana

Palib., 1899

27C23B33-38C1-5C83-B1F0-9EFDC869252F

##### Distribution

Korea

#### Akebia quinata

(Houtt.) Decne., 1839

0B431D53-BC35-53FC-BCF0-B7CCEED1A860

##### Distribution

Central & East China to Japan

#### Stauntonia hexaphylla

(Thunb.) Decne., 1839

DAF9CFC0-0C83-586B-9F09-997ADF544CAB

##### Distribution

South Korea, Central & South Japan

#### Cocculus orbiculatus

(L.) DC., 1817

EB6EE2DC-AD31-58A6-8EB3-D2C6C0FF14FD

##### Distribution

Himalaya to Japan (Kerama Islands) and Central Pacific

#### Menispermum dauricum

DC., 1817

F3800CA7-7243-5C6D-9D10-86A9727181B6

##### Distribution

South Siberia to China and Japan

#### Chloranthus japonicus

Siebold, 1828

63A94A38-5642-55BE-98DC-C669FE4D7982

##### Distribution

South China to Vietnam, South Korea, South Kuril Islands to Japan

#### Aristolochia contorta

Bunge, 1833

8A635670-A30D-5E09-84D8-CC7304A2C9CC

##### Distribution

South Russian Far East to Vietnam and Japan

#### Aristolochia manshuriensis

Kom., 1904

928F8376-7547-566C-B389-DAC10BF4C40F

##### Distribution

South Russian Far East to North & East Central China and Korea

#### Asarum chungbuensis

(C.S.Yook & J.G.Kim) B.U.Oh, 2005

73CFD6C2-7C29-5F32-90DB-A2689B411DE8

##### Distribution

Korea

#### Asarum maculatum

Nakai, 1914

A411F739-9067-591A-ABC1-939C643C5F29

##### Distribution

Korea

#### Asarum mandshuricum

(Maxim.) M.Kim & S.So, 2008

71A3C604-EB80-5C4E-BE99-F3BDAA543022

##### Distribution

East China to South Russian Far East and North Central Japan

#### Asarum mandshuricum var. seoulense

(Nakai) M.Kim & S.So, 2017

8FE19740-1513-5142-92FF-25BE5575BCC0

##### Distribution

East China to South Russian Far East and North Central Japan

#### Asarum misandrum

B.U.Oh & J.G.Kim, 1997

9C91612D-688D-50A0-A67D-93FA798A31EF

##### Distribution

Korea

#### Asarum sieboldii

Miq., 1865

9255E3FB-1F03-5FDB-AAE3-0C11F7F4384B

##### Distribution

South Russian Far East to Korea, Japan

#### Paeonia japonica

(Makino) Miyabe & Takeda, 1910

AB0B7AF9-80E3-511C-BBE5-7A8E5F6C9335

##### Distribution

Korea & Japan

#### Paeonia lactiflora

Pall., 1776

CAAC7254-D7DF-52D4-B3A3-0FBA71A2ABB7

##### Distribution

SouthEast. Siberia to North & East China

#### Paeonia obovata

Maxim., 1859

878D22B1-ACD0-59DC-B283-67863A2765FB

##### Distribution

China to Russian Far East and Japan

#### Actinidia arguta

(Siebold & Zucc.) Planch. ex Miq., 1867

9E265193-B62C-5C2C-B842-3876798F6286

##### Distribution

Russian Far East to China, Temperate East Asia

#### Actinidia kolomikta

(Maxim. & Rupr.) Maxim., 1859

166DDB3B-839C-5A60-A50D-5BF0108C0EDF

##### Distribution

Russian Far East to Central China, North & Central Japan

#### Actinidia polygama

(Siebold & Zucc.) Planch. ex Maxim., 1859

F6501FC9-E1EE-53BE-BC56-300ABBC3D1AF

##### Distribution

South Russian Far East to China, Korea, Japan

#### Camellia japonica

L., 1753

58E8E586-6E1E-58FC-8767-8C3EF625EE9A

##### Distribution

China, Korea, Central & South Japan to Taiwan

#### Eurya japonica

Thunb., 1783

8D4EC025-F4FF-5092-8419-B91B269BAA17

##### Distribution

China to Temperate East Asia

#### Stewartia koreana

Nakai ex Rehder, 1926

1375F4C7-0C78-5063-9354-2633BF68EC70

##### Distribution

Korea & Japan

#### Hypericum ascyron

L., 1753

58DC4B61-EB94-5159-96BE-BDADE713EC8F

##### Distribution

Temperate Asia, East Canada to North Central & East U.S.A.

#### Hypericum erectum

Thunb., 1784

3266F4D9-2336-58FD-9873-425E27355A8B

##### Distribution

South China, South Sakhalin to Temperate East Asia

#### Chelidonium majus asiaticum

H.Hara, 1949

A5B549AE-CC7E-5A91-8F2F-18A1993ED6E7

##### Distribution

Russian Far East to North China and Japan

#### Corydalis alata

B.U.Oh & W.R.Lee, 2010

7EB7640D-1EFC-5A79-AC07-5995F4A5B4CA

##### Distribution

NorthEast. China, Central Korea

#### Corydalis incisa

(Thunb.) Pers., 1806

1DB2DEB0-A5BF-54E5-B87E-64096D3576EA

##### Distribution

Central & East China to Temperate East Asia

#### Corydalis maculata

B.U.Oh & Y.S.Kim, 1987

E7B2CFDC-4925-5A4D-BC0F-EF967109BF6F

##### Distribution

Korea

#### Corydalis namdoensis

B.U.Oh & J.G.Kim, 2004

E904993D-B161-53AF-AEEF-51B9E3EA76CE

##### Distribution

NorthEast. China, South Korea

#### Corydalis ochotensis

Turcz., 1840

AA7C472A-4BBB-5055-B28E-39FFA37D667A

##### Distribution

Russian Far East to North China

#### Corydalis pauciovulata

Ohwi, 1942

01FEF5C9-86A1-5D8A-8F81-D2CE9B9767A5

##### Distribution

Central & South Korea, Central Japan

#### Corydalis raddeana

Regel, 1862

120EF416-D4DB-5AEA-A413-52DEEB28F17C

##### Distribution

Russian Far East to North & East China and Temperate East Asia

#### Corydalis remota

Fisch. ex Maxim., 1859

1ACCF6A8-C83D-5877-A4D7-B55E66995B31

##### Distribution

Southeast Siberia to North China

#### Corydalis speciosa

Maxim., 1859

13F9CCEC-BDE6-5D8C-8923-3D710A572AF7

##### Distribution

Russian Far East to China and Korea, North & North Central Japan

#### Corydalis turtschaninovii

Besser, 1834

30C8E56D-E471-5D6F-A983-FE5154D4ED9E

##### Distribution

Southeast Siberia to North China

#### Dicentra spectabilis

(L.) Lem., 1847

D0253002-6AF5-5264-8F79-AB817FFB78C9

##### Distribution

NorthEast China to North Korea

#### Hylomecon vernalis

Maxim., 1859

90ECAF81-6498-5191-96C8-85356BAE2245

##### Distribution

Russian Far East to China and Central & South Korea

#### Arabidopsis halleri gemmifera

(Matsum.) O'Kane & Al-Shehbaz, 1997

FC91D221-A7C1-5D20-B62F-5214D6E88022

##### Distribution

East Siberia to Temperate East Asia

#### Arabis hirsuta

(L.) Scop., 1771

B36247F7-209E-5965-86CE-D43DD56B48C7

##### Distribution

Temperate Eurasia, Algeria

#### Barbarea orthoceras

Ledeb., 1824

924544EC-DD89-52C1-92C7-3647B2AD53C9

##### Distribution

Temperate Asia, Subarctic America to North, West & Central U.S.A.

#### Barbarea vulgaris

W.T.Aiton, 1812

40A23B6D-BB26-57AD-A938-F93A0E476E1B

##### Distribution

Europe, Mediterranean to Japan

#### Berteroella maximowiczii

(Palib.) O.E.Schulz, 1919

1F330309-F8D6-5FEA-9EEF-C18FEC856FA2

##### Distribution

North & East China to Korea & Japan

#### Capsella bursa-pastoris

(L.) Medik., 1792

B2778978-99A8-5EBC-AB42-12E757C19DE3

##### Distribution

Temperate Eurasia, North Africa

#### Cardamine fallax

(O.E.Schulz) Nakai, 1919

1C1956C4-A9BB-5F9C-B95C-35E9C5E84611

##### Distribution

Temperate Eurasia, Subarctic America to North, Central & East U.S.A.

#### Cardamine flexuosa

With., 1796

547099E1-5961-5599-86F1-14E0A512E05E

##### Distribution

Europe to Iran, NorthWest Africa

#### Cardamine impatiens

L., 1753

C07F9423-F381-5BB8-B5C7-9555F919E612

##### Distribution

Temperate Eurasia

#### Cardamine komarovii

Nakai, 1914

B9577059-C30E-5AB2-BC84-23A9CEE03C97

##### Distribution

NorthEast China to Korea

#### Cardamine leucantha

(Tausch) O.E.Schulz, 1903

031E5796-9CE0-5D61-9D84-62E5481CDCFE

##### Distribution

SouthEast Siberia to Japan and China

#### Catolobus pendulus

(L.) Al-Shehbaz, 2005

B8CF42D6-1A34-5487-8967-789AC3DB7A17

##### Distribution

East Europe to Japan

#### Draba nemorosa

L., 1753

5D38D502-6636-57C2-8214-165C32510F70

##### Distribution

Subarctic and Temperate Northern Hemisphere

#### Erysimum amurense

Kitag., 1937

D90B5402-F267-5196-A8C3-65B54EECBB12

##### Distribution

South Siberia to North China

#### Lepidium apetalum

Willd., 1800

37F0DDAC-8B6F-5CDA-B125-1CCF8C092949

##### Distribution

East Europe to Temperate Asia

#### Rorippa palustris

(L.) Besser, 1821

4F6BC2FE-00DE-5181-A542-98970B698E62

##### Distribution

Temperate Northern Hemisphere to Tropical Mountains

#### Thlaspi arvense

L., 1753

A6F1F36C-E038-5AC9-9D69-87B5B2B4465C

##### Distribution

Temperate Eurasia

#### Turritis glabra

L., 1753

D09940D5-0AD5-5B05-8A97-6346799BD7DC

##### Distribution

Temperate Northern Hemisphere to Tropical Mountains

#### Hylotelephium erythrostictum

(Miq.) H.Ohba, 1977

17D57954-38E0-5708-BD83-8925BF825DC3

##### Distribution

China to Korea, South Sakhalin to Japan

#### Hylotelephium viviparum

(Maxim.) H.Ohba, 1977

9BC05FE3-A55C-5DF6-96CA-301E0114626C

##### Distribution

South Russian Far East to Korea

#### Orostachys japonica

(Maxim.) A.Berger, 1930

E5FD26A8-8DD9-5FD7-B160-0AA131243644

##### Distribution

East China to Korea, South Central & South Japan

#### Orostachys minuta

(Kom.) A.Berger, 1930

77C854CA-E0AB-5E0C-90DD-E5334A6B5915

##### Distribution

NorthEast China to Korea

#### Phedimus aizoon

(L.) 't Hart, 1995

992BA6BB-CA8B-54F6-BFBA-06975A6DC2C9

##### Distribution

Siberia to China and North & Central Japan

#### Phedimus kamtschaticus

(Fisch. & C.A.Mey.) 't Hart, 1995

B132003A-3C2E-52E5-8312-383E07B164FD

##### Distribution

Russian Far East to North China and North Japan

#### Phedimus middendorffianus

(Maxim.) 't Hart, 1995

C71B6259-2EF3-56D2-BDD5-1A8F31604D90

##### Distribution

East Siberia to Korea, Japan

#### Sedum bulbiferum

Makino, 1891

75B35923-C90F-579F-A044-426953B00E7C

##### Distribution

Central China to Korea, Central & South Japan to Taiwan

#### Sedum polytrichoides

Hemsl., 1887

5205954B-A03D-5BB2-82EF-C689935D4644

##### Distribution

North & East China to Korea, Japan

#### Sedum sarmentosum

Bunge, 1835

D2B2C15F-9002-56E0-BFC6-CA045EF328B7

##### Distribution

Thailand to Japan

#### Astilbe chinensis

(Maxim.) Franch. & Sav., 1873

84EE7078-6AB9-53F3-B2A8-1D109055FCE8

##### Distribution

Russian Far East to China and South Japan

#### Astilboides tabularis

(Hemsl.) Engl., 1919

F70148B3-44AB-59D8-8E57-74B4F9AEC9F8

##### Distribution

NorthEast China to North Korea

#### Chrysosplenium flagelliferum

F.Schmidt, 1868

07879D60-4061-54D3-AD84-3CE36038FA6E

##### Distribution

Russian Far East to Korea, Japan

#### Chrysosplenium grayanum

Maxim., 1877

A52FCA8C-E68F-5B22-A7F0-2C648BF3F6F2

##### Distribution

East Central Korea, Sakhalin to Japan

#### Chrysosplenium japonicum

(Maxim.) Makino, 1909

64A27A8B-4124-52DE-8AC3-F9452C8D7E42

##### Distribution

East China to Korea, Japan, NorthEast Taiwan

#### Micranthes octopetala

(Nakai) Y.I.Kim & Y.D.Kim, 2015

EDD473DB-BD63-55DF-B415-13E61016A2F9

##### Distribution

Russian Far East to Korea

#### Mukdenia rossii

(Oliv.) Koidz., 1935

2AB235D9-D7C1-58E2-B4D5-EA0370F2BD73

##### Distribution

NorthEast China to Korea

#### Rodgersia podophylla

A.Gray, 1859

9FC636B9-CECA-5CBF-A591-A77BF6C23C1C

##### Distribution

NorthEast. China to Korea, Japan

#### Saxifraga fortunei

Hook., 1863

6A51EF87-AD01-510A-BBA6-11DBD1E6C1E0

##### Distribution

South Central China, South Russian Far East to Korea, Japan

#### Saxifraga stolonifera

Curtis, 1774

299D6499-18DB-51F7-8072-98BED18404A2

##### Distribution

Central & South China, South Korea, Central & South Japan, Taiwan

#### Deutzia glabrata

Kom., 1903

B910476E-6D96-528A-AC2C-5ED53B3DF437

##### Distribution

South Russian Far East to North China

#### Deutzia grandiflora var. baroniana

(Diels) Rehder, 1911

C8247DF2-B155-516B-8EDA-58AF8FF7A800

##### Distribution

North Central & East China

#### Deutzia paniculata

Nakai, 1913

7609B705-F6DF-5117-8AC2-78F1433FA07E

##### Distribution

Korea

#### Deutzia parviflora

Bunge, 1835

2223C49A-4FFC-539D-B2F7-4FE07A2E5C82

##### Distribution

South Russian Far East to China and Korea

#### Deutzia uniflora

Shirai, 1898

56BB4545-5BFB-5D2B-826E-7CF1FF833631

##### Distribution

Central & South Korea, Central Japan

#### Hydrangea macrophylla serrata

(Thunb.) Makino, 1929

BBD1828D-AE38-5252-B532-D0D736ECD255

##### Distribution

Korea & Japan

#### Hydrangea petiolaris

Siebold & Zucc., 1840

B22B561A-DB9A-5441-BA30-2BC2AC0F79EC

##### Distribution

Korea, Sakhalin to Japan

#### Philadelphus schrenkii

Rupr., 1857

1BDFF491-63B4-5FA6-B97D-DE7008D9F41B

##### Distribution

South Russian Far East to North China and Korea

#### Philadelphus tenuifolius

Maxim. & Rupr., 1856

015F1910-5109-5F9B-9121-F076AB9A3DAF

##### Distribution

Russian Far East to Korea

#### Schizophragma hydrangeoides

Siebold & Zucc., 1837

2E9028D6-6ABF-5F17-B691-64DC3F3FEE12

##### Distribution

Korea to Japan

#### Parnassia palustris

L., 1753

B5AF7DD3-1C9B-580A-9A77-641E139260C6

##### Distribution

Subarctic & Temperate Northern Hemisphere

#### Ribes fasciculatum var. chinense

Maxim., 1874

537ED9A3-911C-5D38-A7E8-46A6472AABC9

##### Distribution

Central & East China to Korea, Central & South Japan

#### Ribes komarovii

Pojark., 1926

D02D9451-B77C-5FC2-AB0E-506388B8C02E

##### Distribution

South Russian Far East to North China and North Korea

#### Ribes mandshuricum

(Maxim.) Kom., 1904

F0F07CC7-A6E7-5122-BF79-5D9921B54B54

##### Distribution

Russian Far East to North & East Central China and Korea

#### Ribes maximowiczianum

Kom., 1904

5913310C-2D0D-589C-B6A9-8CB7A2AC5A90

##### Distribution

Siberia to China, Korea & Japan

#### Agrimonia coreana

Nakai, 1918

EE70773E-6216-555F-A4E6-2303360475CF

##### Distribution

South Russian Far East to North & East China, Japan

#### Agrimonia pilosa

Ledeb., 1823

35F674C4-F1B9-51C6-9C4B-360697BC0837

##### Distribution

North & East Central Europe to Japan and North Indo-China

#### Aria alnifolia

(Siebold & Zucc.) Decne., 1874

6E611C4B-244E-5652-BFDC-498D79565BBA

##### Distribution

China to South Russian Far East and Temperate East Asia

#### Aruncus dioicus

(Walter) Fernald, 1939

C73EC4BA-EF87-5835-9D67-2B0093A895E2

##### Distribution

Europe to the Caucasus, East Siberia to Russian Far East, North Central & East U.S.A.

#### Crataegus pinnatifida

Bunge, 1835

011AEDB8-D430-5E26-8E8C-EFA1F4CE250C

##### Distribution

Russian Far East to North & East China

#### Duchesnea chrysantha

(Zoll. & Moritzi) Miq., 1855

E87F4287-A2A7-5087-A76F-D1AC557856F6

##### Distribution

SouthEast China to Temperate East Asia and Central & South Malaysia

#### Duchesnea indica

(Andrews) Teschem., 1835

E4AF1CA3-9E4F-5A43-A1A2-4653935920F9

##### Distribution

Afghanistan to Russian Far East and Malaysia

#### Exochorda serratifolia

S.Moore, 1877

E011005A-F868-5344-862E-EC900C180943

##### Distribution

South Russian Far East to North China and Korea

#### Filipendula glaberrima

Nakai, 1914

23B9514F-7E37-5B2A-988D-D7C2A596BFE7

##### Distribution

Primorye to North China and Korea, Sakhalin to North Japan

#### Geum aleppicum

Jacq., 1781

B037F539-8E13-54AA-BFAB-40F0D01002A2

##### Distribution

Temperate Northern Hemisphere to Mexico

#### Geum japonicum

Thunb., 1784

623B960E-7ECC-5379-864A-C9E0A9E97A6F

##### Distribution

China to Korea & Japan

#### Malus baccata

(L.) Borkh., 1803

0AAECCE6-6220-5136-AF32-D1E03C696FB6

##### Distribution

Siberia to Korea and Himalaya

#### Malus mandshurica

(Maxim.) Kom. ex Skvortsov, 1925

C2E4A41B-270D-5D15-A9A0-C7FD3A0A9663

##### Distribution

Russian Far East to North China, North & Central Japan

#### Potentilla chinensis

Ser., 1825

3B1CFD96-3F2F-5C8E-A3C3-402597FD2598

##### Distribution

Mongolia to Russian Far East and Temperate East Asia

#### Potentilla cryptotaeniae

Maxim., 1873

7B3660C6-917B-5CFF-A33F-D14204E4A265

##### Distribution

Russian Far East to Central China and Japan

#### Potentilla dickinsii

Franch. & Sav., 1878

AB8D717B-017B-54CB-B9A8-E883F509D78D

##### Distribution

North & East China to Sakhalin and Japan

#### Potentilla discolor

Bunge, 1833

AA2261F8-0754-5DA2-96E9-0617595FDEF7

##### Distribution

South Russian Far East to China and Central & South Japan

#### Potentilla fragarioides var. major

Maxim., 1859

B2B755BE-6C83-55F9-8E69-F97483181491

##### Distribution

Mongolia to Russian Far East and Temperate East Asia

#### Potentilla freyniana

Bornm., 1905

A9256ADB-59BB-5AC3-9180-A848DD6008FA

##### Distribution

Russian Far East to China and Temperate East Asia

#### Potentilla nivea

L., 1753

BA4778CE-6E0F-5347-90CA-F3BF78314BA7

##### Distribution

Subarctic & Subalpine

#### Pourthiaea villosa

(Thunb.) Decne., 1874

92BCD151-3061-5EA8-8868-ED8D147A7EB5

##### Distribution

Central & South China to Korea, Japan, North Taiwan

#### Prunus armeniaca

L., 1753

C07A1B57-E3EB-5C4F-A833-FFD346A12610

##### Distribution

Central Asia to North & Central China

#### Prunus buergeriana

Miq., 1865

D6E15040-2CC4-529A-86A1-D5FFE8289CCA

##### Distribution

Himalaya to Korea & Central & South Japan

#### Prunus choreiana

Nakai ex H.T.Im, 2006

4EC465E8-911C-5D62-A322-7550CD92D7D1

##### Distribution

Korea

#### Prunus davidiana

(Carrière) Franch., 1884

BE5B22E9-58A8-5521-8E81-695FC2C0B484

##### Distribution

North Central China to Korea

#### Prunus japonica var. nakaii

(H.Lév.) Rehder, 1922

5672637E-5D00-51B0-BEA8-C4446BB53E3C

##### Distribution

NorthEast China to Korea

#### Prunus mandshurica

(Maxim.) Koehne, 1893

80771523-3F59-5F60-867F-421A9377C442

##### Distribution

South Russian Far East to Korea

#### Prunus padus

L., 1753

441271AC-747C-5CD4-A55F-39604EA6A512

##### Distribution

Temperate Eurasia, Morocco

#### Prunus persica

(L.) Batsch, 1801

B7B95726-50E5-5B25-8F0B-6E4567F67D54

##### Distribution

North Central China

#### Prunus sargentii

Rehder, 1908

30F87F61-8492-57E2-8C12-04C268CE83A3

##### Distribution

Russian Far East to Japan, Korea

#### Prunus sargentii var. verecunda

(Koidz.) Chin S.Chang, 2004

CADCB7D0-BD7B-5829-A6FB-B3449FACEB80

##### Distribution

North & East China to Korea, Japan

#### Prunus serrulata var. pubescens

(Makino) Nakai, 1915

F90130C6-A85E-5C93-B7E3-8093C1D1737F

##### Distribution

North & East China to Korea, Japan

#### Prunus serrulata f. spontanea

(E.H.Wilson) Chin S.Chang, 2007

B17F0403-F029-513B-B99C-18C9EBECBC99

##### Distribution

Central & South Japan

#### Prunus sibirica

L., 1753

06EE7806-AED5-5BAC-A362-E2B41C486E8C

##### Distribution

South Siberia to North China and Korea

#### Prunus tomentosa

Thunb., 1784

995F200C-8C31-519E-985D-7C74631D9FC5

##### Distribution

Tibet to China, Sakhalin

#### Prunus yedoensis

Matsum., 1901

3DB76253-4696-53A5-B963-1FD80A2C6B49

##### Distribution

Japan, Korea

#### Pyrus calleryana var. fauriei

(C.K.Schneid.) Rehder, 1920

6237C2D3-C648-54A6-8305-4889D04CBEAD

##### Distribution

Korea, Japan & Taiwan

#### Pyrus ussuriensis

Maxim. ex Rupr., 1856

2B36B1F5-24EC-5D93-BBA0-E1735A3DF847

##### Distribution

North & East China to Korea, Russian Far East to North Japan

#### Rhaphiolepis indica var. umbellata

(Thunb. ex Murray) H.Ohashi, 1988

9FC97315-1264-5DCF-BA21-F5783EF563CB

##### Distribution

China to Temperate East Asia

#### Rosa acicularis

Lindl., 1820

047C2246-1D90-54C7-AF85-CE93850CE1F0

##### Distribution

North Europe to North & Central Japan, Central Alaska

#### Rosa davurica

Pall., 1788

85C18913-E7B8-5AD9-AA44-B4B1103F6B30

##### Distribution

Siberia to Russian Far East and Korea

#### Rosa koreana

Kom., 1901

8845499D-E93D-5CA9-BDF9-D053CECC2132

##### Distribution

Russian Far East to Korea

#### Rosa lucieae

Franch. & Rochebr. ex Crép., 1871

DC5857C8-5318-5294-928B-7F981C6E031E

##### Distribution

SouthEast China, South Korea, Central & South Japan to Philippines

#### Rosa multiflora

Thunb., 1784

5C4BB17A-B281-538E-AB00-C018D80EECED

##### Distribution

East China to Korea, Japan

#### Rubus corchorifolius

L.f., 1782

31431C43-6699-5F8D-B31F-20BB5F9E4DAC

##### Distribution

China to North Indo-China, Korea, Japan

#### Rubus coreanus

Miq., 1867

02CD2C50-5BE5-5A7E-854E-49B2C4834C27

##### Distribution

North & East China to Korea

#### Rubus crataegifolius

Bunge, 1833

7EA0F9D8-1F52-549A-A84A-FF5302284038

##### Distribution

South Siberia to Korea, Japan

#### Rubus idaeus var. microphyllus

Turcz., 1843

12EE4EC6-1E56-5B6D-AD83-CC44A5293E45

##### Distribution

Subarctic to North & Central China and Mexico

#### Rubus parvifolius

L., 1753

957D7DA2-BC4A-58B8-9EA9-A53332AECDA0

##### Distribution

China to Vietnam, Sakhalin to Temperate East Asia, East & South East Australia

#### Rubus phoenicolasius

Maxim., 1872

1E50F700-6353-5FA3-80D1-629860A0F1C7

##### Distribution

China (Qinghai) to Korea, Japan

#### Rubus pungens

Cambess., 1844

57C43688-E613-5911-8478-AD1A41391792

##### Distribution

North Pakistan to Primorye and Temperate East Asia

#### Sanguisorba hakusanensis

Makino, 1907

D7D5A584-8175-5D8E-B9F7-9EA1DF251AD0

##### Distribution

Korea & Japan

#### Sanguisorba officinalis

L., 1753

A1E8AD6F-5AB7-528A-887C-25CB73FA2F49

##### Distribution

Temperate Northern Hemisphere

#### Sorbaria sorbifolia var. stellipila

Maxim., 1879

AE3D46E6-A1DB-5950-99A5-34BC4D378FBB

##### Distribution

NorthEast China to Korea, Japan

#### Sorbus commixta

Hedl., 1901

93E05EA9-3170-5448-8852-54DA6799CF0B

##### Distribution

Central China to Korea, Sakhalin to Japan

#### Spiraea blumei

G.Don, 1832

3ADDD1C4-B08A-558B-BD3D-85108E01E389

##### Distribution

China to Korea, Central & South Japan

#### Spiraea chamaedryfolia

L., 1753

9E596BFB-DA98-5A04-AB9C-DD54AD49A48E

##### Distribution

SouthEast Europe to Korea, Japan

#### Spiraea chamaedryfolia var. pilosa

(Nakai) H.Hara, 1952

5A39D86D-CD7F-52EC-AC9D-619E7179F072

##### Distribution

SouthEast Europe to Korea, Japan

#### Spiraea chartacea

Nakai, 1928

23866886-B2B1-5CEA-A3F0-953DF4F6BBD2

##### Distribution

Korea & Japan

#### Spiraea chinensis

Maxim., 1879

5CDC53F3-1DC3-5108-B653-98488F7357E6

##### Distribution

China to Korea

#### Spiraea fritschiana

C.K.Schneid., 1905

F17DE247-CE55-519E-8773-E9C2E910128A

##### Distribution

China to Korea

#### Spiraea prunifolia f. simpliciflora

Nakai, 1909

81E94034-E8A6-5E09-A7BB-652223086B25

##### Distribution

South China

#### Spiraea salicifolia

L., 1753

15906F90-C96A-5878-B38C-67DD646BFDC7

##### Distribution

East Central Europe to North & Central Japan

#### Spiraea trichocarpa

Nakai, 1909

4D84CBD2-9955-57CA-AC20-8871F78A4066

##### Distribution

North China to Korea

#### Stephanandra incisa

(Thunb.) Zabel, 1885

2EE4D07A-1F74-5AA0-B0FB-33706ECAC897

##### Distribution

China to Temperate East Asia

#### Albizia julibrissin

Durazz., 1772

04044246-6253-57D3-AAF9-659935658467

##### Distribution

East Transcaucasus to Japan

#### Amorpha fruticosa

L., 1753

A5C7A7B4-4979-5C9C-8828-15C7793D1C56

##### Distribution

U.S.A. to North Mexico

#### Amphicarpaea bracteata edgeworthii

(Benth.) H.Ohashi, 1966

03926D56-2584-5FFE-BCAA-3A22EDB3ABBB

##### Distribution

Himalaya to Russian Far East and Temperate East Asia

#### Astragalus penduliflorus var. dahuricus

(DC.) X.Y. Zhu, 2005

BF6E2754-D028-58A9-BD25-2DB3A90A8A8D

##### Distribution

Siberia to Russian Far East and West & North China

#### Caragana fruticosa

(Pall.) Besser, 1816

2BB5C58F-ADBD-555A-873A-D530CCE8958B

##### Distribution

Central Asia to Siberia and NorthEast China

#### Chamaecrista nomame

(Makino) H.Ohashi, 1989

C0CE0BAF-B916-5AFD-AD6C-1D6CA39DD7F0

##### Distribution

Eritrea to Zimbabwe, Madagascar, Tropical & Subtropical Asia to North & East Australia

#### Glycine max soja

(Siebold & Zucc.) H.Ohashi, 1982

9ABDF868-4E5B-567B-9D26-11A765F55AEB

##### Distribution

Russian Far East to China and Temperate East Asia

#### Hylodesmum oldhamii

(Oliv.) H.Ohashi & R.R.Mill, 2000

FE033190-5988-5E2B-81C7-1D1C7DB9AF11

##### Distribution

South Russian Far East to China and Central & South Japan

#### Hylodesmum podocarpum

(DC.) H.Ohashi & R.R.Mill, 2000

BCAE3F53-D835-5FBF-B0C4-C20A50F88F02

##### Distribution

Himalaya to Temperate East Asia and Philippines

#### Hylodesmum podocarpum fallax

(Schindl.) H.Ohashi & R.R.Mill, 2000

931D0615-ABB3-51E5-91E8-D50983D769F4

##### Distribution

Central & South China to Central & South Japan

#### Hylodesmum podocarpum oxyphyllum

(DC.) H.Ohashi & R.R.Mill, 2000

442E31B4-8AF6-5957-91CA-F1CAF2560A1E

##### Distribution

Himalaya to South Russian Far East and Temperate East Asia

#### Hylodesmum podocarpum var. mandshuricum

(Maxim.) H.Ohashi & R.R.Mill, 2000

E0A83C3B-B15F-578E-BD5F-E3BE25C6AB79

##### Distribution

Himalaya to South Russian Far East and Temperate East Asia

#### Indigofera grandiflora

B.H.Choi & S.K.Cho, 1996

D097B402-E678-57EC-ACD2-8F147234BCC0

##### Distribution

Korea

#### Indigofera kirilowii

Maxim. ex Palib., 1899

9D8CBC35-A1D6-576B-8A2B-A0C54322F846

##### Distribution

China to Korea, Japan

#### Indigofera pseudotinctoria

Matsum., 1902

9C3C64B1-0B79-5782-B8C0-B2185F7ECA2B

##### Distribution

China to Temperate East Asia

#### Kummerowia stipulacea

(Maxim.) Makino, 1914

E3AA5752-09C0-5610-AF89-AB8BC9B157B5

##### Distribution

Russian Far East to China and Temperate East Asia

#### Kummerowia striata

(Thunb.) Schindl., 1912

065BF8B3-5493-50FD-888A-86B191C531BF

##### Distribution

Russian Far East to North Indo-China

#### Lathyrus davidii

Hance, 1871

9200D977-0002-53C9-894D-9C0562E9ED6D

##### Distribution

Russian Far East to China and Korea, Japan

#### Lespedeza bicolor

Turcz., 1840

58FDD2DF-D421-5CE1-BEAA-16E73503A41B

##### Distribution

SouthEast Siberia to Korea, Japan

#### Lespedeza cuneata

(Dum.Cours.) G.Don, 1832

44BA9129-2125-5F24-8C3E-2A425CA33DEC

##### Distribution

Afghanistan to Japan and Tropical Asia, East & South East Australia

#### Lespedeza cyrtobotrya

Miq., 1867

4F030DCB-579E-5774-A510-C7E1AB078CC5

##### Distribution

South Russian Far East to North & East China and Central & South Japan

#### Lespedeza juncea

(L.f.) Pers., 1807

F7C607F6-4230-5AFB-8958-C12351E48646

##### Distribution

Temperate Asia to North India

#### Lespedeza maritima

Nakai, 1923

E7F89353-E86A-536E-A73F-6DA95BF152D5

##### Distribution

Korea

#### Lespedeza maximowiczii

C.K.Schneid., 1907

48A49BDA-5647-534C-9D27-CCA8AFEED1B3

##### Distribution

Korea & Japan

#### Lespedeza maximowiczii var. tomentella

(Nakai) Nakai, 1927

B5E004D8-226D-5EC7-B72E-38206C532AC0

##### Distribution

Korea

#### Lespedeza virgata

(Thunb.) DC., 182

C190EAAA-4F13-57C6-8771-1E7D8D88F402

##### Distribution

China to Temperate East Asia

#### Lotus corniculatus var. japonica

Regel, 1865

84A9C309-3F6D-572D-8F6D-B697F7A73365

##### Distribution

Temperate East Asia

#### Maackia amurensis

Rupr., 1856

23167C84-227E-5F89-AFD7-ABDB5B8008C6

##### Distribution

Russian Far East to North China and Japan

#### Medicago lupulina

L., 1753

15F22DE7-1F30-5D1A-A701-F632FEC19EC7

##### Distribution

Macaronesia, Europe to the Caucasus, North & Northeast Tropical Africa to Arabian Peninsula Indian Subcontinent to China

#### Medicago sativa

L., 1753

D93E221C-B3A7-5137-84A4-8302084D7C7E

##### Distribution

Mediterranean to West Siberia and Iran

#### Melilotus albus

Medik., 1787

64FAC103-6EF9-5925-94E7-FE404DCAFEEF

##### Distribution

Europe to China, North Africa to Myanmar, Ethiopia to South Africa

#### Pueraria lobata

(Willd.) Ohwi, 1947

EF4E9EDB-9A23-5747-BDEE-0A00910FDB20

##### Distribution

China to North Australia

#### Robinia pseudoacacia

L., 1753

E89B38AA-13F2-5608-B58C-F47D76C84C07

##### Distribution

East Central & East U.S.A

#### Sophora flavescens

Aiton, 1789

09D636A9-60D5-5144-BB3B-C9F7A2EC58ED

##### Distribution

Siberia to Temperate East Asia

#### Sophora koreensis

Nakai, 1919

77217D28-4AB1-59A3-8274-168C3F9902A6

##### Distribution

Korea

#### Styphnolobium japonicum

(L.) Schott, 1829

AE6D4648-72AF-514B-A772-D6C67F321293

##### Distribution

Central & South China

#### Trifolium dubium

Sibth., 1794

A3523EB0-B55F-554F-9089-3ADD6BECCB1E

##### Distribution

Macaronesia, Europe to Mediterranean and Caucasus

#### Trifolium pratense

L., 1753

45C62ACB-24B8-510A-BA46-895FB64F2A69

##### Distribution

Macaronesia, NorthWest Africa, Europe to Mongolia and Himalaya

#### Trifolium repens

L., 1753

DBA5698B-98D0-550B-B92C-5096F75BA132

##### Distribution

Macaronesia, NorthWest. Africa, Egypt to Zimbabwe, Europe to Mongolia and Himalaya

#### Vicia amoena

Fisch. ex Ser., 1825

54BD6C8D-FAD8-5472-8B2F-72951F36A49C

##### Distribution

Temperate Asia

#### Vicia amurensis

Oett., 1906

56DAA274-0B0B-5BF8-8EF4-4C07CE873842

##### Distribution

South Siberia to Korea, Japan

#### Vicia chosenensis

Ohwi, 1936

9FD6AD7A-52E8-57B4-9623-1FFB257EB757

##### Distribution

Korea

#### Vicia cracca

L., 1753

439B7346-2ADE-5532-8854-B04C0678B817

##### Distribution

Temperate Eurasia

#### Vicia hirsuta

(L.) Gray, 1822

80CC0AC4-D6F9-5822-A2CD-8A152F15A49A

##### Distribution

Macaronesia, Temperate Eurasia, North Africa to Tanzania

#### Vicia sativa nigra

Ehrh., 1780

C9A5F4EB-0968-5B62-91A1-7C30C3A81804

##### Distribution

Macaronesia, Temperate Eurasia, North Africa to Kenya

#### Vicia unijuga

A.Braun, 1853

58A4130E-4F0F-5A27-9171-3E00FC4799D3

##### Distribution

Temperate Asia

#### Vicia venosa

(Willd. ex Link) Maxim., 1872

E900ACAD-880F-552D-B8DC-D93177164E31

##### Distribution

Siberia to China and Japan

#### Vicia venosa var. cuspidata

Maxim., 1886

4C65F3B4-9DAB-58A9-8193-C62370366905

##### Distribution

South China to Korea, Central Japan

#### Vigna angularis var. nipponensis

(Ohwi) Ohwi & H.Ohashi, 1969

ADEFF87F-7123-56B9-8183-080123126F6F

##### Distribution

Nepal to South China and North Indo-China, Taiwan, Central & South Japan

#### Wisteria floribunda

(Willd.) DC., 1825

F448B581-AE7F-5E74-974A-BF3E54073A95

##### Distribution

South Central & South Japan

#### Oxalis acetosella

L., 1753

2E23B915-DE76-5166-ADFC-F4712B8D00AF

##### Distribution

Europe to Japan, Algeria

#### Oxalis corniculata

L., 1753

CBD76E65-62DE-5D9D-83B1-28DA69B8B8D6

##### Distribution

Mexico to Venezuela and Peru, Caribbean

#### Oxalis obtriangulata

Maxim., 1868

E5BC5AAA-183F-5D03-999B-0444D6495286

##### Distribution

Russian Far East to Korea, Japan

#### Oxalis stricta

L., 1753

53E33A9D-F043-5D65-93C1-77775379CE00

##### Distribution

Central & East China to North & Central Japan, North America

#### Geranium koreanum

Kom., 1901

CBAFFD51-AF94-566C-AFB8-8C7212D2BD67

##### Distribution

East China to Korea

#### Geranium krameri

Franch. & Sav., 1878

58A4054F-1FB1-5860-85B5-78A9D9EC7206

##### Distribution

Russian Far East to Korea, Central & South Japan

#### Geranium sibiricum

L., 1753

9813CB2E-AECB-536F-95D1-90BE307ED666

##### Distribution

Romania to Temperate Asia

#### Geranium thunbergii

Siebold & Zucc., 1845

113543C2-4180-5265-89E4-6CDF5488DAD4

##### Distribution

China, Kuril Islands to Temperate East Asia

#### Acalypha australis

L., 1753

84020775-5CAF-5B38-84C5-C3EBCBC37685

##### Distribution

South Russian Far East to North Philippines

#### Euphorbia humifusa

Willd. ex Schltdl., 1814

6EF62120-1AD9-5AF2-854C-35BA8D1E7186

##### Distribution

Moldova to Temperate Asia

#### Euphorbia hypericifolia

L., 1753

9102EA0C-D155-52E4-AFF3-1C650BB19B63

##### Distribution

Tropical & Subtropical America

#### Euphorbia maculata

L., 1753

14BCE787-2DD4-598C-BE31-5DF0E73CAA32

##### Distribution

SouthEast Canada to Belize, Cuba, Bahamas

#### Euphorbia sieboldiana

Morren & Decne., 1836

3CB2A6A4-B702-5890-A7DA-C99C3552AAE0

##### Distribution

China to Russian Far East and Temperate East Asia

#### Mallotus japonicus

(L.f.) Müll.Arg., 1865

355C0F60-2CCA-5DA8-ACA2-E61DD172AF3B

##### Distribution

China to Temperate East Asia

#### Mercurialis leiocarpa

Siebold & Zucc., 1845

7127E354-A346-5932-A053-2395CA29D5FC

##### Distribution

Nepal to Temperate East Asia

#### Neoshirakia japonica

(Siebold & Zucc.) Esser, 1998

AD392E10-4DAC-5050-B02F-40F593147E2F

##### Distribution

South & East China, Korea (including Jeju-do), Central & South Japan to Nansei-shoto

#### Phyllanthus ussuriensis

Rupr. & Maxim., 1857

33782B4F-7EF9-531F-A18B-DB329BDA87B5

##### Distribution

Mongolia to China and Temperate East Asia

#### Securinega suffruticosa

(Pall.) Rehder, 1932

C7121AD9-760E-56AF-8DDA-C34A0D977649

##### Distribution

Temperate Asia

#### Dictamnus dasycarpus

Turcz., 1842

5CE27A8E-C5E3-52CD-9059-22D19809FAD0

##### Distribution

South East Siberia to China and Korea

#### Orixa japonica

Thunb., 1783

66641559-243E-567F-8D4A-48CECD37ADC4

##### Distribution

Central & South China to South Korea, Central & South Japan

#### Phellodendron amurense

Rupr., 1857

D6824406-7170-5C51-BD1E-64434B362754

##### Distribution

Russian Far East to North & East China, Temperate East Asia

#### Tetradium daniellii

(Benn.) T.G.Hartley, 1981

C9C30ACA-2D6A-5B0B-881E-9A88A16B2C48

##### Distribution

SouthEast Tibet to China

#### Zanthoxylum piperitum

(L.) DC., 1824

1BD7F82F-9496-5FC7-B453-A03FFE14CAB6

##### Distribution

China to South Korea, Japan

#### Zanthoxylum schinifolium

Siebold & Zucc., 1845

0F9F6591-27CB-5A9C-9207-526079271463

##### Distribution

Central & East China to Temperate East Asia

#### Ailanthus altissima

(Mill.) Swingle, 1916

BC442EE0-05D1-5628-B4E2-16C304B117A1

##### Distribution

China

#### Picrasma quassioides

(D.Don) Benn., 1844

BB0C03ED-F58B-5503-8690-B15FBEC62B48

##### Distribution

Himalaya to Temperate East Asia

#### Rhus chinensis

Mill., 1768

2F891F10-F067-5650-AB99-C70C61761112

##### Distribution

North Pakistan to Japan, Sumatra

#### Toxicodendron sylvestre

(Siebold & Zucc.) Kuntze, 1891

C2D89EF1-4339-5223-BE6C-7BE645270C21

##### Distribution

South China, South Korea, South Central & South Japan to Taiwan

#### Toxicodendron trichocarpum

(Miq.) Kuntze, 1891

7C97DF6D-790D-5D5C-90B3-8FFB56DD51B0

##### Distribution

South China to Korea, Kuril Islands to Japan

#### Acer barbinerve

Maxim., 1867

4204C0EF-9737-584E-BF3D-69889214011D

##### Distribution

South Russian Far East to North Korea

#### Acer komarovii

Pojark., 1949

72271F2F-7115-5872-AD57-7A65A757096E

##### Distribution

South Russian Far East to Korea

#### Acer palmatum

Thunb., 1784

21C501E3-FE30-5C1F-B3E3-FB118A70C5E3

##### Distribution

SouthWest Korea, Central & S. Japan

#### Acer pictum var. mono

(Maxim.) Maxim., 1880

F391E1D1-80C6-522F-B432-3DB2BC6AFD9F

##### Distribution

China to Mongolia and Russian Far East, North & Central Japan

#### Acer pseudosieboldianum

(Pax) Kom., 1903

DE06A427-0F24-524D-9491-86D58AC34256

##### Distribution

South Russian Far East to Korea

#### Acer negundo

L., 1753

DEAE84E3-74F1-5C46-A346-ADB989EDFB1C

##### Distribution

Canada to Honduras

#### Acer tataricum ginnala

(Maxim.) Wesm., 1890

49FA0365-9FE7-5FA5-8B32-2200D04D9DD9

##### Distribution

Russian Far East to North & East Central China and Korea

#### Acer tegmentosum

Maxim., 1856

9B2F199C-A497-59FA-A6FE-CA79F666824F

##### Distribution

Russian Far East to Korea

#### Acer triflorum

Kom., 1901

9A91C040-AF88-5EF6-BBFD-799F8CBFAE86

##### Distribution

NorthEast China to Korea

#### Acer truncatum

Bunge, 1833

E24E1FB0-58F3-581F-8A99-3CEDEB8E5A71

##### Distribution

Russian Far East to North & East China, Korea, Japan

#### Acer ukurunduense

Trautv. & C.A.Mey., 1856

CCD14184-8FFF-59FC-8E1C-51E41B5C485E

##### Distribution

Russian Far East to Korea and Japan

#### Meliosma pinnata var. oldhamii

(Miq. ex Maxim.) Beusekom, 1971

43D77B06-3C71-568A-90BB-2E395769E650

##### Distribution

Central & South China to Korea, Japan

#### Impatiens noli-tangere

L., 1753

B8724C93-E9F3-50B6-BE0A-1C998FF159BA

##### Distribution

Temperate Eurasia, Alaska to Central Canada and West Central California

#### Impatiens textorii

Miq., 1865

82CCDD35-075A-562B-B2AD-E5FC78DDE4B2

##### Distribution

Russian Far East to North China and Japan

#### Impatiens textorii var. koreana

(Nakai) Nakai, 1917

8C00AD79-7700-588A-8753-BED9E68B91FD

##### Distribution

Russian Far East to North China and Japan

#### Ilex macropoda

Miq., 1867

72CD8DA4-20D5-5CC4-8958-82342B975DBD

##### Distribution

SouthEast China (to Hubei), Korea, Japan

#### Celastrus flagellaris

Rupr., 1857

377868A3-BDC6-58F9-A5D7-2F044692E826

##### Distribution

South Russian Far East to North China and Korea, Central & South Japan

#### Celastrus orbiculatus

Thunb., 1784

009146E1-46D2-56B2-946B-035966F6FEA2

##### Distribution

Russian Far East to China and Central & South Japan

#### Celastrus stephanotiifolius

(Makino) Makino, 1926

BE3E7767-F0F0-5BB5-9BC9-232B8D8B77E7

##### Distribution

Korea & Japan

#### Euonymus alatus

(Thunb.) Siebold, 1830

15AD90C3-6695-501D-950C-4541501808C2

##### Distribution

South Siberia to Japan and China

#### Euonymus alatus f. ciliato-dentatus

(Franch. & Sav.) Hiyama, 1956

371D296C-8108-501E-B51F-436CB3D87D41

##### Distribution

South Siberia to Japan and China

#### Euonymus fortunei

(Turcz.) Hand.-Mazz., 1933

A5389311-A5AA-5C91-93AF-F3F7C7DA8BC2

##### Distribution

Assam to Temperate East Asia and West & Central Malaysia

#### Euonymus hamiltonianus

Wall., 1824

E612901F-2AF7-5534-A99B-CAE7CADCD6C4

##### Distribution

East Afghanistan to Central & South Japan

#### Euonymus macropterus

Rupr., 1857

34D8E944-80AF-5C57-BD50-D61998367C2E

##### Distribution

Russian Far East to Korea and Japan

#### Euonymus oxyphyllus

Miq., 1865

B262058D-B510-5C30-851C-78F8CEE00C43

##### Distribution

Kuril Islands to China and Temperate East Asia

#### Euonymus pauciflorus

Maxim., 1859

6A4ABE37-FA52-584D-A65F-06943C4EED1B

##### Distribution

Central Europe to Korea, Central Japan

#### Euonymus sachalinensis

(F.Schmidt) Maxim., 1882

06D85EAF-4534-557A-8E65-3C216DF2BC56

##### Distribution

South Russian Far East to North East China and North & Central Japan

#### Tripterygium regelii

Sprague & Takeda, 1912

E5A9B700-1148-5C84-A64B-EFB838A19D73

##### Distribution

North East China to Korea, Japan

#### Euscaphis japonica

(Thunb.) Kanitz, 1878

3530B8EF-1D5D-5CF2-9962-7D8A80C9B48D

##### Distribution

Central & South China to North Vietnam and Temperate East Asia

#### Staphylea bumalda

DC., 1825

009C9345-D91F-5AD6-AB7B-09E239E00464

##### Distribution

China to Korea, Japan

#### Buxus sinica var. insularis

(Nakai) M.Cheng, 1980

4A2A3990-7A40-53BC-8556-A48841287ECF

##### Distribution

Korea & Japan

#### Berchemia berchemiifolia

(Makino) Koidz., 1925

CEF4382C-E76D-5CFC-9580-CE21FA07F07E

##### Distribution

Korea & Japan

#### Hovenia dulcis

Thunb., 1781

B4DC8521-477D-5F47-9EFB-1D705C0F5B34

##### Distribution

Indian Subcontinent to Korea, Japan

#### Rhamnus davurica

Pall., 1776

53100134-8E40-5192-8279-4D5313BAB6F4

##### Distribution

South Siberia to Russian Far East and Korea

#### Rhamnus koraiensis

C.K.Schneid., 1908

316CCBBC-48F2-50FA-A583-F5A3AF2E7C01

##### Distribution

Russian Far East to China, Korea and Japan

#### Rhamnus ussuriensis

J.J.Vassil., 1940

63D86674-6FC2-5ECA-8C6A-67F006F53D55

##### Distribution

Russian Far East to North Vietnam and Korea

#### Rhamnus yoshinoi

Makino, 1904

3CFDF111-BF45-5064-9413-1EF470160F38

##### Distribution

China to Korea, South Central & South Japan

#### Ziziphus jujuba

Mill., 1768

352D476E-7A54-5BF1-B65B-A321DC44461B

##### Distribution

North & East China to South Korea

#### Ziziphus jujuba var. inermis

(Bunge) Rehder, 1922

62854E61-DEBD-5C6E-A894-8A0F3FBA8DF3

##### Distribution

North & East China to South Korea

#### Ampelopsis glandulosa var. heterophylla

(Thunb.) Momiy., 1977

C61B42EF-A46D-5229-B46F-F7CBE424C494

##### Distribution

South Russian Far East to China, South Sakhalin to Japan

#### Parthenocissus tricuspidata

(Siebold & Zucc.) Planch., 1887

407142EE-DED7-55D9-BB80-22617241B474

##### Distribution

South Russian Far East to East China and Temperate East Asia

#### Vitis amurensis

Rupr., 1857

31688ABA-4319-5FFB-9F18-4A381731195B

##### Distribution

Russian Far East to East China and Korea, Japan

#### Vitis coignetiae

Pulliat ex Planch., 1883

F8A31F1E-011B-54E4-9001-393CCC528F20

##### Distribution

South Korea, Sakhalin to Japan

#### Vitis flexuosa

Thunb., 1794

91999F92-D0F3-57DE-B4D5-338B022F5747

##### Distribution

China to Korea, Japan and Tropical Asia

#### Vitis heyneana ficifolia

(Bunge) C.L.Li, 1996

F062D155-5A1A-5F11-972A-AFC79E5DC8DA

##### Distribution

North Central & East Central China to South Korea, Japan

#### Corchoropsis tomentosa

(Thunb.) Makino, 1903

5B4D99B9-BFB7-5871-8386-50087457BBBA

##### Distribution

Caribbean to North Venezuela

#### Corchoropsis tomentosa var. psilocarpa

(Harms & Loes. ex Gilg & Loes.) C.Y.Wu & Y.Tang, 1994

FFB3C5AE-3B9D-54F2-92FF-732750437989

##### Distribution

North & East China to Korea

#### Grewia biloba

G.Don, 1831

BBF13966-24F2-5F4C-8DCB-A83980D3F68B

##### Distribution

Central & South China to Korea, Taiwan

#### Tilia amurensis

Rupr., 1869

7A34D913-ED10-5CE9-BD1C-CA2ED12A3DF5

##### Distribution

Russian Far East to Korea

#### Tilia mandshurica

Rupr. & Maxim., 1856

EB5E32EB-1FA8-5B1C-967F-8C980DD78BF8

##### Distribution

Russian Far East to North & East Central China and Central Japan

#### Tilia taquetii

C.K.Schneid., 1909

AD9EE320-1C8E-5853-BC7E-2285A8A47C60

##### Distribution

Russian Far East to Korea

#### Triumfetta japonica

Makino, 1913

5F1A2FA8-A679-5071-8351-A20FEA1C1963

##### Distribution

Korea, South Central & South Japan to Philippines

#### Citrus trifoliata

L., 1763

AC352AFA-B6FE-5082-BB23-CE4249340A4B

##### Distribution

China

#### Viola acuminata

Ledeb., 1842

1AD5FEB4-4D53-5C08-AB6D-8980B4970DDE

##### Distribution

Siberia to China and North & Central Japan

#### Viola albida

Palib., 1900

8387E81E-277F-551E-A384-D30FEDBA6880

##### Distribution

South Russian Far East to Korea

#### Viola albida var. chaerophylloides

(Regel) F.Maek. ex H.Hara, 1954

5A6BE3BE-E60F-5DD7-861F-B5EDF75747B1

##### Distribution

South Russian Far East to China and Korea, South Japan

#### Viola collina

Besser, 1816

62C835C6-BFB6-5CEE-8887-A65DA40D8B2E

##### Distribution

Temperate Eurasia

#### Viola hirtipes

S.Moore, 1879

7FB668E6-6894-500E-BC79-723886FB7B55

##### Distribution

South Russian Far East to Korea, Japan

#### Viola japonica

Langsd. ex Ging., 1824

FE743433-0D66-54D9-8681-7290210A240F

##### Distribution

Korea, Japan & Taiwan

#### Viola keiskei

Miq., 1866

431F4692-4DCF-5B9D-BE88-EA40683E6AD3

##### Distribution

Korea & Japan

#### Viola lactiflora

Nakai, 1914

B8500FCB-B49A-5766-8793-B8AD41AF9B7D

##### Distribution

East China to Korea

#### Viola mandshurica

W.Becker, 1917

50AFECCD-D8D7-5BCA-A7ED-FFAC9C46D43E

##### Distribution

Russian Far East to North & East China and Japan

#### Viola orientalis

(Maxim.) W.Becker, 1915

54FBE366-2273-51A4-B589-B8589C674D86

##### Distribution

South Russian Far East to East China and Korea, Central & South Japan

#### Viola ovato-oblonga

(Miq.) Makino, 1907

76F3F0CC-F64C-5411-959E-701DAA92A08D

##### Distribution

Korea & Japan

#### Viola phalacrocarpa

Maxim., 187

DC6B52C3-F075-5860-B524-1808558DB074

##### Distribution

South Russian Far East to Korea, Japan

#### Viola philippica

Cav., 1800

BCC5B76C-AF0F-53AC-9A79-A10DFA3C7975

##### Distribution

Asia

#### Viola rossii

Hemsl., 1886

E955B35A-6DB3-5C0B-9CD3-1675BDC8AA78

##### Distribution

South Russian Far East to East Central & East China and Korea, Japan

#### Viola selkirkii

Pursh ex Goldie, 1822

8CE9E7BE-A110-5DC6-A23F-479FA1F8ADBF

##### Distribution

North Europe to Japan, Subarctic America to North & West Central U.S.A.

#### Viola sororia

Willd., 1806

4D082647-AC5D-508A-B9EC-37B1E37B6721

##### Distribution

Canada to U.S.A. and East Mexico

#### Viola tenuicornis

W.Becker, 1920

488F180F-4609-5BA4-AEFE-37C9A62235D8

##### Distribution

NorthEast China to Korea

#### Viola tokubuchiana var. takedana

(Makino) F.Maek., 1954

731612B9-F5A7-5681-ADFC-3BD4AE4D2B8B

##### Distribution

NorthEast China to South Korea, Japan

#### Viola variegata

Fisch. ex Link, 1821

424F6B3A-D0AF-5802-8C8F-69892F0CA937

##### Distribution

SouthEast. Siberia to North China and Japan

#### Viola verecunda

A.Gray, 1858

22F5E1B2-8031-52B0-958D-FEAB73B13ADF

##### Distribution

Asia

#### Viola violacea

Makino, 1891

56070145-E301-575C-A613-6733029F57EA

##### Distribution

SouthEast China, South Korea, Central & South Japan

#### Sicyos angulatus

L., 1753

1DCFD18F-06F6-512E-A18C-55B710D5B5B7

##### Distribution

East Canada to Central & East U.S.A.

#### Elaeagnus macrophylla

Thunb., 1784

B5F8072D-EE59-5973-B786-8BA45163312E

##### Distribution

East China, South Korea (including Jeju-do), South Central & South Japan to Taiwan

#### Elaeagnus umbellata

Thunb., 1784

6F9AF35C-C059-54BB-9FA5-8E63CD7B4A53

##### Distribution

Afghanistan to Temperate East Asia

#### Lythrum salicaria

L., 1753

1C385426-EB0B-5333-BA59-A9BD6EBDA199

##### Distribution

Temperate Eurasia, North West Africa, Ethiopia, Australia

#### Circaea alpina

L., 1753

95F5C83D-D7F2-5316-839A-35A005FA3C1F

##### Distribution

Temperate Northern Hemisphere

#### Circaea cordata

Royle, 1835

A850C60C-7C9A-51AB-9271-DBFFEF24476B

##### Distribution

NorthEast Pakistan to Russian Far East and Temperate East Asia

#### Circaea lutetiana quadrisulcata

(Maxim.) Asch. & Magnus, 1870

064A5808-4ED0-5741-AAE3-1F9D7EABCAFE

##### Distribution

European Russia to North & North Central Japan

#### Circaea mollis

Siebold & Zucc., 1845

A198984D-68B3-527F-8D57-C7FC7B9411DC

##### Distribution

South Russian Far East to Indo-China

#### Ludwigia prostrata

Roxb., 1820

F621C09E-E99F-5E53-95A8-E371107805D0

##### Distribution

South Russian Far East to Tropical & Subtropical Asia

#### Oenothera biennis

L., 1753

C7DC8622-5FB6-5ACD-9F66-37C08F6FFB78

##### Distribution

Canada to Central Mexico

#### Alangium platanifolium var. trilobum

(Miq.) Ohwi, 1965

BEE3D5B0-DBCB-5257-91C6-A01921DAE2B0

##### Distribution

South Russian Far East to China, Temperate East Asia

#### Aucuba japonica

Thunb., 1783

05BFC80C-81AC-5E02-85F1-D427EBCF3329

##### Distribution

SouthEast. China to Temperate East Asia

#### Cornus controversa

Hemsl., 1909

091247A1-65BE-537B-B567-0B2A12C36DDA

##### Distribution

Central Himalaya to South Kuril Islands and North Indo-China

#### Cornus kousa

Bürger ex Hance, 1873

DB48D8C2-80CC-5775-A081-465AA0DAAF17

##### Distribution

China to Temperate East Asia

#### Cornus macrophylla

Wall., 1820

A0EED03D-C111-5AF3-BFD6-4D496C186CCD

##### Distribution

Afghanistan to Sakhalin, Korea

#### Cornus walteri

Wangerin, 1908

564AF042-73FB-5289-B6E1-4D5DBE19EB24

##### Distribution

China to Korea

#### Aralia cordata

Thunb., 1784

9A7A73C8-5F01-578A-8660-87EA4D7E4A28

##### Distribution

SouthEast China (to Hubei), Sakhalin to Temperate East Asia

#### Aralia cordata var. continentalis

(Kitag.) Y.C.Chu, 1989

AA435C79-251E-5A8D-9FBC-6F16F8809B1B

##### Distribution

South Russian Far East to China and Korea

#### Aralia elata

(Miq.) Seem., 1868

23DC5F60-6B0F-5BE2-A2E6-6D8CED9A718F

##### Distribution

Russian Far East to China and Temperate East Asia

#### Eleutherococcus divaricatus

(Siebold & Zucc.) S.Y.Hu, 1980

04B1EF8E-CC71-577E-A9B8-6CA4472DA7B7

##### Distribution

Korea & Japan

#### Eleutherococcus divaricatus var. chiisanensis

(Nakai) C.H.Kim & B.-Y.Sun, 2000

84627AB8-CAAD-57B7-A122-F6F58DFF19BE

##### Distribution

Korea & Japan

#### Eleutherococcus sessiliflorus

(Rupr. & Maxim.) S.Y.Hu, 1980

D99A1853-C3B1-586B-B7AA-CE4ED7FAF922

##### Distribution

Russian Far East to North China and Korea

#### Hedera rhombea

(Miq.) Paul, 1867

97CA1CB8-D703-59EB-BAB9-A8F539E2D12A

##### Distribution

Temperate East Asia

#### Kalopanax septemlobus

(Thunb.) Koidz., 1925

1A899514-719A-5373-9452-8DB1C82345F4

##### Distribution

Russian Far East to China and Temperate East Asia

#### Oplopanax elatus

(Nakai) Nakai, 1927

A36A7B26-4332-5723-A014-8A972B86F660

##### Distribution

South Primorye, China (E. Jilin), Korea

#### Angelica amurensis

Schischk., 1951

40598E63-0419-5A32-BF61-993FAEE40D0D

##### Distribution

Russian Far East to Central China and Korea, Japan

#### Angelica cartilaginomarginata

(Makino ex Y.Yabe) Nakai, 1909

384FCDAC-9680-5765-8185-404F3307518B

##### Distribution

Northeast China to Central & South Japan

#### Angelica dahurica

(Fisch. ex Hoffm.) Benth. & Hook.f. ex Franch. & Sav., 1873

283F3255-9498-58DE-BFDE-356E3A1F18DD

##### Distribution

South Siberia to North China and Japan

#### Angelica decursiva

(Miq.) Franch. & Sav., 1873

F8AD3E0B-7993-58E4-BA0A-0E2A9F11BB49

##### Distribution

Vietnam to South Russian Far East and Temperate East Asia

#### Angelica gigas

Nakai, 1917

076C45A9-BEA7-5AD2-B673-F1081DC9BA16

##### Distribution

Northeast China to Korea

#### Angelica polymorpha

Maxim., 1873

F822FB00-F614-5DC9-B622-E7E891949328

##### Distribution

North & East China to Central & South Japan

#### Angelica reflexa

B.Y.Lee, 2013

3981C1DC-3698-57A1-8C49-311654A5C8C1

##### Distribution

Russian Far East to Korea and North & Central Japan, Aleutian Islands to West U.S.A.

#### Anthriscus caucalis

M.Bieb., 1808

B367B5A0-5F9E-5697-9423-4616290C9251

##### Distribution

Macaronesia to Northwest Africa, Europe to Caucasus

#### Anthriscus sylvestris

(L.) Hoffm., 1814

5133D884-7534-5262-8675-80D423889F7E

##### Distribution

Temperate Eurasia to Tropical African Mountains

#### Bupleurum longeradiatum

Turcz., 1844

C654ADD5-267D-596D-A514-811D7D229BC7

##### Distribution

Southeast Siberia to Japan and South-Central China

#### Cnidium monnieri

(L.) Cusson, 1782

0A102586-8ADB-5A9D-99D8-F81095896932

##### Distribution

Temperate Asia to Indo-China

#### Ligusticum officinale

(Makino) Kitag., 1963

43F5B635-5E4A-5667-8D59-886AA476CFE9

##### Distribution

Japan, Korea

#### Oenanthe javanica

DC., 1830

D3598209-F634-589D-B574-A3AB17DE3042

##### Distribution

SouthEast Siberia to Tropical & Subtropical Asia

#### Osmorhiza aristata

(Thunb.) Rydb., 1894

321AB573-DB5B-5B37-9BF9-2A5BCD8AD337

##### Distribution

Temperate Asia

#### Ostericum grosseserratum

(Maxim.) Kitag., 1936

0BCBC190-1359-5302-9285-EA088723A4EC

##### Distribution

Mongolia to Korea and China

#### Ostericum sieboldii

(Miq.) Nakai, 1942

E1E66A44-09EF-5EE4-8446-1E4D5A2E104F

##### Distribution

Russian Far East to North China and Korea, Central & South Japan

#### Peucedanum terebinthaceum

(Fisch. ex Trevir.) Turcz., 1838

67B69C58-253F-5B01-93D9-E4137C8C5739

##### Distribution

South Siberia to Japan and North China

#### Pimpinella brachycarpa

(Kom.) Nakai, 1909

7345C955-662F-559C-94EE-B04AB0CA5D7C

##### Distribution

South Russian Far East to Central & East China and Korea, South Central & South Japan

#### Sanicula chinensis

Bunge, 1835

C91DEB14-48A8-5522-8CE7-3E85D5725851

##### Distribution

Russian Far East to China, Korea and Japan

#### Sillaphyton podagraria

(H.Boissieu) Pimenov, 2016

9864B6AF-850C-5C83-A6F9-F2809B1918D0

##### Distribution

Korea

#### Torilis japonica

(Houtt.) DC., 1830

8D8FB634-A047-5F55-9BBF-3F91C20E5A8C

##### Distribution

Temperate Eurasia to North Indo-China

#### Torilis scabra

(Thunb.) DC., 1830

0A715DD3-6354-5A6B-940F-D9BE9696F64E

##### Distribution

China to Temperate East Asia

#### Chimaphila japonica

Miq., 1866

B736192B-5790-5D55-9D90-CE5D7D22B156

##### Distribution

Russian Far East to Bhutan, Japan to Taiwan

#### Pyrola japonica

Klenze ex Alef., 1856

A2DAD837-BC5E-52B8-BBA4-77E28B0FC340

##### Distribution

South Russian Far East to North & East Central China and Korea, Japan, Taiwan

#### Rhododendron brachycarpum

D.Don ex G.Don, 1834

CA911305-38AB-542D-B9D4-EC98757F09B4

##### Distribution

Korea & Japan

#### Rhododendron mucronulatum

Turcz., 1837

ED445FCF-10EA-5119-896B-E9236BFF6E90

##### Distribution

Russian Far East to North & East China and Korea, West Central & South Japan

#### Rhododendron mucronulatum var. ciliatum

Nakai, 1917

9C4BE1C4-35C7-53E8-B029-6856EEB589AD

##### Distribution

Russian Far East to North & East China and Korea, West Central & South Japan

#### Rhododendron schlippenbachii

Maxim., 1870

02BD8AB7-605A-5A88-B71C-93160F79E41C

##### Distribution

South Russian Far East to North China and Japan

#### Rhododendron yedoense f. poukhanense

(H.Lév.) Sugim. ex T.Yamaz., 1996

4E690001-BF5E-587B-ACA8-40B93F956DD8

##### Distribution

Korea & Japan

#### Vaccinium bracteatum

Thunb., 1784

D7083B5A-685A-52B3-BB3A-9B715CF54BC8

##### Distribution

South China to South Central & South Japan and West Malaysia

#### Vaccinium hirtum var. koreanum

(Nakai) Kitam., 1972

0745C54F-BA3C-5132-B476-E7E2A70D12CB

##### Distribution

China to Korea

#### Vaccinium oldhamii

Miq., 1866

20C79990-4937-5CCD-B664-DE641304F143

##### Distribution

East China, South Korea, Japan

#### Vaccinium vitis-idaea

L., 1753

ECD04CBF-8744-51D9-9879-C18E82C8A44D

##### Distribution

Subarctic & Temperate Northern Hemisphere

#### Ardisia japonica

(Thunb.) Blume, 1826

53C1FF8D-D097-5B80-834F-95E9B733D90E

##### Distribution

Central & South China to Temperate East Asia

#### Androsace umbellata

(Lour.) Merr., 1919

43B46441-D4D5-581B-A878-099B88876368

##### Distribution

North Pakistan to Russian Far East and Philippines (N. Luzon), NorthEast New Guinea

#### Lysimachia clethroides

Duby, 1844

ADB811B8-BC78-51B0-8EE0-E05C5379CC86

##### Distribution

South Russian Far East to Indo-China and Temperate East Asia

#### Primula jesoana

Miq., 1867

37CA1F39-8377-52BD-9E2D-95771B196CE1

##### Distribution

Primorye to East China and Korea, North & Central Japan

#### Diospyros lotus

L., 1753

CFCB7DA8-B058-5DB7-8EA0-9B800896FC9C

##### Distribution

NorthEast & South Central Türkiye to Korea

#### Styrax japonicus

Siebold & Zucc., 1837

198A0F8B-6CC4-5391-999D-164C094FA49C

##### Distribution

Nepal to Japan and North Philippines

#### Styrax obassia

Siebold & Zucc., 1839

EE6B380A-1BDE-595D-9517-822A5EBC0362

##### Distribution

East & SouthEast China to Korea, Japan

#### Symplocos sawafutagi

Nagam., 1993

52E7BE92-F6D2-527F-B415-7C5197559ABF

##### Distribution

Korea & Japan

#### Symplocos tanakana

Nakai, 1918

BA9684F2-AFDA-567B-9E1F-DF7EF8934376

##### Distribution

East China, South Korea, Japan

#### Forsythia saxatilis

(Nakai) Nakai, 1921

1B11A588-11B0-5B01-97C2-1006124586AD

##### Distribution

Korea

#### Fraxinus chiisanensis

Nakai, 1929

3B43EAD9-C783-55E4-9ECB-1D9E2F919DFE

##### Distribution

Korea

#### Fraxinus mandshurica

Rupr., 1857

89F4BF01-4019-5619-B771-53FC642C76FE

##### Distribution

Russian Far East to Central China, North & Central Japan

#### Fraxinus rhynchophylla

Hance, 1869

2F13F354-0235-54B3-8DD0-3DA01BE45AC8

##### Distribution

South Russian Far East to East China and Japan

#### Fraxinus sieboldiana

Blume, 1851

C8CF6035-3C0B-5E10-9F0E-D4ADA6327137

##### Distribution

SouthEast China to Korea, Central & South Japan

#### Ligustrum obtusifolium

Siebold & Zucc., 1846

C9B2FC5F-B903-597A-B0D0-64FAECF9D5BC

##### Distribution

East China to Korea, Japan

#### Ligustrum ovalifolium

Hassk., 1844

897BD209-CBEA-512D-AF2E-5017A430401D

##### Distribution

Korea & Japan

#### Syringa fauriei

H.Lév., 1910

1A013B4D-D08E-5804-AF3F-676E315E23EC

##### Distribution

Russian Far East to Korea

#### Syringa pubescens Patula

(Palib.) M.C.Chang & X.L.Chen, 1990

F7B6EB36-2BF0-5A46-BFE4-E9130013798A

##### Distribution

North East China to Korea

#### Syringa reticulata

(Blume) H.Hara, 1941

BB3C1C42-9C12-538D-B83F-207159AADB74

##### Distribution

Russian Far East to China and Japan

#### Syringa villosa wolfii

(C.K.Schneid.) Y.Chen & D.Y.Hong, 2007

B57D4419-375E-5C4A-A3F7-F2DB6E461524

##### Distribution

South Russian Far East to Korea

#### Mitrasacme pygmaea

R.Br., 1810

3A74AB5B-8283-5B09-ACBF-05A2974B57C8

##### Distribution

Tropical & Subtropical Asia to West Pacific

#### Gentiana scabra

Bunge, 1836

6A8F2BC1-D875-559F-8357-70CB3C0C54D9

##### Distribution

SouthEast Siberia to Japan, Korea and East China

#### Gentiana squarrosa

Ledeb., 1812

CACFC793-0B16-510A-92E5-14B9F1C66F19

##### Distribution

Siberia to North Pakistan and Japan

#### Gentiana triflora var. japonica

(Kusn.) H.Hara, 1949

B3A9C853-EC5A-59C1-850A-168E7A5D2138

##### Distribution

Sakhalin to North & Central Japan, Korea

#### Gentiana zollingeri

Fawc., 1883

8B061BAB-B7DD-57B5-B566-7ED267C2F04C

##### Distribution

Russian Far East to Central China and Japan

#### Cynanchum ascyrifolium

(Franch. & Sav.) Matsum., 1912

44D9C6DE-B35C-59B3-A927-46D877F08BB3

##### Distribution

North & East China to Russian Far East and Japan

#### Cynanchum paniculatum

(Bunge) Kitag. ex H.Hara, 1948

65438F6B-65F6-5425-8231-2178121D1B7F

##### Distribution

South Siberia to Temperate East Asia and China

#### Cynanchum wilfordii

(Maxim.) Maxim. ex Hook.f., 1883

0639E9EB-A829-5EFF-9921-F4C11757654B

##### Distribution

Russian Far East to China, Central & South Japan

#### Metaplexis japonica

(Thunb.) Makino, 1903

64555DFC-4DDE-5AC4-ABAF-C00FF84D16D7

##### Distribution

China to Russian Far East Korea and Japan

#### Trachelospermum asiaticum

(Siebold & Zucc.) Nakai, 1922

E45A1FC0-8B4A-5D09-A274-CC8A3D687E48

##### Distribution

North India to Central & South Japan and Borneo

#### Tylophora floribunda

Miq., 1866

DBDDC2B0-9E2C-59BC-A243-412A60D97BBB

##### Distribution

East Central & SouthEast China to Central & South Japan

#### Asperula lasiantha

Nakai, 1938

03C829D7-A6DD-5566-A271-329C5584A285

##### Distribution

Korea

#### Galium bungei var. trachyspermum

(A.Gray) Cufod., 1940

DE6759B6-564B-52E3-9389-63C039382841

##### Distribution

Central & South China, Korea, Japan

#### Galium dahuricum

Turcz. ex Ledeb., 1844

9A06D24F-D397-5210-A467-880CBB41FACB

##### Distribution

South Siberia to Russian Far East and China

#### Galium gracilens

(A.Gray) Makino, 1903

92E0B700-7E3F-5033-8ADD-79A04F482760

##### Distribution

Central & South China, Korea, Japan

#### Galium maximowiczii

(Kom.) Pobed., 1970

82BAEC22-62B1-585A-BE8D-BE0E1EEEDE2A

##### Distribution

China to Russian Far East Korea and Japan

#### Galium odoratum

(L.) Scop., 1771

938A5DBD-516F-59F1-A2E0-ACB978E0E8E5

##### Distribution

Temperate Eurasia, Algeria

#### Galium pogonanthum

Franch. & Sav., 1878

B29B2119-754D-5FAF-8432-1C08BB0F0F4C

##### Distribution

China to Temperate East Asia

#### Galium spurium var. echinospermum

(Wallr.) Klett & Richt., 1830

5D446EB8-0FA8-5466-9E4D-27C5CAF29CD9

##### Distribution

Temperate Northern Hemisphere

#### Galium tricornutum

Dandy, 1957

8B06CA91-6D91-5064-A13E-1549B2CA092B

##### Distribution

Europe to West Himalaya and Arabian Peninsula

#### Galium trifidum

L., 1753

14898C0D-2B09-5F87-8090-36BBD82284D6

##### Distribution

Temperate Northern Hemisphere to Mexico, Hispaniola

#### Galium trifloriforme

Kom., 1901

B15B959D-6B01-52C6-8C45-90CE245E0649

##### Distribution

China (Qinghai) to Korea, Sakhalin to Japan

#### Galium verum asiaticum

(Nakai) T.Yamaz., 1993

C06C8581-724B-57C4-BA7C-C818F5208350

##### Distribution

Russian Far East to China and Japan

#### Paederia foetida

L., 1767

4EA62B6B-2236-5593-82C6-70D76133DB0E

##### Distribution

East Nepal to Korea, Japan and Malesia

#### Rubia argyi

(H.Lév. & Vaniot) H.Hara, 1972

49C06799-6FB2-55B7-87E8-87C8E3D03B52

##### Distribution

Central & South China to Temperate East Asia

#### Rubia chinensis

Regel & Maack, 1861

5EC08FE9-46D2-5F53-904D-0C348B9ED2D4

##### Distribution

Russian Far East to Korea, Japan

#### Rubia cordifolia

L., 1767

D2C09DAA-0432-5579-A362-BAC189B55F4F

##### Distribution

Greece, Sudan to South Africa, Asia

#### Calystegia hederacea

Wall., 1824

C8FFF68D-C5F6-5520-B74C-F16953222E1E

##### Distribution

Ethiopia, Afghanistan to Korea, Japan

#### Calystegia pubescens

Lindl., 1846

C450A7B8-D292-5FD6-931B-3745CA56301D

##### Distribution

Central & East China to Korea, Kuril Islands to Japan

#### Cuscuta australis

R.Br., 1810

E2614F50-5A33-535B-BD9D-B247C890AFEF

##### Distribution

Old World

#### Ipomoea nil

(L.) Roth, 1797

1E720AB4-4ECF-5289-9848-BB4ECDB6E226

##### Distribution

Tropical & Subtropical America

#### Quamoclit coccinea

(L.) Moench, 1794

12552354-EDC5-504E-9ED2-96523BC7367B

##### Distribution

Central & East U.S.A

#### Bothriospermum zeylanicum

(J.Jacq.) Druce, 1917

901DD3B0-0D1A-52F5-ABEE-87FE15D6739E

##### Distribution

Central Asia to Japan and Philippines

#### Brachybotrys paridiformis

Maxim. ex Oliv., 1878

84A5FE40-DC29-55DA-8666-4FC19B54042A

##### Distribution

Primorye to Korea

#### Lithospermum erythrorhizon

Siebold & Zucc., 1846

6D4CE0AB-47D0-57B5-BB6B-322558D7A3A0

##### Distribution

East Siberia to Korea, Japan

#### Trigonotis peduncularis

(Trevis.) Benth. ex Hemsl., 1890

B4EC8434-390D-55A3-A04B-7C48A78333B9

##### Distribution

South European Russia to Temperate Asia

#### Callitriche palustris

L., 1753

E78FDE0D-DEF9-54A1-A4BC-5FE48B22080C

##### Distribution

Temperate Northern Hemisphere to West Malesia

#### Callicarpa dichotoma

(Lour.) K.Koch, 1872

279131E2-02B8-5C7A-93BA-34FD85CF9D40

##### Distribution

Central & South Japan, Korea to Vietnam

#### Callicarpa japonica

Thunb., 1784

D6130C1B-E1EA-514D-A853-8A1B6E048DC7

##### Distribution

China to Temperate East Asia

#### Clerodendrum trichotomum

Thunb., 1784

EB134273-6F61-5B1E-A9B6-E40046244788

##### Distribution

China, Temperate East Asia to North Philippines

#### Tripora divaricata

(Maxim.) P.D.Cantino, 1999

9847239A-A23D-5B0F-8611-916E05200746

##### Distribution

China to Korea, Japan

#### Agastache rugosa

(Fisch. & C.A.Mey.) Kuntze, 1891

43286293-C746-5266-ADE4-7C1928F0FE9C

##### Distribution

Russian Far East to Temperate East Asia

#### Clinopodium chinense var. parviflorum

(Kudô) H.Hara, 1936

EB072BC2-F077-56C6-AFC9-0FAD455C17FA

##### Distribution

Korea, South Kuril Islands to Japan

#### Clinopodium micranthum

(Regel) H.Hara, 1940

5C1616B5-EE35-5D25-ADA7-282E424A08C2

##### Distribution

Korea, Sakhalin to Japan

#### Clinopodium multicaule

(Maxim.) Kuntze, 1891

1CBDB32D-A508-506D-B9A2-B2CA26ADDA78

##### Distribution

Korea & Japan

#### Clinopodium multicaule var. shibetchense

(H.Lév.) Melnikov, 2016

55B566FE-5713-5A39-8312-C64591B3B276

##### Distribution

Korea & Japan

#### Dracocephalum argunense

Fisch. ex Rchb., 1821

E0BE897F-1E5B-566C-BC1A-FC48E84317AE

##### Distribution

SouthEast Siberia to Korea, North & Central Japan

#### Elsholtzia ciliata

(Thunb.) Hyl., 1941

E9F67917-9A0A-5F78-B0C3-C76CAE62792E

##### Distribution

Temperate Asia to Peninsula Malaysia

#### Elsholtzia splendens

Nakai ex F.Maek., 1934

2CA4724F-6F2A-5094-9ED5-8CB772B30CAC

##### Distribution

China to Korea

#### Isodon excisus

(Maxim.) Kudô, 1929

77C4E96F-86A1-50CC-A78D-C0215DB54825

##### Distribution

Russian Far East to Korea

#### Isodon inflexus

(Thunb.) Kudô, 1929

55A4957F-5C35-50EA-9E9B-AD8D015C1941

##### Distribution

China to Korea, Japan

#### Isodon japonicus

(Burm.f.) H.Hara, 1948

402DEDAB-24A8-5E49-99A9-81E12791B523

##### Distribution

Russian Far East to Central China and Japan

#### Isodon serra

(Maxim.) Kudô, 1929

EDD22911-A7E5-56F3-8218-8FB2C85E4A9B

##### Distribution

South Russian Far East to Taiwan

#### Lamium album Barbatum

(Siebold & Zucc.) Mennema, 1989

66FB9422-A530-512F-90FF-A4C3950B3E86

##### Distribution

Siberia to Japan and China

#### Lamium amplexicaule

L., 1753

F69A9AFA-EAB8-5933-8B67-6F5492881904

##### Distribution

Temperate Eurasia, Macaronesia to Ethiopia

#### Lamium purpureum

L., 1753

C08A7B24-2857-5697-9B58-3CA3614D8A27

##### Distribution

Macaronesia, Mediterranean, Europe to West Siberia

#### Leonurus japonicus

Houtt., 1778

3C55E16F-A181-52F7-89A0-76DC0D376BD0

##### Distribution

China to Russian Far East and North Australia

#### Leonurus macranthus

Maxim., 1859

A5F56189-5BC9-5EAE-B3AF-87BFFC690FA9

##### Distribution

Russian Far East to North China and Japan

#### Lycopus lucidus

Turcz. ex Benth., 1848

C59345CE-6C67-5167-AC64-3F42DFBE9672

##### Distribution

South & East Siberia to Temperate East Asia

#### Meehania urticifolia

(Miq.) Makino, 1899

55D7338F-2C87-5159-A7C5-564E64FF5237

##### Distribution

South Primorye to Korea, Central & South Japan

#### Mosla scabra

(Thunb.) C.Y.Wu & H.W.Li, 1974

3D65085E-B1D9-5686-B53A-18FCBE03A2F8

##### Distribution

China to Vietnam and Temperate East Asia

#### Phlomis umbrosa

Turcz., 1840

47751708-9018-5B57-9FFE-F10DFBC16C08

##### Distribution

Central & East China to Korea

#### Prunella vulgaris Asiatica

(Nakai) H.Hara, 1948

605BD447-0D3D-5C3A-A113-9048219D5315

##### Distribution

East China to far East Asia and Alaska (Aleutian Islands)

#### Scutellaria baicalensis

Georgi, 1775

4609D00D-CB3A-545F-A0C2-EAD1D57E2C87

##### Distribution

South Siberia to North Korea and Vietnam The Royal Botanic Garden, Kew (2023)

#### Scutellaria indica

L., 1753

41CF6F22-6F36-5864-ACFC-93ACF9DB9C1D

##### Distribution

Tropical & Subtropical Asia

#### Scutellaria pekinensis var. transitra

(Makino) H.Hara, 1948

B8953239-4B72-5DD4-A836-8BAD694A55FC

##### Distribution

SouthEast China to Korea, Japan

#### Scutellaria pekinensis var. ussuriensis

(Regel) Hand.-Mazz., 1939

98D72293-F6FD-5DBC-9BBD-4E80C47F1078

##### Distribution

Mongolia to Korea, North & Central Japan

#### Teucrium viscidum var. miquelianum

(Maxim.) H.Hara, 1937

9AAA1408-AEF2-5925-B3AC-9EEF36EFA82D

##### Distribution

South Korea, South Kuril Islands to Japan

#### Physalis alkekengi

L., 1753

AA35546E-229C-55E6-9F17-B32F3C5840AF

##### Distribution

Temperate Eurasia

#### Solanum japonense

Nakai, 1923

0764170B-659E-5708-AB64-E0C5BCE645D7

##### Distribution

Himalaya to China and North Indo-China, Temperate East Asia, Central Sumatra

#### Solanum lyratum

Thunb., 1784

9522794F-47FB-5ECD-9A8B-3810DC9BEF38

##### Distribution

China to Temperate East Asia and Indo-China

#### Solanum nigrum

L., 1753

2FB55DC1-00B2-579E-9776-ED8BAF2BE984

##### Distribution

Temperate Eurasia, Macaronesia, North & Northeast Tropical Africa

#### Mazus pumilus

(Burm.f.) Steenis, 1958

4478DD27-01A4-53CB-9AD5-D530DF57FFDC

##### Distribution

Asia

#### Mazus stachydifolius

(Turcz.) Maxim., 1875

88E10610-DF92-51F1-B986-FC0B1E20C0B6

##### Distribution

SouthEast Siberia to China and Temperate East Asia

#### Melampyrum roseum

Maxim., 1859

60CEA77C-306E-572D-AC22-A7DABDC64A70

##### Distribution

Russian Far East to China, Japan to North Taiwan

#### Melampyrum roseum var. japonicum

Franch. & Sav., 1873

08670373-A27C-5CC3-8000-01B90CD8723B

##### Distribution

Russian Far East to China, Japan to North Taiwan

#### Melampyrum roseum var. ovalifolium

(Nakai) Nakai ex Beauverd, 1916

FC55CB20-0512-555B-AEB1-366FC8CE4CFD

##### Distribution

China (Zhejiang), Korea, West Japan

#### Melampyrum setaceum var. nakaianum

(Tuyama) T.Yamaz., 1954

6F261D34-FA9E-5F00-84E6-CE797BAC061E

##### Distribution

Russian Far East to Korea, Japan

#### Paulownia coreana

Uyeki, 1925

CE177663-8946-5ABF-94BE-C2761584755B

##### Distribution

Central & East China, South Korea

#### Pedicularis resupinata

L., 1753

29A3FB2C-BA46-59FB-8B01-5999988D9251

##### Distribution

E. European Russia to Korea, Japan

#### Phtheirospermum japonicum

(Thunb.) Kanitz, 1878

3B05DDBE-1008-5A04-82CB-174F3BBE1E05

##### Distribution

Russian Far East to China and Japan

#### Pseudolysimachion dauricum

(Steven) Holub, 1967

8D5BED94-6CF0-58D6-948B-8E82F45FDB0E

##### Distribution

South Siberia to Russian Far East and North & East Central China

#### Pseudolysimachion pyrethrinum

(Nakai) T.Yamaz., 1968

FACC6549-1914-5D52-99B9-4A3FD2212B81

##### Distribution

Korea

#### Veronica anagallis-aquatica

L., 1753

238BD856-7956-5C93-91B9-840EA52EB4B5

##### Distribution

Temperate Eurasia to Tropical Mountains

#### Veronica arvensis

L., 1753

D39884D6-2E56-5AC9-887A-11E54E792B32

##### Distribution

Macaronesia, NorthWest Africa, Europe to SouthWest Siberia and West Himalaya

#### Veronica persica

Poir., 1808

02767645-EC1B-5DED-B3D5-2620D74ECC0F

##### Distribution

Caucasus to North Iran

#### Justicia procumbens

L., 1753

4A5EF666-8004-50D0-8F9D-E8561209B4FC

##### Distribution

Tropical & Subtropical Asia to Central China

#### Strobilanthes oliganthus

Miq., 1865

DD26921E-7ADE-5F39-8B1B-368EC3A52CCE

##### Distribution

South Korea (Jeju island) & Japan

#### Phryma leptostachya var. oblongifolia

(Koidz.) Honda, 1936

EA981499-4F0C-543A-819A-1EA5F58B5960

##### Distribution

Korea & Japan

#### Plantago asiatica

L., 1753

199F867B-C8BC-50F7-8FC9-CB4F81E5FBD9

##### Distribution

Asia

#### Lonicera chrysantha

Turcz. ex Ledeb., 1844

5F851B9C-0EEF-5906-8876-2124FDAEC5B9

##### Distribution

SouthEast Siberia to China and North Japan

#### Lonicera harae

Makino, 1914

F18E2D30-9771-5506-80C6-496657E155AC

##### Distribution

Korea & Japan

#### Lonicera japonica

Thunb., 1784

E445C01A-A789-5629-A661-21D1B508FC53

##### Distribution

China to Temperate East Asia

#### Lonicera maackii

(Rupr.) Maxim., 1859

D4B12E9F-49C4-5777-B0E1-F8FCACDF2400

##### Distribution

Russian Far East to China and Central Japan

#### Lonicera praeflorens

Batalin, 1892

A2881694-E538-5ED5-B251-6BFCDEA30FA2

##### Distribution

South Russian Far East to Korea, Central Japan

#### Lonicera subhispida

Nakai, 1921

62D93AEE-1B7D-5915-ACE9-ADFC96F5CC1B

##### Distribution

South Russian Far East to North Korea

#### Lonicera subsessilis

Rehder, 1920

67E7DD94-7A7C-5844-97FF-C59FB0F5A465

##### Distribution

Korea

#### Lonicera vidalii

Franch. & Sav., 1877

90A8B59D-F5AD-591B-B554-15398851B2D4

##### Distribution

Korea & Japan

#### Sambucus racemosa Kamtschatica

(E.Wolf) Hultén, 1930

CE2EDE49-94C3-5606-B014-B20F4C9CB2BF

##### Distribution

Korea, Kamchatka to Japan

#### Sambucus williamsii

Hance, 1866

FAA69F36-57AC-5FE8-ADD9-0AB3B0BFDE8E

##### Distribution

South Siberia to China and South Korea, Japan

#### Viburnum carlesii

Hemsl., 1888

29025CB4-9365-5633-BB7C-AD43228A516D

##### Distribution

SouthEast China, Korea, West & South Japan

#### Viburnum dilatatum

Thunb., 1784

C1C56E53-8637-5B90-954A-A99DBA87D6C2

##### Distribution

Central & South China to Temperate East Asia

#### Viburnum erosum

Thunb., 1784

916473EB-D155-590B-B6DB-20F5F03C1FAA

##### Distribution

Central & South China to Korea, Central & South Japan, Taiwan

#### Viburnum opulus var. calvescens

(Rehder) H.Hara, 1956

5AC17F2C-5742-526E-8C23-AF10AC74CB23

##### Distribution

Southeast Siberia to China and North & Central Japan

#### Weigela florida

(Bunge) A.DC., 1839

5624089B-9C49-5079-AB80-26D4A7672051

##### Distribution

South Russian Far East to North & East China and Korea, Japan

#### Weigela subsessilis

(Nakai) L.H.Bailey, 1929

BE7ACAE0-01F5-5810-AE28-EC4E79733945

##### Distribution

Korea

#### Zabelia biflora

(Turcz.) Makino ex Hisauti & H.Hara, 1954

A953F3D1-495B-5C7C-A301-FDAB3C57BA46

##### Distribution

South Russian Far East to North & East Central China and South Korea

#### Zabelia tyaihyonii

(Nakai) Hisauti & H.Hara, 1954

7340D257-66B9-5B7B-9A1F-BC4E5823D518

##### Distribution

Northeast China to Korea, Sakhalin

#### Patrinia rupestris

(Pall.) Dufr., 1811

2A4AEAB5-BEF0-583E-9379-61C2D51D93DE

##### Distribution

Siberia to Korea and China

#### Patrinia scabiosifolia

Fisch. ex Trevir., 1820

89063237-5FF2-5867-B273-8366AB53A617

##### Distribution

Southeast Siberia to Russian Far East and Vietnam, Japan

#### Patrinia villosa

(Thunb.) Dufr., 1811

3690B30A-6A72-560F-A307-2D28BF90F14B

##### Distribution

South China to Vietnam, Temperate East Asia

#### Valeriana fauriei

Briq., 1914

BAD9E69F-6865-597E-9C9F-BC310E1DB600

##### Distribution

Russian Far East to Temperate East Asia

#### Adenophora remotiflora

(Siebold & Zucc.) Miq., 1866

1F3C7EFC-47A5-5EFF-9C47-47F38B01282E

##### Distribution

South Russian Far East to North China and Japan

#### Adenophora triphylla

(Thunb.) A.DC., 1830

828D03E7-B650-5CAC-85B7-CBED0B98AF0B

##### Distribution

South Siberia to Sakhalin and North Indo-China

#### Adenophora triphylla var. japonica

(Regel) H.Hara, 1951

64E0CF7E-175A-5E8D-BCA3-551FC4EB5983

##### Distribution

South Siberia to Sakhalin and North Indo-China

#### Asyneuma japonicum

(Miq.) Briq., 1931

043C86EB-3D32-52CB-9C71-13D3FB7EE060

##### Distribution

Russian Far East to Korea, Japan

#### Campanula punctata

Lam., 1785

BBBA2995-914C-5FAA-9AB8-872ED5FFC628

##### Distribution

East Siberia to China and Japan

#### Codonopsis lanceolata

(Siebold & Zucc.) Benth. & Hook.f. ex Trautv., 1879

069100FC-4F6E-5102-84D0-D4286E8DEE60

##### Distribution

Russian Far East to China, Korea, Japan

#### Peracarpa carnosa

(Wall.) Hook.f. & Thomson, 1857

B4709727-1DE2-5932-BA3D-9367E6928BA5

##### Distribution

Kamchatka to Tropical Asia

#### Platycodon grandiflorus

(Jacq.) A.DC., 1830

7B8232BF-5B6D-580A-9F3D-9E52C93DD2F8

##### Distribution

SouthEast Siberia to Japan and China

#### Achillea alpina

L., 1753

2B542F84-DD36-52C6-A53D-F178EB318FFC

##### Distribution

Siberia to Japan and China

#### Adenocaulon himalaicum

Edgew., 1846

65B7903E-A815-501C-ACB0-BC12295BC60B

##### Distribution

Himalaya to Russian Far East and Japan

#### Ageratina altissima

(L.) R.M.King & H.Rob., 1970

4839EE1C-606C-5EFA-936B-321031B74D42

##### Distribution

East Canada to U.S.A.

#### Ainsliaea acerifolia

Sch.Bip., 1855

D14FEFD1-6452-5F67-A67C-D9F707957FAD

##### Distribution

NorthEast China to Korea, Central & South Japan

#### Ambrosia artemisiifolia

L., 1753

579D5975-0AC3-5079-AD75-2E705B8E7474

##### Distribution

Subarctic America to U.S.A.

#### Ambrosia trifida

L., 1753

54C865F4-328B-5690-93EF-8F7B6CDACE94

##### Distribution

North America

#### Artemisia angustissima

Nakai, 1915

7ED80D3A-E2E1-53FE-99F5-3C1091F169AE

##### Distribution

China, Korea & Japan

#### Artemisia capillaris

Thunb., 1780

82630642-3575-548C-A6A3-53D1F95B1FA2

##### Distribution

Pakistan to Japan

#### Artemisia codonocephala

Diels, 1912

3AA6555A-EE91-5B5B-9A33-66FB4C676828

##### Distribution

East Europe to Russian Far East & China

#### Artemisia indica

Willd., 1803

86143C82-8351-5AAB-827C-09B67678D192

##### Distribution

Indian Subcontinent to Japan & Philippines

#### Artemisia japonica

Thunb., 1780

058C99FE-CF4B-5D01-B8B5-0C1CBE9EB14E

##### Distribution

Afghanistan to Russian Far East & Philippines

#### Artemisia keiskeana

Miq., 1866

46D63EC9-783C-537F-BB8B-A539F2B0FF0E

##### Distribution

South Russian Far East to Japan & North China

#### Artemisia lancea

Vaniot, 1903

0A2ECAAF-7139-5F6F-A9AA-4381EA2679C5

##### Distribution

Mongolia to Korea, Japan & China

#### Artemisia rubripes

Nakai, 1917

C278A255-9E02-5A04-8C0D-B11BD720E7D0

##### Distribution

East Europe, Mongolia to Japan & China

#### Artemisia sacrorum var. iwayomogi

(Kitam.) M.S.Park & G.Y.Chung, 2016

67C46FCB-2156-5410-AB83-D8C2A7BBEA44

##### Distribution

Central Asia to Russian Far East and Himalaya, Japan

#### Artemisia selengensis

Turcz. ex Besser, 1832

95106DE6-F72A-51E3-B126-8DF1FADEEFDF

##### Distribution

South Siberia to Japan and Thailand

#### Artemisia stolonifera

(Maxim.) Kom., 1907

CECF5EAB-503B-53BB-866D-6E9D76C4884F

##### Distribution

Russian Far East to China

#### Aster ageratoides

Turcz., 1837

DD907A75-65C6-5744-962A-EF55ABD76A1D

##### Distribution

Himalaya to Temperate East Asia

#### Aster hispidus

Thunb., 1784

0A5A1398-0A4B-5375-B11D-37BE4FF716F3

##### Distribution

Mongolia to Korea, Japan and Vietnam

#### Aster incisus

Fisch., 1812

2403DD20-4D88-510F-A75E-A8DA339C77CE

##### Distribution

Southeast Siberia to Northeast China, Central & South Japan

#### Aster koraiensis

Nakai, 1909

40F70762-730B-59E8-BD13-0887E3D1A482

##### Distribution

Korea

#### Aster maackii

Regel, 1861

F40063C5-5EFD-5B3A-98C5-D60353B7C021

##### Distribution

Mongolia to Korea & Japan

#### Aster meyendorffii

(Regel & Maack) Voss, 1894

D3FB7441-C60A-5990-92F1-083D4E09AF24

##### Distribution

South Russian Far East to North China and Japan

#### Aster scaber

Thunb., 1784

8DC05AFD-4238-50F4-9995-E766EE8E8EAB

##### Distribution

China to Russian Far East and Japan

#### Aster tataricus

L.f., 1782

59BEC875-581F-520D-B379-7207879BC300

##### Distribution

South Siberia to Korea, Japan

#### Aster yomena

(Kitam.) Honda, 1938

C539AEDA-2A28-5EA2-AA1F-852881F1E3CF

##### Distribution

South Korea, Central & South Japan

#### Atractylodes ovata

(Thunb.) DC., 1838

12EDF383-6C09-5AD3-A7BE-15258472F4CC

##### Distribution

Russian Far East to China and Japan

#### Bidens bipinnata

L., 1753

F8292907-2AFB-5EEA-81C5-E96CD20D783B

##### Distribution

East Canada to Central & East U.S.A. and Arizona

#### Bidens biternata

(Lour.) Merr. & Sherff ex Sherff, 1929

AA36F945-F6CE-553E-B7E5-8CEAC3C540AF

##### Distribution

Tropical & Subtropical Old World

#### Bidens frondosa

L., 1753

C5F6964E-CFB7-52A1-9A55-E082D6C80D58

##### Distribution

Canada to U.S.A

#### Bidens parviflora

Willd., 1809

3E87933A-F2CF-5D60-8D7A-79B576BF7E25

##### Distribution

South Siberia to Japan and China

#### Bidens tripartita

L., 1753

63DCCC16-5083-534E-AC8C-1D81E5E29BC3

##### Distribution

Temperate Northern Hemisphere

#### Carduus crispus

L., 1753

EB575DB0-90DD-5FCD-A101-22E6AFFE31BF

##### Distribution

Europe to Siberia and Caucasus

#### Carpesium abrotanoides

L., 1753

01BFAC09-5C55-5D9D-9ED8-9DE97B207D3A

##### Distribution

South & Central Europe to Japan and Himalaya

#### Carpesium cernuum

L., 1753

6351146B-63A1-5957-9201-E06B65F6DB65

##### Distribution

Eurasia

#### Carpesium divaricatum

Siebold & Zucc., 1846

93D4F220-4B73-5917-8087-DBF3BE9CEC00

##### Distribution

China to Temperate East Asia

#### Carpesium macrocephalum

Franch. & Sav., 1878

3A73C710-260E-5107-A3EB-3AB4AB383050

##### Distribution

South Russian Far East to Japan

#### Chrysanthemum boreale

Makino, 1909

31F748D4-3FBA-5385-BB7E-9EEE09D2A642

##### Distribution

China to Korea, Japan

#### Cirsium japonicum var. maackii

(Maxim.) Matsum., 1912

C50FD996-6CE2-5A62-AF9F-59854E231213

##### Distribution

Russian Far East to Japan and Taiwan

#### Cirsium pendulum

Fisch. ex DC., 1838

85E81A65-E861-59C7-A189-A10C72A8DFD2

##### Distribution

South Siberia to Japan and China

#### Cirsium setidens

(Dunn) Nakai, 1920

C5DFA28C-72E0-54B0-8A09-70494D4ED0D1

##### Distribution

Korea

#### Conyza canadensis

(L.) Cronquist, 1943

97650A51-B5E4-55FB-BC36-03601FA294AF

##### Distribution

New World

#### Crepidiastrum chelidoniifolium

(Makino) J.H.Pak & Kawano, 1992

9BFCDB9D-7328-5B40-9E2A-A4FB254873FC

##### Distribution

South Russian Far East to Korea, Central & South Japan

#### Crepidiastrum denticulatum

(Houtt.) J.H.Pak & Kawano, 1992

8765C2D2-4FF0-5702-B329-A8F064465CAB

##### Distribution

Vietnam to Russian Far East and Japan

#### Crepidiastrum sonchifolium

(Maxim.) J.H.Pak & Kawano, 1992

5633CBBB-6DA3-5E20-B14B-DEAB58BAE9AF

##### Distribution

Mongolia to Russian Far East and North Indo-China

#### Dendranthema oreastrum

(Hance) Y.Ling, 1980

323D6D8B-8805-5568-8927-B6B0140E49F5

##### Distribution

South Russian Far East to North & Central China and Korea

#### Dendranthema zawadskii

(Herbich) Tzvelev, 1961

A6D80CE2-3560-52F6-9989-DDFDAE122632

##### Distribution

Siberia to Korea, Japan & China

#### Dendranthema zawadskii var. latiloba

(Maxim.) Kitam., 1978

F01C88C8-3B17-5DFB-9438-04FFCCDDF572

##### Distribution

Russian Far East to Japan & China

#### Eclipta thermalis

Bunge, 1833

FE5904A8-D0C6-5B05-8668-9E3227A091CE

##### Distribution

Temperate & Subtropical America

#### Erechtites hieraciifolius

(L.) Raf. ex DC., 1838

CEE942AD-7990-5938-894A-30760821F694

##### Distribution

East Canada to Tropical & Subtropical America

#### Erigeron annuus

(L.) Desf., 1804

D9F237AF-9AFF-5F6C-9E2F-7795D7B08E70

##### Distribution

Canada to U.S.A., Nicaragua to Panama

#### Erigeron philadelphicus

L., 1753

DB84C075-CC5E-5B34-A48D-6A2305EF6AA6

##### Distribution

Subarctic America to U.S.A.

#### Erigeron strigosus

Muhl. ex Willd., 1803

630B93BC-D4CF-5FCA-9C5E-15DAC2DD397C

##### Distribution

Canada to U.S.A.

#### Eupatorium japonicum

Thunb., 1784

F2C56701-9CF8-5A71-8BFF-1242F35F5693

##### Distribution

China to Japan and Vietnam

#### Eupatorium lindleyanum

DC., 1836

5F3431E1-DA09-5777-9A69-4FE339E2E797

##### Distribution

Russian Far East to Japan and Indo-China, Philippines

#### Eupatorium makinoi var. oppositifolium

(Koidz.) Kawah. & Yahara, 1995

B5848A98-48E0-506C-94FD-88FB20AA7406

##### Distribution

Russian Far East to Vietnam & Temperate East Asia

#### Eupatorium tripartitum

(Makino) Murata & H.Koyama, 1982

60328EC8-C6AC-56F3-8D29-E9F7E97C62F1

##### Distribution

Russian Far East to Vietnam & Temperate East Asia

#### Galinsoga quadriradiata

Ruiz & Pav., 1798

FE06F662-6F8E-5899-934C-C0AA6B9F10DE

##### Distribution

Mexico to South Tropical America

#### Hemisteptia lyrata

(Bunge) Fisch. & C.A.Mey, 1836

DA2A43B7-6F33-5A96-9515-7325611E7EF4

##### Distribution

Northeast India to Japan & Indo-China, East Australia

#### Hieracium umbellatum

L., 1753

9F36BBF8-988B-54E3-8246-C4CB346CFBBF

##### Distribution

Temperate Northern Hemisphere

#### Ixeridium dentatum

(Thunb.) Tzvelev, 1964

B4E72C21-4AA0-50B3-9323-EFB6894D562A

##### Distribution

China to Korea, Kuril Islands & Japan

#### Ixeris stolonifera

A.Gray, 1858

76C3172D-A323-52C1-9862-01174F2167ED

##### Distribution

Temperate East Asia

#### Ixeris strigosa

(H.Lév. & Vaniot) J.H.Pak & Kawano, 1992

7F9A12A5-4A51-5511-8B30-06DC11FBFAE1

##### Distribution

South China to Russian Far East and Temperate East Asia

#### Lactuca indica

L., 1771

31B17D2B-82A0-5E34-B02B-2AB62C451A62

##### Distribution

SouthEast Siberia to Japan and Malesia

#### Lactuca indica var. laciniata

(Houtt.) H.Hara, 1952

6FBC9053-78E6-5AE3-A4BF-E86AE6AFCB37

##### Distribution

Southeast Siberia to Japan and Malaysia

#### Lactuca raddeana

Maxim., 1874

D7FAEDAB-166C-591C-B095-4DDC71B9EF3E

##### Distribution

Russian Far East to Japan and Vietnam

#### Lactuca triangulata

Maxim., 1859

7D1220BB-80D9-58AB-9705-A446393545B3

##### Distribution

Russian Far East to North China and Japan, Vietnam

#### Leibnitzia anandria

(L.) Nakai, 1937

EB50FDEA-3772-512E-A7B9-4F01E24C22B6

##### Distribution

Temperate Asia

#### Ligularia fischeri

(Ledeb.) Turcz., 1847

C71A7866-CD19-5FB0-9C29-19F0BCBC9E56

##### Distribution

NorthEast Pakistan to South Siberia, Korea and Japan

#### Parasenecio auriculatus var. matsumurana

(Nakai) M.Kim, 2 017

8D068625-34DA-5098-BA20-A5C3CB72321E

##### Distribution

Russian Far East to North China, Korea and North Japan, Aleutian Islands (Alaska)

#### Petasites japonicus

(Siebold & Zucc.) Maxim., 1866

C1F348DE-B4B0-50A0-9F27-F5EAD3845F93

##### Distribution

China to South Russian Far East and Central & South Japan

#### Pseudognaphalium affine

(D.Don) Anderb., 1991

4198AFA9-B1C9-54CC-BF49-34C336008B42

##### Distribution

Caucasus to Temperate East Asia and Indo-China

#### Rhynchospermum verticillatum

Reinw., 1828

D5015AA2-7FAC-5920-A44C-39B4F773D888

##### Distribution

Nepal to Korea, Japan and Indo-China, Java

#### Saussurea gracilis

Maxim., 1874

0FAE45D9-0267-541C-9845-8B51B5885C47

##### Distribution

Korea, Central & South Japan

#### Saussurea macrolepis

(Nakai) Kitam., 1933

3131438B-0FA3-5BAD-AFA7-362D939110F8

##### Distribution

Korea

#### Saussurea odontolepis

(Herder) Sch.Bip. ex Maxim., 1883

8EE55830-8B31-59BA-AFCB-3B9EE8A51751

##### Distribution

Mongolia to Russian Far East and North China

#### Saussurea pulchella

(Fisch.) Fisch. ex Colla, 1834

8404463A-7098-589A-A350-6F05F7F26BC7

##### Distribution

SouthEast Siberia to Japan and North China

#### Saussurea seoulensis

Nakai, 1911

DAC649FE-1E87-56C5-9D37-847DDC45D3CB

##### Distribution

Korea

#### Scorzonera albicaulis

Bunge, 1833

55ACBF63-2041-5833-B0E3-91928831F748

##### Distribution

SouthEast Siberia to Russian Far East and China

#### Senecio vulgaris

L., 1753

CADF0D10-F83D-59DD-9021-30B2BDF2A3B0

##### Distribution

Macaronesia, Europe to China and Arabian Peninsula

#### Sigesbeckia glabrescens

(Makino) Makino, 1917

778E8375-34FA-5389-A384-16BA6136C4EC

##### Distribution

South China to Japan

#### Sigesbeckia orientalis pubescens

(Makino) H.Koyama, 1995

5A8A30A7-86CB-5BCF-8CB3-23CF2AB8BC2B

##### Distribution

China to Korea, Japan

#### Solidago altissima

L., 1753

4392EE76-311E-5C07-AC67-187445B25345

##### Distribution

Canada to Mexico

#### Solidago gigantea

Aiton, 1789

E4FDB1EC-559A-57BC-AE4E-510F6C71327D

##### Distribution

Canada to North East Mexico

#### Solidago virgaurea asiatica

(Nakai ex H.Hara) Kitam. ex H.Hara, 1952

31142037-1A1B-5F92-AFA3-1637FBEEAA76

##### Distribution

North Pakistan to Russian Far East and Philippines, Alaska (Aleutian Islands)

#### Sonchus asper

(L.) Hill, 1769

25123350-8A87-5D73-AE2B-126CD8E7F1D8

##### Distribution

Temperate Eurasia, North Africa to Sahel and Somalia

#### Sonchus oleraceus

L., 1753

461F3440-94F9-5A97-868B-1E19BC2A2DF6

##### Distribution

Macaronesia, Europe to Mediterranean, Sahara to Arabian Peninsula

#### Stemmacantha uniflora

(L.) Dittrich, 1984

6E78B920-94C0-59FC-8416-E2F14325D9F7

##### Distribution

South Siberia to Korea & Japan

#### Symphyotrichum pilosum

(Willd.) G.L.Nesom, 1995

C8740A95-CB04-5DC9-92AF-C9E1CB555C45

##### Distribution

East Canada to North Central & East U.S.A.

#### Syneilesis palmata

(Lam.) Maxim., 1874

3A10F667-6EBF-5C97-A10A-775FE857D3F4

##### Distribution

Korea, Central & South Japan

#### Synurus deltoides

(Aiton) Nakai, 1932

2A820527-795D-5DC0-8078-9E13420B83A2

##### Distribution

South Siberia to Central & South Japan and China

#### Tagetes minuta

L., 1753

72BE1633-95D5-57C4-83A5-D76E0BC12813

##### Distribution

Brazil to South America

#### Taraxacum officinale

F.H.Wigg., 1780

3B97B353-E8F1-5C30-B134-F108133EA94C

##### Distribution

Macaronesia, Europe to Siberia, North West Africa

#### Tephroseris flammea

(DC.) Holub, 1973

227F679C-067C-5033-B5CB-BA92651BCA77

##### Distribution

South Siberia to Japan and North China

#### Tephroseris kirilowii

(Turcz. ex DC.) Holub, 1977

995AF0D7-0DB8-59AE-A223-C835D4974421

##### Distribution

Siberia to Japan and China

#### Youngia japonica

(L.) DC., 1838

394D4692-680A-56F5-B7A4-9D6681FC35D9

##### Distribution

Tropical & Subtropical Asia

#### Potamogeton oxyphyllus

Miq., 1867

37286543-BCBC-591C-8040-D19DC6156D33

##### Distribution

South Russian Far East to North Sumatra

#### Allium macrostemon

Bunge, 1833

C9C98337-9C4D-5E3D-9ACA-2BDB0CADC09B

##### Distribution

Russian Far East to China and Japan

#### Allium tuberosum

Rottler ex Spreng., 1825

C418E167-1A18-5E4F-B3B3-B4652F7E9F7D

##### Distribution

Himalaya to China

#### Allium thunbergii

G.Don, 1827

A30DF5B8-5F3E-5462-8A4B-7E87B9303E4E

##### Distribution

East Central China to Temperate East Asia

#### Asparagus cochinchinensis

(Lour.) Merr., 1919

04148194-6C30-589F-A113-C2CB4694A940

##### Distribution

Japan to Indo-China and Philippines

#### Asparagus oligoclonos

Maxim., 1859

3FBF6A9A-A378-56CD-8C91-9C531941A55B

##### Distribution

Mongolia to Korea & Japan

#### Asparagus schoberioides

Kunth, 1850

D0DB4191-91AB-5495-BAFF-5E80B1C57F28

##### Distribution

Southeast Siberia to Korea & Japan

#### Barnardia japonica

(Thunb.) Schult.f., 1829

6B19C0E8-8AE8-514F-B905-8073A558898D

##### Distribution

China to Temperate East Asia

#### Convallaria keiskei

Miq., 1867

9A064448-E5BD-5F58-9F1D-0DDA7FD8B2E6

##### Distribution

SouthEast Siberia to Korea & Japan

#### Disporum smilacinum

A.Gray, 1857

3233B0DC-73D4-5654-B024-ECFB81E5BE00

##### Distribution

Sakhalin to China, Korea and Japan

#### Disporum uniflorum

Baker, 1875

B11283A0-5120-53FF-82A1-822B6E757D19

##### Distribution

China to Korea

#### Disporum viridescens

(Maxim.) Nakai, 1911

0EBE356E-ECAD-5CC3-9874-B3AB93F1C9CF

##### Distribution

Russian Far East to Korea, North & Central Japan

#### Erythronium japonicum

Decne., 1854

46D9EE25-342C-5C79-81C0-D60A770548A5

##### Distribution

Northeast China to Korea, South Sakhalin to Japan

#### Heloniopsis koreana

Fuse, N.S.Lee & M.N.Tamura, 2004

098D34C4-DE06-5A19-B3F3-1320ED840463

##### Distribution

Korea

#### Hemerocallis dumortieri

C.Morren, 1834

B004BB7F-CF2B-51C6-AFA1-5882677E74FB

##### Distribution

China (Jilin) & Korea

#### Hemerocallis fulva

(L.) L., 1762

9FD197E9-DE9D-5B7C-97AB-084A1AF5E816

##### Distribution

China to Temperate East Asia

#### Hemerocallis hakuunensis

Nakai, 1943

21EEA186-0DCF-5AD6-A0B1-F25E15C65F80

##### Distribution

Korea & Japan

#### Hosta capitata

(Koidz.) Nakai, 1930

A70DCC55-9BF1-51D9-BDC4-C8EAF82A30E4

##### Distribution

Korea & Japan

#### Hosta clausa

Nakai, 1930

71BE9C3C-2F4B-5104-9BA9-3EF4A7B66059

##### Distribution

Russian Far East to Korea

#### Hosta longipes

(Franch. & Sav.) Matsum., 1894

84C67094-D4EB-548B-BCBF-76B2ADD612DC

##### Distribution

Korea & Japan

#### Hosta minor

(Baker) Nakai, 1911

1B8DF75F-1D57-50BD-9247-5223F3C3B2B1

##### Distribution

Korea

#### Lilium amabile

Palib., 1901

9D168BE5-0152-599E-BDC2-80BA794CC9A7

##### Distribution

China (SouthEast Liaoning) to Korea

#### Lilium callosum

Siebold & Zucc., 1835

AFF2D3B8-870E-5372-984E-4BED88D365D1

##### Distribution

Russian Far East to East China and Temperate East Asia

#### Lilium distichum

Nakai ex Kamib., 1915

9303FEE0-90D5-5983-979B-32076B18CD61

##### Distribution

Russian Far East to Korea

#### Lilium lancifolium

Thunb., 1794

E84CCD30-3E66-521D-B828-21CAD93DEF17

##### Distribution

Russian Far East to Japan and Tibet

#### Lilium tsingtauense

Gilg, 1904

9048C372-106E-5967-B941-62F6D9426903

##### Distribution

China (Anhui, Shandong) to Korea

#### Liriope muscari

(Decne.) L.H.Bailey, 1929

94F1FD59-A902-5173-ACA9-684A5FF0A7A6

##### Distribution

China to Temperate East Asia

#### Liriope spicata

(Thunb.) Lour., 1790

C10F4928-C549-5649-8281-FD82C8EE4452

##### Distribution

Korea, Central & South Japan to Cambodia

#### Maianthemum japonicum

(A.Gray) LaFrankie, 1986

1F73E792-84CB-5A17-BC4B-DA98B8364F0D

##### Distribution

Russia Far East to Central China, Korea & Japan

#### Ophiopogon japonicus

(A.Gray) LaFrankie, 1986

005E5C33-1598-5B16-B7B3-E4E46EC61D8C

##### Distribution

Central & South China to Vietnam, Temperate East Asia to Philippines

#### Paris verticillata

M.Bieb., 1819

031E10BA-00F1-5BE4-8D3A-8FC84447FA1E

##### Distribution

North Kazakhstan to Siberia, Korea and Japan

#### Polygonatum inflatum

Kom., 1901

10598B57-A28F-56FF-A99C-15E55DD56FF6

##### Distribution

Primorye to Korea, South Central & South Japan

#### Polygonatum involucratum

(Franch. & Sav.) Maxim., 1883

3C0FFAB4-728E-5C78-917C-13734373A12E

##### Distribution

Russian Far East to Korea, Japan

#### Polygonatum lasianthum

Maxim., 1883

3A1F94A5-2FA9-53C6-8380-6A7FE7BEB8FD

##### Distribution

Korea & Japan

#### Polygonatum odoratum var. pluriflorum

(Miq.) Ohwi, 1949

CBC0D08F-B0A6-5B02-82E7-33FF1B5D4E17

##### Distribution

Korea & Japan

#### Polygonatum thunbergii

C.Morren & Decne., 1834

172DE7B9-EA2C-5701-AC0B-ED3C54B40973

##### Distribution

Russian Far East to Korea, Japan

#### Smilax china

L., 1753

2E2CC9A4-D214-5CFB-B252-0C20B1543007

##### Distribution

China to Japan and Philippines

#### Smilax nipponica

Miq., 1868

CD0E84D5-93A9-5F05-BFFA-847674C304B2

##### Distribution

China to Temperate East Asia

#### Smilax riparia

A.DC., 1878

234C1CE8-D744-5AED-ADF6-77352AE79992

##### Distribution

South Russian Far East to China and Philippines

#### Smilax sieboldii

Miq., 1868

17AFA43F-DC3A-5A8B-87E1-7D715E11D8AF

##### Distribution

East China to Temperate East Asia

#### Smilax sieboldii f. inermis

(Nakai) H.Hara, 1958

0CF995A0-06F3-5707-BD19-365337F8FB26

##### Distribution

East China to Temperate East Asia

#### Trillium camschatcense

Ker Gawl., 1805

9CAE062A-1FB9-52C5-8B54-6FD4FDB6D21C

##### Distribution

Russian Far East to Korea and North & North Central Japan

#### Tulipa edulis

(Miq.) Baker, 1874

2EEE2065-65E8-52A7-A4D4-BEDE84C492DF

##### Distribution

China to Korea, Central & South Japan

#### Veratrum maackii var. japonicum

(Baker) Shimizu, 1960

C11C04EF-598E-5D6A-8D41-F05730FE7089

##### Distribution

Korea & Japan

#### Veratrum oxysepalum

Turcz., 1840

4984C101-F5A2-532B-94B2-78CC97A27E97

##### Distribution

Siberia to Korea, West Alaska and Japan

#### Dioscorea japonica

Thunb., 1784

06E95A1F-1649-5E79-B232-9205E93BF1C2

##### Distribution

Assam to Korea & Japan

#### Dioscorea nipponica

Makino, 1891

B3109711-CA58-5BD9-B0A2-F0510E1B237C

##### Distribution

Central China to North & Central Japan

#### Dioscorea polystachya

Turcz., 1837

0E7F0E66-5328-57A6-9FE6-256504F734E5

##### Distribution

South Russian Far East to Central & South China, Korea and Kuril Islands, Taiwan

#### Dioscorea quinquelobata

Thunb., 1784

81898C0A-D659-5DD9-AB9B-772DA691A49C

##### Distribution

SouthEast China to Temperate East Asia

#### Dioscorea septemloba

Thunb., 1784

5EBA150E-B7CF-54D7-98DB-E3680CDC542F

##### Distribution

China to Korea, Central & South Japan

#### Dioscorea tokoro

Makino ex Miyabe, 1889

841AC065-5D27-5450-A852-0FF746CFFD3E

##### Distribution

China to Korea & Japan

#### Iris rossii

Baker, 1877

8A96CA76-7F6B-5A95-BF99-F609EC63588C

##### Distribution

NorthEast China (Liaoning) to Korea, SouthWest & South Japan

#### Iris sanguinea

Hornem., 1813

08DC1BB1-AB0A-5AFA-8CC8-874D3782A43D

##### Distribution

South Siberia to Korea & Japan

#### Juncus decipiens

(Buchenau) Nakai, 1928

4DF445C0-5620-54D1-BBAF-56DF5B7CFBA4

##### Distribution

Assam to Russian Far East and New Guinea

#### Juncus papillosus

Franch. & Sav., 1876

AC93709C-4C43-59E7-ABE6-4C17144FD8E3

##### Distribution

Russian Far East to East China and Japan

#### Juncus tenuis

Willd., 1799

1F3BC988-C21F-53A1-BB13-11D1FC4B1FFB

##### Distribution

East Canada to Mexico

#### Luzula capitata

(Miq. ex Franch. & Sav.) Kom., 1927

9E0F9D8D-A907-5BC8-8527-0C20C86F3F9A

##### Distribution

Russian Far East to Korea & Japan

#### Luzula multiflora

(Ehrh.) Lej., 1811

02A1C484-D5A4-5B31-A2F3-3A66C6067DB1

##### Distribution

Subarctic & Temperate Northern Hemisphere, Costa Rica, Argentina to Falkland Islands

#### Luzula rufescens var. macrocarpa

Buchenau, 1906

95934191-8181-5F7A-9668-13E74C16D884

##### Distribution

Russian Far East to Korea

#### Commelina communis

L., 1753

7F956CB7-AE5C-5659-9907-DDA217BD187D

##### Distribution

East Europe to Korea, Japan and Indo-China

#### Streptolirion volubile

Edgew., 1845

C2F903CD-52C8-56EC-9458-651A84429B58

##### Distribution

Himalaya to Primorye and Indo-China

#### Achnatherum pekinense

(Hance) Ohwi, 1953

361B917A-902E-5C2D-9632-276785539CAF

##### Distribution

North & Central China to Russian Far East and Japan

#### Agrostis clavata

Trin., 1821

852D3BAE-E30D-5C7F-B7D1-53CD1DEC4399

##### Distribution

North Europe to Temperate East Asia, New Guinea, Alaska to Yukon

#### Agrostis clavata var. nukabo

Ohwi, 1941

682D0928-0D6F-5A8B-884B-809918FAF6AE

##### Distribution

North Europe to Temperate East Asia, New Guinea, Alaska to Yukon

#### Alopecurus aequalis

Sobol., 1799

B1907DA7-BFE9-5D7A-AA8C-F6556CEA1538

##### Distribution

Temperate Northern Hemisphere to Andes

#### Arthraxon hispidus

(Thunb.) Makino, 1912

43B0F055-C5E9-5FC1-839D-9FF2987EF347

##### Distribution

Tropical Africa, West Indian Ocean, Asia to East Australia

#### Arundinella hirta

(Thunb.) Tanaka, 1925

646D310A-64BD-5D36-876D-A18C453B4483

##### Distribution

South Siberia to Temperate East Asia and North Indo-China

#### Arundinella hirta var. ciliata

(Thunb.) Koidz., 1925

7049DA42-5E8D-53F6-B087-C31D3D66FBC0

##### Distribution

South Siberia to Temperate East Asia and North Indo-China

#### Beckmannia syzigachne

(Steud.) Fernald, 1928

AE7331F7-FF1F-51D4-A49E-97C9836E258E

##### Distribution

European Russia to Russian Far East, Subarctic America to North & West U.S.A.

#### Bothriochloa ischaemum

(L.) Keng, 1936

CA6449CE-BF49-5969-8340-09DCD7095F9E

##### Distribution

Temperate Eurasia, NorthWest Africa

#### Brachyelytrum japonicum

(Hack.) Matsum. ex Honda, 1930

55BDADF5-F148-5ABA-B7CA-9879BB85A32E

##### Distribution

Central & East China, South Korea (including Jeju-do), Central & South Japan

#### Bromus japonicus

Thunb., 1784

00BACFBA-EE41-5FFF-AD76-C5BD6A44E4D2

##### Distribution

Mediterranean to Temperate Eurasia

#### Calamagrostis arundinacea

(L.) Roth, 1788

F294C7AB-408D-560B-848E-8FED1D72D436

##### Distribution

Temperate Eurasia, New Guinea

#### Calamagrostis purpurea

(Trin.) Trin., 1824

CE4D2E04-EDBD-5569-8FB1-0E7BA1AACF02

##### Distribution

Subarctic & Subalpine

#### Cleistogenes hackelii

(Honda) Honda, 1936

BA320127-5B04-5978-945C-044BA5A31DE4

##### Distribution

South Russian Far East to China, Korea and Japan

#### Cymbopogon goeringii

(Steud.) A.Camus, 1921

53AEDF90-70CB-5BAC-9302-62F3D06E4AE6

##### Distribution

Central & South China to Vietnam, Temperate East Asia

#### Dactylis glomerata

L., 1753

37E272B3-FC36-554F-86AE-29E1F624BAE0

##### Distribution

Macaronesia, Mediterranean to Temperate Eurasia

#### Diarrhena fauriei

(Hack.) Ohwi, 1941

32326DDF-6679-5A19-90E3-2197C6888506

##### Distribution

South Russian Far East to East China and Central Japan

#### Diarrhena japonica

Franch. & Sav., 1878

BDF862DF-D21F-592D-80C6-2EDF0807413C

##### Distribution

Northeast China, Korea (Jeju-do), Kuril Islands to Japan

#### Diarrhena mandshurica

Maxim., 1888

1E23FCDA-0976-51AE-B9C7-9712D4CA3CBF

##### Distribution

Russian Far East to North & East China

#### Digitaria ciliaris

(Retz.) Koeler, 1802

A0C7A9B6-49C5-5717-BA69-8CA51EC01F99

##### Distribution

Tropical & Subtropical Old World

#### Echinochloa crus-galli

(L.) P.Beauv., 1812

FDDA93B8-F8A7-5380-92CE-F69FC42E8C3C

##### Distribution

South & East Europe to Asia, West, East & South Tropical Africa to South Africa, Madagascar

#### Eleusine indica

(L.) Gaertn., 1788

7AED8B36-CE7B-5B22-884A-EAEA0E3D3A2A

##### Distribution

Tropical & Subtropical Old World

#### Elymus ciliaris

(Trin. ex Bunge) Tzvelev, 1972

72D38DA4-106C-5632-9A53-6A3D2FF49E10

##### Distribution

Assam to Russian Far East and Temperate East Asia

#### Elymus tsukushiensis var. transiens

(Hack.) K.Osada, 1990

3F92EAEE-BBB8-5422-8573-D56E9C99CF53

##### Distribution

South Russian Far East to China and Temperate East Asia

#### Eragrostis ferruginea

(Thunb.) P.Beauv., 1812

0A1BA6AA-6C33-51D4-81F9-51D40AD77E21

##### Distribution

Himalaya to Temperate East Asia

#### Eriochloa villosa

(Thunb.) Kunth, 1829

E34F7D0B-74E7-59E6-81DD-A42BB67EAAEB

##### Distribution

Russian Far East to Vietnam and Temperate East Asia

#### Festuca arundinacea

Schreb., 1771

11D0B14C-3E14-5998-9AA2-21E2FB8BD853

##### Distribution

Europe to Xinjiang and Himalaya, Macaronesia to North Africa

#### Festuca ovina

L., 1753

F4C0E22E-A94E-5283-B9E6-300702030337

##### Distribution

Temperate Eurasia, Alaska to West U.S.A.

#### Festuca parvigluma

Steud., 1854

42534DA1-FE9C-571C-A3EA-A43DE20BE8A3

##### Distribution

Nepal to Temperate East Asia

#### Festuca rubra

L., 1753

A4B5401D-0717-57B9-936E-1DB00460E1FA

##### Distribution

Subarctic & Temperate Northern Hemisphere to Mexico

#### Hierochloe odorata

(L.) P.Beauv., 1812

69C0AF79-D38A-545A-9AB8-2A2E06CFCE21

##### Distribution

Subarctic & Temperate Northern Hemisphere

#### Hystrix longe-aristata

(Hack.) Honda, 1930

54B39BF2-B556-58A5-850D-351F6B943137

##### Distribution

West & Central Himalaya to Korea & Japan

#### Koeleria macrantha

(Ledeb.) Schult., 1824

30B6553B-D7B5-55D9-B6DC-A084E6498B8A

##### Distribution

Temperate Northern Hemisphere to Mexico

#### Melica nutans

L., 1753

8D53B907-3C76-552D-8047-C448FECE9B01

##### Distribution

Temperate Eurasia

#### Melica onoei

Franch. & Sav., 1878

325BC96D-B92A-57CA-A968-F7499B8D4922

##### Distribution

Pakistan to China, Temperate East Asi

#### Melica scabrosa

Trin., 1833

051FCF18-B85D-553B-A8EA-266CAB8AD509

##### Distribution

Tibet to Mongolia and Korea

#### Microstegium vimineum

(Trin.) A.Camus, 1922

E98AC39D-AA13-58A4-819B-467EB3B01E39

##### Distribution

Himalaya to Japan and Jawa

#### Milium effusum

L., 1753

68E10BE6-7276-5F78-A30C-89FAC9376B62

##### Distribution

Temperate Northern Hemisphere

#### Miscanthus sinensis var. purpurascens

(Andersson) Matsum., 1895

F08384E7-E582-5BAF-A6F6-4C7B04098D60

##### Distribution

China to Malesia, Russian Far East to Temperate East Asia

#### Muhlenbergia hakonensis

(Hack. ex Matsum.) Makino, 1917

6F353B5B-EB50-5202-8F4F-9C87F8626C4C

##### Distribution

China (Sichuan, Anhui), Korea, South Central & South Japan

#### Muhlenbergia japonica

Steud., 1854

372BD959-44E9-586D-87D4-907BB7A46999

##### Distribution

China to Russian Far East and Japan

#### Oplismenus undulatifolius

(Ard.) P.Beauv., 1812

E134B19E-AB83-580D-BBAE-A3E4BEE5E60E

##### Distribution

South Central & South Europe to Iran, Cameroon, Ethiopia to South Africa, West Indian Ocean, Central & South China to Japan and Tropical Asia, East Australia

#### Panicum bisulcatum

Thunb., 1815

AA208F83-DCD3-5C2E-9305-40296C0335A5

##### Distribution

Assam to Russian Far East and Central Malesia

#### Paspalum thunbergii

Kunth, 1829

FC7A9E06-DD1A-52D5-9C61-21018BD2C5D6

##### Distribution

East Himalaya to Temperate East Asia

#### Pennisetum alopecuroides

(L.) Spreng., 1824

52CD9A89-9C26-5320-A084-6F43A18BD7A2

##### Distribution

China to Temperate East Asia and West & Central Malesia, North West & East Australia

#### Phaenosperma globosum

Munro ex Benth., 1881

129150C5-54A8-55EB-83A2-0E97C346DDB0

##### Distribution

China to Assam, Temperate East Asia

#### Phalaris arundinacea

L., 1753

B0740CD2-4620-5C6E-8AC4-B478A33D6890

##### Distribution

Temperate & Subtropical Northern Hemisphere to Tropical Mountains

#### Phragmites australis

(Cav.) Trin. ex Steud., 1841

A8D2DDD7-CD27-5F93-B871-26C6293D5FB3

##### Distribution

Temperate & Subtropical to Tropical Mountains

#### Phragmites japonicus

Steud., 1854

FE5C8189-7A53-5469-9548-A6E81DC842B0

##### Distribution

Russian Far East to Korea & Japan

#### Poa acroleuca

Steud., 1854

E1B9B084-F6DE-5C11-84D1-711DEA8E4B48

##### Distribution

Tibet to Sakhalin and Temperate East Asia

#### Poa annua

L., 1753

605CCDE1-4C0B-5B64-A270-17D635114114

##### Distribution

Temperate Old World to Tropical Mountains

#### Poa hisauchii

Honda, 1928

43CF5271-0DB2-523C-98EA-A9C463824AED

##### Distribution

Primorye, China (Hebei, Zhejiang), South Korea, South Kuril Islands to Japan

#### Poa matsumurae

Hack., 1899

75BF7D4B-2679-5239-A232-B6898C083506

##### Distribution

North Korea & Japan

#### Poa nemoralis

L., 1753

D8524702-57CE-527A-B580-C6BBD7B30A6F

##### Distribution

Subarctic & Temperate Northern Hemisphere, NorthWest Africa

#### Poa pratensis

L., 1753

67CEF79E-3188-5E4D-A2CD-310E8C60EDF0

##### Distribution

Subarctic to Temperate Northern Hemisphere and North Mexico

#### Poa sphondylodes

Trin., 1833

CE822FFD-320E-5BFB-A3E1-003A09540B1E

##### Distribution

South Siberia to Temperate East Asia

#### Poa viridula

Palib., 1902

7578BF0D-B6EF-5D2E-A35D-99D8C119FCE4

##### Distribution

Azores, Morocco, Temperate Eurasia

#### Sasa borealis

(Hack.) Makino & Shibata, 1901

D1B6F77C-CA57-5064-97B9-F1015B05EDCC

##### Distribution

Korea, Sakhalin to Japan

#### Sasa quelpaertensis

Nakai, 1933

54183617-AD8A-5781-B3A7-BFC74EA06351

##### Distribution

Korea, Sakhalin to Japan

#### Schizachyrium brevifolium

(Sw.) Nees ex Büse, 1854

389C4CD7-2F81-531D-942E-EF4DFC40EC80

##### Distribution

Tropics & Subtropics

#### Setaria faberi

R.A.W.Herrm., 1910

9FFD0589-F05D-5A0A-B0C0-E373FD6EB0F4

##### Distribution

West Siberia, Russian Far East to China, Temperate East Asia

#### Setaria pumila

(Poir.) Roem. & Schult., 1817

4779B20C-ABEF-5DBE-97EA-C609D25EA076

##### Distribution

Old World

#### Setaria viridis

(L.) P.Beauv., 1812

3157E5AF-22AC-55C1-9567-481E140EC2B6

##### Distribution

Old World to Central & SouthEast Australia

#### Sibirotrisetum bifidum

(Thunb.) Barberá, 2019

98FEC17E-3B27-5C8B-BF81-DFCA2EA40CAF

##### Distribution

China to Temperate East Asia, New Guinea

#### Spodiopogon cotulifer

(Thunb.) Hack., 1889

E3434D1F-25ED-5B1E-8AE2-01DAB950890D

##### Distribution

West Himalaya, Assam to Central & South China, Temperate East Asia

#### Spodiopogon sibiricus

Trin., 1820

7EC5C8C8-2E60-5AB1-A9F1-6E7FF7B9A112

##### Distribution

Siberia to Japan and China

#### Sporobolus piliferus

(Trin.) Kunth, 1833

226F91D9-4B76-5D2A-A069-4194080CBE80

##### Distribution

Tropics & Subtropics

#### Themeda triandra

Forssk., 1775

3559890B-AB99-5CBF-86A8-3013C664A433

##### Distribution

Africa, Tropical & Subtropical Asia to Australia

#### Vulpia myuros

(L.) C.C.Gmel., 1805

EEBF23BD-2199-5046-8BFE-93B9A4A26F38

##### Distribution

Europe to Korea, Taiwan and Sri Lanka, Macaronesia to Arabian Peninsula and North Tanzania

#### Zoysia japonica

Steud., 1854

926AD525-8E4D-5F87-A875-421AB2174E52

##### Distribution

South Russian Far East to East China and Temperate East Asia

#### Arisaema amurense

Maxim., 1859

7A10F12A-07E1-5A65-B766-0A9F1F04CADE

##### Distribution

South Russian Far East to Korea

#### Arisaema amurense f. serratum

(Nakai) Kitag., 1939

B3357208-CE47-51CE-9A7F-494B46FBC138

##### Distribution

South Russian Far East to Korea

#### Arisaema heterophyllum

Blume, 1836

8E7E9B48-59BF-5583-92D4-461B963C7308

##### Distribution

China to Temperate East Asia

#### Arisaema ringens

(Thunb.) Schott, 1832

69D84622-2798-5AE4-9F68-615C3C7422E4

##### Distribution

East China (Zhoushan Islands), South Korea, Central Japan to Taiwan

#### Arisaema serratum

(Thunb.) Schott, 1832

FE148E70-656F-5078-BA17-1610C46497CA

##### Distribution

Primorye to Korea, South Kuril Islands to Japan

#### Arisaema thunbergii

Blume, 1836

803754B8-0E5D-5764-89E6-B655585311DC

##### Distribution

Temperate East Asia

#### Pinellia ternata

(Thunb.) Makino, 1901

F8FBCD8F-1544-5385-BDF7-4B4BD46F3280

##### Distribution

China to Temperate East Asia

#### Typha angustifolia

L., 1753

85C467FA-D599-5D99-AC8F-AB9235247E63

##### Distribution

Temperate Northern Hemisphere

#### Carex accrescens

Ohwi, 1931

F5FA6B14-63D2-5E91-BF2A-62906D6CE716

##### Distribution

Siberia to Korea, North & North Central Japan

#### Carex aphanolepis

Franch. & Sav., 1878

36E7DAF3-27EF-5076-BA56-AB3BD41B984A

##### Distribution

Russian Far East to East China, Japan, Vietnam

#### Carex bostrychostigma

Maxim., 1886

26575BA2-F326-5118-B3A7-5C05D6CFADEB

##### Distribution

Russian Far East to East China, Central & South Japan

#### Carex breviculmis

R.Br., 1810

59499BF5-7CB2-59F9-A712-136D47A737A4

##### Distribution

India to South China and New Zealand

#### Carex brevispicula

G.H.Nam & G.Y.Chung, 2020

810680B6-08A4-5AFB-AE80-1949EAEE1977

##### Distribution

Korea

#### Carex ciliato-marginata

Nakai, 1914

B50ED2A9-4208-5EC5-8405-23DC45FC5515

##### Distribution

East China to Central & South Japan

#### Carex fernaldiana

H.Lév. & Vaniot, 1901

10B5C0BF-0196-5BF6-96CC-8BEBE4CCC634

##### Distribution

Russian Far East to East China and Japan

#### Carex filipes

Franch. & Sav., 1878

A3414FDE-5CC1-5966-AFD8-45E61C6C07E6

##### Distribution

South China to Korea & Japan

#### Carex filipes var. oligostachys

Kük., 1909

FF8FF9D8-143B-5BF7-B2D6-5505483391E8

##### Distribution

South Russian Far East to North China, Korea & Japan

#### Carex forficula

Franch. & Sav., 1878

13218991-AC19-5823-98CF-90018CE3A4D0

##### Distribution

Russian Far East to East China, Korea & Japan

#### Carex fusanensis

Ohwi, 1932

D87F3400-A6BA-504C-B822-79399EB0B8F1

##### Distribution

Korea

#### Carex gibba

Wahlenb., 1803

5C30B4C0-CC2B-50C0-9A79-C8B547C086BD

##### Distribution

Korea to Vietnam, Central & South Japan

#### Carex heterolepis

Bunge, 1833

93DCB2E7-41BB-5293-B441-B73E354F304F

##### Distribution

South Siberia to China, Korea & Japan

#### Carex humilis var. nana

(H.Lév. & Vaniot) Ohwi, 1936

AD0A6BE0-8858-583B-96B9-074E3C25F448

##### Distribution

Siberia to Korea & Japan

#### Carex japonica

Thunb., 1784

BA3278AF-D441-553E-B56F-C8B94D578505

##### Distribution

China to Korea, Sakhalin and Japan

#### Carex laevissima

Nakai, 1914

63281B4F-7016-5A53-97B3-C984329AA3E9

##### Distribution

South Siberia, South Russian Far East to Korea, Japan

#### Carex lanceolata

Boott, 1857

7FFFFDE2-72C0-5F4F-9ED0-8F55F1CAA7A7

##### Distribution

Siberia to Korea & Japan

#### Carex leiorhyncha

C.A.Mey., 1831

A7956EC0-5431-54EF-9868-DD4A15C302D5

##### Distribution

Siberia to North China & Korea

#### Carex lenta

D.Don, 1824

8CE37F69-F2C6-5170-896B-C9BC20DD4F2D

##### Distribution

Central & South Japan to Korea & Tropical Asia

#### Carex mira

Kük., 1905

3F881297-7295-517F-A663-B36722066352

##### Distribution

Korea & Japan

#### Carex neurocarpa

Maxim., 1859

29ED5172-F601-5F25-9EE8-A197D3348BEC

##### Distribution

South Russian Far East to North & East China and Central Japan

#### Carex okamotoi

Ohwi, 1936

6E11613F-E483-5D89-8A5F-2CED21B8333D

##### Distribution

Korea

#### Carex onoei

Franch. & Sav., 1875

5A200686-611D-571A-9FCE-1902ECF58E02

##### Distribution

South Russian Far East to North & East China, North & Central Japan

#### Carex pediformis

C.A.Mey., 1831

85764CB3-0D5B-53F2-85EF-0ECE52C698F8

##### Distribution

East Europe to Korea

#### Carex pediformis var. pedunculata

Maxim., 1859

0A212EFC-31DC-5CF5-B4BD-632A9AD2ED8B

##### Distribution

Russian Far East to Korea

#### Carex polyschoena

H.Lév. & Vaniot, 1903

CAA56CC8-DE94-5F28-A928-96C4E435ACC5

##### Distribution

Russian Far East to East China and Japan

#### Carex siderosticta

Hance, 1873

5AC213E7-D4FB-5F20-A744-FF2255A3AB7D

##### Distribution

Russian Far East to East China, Korea & Japan

#### Carex tristachya

Thunb., 1784

FCFE5FD7-2943-5FD4-BCA1-91C6510A0BA2

##### Distribution

South China to Temperate East Asia, Malesia to New Guinea

#### Carex ussuriensis

Kom., 1901

89A8EB77-120D-5F7D-9435-03188CCCA1CE

##### Distribution

Russian Far East to North China

#### Cyperus amuricus

Maxim., 1859

B35DEEA3-B5FB-5822-91AF-A14775B5BF3E

##### Distribution

Russian Far East to China and Temperate East Asia

#### Cyperus difformis

L., 1756

49D3961F-EAB7-5C42-80B6-712D16607060

##### Distribution

Tropical & Subtropical Old World

#### Cyperus iria

L., 1753

F2597201-6CA0-53BD-A651-122D52DFB097

##### Distribution

Tropical & Subtropical Old World to Central Asia

#### Cyperus microiria

Steud., 1854

C722107B-ADB5-5A0D-AE01-D23B6416AFE4

##### Distribution

Japan to Himalaya

#### Kyllinga brevifolia var. leiolepis

(Franch. & Sav.) H.Hara, 1938

87992BE4-FD2A-5AA3-B4C7-C906476B60D6

##### Distribution

Central Himalaya to Korea & Japan

#### Scirpus wichurae

Boeck., 1870

FDFB70EE-D3D9-5F5A-897D-A0335EB3EC46

##### Distribution

East Himalaya to Korea & Japan

#### Trichophorum polygamum

D.C.Son & K.S.Chang, 2019

8BD773D2-D7DA-51E2-A1D3-43C5C45E476C

##### Distribution

Korea

#### Zingiber mioga

(Thunb.) Roscoe, 1807

CA522945-3049-504A-8B62-479011847D46

##### Distribution

South China to South Central & South Japan

#### Calanthe discolor

Lindl., 1838

A44145A4-5B08-5465-BA75-3FC1BC667661

##### Distribution

South China to South Korea & Japan

#### Cephalanthera erecta

(Thunb.) Blume, 1859

4F4B4BF2-14AD-5F84-9A6C-AF289F5FA0FA

##### Distribution

Nepal to Temperate East Asia

#### Cephalanthera falcata

(Thunb.) Blume, 1859

E9407F1F-178E-5362-AA4B-67F98EC30A41

##### Distribution

South China, South Korea, Central & South Japan

#### Cephalanthera longibracteata

Blume, 1859

62CCB88D-088C-53F7-B738-0375890A729E

##### Distribution

Russian Far East to Korea, Japan, Laos

#### Cymbidium goeringii

(Rchb.f.) Rchb.f., 1852

6132E568-13FB-580E-A368-D1B5E6736F60

##### Distribution

Himalaya to Korea & Japan

#### Cyrtosia septentrionalis

(Rchb.f.) Garay, 1986

3C40AA05-AD85-52D6-A0C6-768446322AE6

##### Distribution

SouthEast China, South Korea, Japan to Taiwan

#### Gastrodia elata

Blume, 1856

8F344C4E-868F-5163-9C56-2CE2282E9A69

##### Distribution

Himalaya to Russian Far East and Temperate East Asia

#### Goodyera henryi

Rolfe, 1896

E58FF68C-F338-524D-A216-6A96364B684A

##### Distribution

Central Nepal, Central & South China to South Kuril Islands and Taiwan

#### Goodyera repens

(L.) R.Br., 1813

6EE0C369-A111-509B-8D96-0383C6B0A0D7

##### Distribution

Temperate Northern Hemisphere

#### Goodyera schlechtendaliana

Rchb.f., 1850

48E3DEC3-0D79-5A4A-BA7B-3536E96F0EB4

##### Distribution

Tibet to Korea, Japan and Sumatra

#### Hemipilia gracilis

(Blume) Y.Tang, H.Peng & T.Yukawa, 2015

76E691D3-5E2E-5BD7-8B87-06DE120C80AE

##### Distribution

China to Temperate East Asia

#### Liparis kumokiri

F.Maek., 1936

8E2F8AED-938D-56A7-A798-027EC2B482B3

##### Distribution

South Russian Far East to Korea, Central & South Japan

#### Liparis suzumushi

Tsusumi, T.Yukawa & M.Kato, 2019

30D98D73-D69F-5B21-B302-CE01371A2372

##### Distribution

Korea & Japan

#### Spiranthes sinensis

(Pers.) Ames, 1908

CAC4AAB6-8389-550C-8658-F9F760CBE081

##### Distribution

Assam to South Central Japan and New Caledonia

## Analysis

### Vascular flora of algific talus slopes in South Korea

Vascular plants of the 25 algific talus slopes distributed in South Korea included 1,052 taxa of 125 families, 486 genera, 947 species, 23 subsp., 75 var. and 7 f. (Suppl. materials 2, 3). Pteridophytes included a total of 77 taxa (7.3%) of 16 families, 35 genera, 74 species, 1 subsp., 2 var. and 2 f.; gymnosperms included a total of 14 taxa (1.3%) of 5 families, 9 genera, 14 species; angiosperms included a total of 771 taxa (73.3%) of 92 families, 352 genera, 682 species, 22 subsp., 62 var. and 5 f. for dicotyledons and a total of 190 taxa (18.1%) of 12 families, 90 genera, 177 species, 11 var. and 2 f. for monocotyledons. These accounted for approximately 22.27% of all vascular plants in South Korea (4,724 species) (Korea National Arboretum 2023). The plant family that showed the largest number of detected species was Asteraceae (96 taxa, 9.1%), followed by Poaceae (70 taxa, 6.7%), Rosaceae (65 taxa, 6.2%), Fabaceae (49 taxa, 4.7%), Liliaceae (44 taxa, 4.2%), Ranunculaceae (39 taxa, 3.7%) and Cyperaceae (36 taxa, 3.4%).

The most frequently detected species across the 25 algific talus slopes in South Korea was *P.tricuspidata* (n = 23), followed by *F.rhynchophylla*, *L.obtusiloba* and *R.mucronulatum* (n = 21); A.glandulosavar.brevipedunculata and *P.tenuifolius* (n = 20); U.davidianavar.japonica, *A.arguta*, *C.japonica*, *L.maximowiczii*, *V.acuminata* and *O.undulatifolius*. Magnoliophyta (n = 19); A.platanifoliumvar.trilobum, *A.yokoscense*, *E.alatus*, and *D.crassirhizoma* (n = 18); *T.trichocarpum*, *S.suffruticosa*, P.odoratumvar.pluriflorum, *Z.schinifolium*, *Q.mongolica*, *A.pseudosieboldianum* and *R.crataegifolius* (n = 17); 9 taxa, including *C.lanceolata* and *A.ageratoides* (n = 16); 11 taxa, including A.pictumvar.mono and *A.scaber* (n = 15); 11 taxa, including *D.chinensis* and *C.heterophylla* (n = 14); 15 taxa, including *M.amurensis* and *V.collina* (n = 13); 11 taxa, including *Q.aliena*, *S.incisa* and *S.sawafutagi*. (n = 12); 24 taxa, including *A.reflexipinnum*. and *A.incisum* (n = 11). In contrast, plants of 783 taxa were detected five or less times: 321 taxa, including C.ochotensisvar.raddeana and *A.longecassidatum* were detected once; 209 taxa, including *P.aizoon* and *S.trichocarpa* were detected twice; 112 taxa, inluding *R.davurica* and *U.laciniata* were detected three times; 75 taxa, including *L.glauca* and *D.mandshurica* were detected four times; *B.chinensis* and *V.orientalis* were detected five times.

### Rare plants and Red list species

**Figure 2.**
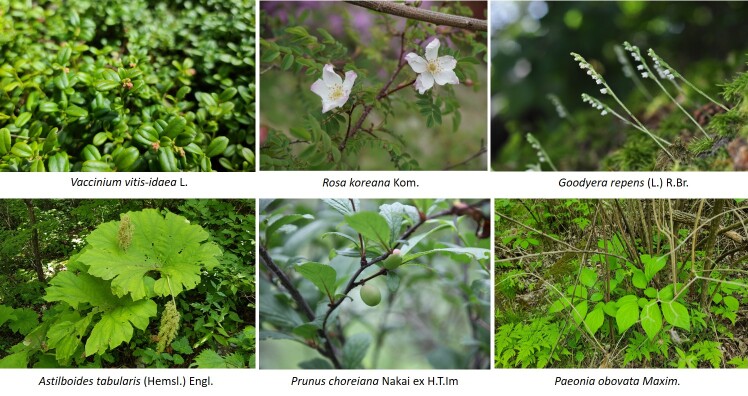
Pictures showing rare plants and Red list on the algific talus slope in South Korea.

A total of 55 taxa (Suppl. material 4, Fig. 2) detected across the 25 algific talus slopes in South Korea were found to be rare plants designated by the Korea Forest Service. Seven taxa were Critically Endangered (CR) species, including *Cystopterisfragilis* (L.) Bernh., AspleniumtrichomanesL.subsp.quadrivalens D.E. Mey., *Aconitumcoreanum* (H. Lév.) Rapaics, *Paeoniaobovata* Maxim., Vacciniumvitis-idaea L., *Cyrtosiaseptentrionalis* (Rchb.f.) Garay and Goodyerarepens (L.) R.Br. Ten taxa were Endangered (EN) species, including *Zabeliatyaihyonii* (Nakai) Hisauti & H. Hara, *Astilboidestabularis* (Hemsl.) Engl., *Micranthesoctopetala* (Nakai) Y.I. Kim & Y.D. Kim, *Deutziapaniculata* Nakai, *Rosakoreana* Kom. and *Sophorakoreensis* Nakai. Fifteen taxa were Vulnerable (VU) species, including *Adiantumpedatum* L., *Athyriumspinulosum* (Maxim.) Milde, *Piceajezoensis* (Siebold & Zucc.) Carrière, *Illiciumanisatum* L. and *Aconitumaustrokoreense* Koidz. Twenty taxa were Least Concern (LC) species, including *Lycopodiumannotinum* L., *Selaginellahelvetica* (L.) Spring, *Aristolochiamanshuriensis* Kom., GentianatrifloraPall.var.japonica (Kusn.) H. Hara and *Goodyeraschlechtendaliana* Rchb.f. Three taxa were Data Deficient (DD) species, including *Gymnocarpiumdryopteris* (L.) Newman, Eleutherococcusdivaricatus(Siebold & Zucc.)S.Y. Huvar.chiisanensis (Nakai) C.H. Kim & B.-Y. Sun and *Pseudolysimachionpyrethrinum* (Nakai) T. Yamaz.

A total of 52 taxa (Suppl. material 4) detected across the 25 algific talus slopes in South Korea were found to be Red List species of vascular plants on the Korean Peninsula, accounting for 9.58% of the 543 taxa of Red List species in total. While there were no CR species, four taxa were EN species, including *Piceajezoensis* (Siebold & Zucc.) Carrière, *Paeoniaobovata* Maxim., *Vacciniumvitis-idaea* L. and *Prunus×yedoensis* Matsum., accounting for 4.7% of the total EN species; seven taxa were VU species, including *Gymnocarpiumdryopteris* (L.) Newman, *Athyriumspinulosum* (Maxim.) Milde, *Aconitumcoreanum* (H. Lév.) Rapaics and *Goodyerarepens* (L.) R.Br., accounting for 7.3% of the total VU species; 14 taxa were Near Threatened (NT) species, including *Lycopodiumannotinum* L., *Cystopterisfragilis* (L.) Bernh., *Aconitumaustrokoreense* Koidz. and *Rosakoreana* Kom., accounting for 25% of the total NT species; 26 taxa were LC species, including AspleniumtrichomanesL.subsp.quadrivalens D.E.Mey., *Paeoniajaponica* (Makino) Miyabe & Takeda and *Tylophorafloribunda* Miq., accounting for 27% of the total LC species; one taxon, *Celtischoseniana* Nakai, was a DD species, accounting for 2.5% of the total DD species.

The detection frequency of the 67 taxa of rare plants and Red List species was n = 9 for two taxa (2.94%): *Paeoniajaponica* (Makino) Miyabe & Takeda and *Syringareticulata* (Blume) H.Hara, n = 5 for two taxa (2.94%): *Spiraeachartacea* Nakai and *Rodgersiapodophylla* A.Gray, n = 4 for two taxa (2.94%): *Aristolochiamanshuriensis* Kom. and *Arisaemaheterophyllum* Blume, n = 3 for six taxa (8.82%): *Rosakoreana* Kom., *Actaeabifida* (Nakai) J. Compton, *Aristolochiacontorta* Bunge, *Violaalbida* Palib., SyringavillosaVahlsubsp.wolfii (C.K. Schneid) Y. Chen & D.Y. Hong, and *Polypodiumsibiricum* Sipliv., n = 2 for 13 taxa (19.12%), including *Forsythiasaxatilis* (Nakai) Nakai and *Berchemiaberchemiifolia* (Makino) Koidz. and n = 1 for 42 taxa (63.24%), including *Goodyerarepens* (L.) R.Br., *Calanthediscolor* Lindl. and *Aconitumbarbatum* Patrin ex Pers.

### Endangered wildlife

Endangered wildlife refers to those animal species designated for protection by the Ministry of Environment, based on the Wildlife Protection and Management Act for effective conservation (Korea Ministry of Envrionment 2023). The Act states that all prohibited provisions and responsibilities regarding endangered species and violation of the Act could result in a fine up to 50 million KRW or a prison sentence up to 7 years. The Act does not simply stipulate the prohibited provisions and respomsibilities, but also states the regulations regarding the duty of the government to ensure the protection and survival of endangered species. This encompasses the conservation of wildlife habitats, development of protection measures on endangered species, relevant feild study and research, designation of an institution for protection outside habitats and promotion of projects on the restoration of endangered species. Endangered wildlife is at risk of extinction in the near future, based on the markedly reduced population or a very low number of entities due to natural or artificial threats. Thus, these species are legally protected and managed through designation by the law. Currently, 60 taxa of Class I EN species and 207 taxa of Class II EN species have been designated for independent management. The proportion of vascular plants amongst them is 11 taxa of Class I species and 77 taxa of Class II species.

The endangered wildlife species detected on the 25 algific talus slopes in South Korea included a total of five taxa (Suppl. material 4): *Aconitumaustrokoreense* Koidz., *Aconitumcoreanum* (H. Lév.) Rapaics, *Paeoniaobovata* Maxim., *Astilboidestabularis* (Hemsl.) Engl. and *Cyrtosiaseptentrionalis* (Rchb.f.) Garay, all of which correspond to Class II EN species (Table 4). The location in which each of these species was detected was as follows: Gwangjeom-dong, Hamyang-gun, Gyeongsangnam-do (A-E-2) for *Aconitumaustrokoreense* Koidz.; Unchi-ri, Jeongseon-gun, Gangwon-do (A-C-2) for *Aconitumcoreanum* (H. Lév.) Rapaics; Yeotan-ri, Jeongseon-gun, Gangwon-do (A-T-4) for *Paeoniaobovata* Maxim.; Jangyeol-ri, Jeongseon-gun, Gangwon-do (A-T-5) and Binggye-ri, Uiseong-gun, Gyeongsangbuk-do (A-C-4) for *Astilboidestabularis* (Hemsl.) Engl.; Seonheul-ri, Jeju-si, Jeju-do (A-V-1) for *Cyrtosiaseptentrionalis* (Rchb.f.) Garay.

### Endemic plants of the Korean Peninsula

**Figure 3.**
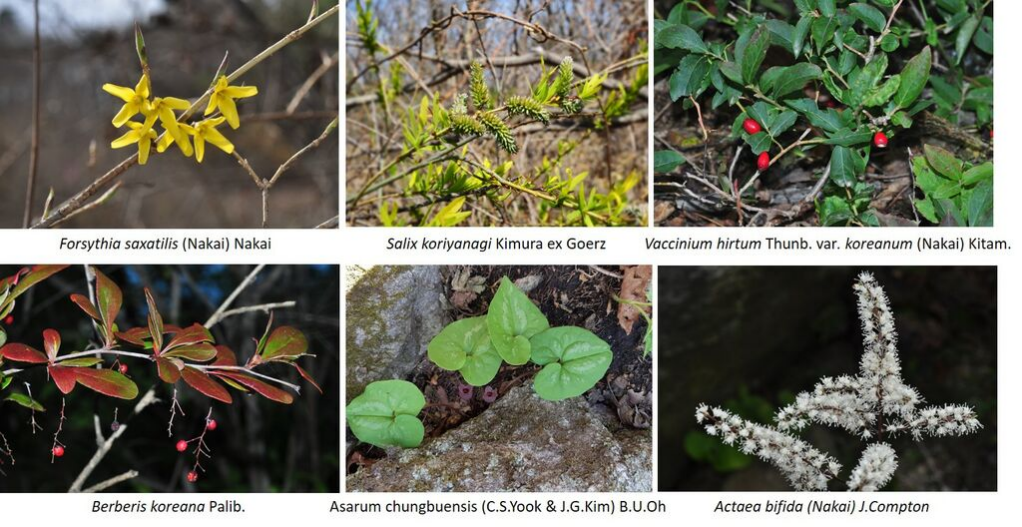
Pictures showing endemic plants of the Korean Peninsula on the algific talus slope in South Korea.

Among the vascular plants detected on the 25 algific talus slopes in South Korea, the endemic plants of the Korean Peninsula comprised 54 taxa (Suppl. material 5, Fig. 3), including *Populus×tomentiglandulosa* T.B. Lee, *Salixkoriyanagi* Kimura ex Goerz, *Aconitumaustrokoreense* Koidz., *Clematistrichotoma* Nakai, *Corydalismaculata* B.U.Oh & Y.S.Kim, *Micranthesoctopetala* (Nakai) Y.I.Kim & Y.D.Kim, *Sillaphytonpodagraria* (H.Boissieu) Pimenov, *Weigelasubsessilis* (Nakai) L.H.Bailey, *Heloniopsiskoreana* Fuse, N.S.Lee & M.N.Tamura and *Hemerocallishakuunensis* Nakai, accounting for 12.53% of the 431 taxa of all the endemic plants of the Korean Peninsula. In addition, the endemic species detected on the 25 algific talus slopes in South Korea comprised 44 taxa (Suppl. material 5), including *Aconitumpseudolaeve* Nakai, *Clematisbrachyura* Maxim. and ThalictrumactaeifoliumSiebold & Zucc.var.brevistylum Nakai, accounting for 11.70% of the 376 taxa of endemic species in total.

The detection frequency for the 58 taxa of endemic plants on the Korean Peninsula and endemic species across the 25 sites was the highest at n = 11 for *Weigelasubsessilis* (Nakai) L.H.Bailey., followed by n = 9 for two taxa: *Clematistrichotoma* Nakai and *Clematisurticifolia* Nakai ex Kitag.; n = 7 for two taxa: VacciniumhirtumThunb.var.koreanum (Nakai) Kitam. and *Hemerocallishakuunensis* Nakai; n = 6 for two taxa: Asarummandshuricum(Maxim.)M.Kim & S. Sovar.seoulense (Nakai) M. Kim & S. and *Lonicerasubsessilis* Rehder; n = 5 for three taxa: *Asperulalasiantha* Nakai, *Liliumamabile* Palib. and *Carexokamotoi* Ohwi; n = 4 for three taxa: ThalictrumactaeifoliumSiebold & Zucc.var.brevistylum Nakai, *Angelicareflexa* B.Y. Lee and RhododendronyedoenseMaxim.f.poukhanense (H. Lév.) Sugim. ex T. Yamaz.; n = 3 for eight taxa, including *Salixkoriyanagi* Kimura ex Goerz and *Aconitumpseudolaeve* Nakai; n = 2 for 14 taxa, including *Populus×tomentiglandulosa* T.B. Lee and *Broussonetia×hanjiana* M. Kim; n = 1 for 24 taxa, including *Pseudostellariacoreana* (Nakai) Ohwi and *Indigoferagrandiflora* B.H. Choi & S.K. Cho.

### Floristic target species

**Figure 4.**
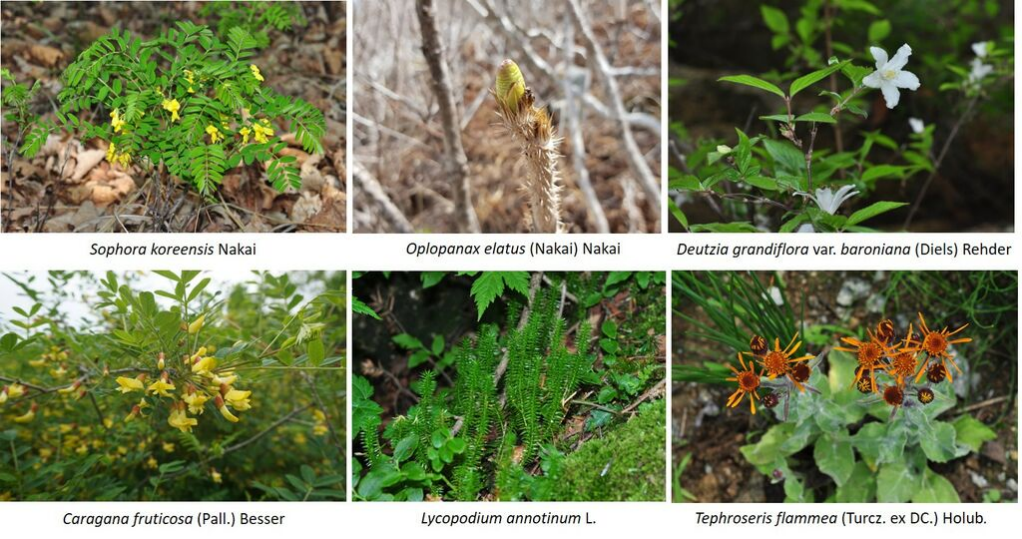
Pictures showing floristic target plants on the algific talus slope in South Korea.

The floristic target species detected on the 25 algific talus slopes in South Korea included a total of 317 taxa (Suppl. material 6, Fig. 4), accounting for 21.48% of the 1,476 taxa of all floristic target species. For Degree V, 12 taxa were detected, including *Thujakoraiensis* Nakai and *Tephroserisflammea* (Turcz. ex DC.) Holub, accounting for 4.65% of the 258 taxa of all Degree V species. For Degree IV, 48 taxa were detected, including *Microlepiastrigosa* (Thunb.) C.Presl, *Morusmongolica* (Bureau) C.K. Schneid. and *Caraganafruticosa* (Pall.) Besser, accounting for 10.90% of the 440 taxa of all Degree IV species. For Degree III, 103 taxa were detected, including *Lycopodiumobscurum* L., Acerukurunduense Trautv. & C.A. Mey. and *Hostaclausa* Nakai, accounting for 27.76% of the 371 taxa of all Degree III species. For Degree II, 58 taxa were detected, including *Huperziamiyoshiana* (Makino) Ching, *Pyrrosiapetiolosa* (Christ) Ching and *Hylotelephiumviviparum* (Maxim.) H. Ohba, accounting for 28.02% of the 207 taxa of all Degree II species. For Degree I, 96 taxa were detected, including Polystichumovato-paleaceum(Kodama)Sa. Kuratavar.coraiense (Christ) Sa. Kurata, *Clematispatens* C.Morren & Decne. and *Vacciniumoldhamii* Miq., accounting for 48% of the 200 taxa of all Degree I species.

The detection frequency for the 317 taxa of floristic target species according to the respective degree was as follows. For the 12 taxa of Degree V species, the detection frequency was n = 5 for *Asperulalasiantha* Nakai; n = 2 for two taxa: *Astilboidestabularis* (Hemsl.) Engl. and *Oplopanaxelatus* (Nakai) Nakai; n = 1 for nine taxa, including *Thujakoraiensis* Nakai and *Sophorakoreensis* Nakai. For the 48 taxa of Degree IV species, the detection frequency was n = 5 for *Ulmusmacrocarpa* Hance and *Rodgersiapodophylla* A. Gray; n = 4 for DeutziagrandifloraBungevar.baroniana (Diels) Rehder; n = 3 for seven taxa, including *Woodsiamacrochlaena* Mett. ex Kuhn and *Anemonereflexa* Steph. ex Willd.; n = 2 for 11 taxa, including *Laporteacuspidata* (Wedd.) Friis and *Berberiskoreana* Palib.; n = 1 for 27 taxa, including *Athyriumiseanum* Rosenst. and *Phedimusmiddendorffianus* (Maxim.) 't Hart. For the 103 taxa of Degree III species, the detection frequency was n = 11 for *Betulaschmidtii* Regel and *Philadelphusschrenkii* Rupr.; n = 10 for *Betuladavurica* Pall.; n = 9 for four taxa, including *Dryopterisfragrans* (L.) Schott and Sorbariasorbifolia(L.)A. Braunvar.stellipila Maxim.; n = 8 for *Actinidiakolomikta* (Maxim. & Rupr.) Maxim.; n = 7 for four taxa, including *Urticaangustifolia* Fisch. ex Hornem. and *Spiraeachamaedryfolia* L.; n = 6 for four taxa, including *Actaeaasiatica* H. Hara and *Ribesmandshuricum* (Maxim.) Kom.; n = 5 for two taxa: *Betulachinensis* Maxim. and *Celtiskoraiensis* Nakai; n = 4 for two taxa: *Acerpalmatum* Thunb. and *Brachybotrysparidiformis* Maxim. ex Oliv.; n = 3 for ten taxa, including *Selaginellatamariscina* (P. Beauv.) Spring and *Ulmuslaciniata* (Trautv.) Mayr; n = 2 for 18 taxa, including *Abiesnephrolepis* (Trautv. ex Maxim.) Maxim. and *Zabeliabiflora* (Turcz.) Makino; n = 1 for 55 taxa, including *Dracocephalumargunense* Fisch. ex Rchb. and *Lycopodiumobscurum* L. For the 58 taxa of Degree II species, the detection frequency was the highest at n = 16 for *Schisandrachinensis* (Turcz.) Baill., followed by n = 14 for *Tiliaamurensis* Rupr., n = 13 for *Magnoliasieboldii* K. Koch and *Weigelaflorida* (Bunge) A.DC., n = 9 for four taxa, including *Pinuskoraiensis* Siebold & Zucc. and *Tripterygiumregelii* Sprague & Takeda, n = 8 for *Euonymuspauciflorus* Maxim., n = 7 for *Euonymusmacropterus* Rupr., n = 6 for three taxa: *Potentilladickinsii* Franch. & Sav., *Tiliamandshurica* Rupr. & Maxim. and *Lonicerasubsessilis* Rehder, n = 5 for six taxa, including *Polystichumbraunii* (Spenn.) Fée and *Phellodendronamurense* Rupr., n = 4 for *Aristolochiamanshuriensis* Kom. and *Rubiachinensis* Regel & Maack, n = 3 for five taxa, including *Crepidomanesminutum* (Blume) K. Iwats. and *Spiraeasalicifolia* L., n = 2 for 16 taxa, including *Osmundacinnamomea* L. and *Alnusjaponica* (Thunb.) Steud. and n = 1 for 16 taxa, including *Coniogrammeintermedia* Hieron. and ViolatokubuchianaMakinovar.takedana (Makino) F. Maek. For the 96 taxa of Degree I species, the detection frequency was the highest across the 25 sites at n = 19 for UlmusdavidianaPlanch. ex DC.var.japonica (Rehder) Nakai; followed by n = 16 for *Deutziaparviflora* Bunge; n = 12 for *Deutziaglabrata* Kom.; n = 9 for *Deutziauniflora* Shirai and SyringapubescensTurcz.subsp.patula (Palib.) M.C. Chang & X.L. Chen; n = 8 for four taxa, including *Juglansmandshurica* Maxim. and *Linderaerythrocarpa* Makino; n = 7 for three taxa, including *Pileajaponica* (Maxim.) Hand.-Mazz., *Clematispatens* C.Morren & Decne. and *Spiraeablumei* G.Don; n = 6 for five taxa, including *Hepaticaasiatica* Nakai and *Impatiensnoli-tangere* L.; n = 5 for four taxa, including *Ilexmacropoda* Miq. and *Carexokamotoi* Ohwi; and n = 4 for nine taxa, including *Hemipteleadavidii* (Hance) Planch. and *Linderaglauca* (Siebold & Zucc.) Blume.

### Northern lineage plants on the Korean Peninsula and 300 species threatened by climate change

**Figure 5.**
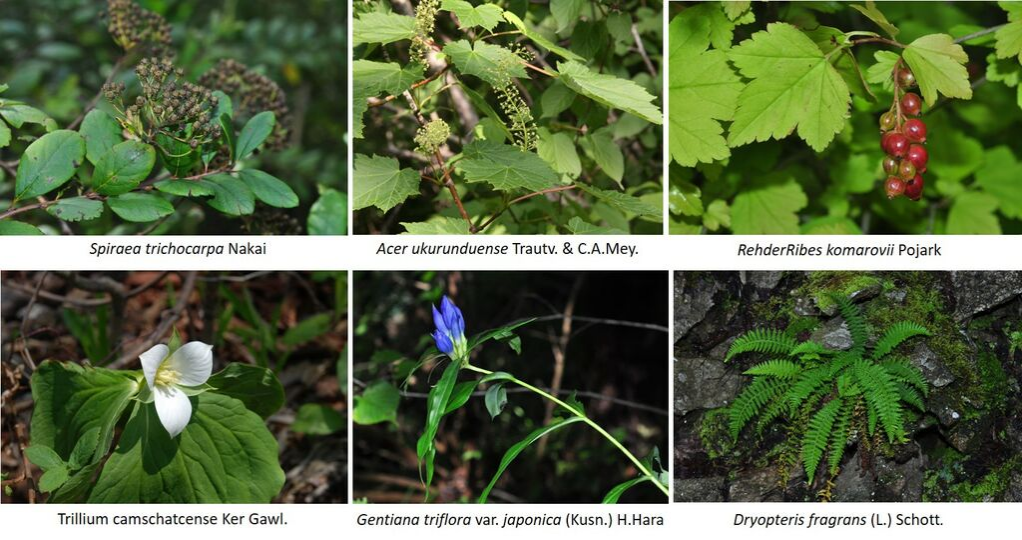
Pictures showing northern lineage plants on the algific talus slope in South Korea.

From the 1,342 taxa of shared species between South Korea and Russia, Gantsetseg et al. (2020) compiled a list of northern lineage plants on the Korean Peninsula that includes 615 taxa after excluding 727 taxa that were distributed worldwide, inhabit marine ecosystems, are distributed mainly in temperate regions of East Asia or are cultivated or invasive alien plants. The northern lineage plants detected on the 25 algific talus slopes in South Korea included a total of 181 taxa (Suppl. material 7, Fig. 5), which accounted for 29.43% of all northern lineage plants. The 181 taxa included *Gymnocarpiumdryopteris* (L.) Newman, *Dryopterisexpansa* (C. Presl) Fraser-Jenk. & Jermy and *Eranthisstellata* Maxim. In addition, among the 300 species of plants adaptable to climate change as reported in Korea National Arboretum (2010), 100 species of northern lineage plants comprised 39 taxa, including *Clematisfusca* Turcz., *Aristolochiamanshuriensis* Kom. and *Spiraeatrichocarpa* Nakai, whereas southern lineage plants comprised 21 taxa, including *Ficuserecta* Thunb. and *Toxicodendronsylvestre* (Siebold & Zucc.) Kuntze.

The detection frequency for the 218 taxa of northern lineage plants on the Korean Peninsula and of northern and southern lineage plants among the 300 plants adaptable to climate change on the Korean Peninsula was the highest across the 25 sites at n = 21 for *Rhododendronmucronulatum* Turcz., followed by n = 20 for *Philadelphustenuifolius* Rupr. & Maxim.; n = 19 for *Violaacuminata* Ledeb.; n = 18 for *Dryopteriscrassirhizoma* Nakai; n = 17 for *Rubuscrataegifolius* Bunge; n = 16 for five taxa, including *Sambucuswilliamsii* Hance and *Deutziaparviflora* Bunge; n = 15 for two taxa, including *Corydalisspeciosa* Maxim. and *Pinusdensiflora* Siebold & Zucc.; n = 14 for *Tiliaamurensis* Rupr.; n = 13 for *Melampyrumroseum* Maxim., *Maackiaamurensis* Rupr.; and *Vitisamurensis* Rupr.; n = 12 for *Pyrolajaponica* Klenze ex Alef. and *Deutziaglabrata* Kom.; n = 11 for four taxa, including *Betulaschmidtii* Regel and *Disporumsmilacinum* A. Gray; and n = 10 for *Aspleniumruprechtii* Sa. Kurata and *Betuladavurica* Pall. Meanwhile, 73 taxa, including *Asarummandshuricum* (Maxim.) M. Kim & S. So were detected once and 41 taxa, including *Ribesmaximowiczianum* Kom. were detected twice.

### Limestone area plants

**Figure 6.**
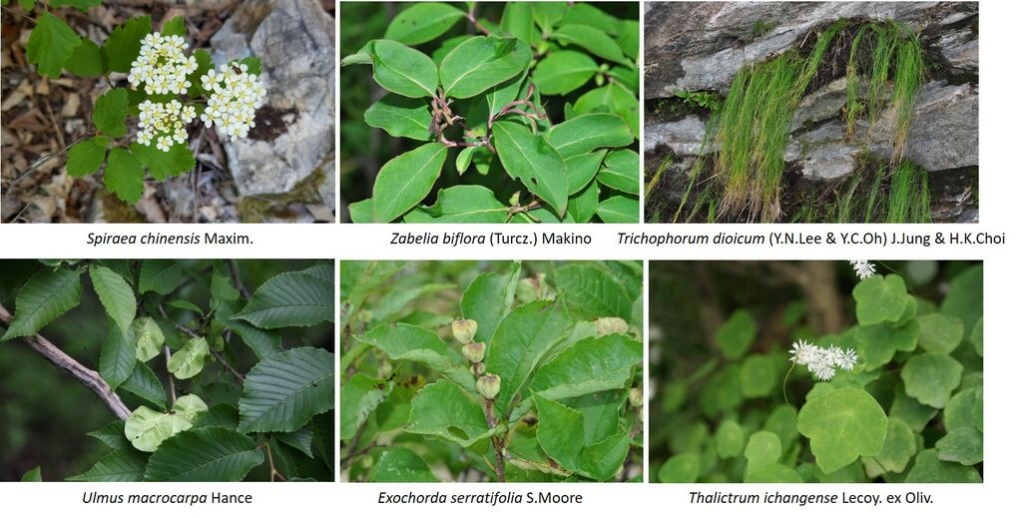
Pictures showing calciphilous plants on the algific talus slope in South Korea.

According to Kim et al. (2021), the floristic inventory for vascular plants in limestone areas on the Korean Peninsula included 1,290 taxa and the distribution characteristics were analyzed for 102 taxa (Suppl. material 8, Fig. 6) of limestone area plants to categorize the Calciphilous Indicator Plant (CIP; 14 taxa), Superative Calciphilous Plant (SCP; 30 taxa) and Comparative Calciphilous plant (CCP; 58 taxa). The limestone area plants detected on the 25 algific talus slopes in South Korea included a total of 32 taxa, among which three taxa: *Prunuschoreiana* Nakai ex H.T.Im, *Zabeliatyaihyonii* (Nakai) Hisauti & H.Hara and *Trichophorumpolygamum* D.C.Son & K.S.Chang were CIP; six taxa, including *Gymnocarpiumjessoense* (Koidz.) Koidz., *Morusmongolica* (Bureau) C.K. Schneid., AstragaluspenduliflorusLam.var.dahuricus (DC.) X.Y. Zhu, *Caraganafruticosa* (Pall.) Besser and *Carexussuriensis* Kom. were SCP; 23 taxa, including *Aspleniumtenuicaule* Hayata, *Polystichumcraspedosorum* (Maxim.) Diels and *Saussureaodontolepis* (Herder) Sch.Bip. ex Maxim. were CCP.

The detection frequency for the 32 taxa of limestone area plants was n = 6 for *Spiraeachinensis* Maxim.; n = 5 for *Ulmusmacrocarpa* Hance and *Celtiskoraiensis* Nakai; n = 3 for *Actaeabifida* (Nakai) J. Compton and Buxussinica(Rehder & E.H. Wilson)M.Chengvar.insularis (Nakai) M. Cheng; n = 2 for 13 taxa, including *Spiraeatrichocarpa* Nakai, *Lithospermumerythrorhizon* Siebold & Zucc. and *Zabeliabiflora* (Turcz.) Makino; n = 1 for 14 taxa, including *Exochordaserratifolia* S. Moore and *Astermaackii* Regel.

### Alien plants, invasive alien plants and introduced disturbing plants

Alien plants that were intentionally or unintentionally, such as airports, introduced into South Korea and have grown wild (Korea National Arboretum 2019a). Seventy-five taxa (Suppl. material 9) of alien plants, including *Rumexcrispus* L., *Amorphafruticosa* L. and *Bidensfrondosa* L., were detected on the 25 algific talus slopes in South Korea. Categorization of the alien plants by life form showed 10 taxa of woody plants, including *Ailanthusaltissima* (Mill.) Swingle and *Robiniapseudoacacia* L., 26 taxa of annual plants, including *Chenopodiumalbum* L., *Anthriscuscaucalis* M. Bieb. and *Tagetesminuta* L., 12 taxa of biennial plants, including *Lepidiumapetalum* Willd. and *Carduuscrispus* L. and 27 taxa of perennial plants, including *Barbareavulgaris* W.T.Aiton, *Trifoliumrepens* L., *Oxaliscorniculata* L. and *Erigeronphiladelphicus* L.

**Figure 7.**
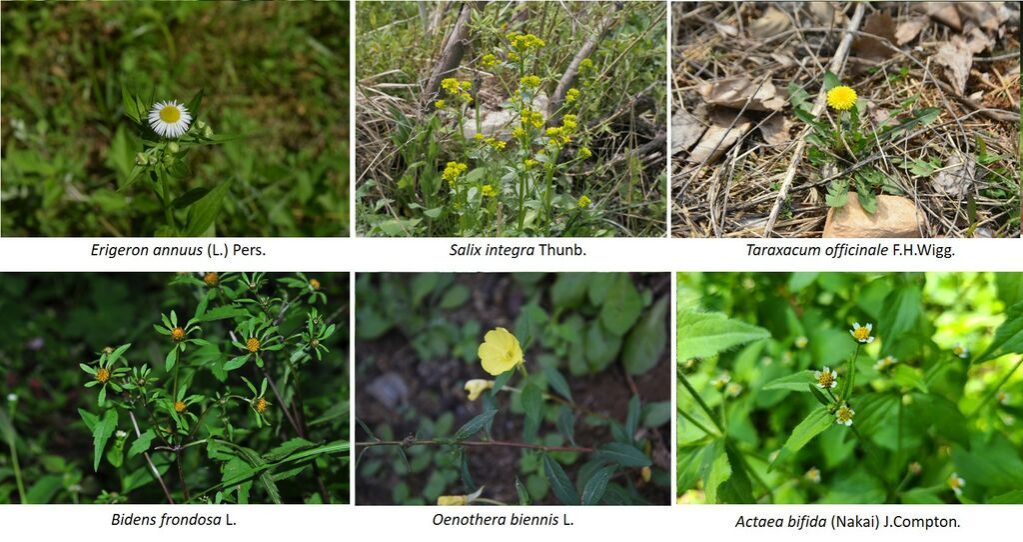
Pictures showing invasive alien plants on the algific talus slope in South Korea.

**Figure 8.**
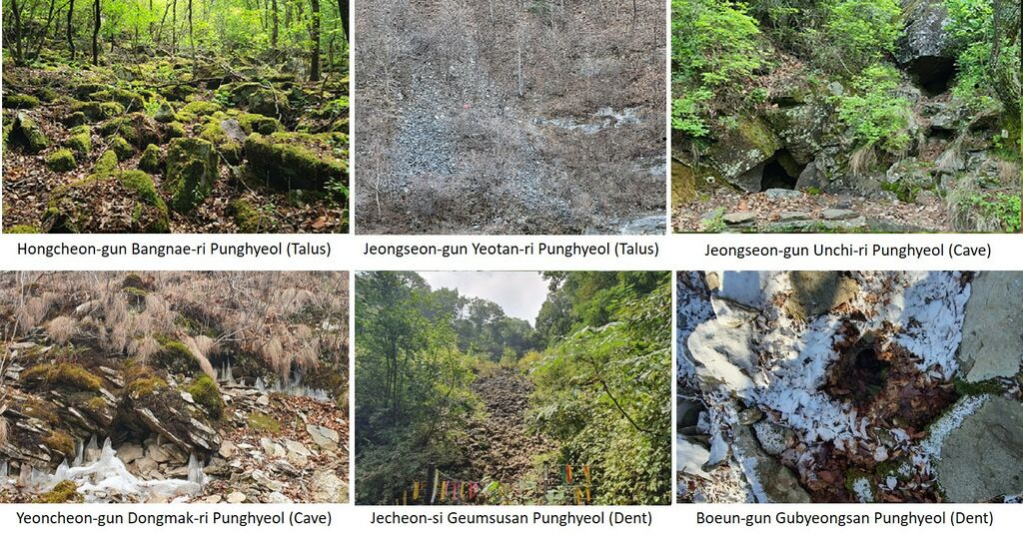
3 major types (Talus, Cave and Dent) of algific talus slopes in South Korea.

Categorization of the alien plants by native habitat indicated one taxon, *Trifoliumrepens* L., from Africa; 10 taxa, including *Rumexcrispus* L. and *Rumexobtusifolius* L. from Europe, Africa and Asia; three taxa: *Cerastiumglomeratum* Thuill., *Anthriscuscaucalis* M.Bieb. and *Solanumnigrum* L., from Europe and Africa; four taxa, including AmaranthusblitumL.subsp.oleraceus (L.) Costea and *Medicagosativa* L. from Europe; 14 taxa, including *Rumexacetosella* L. and *Dactylisglomerata* L. from Europe and Asia; 13 taxa, including *Ailanthusaltissima* (Mill.) Swingle and *Zingibermioga* (Thunb.) Roscoe from Asia; 17 taxa, including *Amaranthusretroflexus* L. and *Ambrosiaartemisiifolia* L. from North America; two taxa: *Ipomoeanil* (L.) Roth and *Tagetesminuta* L., from South America; nine taxa, including *Phytolaccaamericana* L. and *Ecliptathermalis* Bunge from America; one taxon, *Poapratensis* L., from North America, Europe and Asia; and one taxon, *Sicyosangulatus* L. Magnoliophyta, from Noth America and Oceania (Suppl. material 9, Fig. 7).

The alien plants were categorized into archaeophytes, potentially invasive plants and invasive alien plants according to time of invasion and settlement, whereas potentially invasive plants were divided into concerned alien plants and uncertain plants. Invasive alien plants were divided into casual alien plants and naturalized plants (Korea National Arboretum 2019a). Eleven taxa of archaeophytes were considered potentially invasive plants, including *Thlaspiarvense* L., *Oxaliscorniculata* L. and AmaranthusblitumL.subsp.oleraceus (L.) Costea; concerned alien plants were six taxa, including *Pinusrigida* Mill. and *Styphnolobiumjaponicum* (L.) Schott; uncertain plants were four taxa, including *Lepidiumapetalum* Willd. and *Scutellariabaicalensis* Georgi. For invasive alien plants, one taxon, *Veronicaanagallis-aquatica* L., was a casual alien plant and 53 taxa, including *Euphorbiamaculata* L., *Veronicapersica* Poir. and *Conyzacanadensis* (L.) Cronquist, were naturalized plants.

According to Kang et al. (2020), distribution data on invasive alien plants were used to categorisze the distribution into five levels: widespread (WS) species, serious spread (SS) species, concerned spread (CS) species, minor spread (MS) species and potential spread (PS) species. Among the 53 taxa of invasive alien plants detected at 25 algific talus slopes in South Korea, the WS species were 19 taxa, including *Chenopodiumficifolium* Sm., *Ambrosiaartemisiifolia* L. and *Erechtiteshieraciifolius* (L.) Raf. ex DC.; SS species were nine taxa, including *Trifoliumpratense* L. and *Seneciovulgaris* L.; CS species were seven taxa, including *Euphorbiahypericifolia* L. and *Solidagogigantea* Aiton; MS species were 11 taxa, including *Erigeronstrigosus* Muhl. ex Willd. and *Vulpiamyuros* (L.) C.C. Gmel.; PS species were eight taxa, including *Violasororia* Willd. and *Lamiumpurpureum* L.

In this study, the NI, calculated as = (number of alien plants / total number of detected plants) × 100, was 7.13% and the UI, calculated as = (number of alien plants detected in the study site / number of alien plants inhabiting the Korean Peninsula) × 100, was 12.12%. According to Kim et al. (2000), an NI ≥ 4% for a forest indicates disturbance. Thus, the 25 sites of algific talus slopes in South Korea investigated in this study appear to be at an initial stage of disturbance. Meanwhile, preventive measures on potential spread should be implemented due to the high accessibility of sites which are in close proximity to roads.

The detection frequency for the 75 taxa of alien plants was highest at n = 13 for *Erigeronannuus* (L.) Pers., thereby exhibiting the widest level of distribution. The detection frequency was n = 11 for *Oenotherabiennis* L.; n = 9 for *Taraxacumofficinale* F.H.Wigg.; n = 7 for four taxa, including *Bidensfrondosa* L. and *Galinsogaquadriradiata* Ruiz & Pav.; n = 6 for four taxa, including *Robiniapseudoacacia* L. and *Ambrosiaartemisiifolia* L.; n = 5 for four taxa, including *Barbareavulgaris* W.T.Aiton and *Conyzacanadensis* (L.) Cronquist; n = 4 for five taxa, including *Symphyotrichumpilosum* (Willd.) G.L. Nesom and *Chenopodiumficifolium* Sm.; n = 3 for 10 taxa, including *Seneciovulgaris* L. and *Euphorbiahypericifolia* L.; n = 2 for 16 taxa, including *Festucaarundinacea* Schreb. and *Amaranthuspatulus* Bertol.; n = 1 for 29 taxa, including *Quamoclitcoccinea* (L.) Moench and *Ageratinaaltissima* (L.) R.M. King & H. Rob.

The introduced disturbing plants detected on the 25 algific talus slopes in South Korea were eight taxa: *Humulusscandens* (Lour.) Merr., *Rumexacetosella* L., *Sicyosangulatus* L., *Ageratinaaltissima* (L.) R.M. King & H. Rob., *Ambrosiaartemisiifolia* L., *Ambrosiatrifida* L., *Solidagoaltissima* L. and *Symphyotrichumpilosum* (Willd.) G.L. Nesom. The detection frequency was n = 9 for *Humulusscandens* (Lour.) Merr., followed by n = 6 for *Ambrosiaartemisiifolia* L., n = 4 for *Symphyotrichumpilosum* (Willd.) G.L. Nesom, n = 3 for *Ambrosiatrifida* L., n = 2 for *Rumexacetosella* L. and n = 1 for *Sicyosangulatus* L., *Ageratinaaltissima* (L.) R.M. King & H.Rob. and *Solidagoaltissima* L.

### Flora of the algific talus slope by type

The flora by type across the algific talus slope sites in South Korea was as follows (Suppl. material 10): 804 taxa of 114 families, 394 genera, 722 species, 18 subsp., 59 var. and 5 f. for 14 sites of type talus; 701 taxa of 107 families, 361 genera, 623 species, 18 subsp., 53 var. and 7 f. for four sites of type cave; 306 taxa of 82 families, 173 genera, 274 species, 5 subsp., 25 var. and 2 f. for four sites of type dent; 80 taxa of 44 families, 68 genera, 77 species, 1 subsp. and 2 var. for one site of type vertical cave; 173 taxa of 72 families, 129 genera, 152 species, 5 subsp., 14 var. and 2 f. for two sites of type others. Each type accounted for 76.43%, 66.63%, 29.09%, 7.60% and 16.44%, respectively, of the 1,052 taxa of detected plants in total.

Notable plants were analyzed for each type of algific talus slope. First, analysis was based on the class of rare plants above or equal to VU species, as designated by the Korea Forest Service. The six taxa of CR species included three taxa, *Paeoniaobovata* Maxim., *Vacciniumvitis-idaea* L. and *Goodyerarepens* (L.) R.Br., for the type talus; three taxa, *Cystopterisfragilis* (L.) Bernh., AspleniumtrichomanesL.subsp.quadrivalens D.E. Mey. and *Aconitumcoreanum* (H. Lév.) Rapaics, for the type cave; one taxon, *Cyrtosiaseptentrionalis* (Rchb.f.) Garay, for the type vertical cave; and none for the types dent and others. The 11 taxa of EN species included nine taxa, *Astilboidestabularis* (Hemsl.) Engl., *Sophorakoreensis* Nakai etc., for the type talus; four taxa, *Rosakoreana* Kom., *Dracocephalumargunense* Fisch. ex Link etc., for the type cave; three taxa, *Micranthesoctopetala* (Nakai) Y.I. Kim & Y.D. Kim, *Deutziapaniculata* Nakai and *Oplopanaxelatus* (Nakai) Nakai, for the type dent; and none for the types vertical cave and others. The 15 taxa of VU species included five taxa, *Adiantumpedatum* L., *Paeoniajaponica* (Makino) Miyabe & Takeda etc., for the type talus; seven taxa, *Athyriumspinulosum* (Maxim.) Milde, *Scorzoneraalbicaulis* Bunge etc., for the type cave; five taxa, *Piceajezoensis* (Siebold & Zucc.) Carrière, *Paeoniajaponica* (Makino) Miyabe & Takeda etc., for the type dent; two taxa, *Illiciumanisatum* L. and *Calanthediscolor* Lindl., for the type vertical cave; and one taxon, *Aconitumaustrokoreense* Koidz., for the type others. Regarding the NT species of Red list species of vascular plants, the four taxa of EN species included two taxa, *Paeoniaobovata* Maxim. and *Vacciniumvitis-idaea* L., for the type talus; one taxon, *Prunus×yedoensis* Matsum., for the type cave; and one taxon, *Piceajezoensis* (Siebold & Zucc.) Carrière, for the type dent. The eight taxa of VU species included six taxa, *Gymnocarpiumdryopteris* (L.) Newman, *Zabeliatyaihyonii* (Nakai) Hisauti & H. Hara, for the type talus; three taxa, *Athyriumspinulosum* (Maxim.) Milde, *Aconitumcoreanum* (H. Lév.) Rapaics and *Spiraeachartacea* Nakai, for the type cave; and three taxa, *Gymnocarpiumdryopteris* (L.) Newman, *Spiraeachartacea* Nakai and *Oplopanaxelatus* (Nakai) Nakai, for the type dent. Further, the 14 taxa of NT species included eight taxa, *Aconitumbarbatum* Patrin ex Pers., *Prunuschoreiana* Nakai ex H.T. Im etc., for the type talus; four taxa, *Cystopterisfragilis* (L.) Bernh., *Rosakoreana* Kom. etc., for the type cave; three taxa, *Lycopodiumannotinum* L., *Thujakoraiensis* Nakai and *Deutziapaniculata* Nakai, for the type dent; two taxa, *Cyrtosiaseptentrionalis* (Rchb.f.) Garay and *Goodyerahenryi* Rolfe, for the type vertical cave; and one taxon, *Aconitumaustrokoreense* Koidz., for the type others.

Analysis of the endemic plants on the Korean Peninsula and the endemic species across the 25 algific talus slopes by type indicated that the type talus had 45 taxa, including *Aconitumpseudolaeve* Nakai and *Angelicareflexa* B.Y. Lee; the type cave had 26 taxa, including *Clematistrichotoma* Nakai and *Asarumchungbuensis* (C.S. Yook & J.G. Kim) B.U.Oh; the type dent had 21 taxa, including Asarummandshuricum(Maxim.)M.Kim & S. Sovar.seoulense (Nakai) M. Kim & S. So and *Stewartiakoreana* Nakai ex Rehder; the type vertical cave had one taxon, *Sasaquelpaertensis* Nakai; the type others had six taxa, including *Hostaminor* (Baker) Nakai and ThalictrumactaeifoliumSiebold & Zucc.var.brevistylum Nakai. In addition, when the analysis was based on the degree of floristic target species above or equal to III, the 163 taxa included 98 taxa, *Actinidiakolomikta* (Maxim. & Rupr.) Maxim., *Cephalantherafalcata* (Thunb.) Blume etc., for the type talus; 67 taxa, *Woodsiamacrochlaena* Mett. ex Kuhn, *Catolobuspendulus* (L.) Al-Shehbaz etc., for the type cave; 44 taxa, *Lycopodiumobscurum* L., *Aruncusdioicus* (Walter) Fernald etc., for the type dent; 24 taxa, *Coniogrammejaponica* (Thunb.) Diels, *Actinodaphnelancifolia* (Blume) Meisn. etc., for the type vertical cave; and eight taxa, *Lepisorusonoei* (Franch. & Sav.) Ching, *Vacciniumbracteatum* Thunb. etc., for the type others.

Analysis of the alien plants and invasive alien plants detected on the 25 algific talus slopes by type indicated that, for the 75 taxa of alien plants, the type talus had 48 taxa, including *Phytolaccaamericana* L. and *Erigeronannuus* (L.) Pers.; the type cave had 62 taxa, including *Medicagosativa* L. and *Euphorbiahypericifolia* L.; the type dent had four taxa, including *Chenopodiumficifolium* Sm.; the type vertical cave had one taxon, *Zingibermioga* (Thunb.) Roscoe; and the type others had no alien plant. Next, for the 54 taxa of invasive alien plants, the type talus had 34 taxa, type cave had 45 taxa, type dent had four taxa and type vertical cave had one taxon. Categorization based on the level of distribution revealed that the 35 taxa at the type talus included 17 taxa, *Rumexacetosella* L. etc., for the WS species; seven taxa, *Euphorbiamaculata* L. etc., for the SS species; four taxa, *Amaranthuspatulus* Bertol. etc. for the CS species; six taxa, *Melilotusalbus* Medik. etc., for the MS species; one taxon, *Erigeronphiladelphicus* L., for the PS species. The 45 taxa at the type cave included 18 taxa, *Rumexcrispus* L. etc., for the WS species; seven taxa, *Trifoliumpratense* L. etc., for the SS species; five taxa, *Chenopodiumalbum* L. etc., for the CS species; nine taxa, *Sicyosangulatus* L. etc., for the MS species; and six taxa, *Anthriscuscaucalis* M.Bieb., etc., for the PS species. The four taxa at the type dent included one taxon, *Chenopodiumficifolium* Sm., for the WS species; none for the SS and CS species; one taxon, *Barbareavulgaris* W.T. Aiton, for the MS species; two taxa, *Anthriscuscaucalis* M. Bieb. and *Ageratinaaltissima* (L.) R.M. King & H. Rob., for the PS species. The one taxon at the type vertical cave was *Zingibermioga* (Thunb.) Roscoe, which is a PS species.

### Floral distribution on the algific talus slopes by region

Across the 25 algific talus slopes in South Korea, the flora by region was as follows (Suppl. material 11): 431 taxa of 87 families, 256 genera, 377 species, 13 subsp., 36 var. and 5 f. for Unchi-ri, Jeongseon-gun (A-C-2); 361 taxa of 81 families, 232 genera, 326 species, 9 subsp., 24 var. and 2 f. for Binggye-ri, Uiseong-gun (A-C-4); 331 taxa of 84 families, 203 genera, 295 species, 6 subsp., 28 var. and 2 f. for Yeotan-ri, Jeongseon-gun (A-T-4); 298 taxa of 76 families, 176 genera, 267 species, 5 subsp., 23 var. and 3 f. for Bangnae-ri, Hongcheon-gun (A-T-2) and 298 taxa of 80 families, 207 genera, 260 species, 12 subsp., 24 var. and 2 f. for Naeryong-ri, Cheongsong-gun (A-T-13); and 281 taxa of 86 families, 204 genera, 241 species, 11 subsp., 26 var. and 3 f. for Boopyeong-ri, Inje-gun (A-T-7).

Notable plants were analyzed for each region. On the algific talus slope in Seongdong-ri, Pocheon-si (A-T-1), one taxon, *Gastrodiaelata* Blume, was a VU species of rare plants designated by the Korea Forest Service and an LC species of the Red list. Three taxa, *Clematisbrachyura* Maxim., *Asarummisandrum* B.U. Oh & J.G. Kim and *Indigoferagrandiflora* B.H. Choi & S.K. Cho, were endemic plants of the Korean peninsula. Five taxa, including *Ulmusmacrocarpa* Hance and Sorbariasorbifolia(L.)A. Braunvar.stellipila Maxim. were floristic target species of degree III or above. Seven taxa were invasive alien plants, out of which four were WS species, including *Ambrosiaartemisiifolia* L.; one was an SS species, *Symphyotrichumpilosum* (Willd.) G.L. Nesom, two were MS species, *Cerastiumglomeratum* Thuill. and *Ambrosiatrifida* L. and there were no CD or PS species. On the algific talus slope in Bangnae-ri, Hongcheon-gun (A-T-2), one taxon, *Vacciniumvitis-idaea* L., was a CR species of rare plants designated by the Korea Forest Service, two taxa, *Prunuschoreiana* Nakai ex H.T. Im and *Rosakoreana* Kom., were EN species and two taxa, *Actaeabifida* (Nakai) J.Compton and *Paeoniajaponica* (Makino) Miyabe & Takeda, were VU species. One taxon, *Vacciniumvitis-idaea* L., was an EN species of the Red list and three taxa, including *Prunuschoreiana* Nakai ex H.T. Im, were NT species. Sixteen taxa, including VacciniumhirtumThunb.var.koreanum (Nakai) Kitam., were endemic plants of the Korean Peninsula or endemic species. Thirty taxa, including *Ulmuslaciniata* (Trautv.) Mayr, were floristic target species of degree III or above. Eleven taxa, including *Solanumnigrum* L., were alien plants and six taxa were invasive alien plants, out of which four were WS species, including *Bidensfrondosa* L., one was a CS species, *Amaranthuspatulus* Bertol. and one was a PS species, *Erigeronphiladelphicus* L. On the algific talus slope in Shinghi-ri, Pyeongchang-gun (A-T-3), two taxa, *Rosakoreana* Kom. and *Oplopanaxelatus* (Nakai) Nakai, were EN species of rare plants designated by the Korea Forest Service and one taxon, *Adiantumpedatum* L., was a VU species. One taxon, *Oplopanaxelatus* (Nakai) Nakai, was a VU species of the Red list and two taxa, *Rosakoreana* Kom. and *Paeonialactiflora* Pall., were NT species. Nine taxa, including *Viciachosenensis* Ohwi were endemic plants of the Korean Peninsula. Thirty-six taxa, including *Actaeaasiatica* H. Hara were floristic target species of degree III or above. Six taxa, including *Oxaliscorniculata* L. were alien plants and four taxa were invasive alien plants, out of which three were WS species, including *Taraxacumofficinale* F.H. Wigg. and one was an SS species: *Carduuscrispus* L.

On the algific talus slope in Yeotan-ri, Jeongseon-gun (A-T-4), one taxon, *Paeoniaobovata* Maxim., was a CR species of rare plants designated by the Korea Forest Service, two taxa, *Prunuschoreiana* Nakai ex H.T.Im and *Forsythiasaxatilis* (Nakai) Nakai, were EN species and two taxa, *Actaeabifida* (Nakai) J.Compton and *Paeoniajaponica* (Makino) Miyabe & Takeda, were VU species. One taxon, *Paeoniaobovata* Maxim., was an EN species of the Red list, three taxa, including *Gymnocarpiumdryopteris* (L.) Newman were VU species and three taxa, including *Aconitumbarbatum* Patrin ex Pers. were NT species. Twenty-one taxa, including *Liliumamabile* Palib. were endemic plants of the Korean Peninsula. Thirty-eight taxa, including Zabeliabiflora (Turcz.) Makino, were floristic target species of degree III or above. Nineteen taxa, including *Galiumtricornutum* Dandy were alien plants. Thirteen taxa were invasive alien plants, out of which eight were WS species, including *Trifoliumrepens* L.; two were SS species, including *Symphyotrichumpilosum* (Willd.) G.L. Nesom and *Carduuscrispus* L., two were MS species, including *Barbareavulgaris* W.T. Aiton and *Melilotusalbus* Medik. and one was a PS species, *Erigeronphiladelphicus* L. (Lee et al. 2022b). On the algific talus slope in Jangyeol-ri, Jeongseon-gun (A-T-5), two taxa, *Astilboidestabularis* (Hemsl.) Engl. and *Zabeliatyaihyonii* (Nakai) Hisauti & H. Hara, were EN species of rare plants designated by the Korea Forest Service. Two taxa, *Spiraeachartacea* Nakai and *Zabeliatyaihyonii* (Nakai) Hisauti & H. Hara, were VU species of the Red list and one taxon, *Astilboidestabularis* (Hemsl.) Engl., was an NT species. Four taxa, including *Weigelasubsessilis* (Nakai) L.H. Bailey were endemic plants on the Korean Peninsula. Fifteen taxa, including *Caraganafruticosa* (Pall.) Besser were floristic target species of degree III or above. Eleven taxa, including *Conyzacanadensis* (L.) Cronquist were alien plants and six taxa were invasive alien plants, out of which five were WS species, including *Galinsogaquadriradiata* Ruiz & Pav. and *Trifoliumpratense* L. was an SS species. On the algific talus slope in Bongoh-ri, Hwacheon-gun (A-T-6), one taxon, *Goodyerarepens* (L.) R.Br., was a CR species of rare plants designated by the Korea Forest Service and a VU species of the Red list. One taxon, *Clematistrichotoma* Nakai, was an endemic plant on the Korean Peninsula. Seven taxa, including Sorbariasorbifolia(L.) A. Braunvar.stellipila Maxim. were floristic target species of degree III or above. Invasive alien plants included one taxon, *Ambrosiaartemisiifolia* L., a WS species and one taxon, *Symphyotrichumpilosum* (Willd.) G.L. Nesom, an SS species. On the algific talus slope in Boopyeong-ri, Inje-gun (A-T-7), one taxon, *Sophorakoreensis* Nakai, was an EN species of rare plants designated by the Korea Forest Service and one taxon, *Paeoniajaponica* (Makino) Miyabe & Takeda, as a VU species. One taxon, *Sophorakoreensis* Nakai, was an NT species of the Red list. Five taxa, including *Pseudostellariacoreana* (Nakai) Ohwi were endemic plants of the Korean Peninsula. Fifteen taxa, including DeutziagrandifloraBungevar.baroniana (Diels) Rehder were floristic target species of degree III or above. Nineteen taxa, including *Seneciovulgaris* L. were alien plants and fourteen taxa were invasive alien plants, out of which ten were WS species, including *Dactylisglomerata* L., three were SS species, including *Sonchusoleraceus* L. and one was an MS species, *Ambrosiatrifida* L.

On the algific talus slope in Gaeahn-ri, Boeun-gun (A-T-8), there was no rare plants designated by the Korea Forest Service or Red list species. Two taxa, ThalictrumactaeifoliumSiebold & Zucc.var.brevistylum Nakai and *Lonicerasubsessilis* Rehder, were endemic plants of the Korean Peninsula. One taxon, *Woodsiamacrochlaena* Mett. ex Kuhn, was a floristic target species of degree III or above. Four taxa, including *Erechtiteshieraciifolius* (L.) Raf. ex DC. were alien plants and three taxa were invasive alien plants, all of which were WS species, including *Erigeronannuus* (L.) Pers. On the algific talus slope in Oejungbang-ri, Danyang-gun (A-T-9), there was no rare plant designated by the Korea Forest Service or Red list species as an endemic plant of the Korean Peninsula. One taxon, *Celtiskoraiensis* Nakai, was a floristic target species of degree III or above. Four taxa, including *Morusalba* L. were alien plants and two taxa: *Oenotherabiennis* L. and *Erigeronannuus* (L.) Pers., were invasive alien plants, both of which were WS species. On the algific talus slope in Shinjeong-ri, Jeongeup-si (A-T-10), one taxon, *Paeoniajaponica* (Makino) Miyabe & Takeda, was designated as a rare plant by the Korea Forest Service and a VU species of the Red list. One taxon, *Weigelasubsessilis* (Nakai) L.H. Bailey, was an endemic plant of the Korean peninsula. Two taxa, *Cephalantherafalcata* (Thunb.) Blume and *Rhamnusdavurica* Pall., were floristic target species of degree III or above; no alien plant was detected. On the algific talus slope in Beobhwa-ri, Yeongcheon-ri (A-T-11), one taxon, *Paeoniajaponica* (Makino) Miyabe & Takeda, was a VU species of rare plants designated by the Korea Forest Service and an LC species of the Red list. One taxon, *Carexfusanensis* Ohwi, was an endemic plant of the Korean Peninsula. Eight taxa, including *Spiraeachamaedryfolia* L. and *Syringareticulata* (Blume) H. Hara were floristic target species of degree III or above; no alien plant was detected.

On the algific talus slope in Hwabuk-ri, Gunwi-gun (A-T-12), one taxon, *Berchemiaberchemiifolia* (Makino) Koidz., was a VU species of rare plants designated by the Korea Forest Service and an LC species of the Red list. No endemic plant was detected, whereas six taxa, including *Rhamnusussuriensis* J.J. Vassil. and *Celtiskoraiensis* Nakai were floristic target species of degree III or above. Two taxa, *Erigeronannuus* (L.) Pers. and *Taraxacumofficinale* F.H. Wigg., were invasive alien plants, both of which were WS species. On the algific talus slope in Naeryong-ri, Cheongsong-gun (A-T-13), two taxa, *Paeoniajaponica* (Makino) Miyabe & Takeda and *Berchemiaberchemiifolia* (Makino) Koidz., were VU species of rare plants designated by the Korea Forest Service and LC species of the Red list. Twelve taxa, including *Asarumchungbuensis* (C.S. Yook & J.G. Kim) B.U. Oh were endemic plants of the Korean Peninsula. Ninteen taxa, including *Dryopterisfragrans* (L.) Schott were floristic target species of degree III or above. Twenty-four taxa, including *Rumexcrispus* L. were alien plants and twenty-two taxa were invasive alien plants, out of which 12 were WS species, including *Ambrosiaartemisiifolia* L., six were SS species, including *Festucaarundinacea* Schreb., three were CS species, including *Quamoclitcoccinea* (L.) Moench and one was an MS species, *Tagetesminuta* L. On the algific talus slope in Nammyeong-ri, Milyng-si (A-T-14), one taxon, *Deutziapaniculata* Nakai, was an EN species of rare plants designated by the Korea Forest Service and an NT species of the Red list. Eleven taxa, including *Carexokamotoi* Ohwi were endemic plants of the Korean Peninsula or endemic species. Fourteen taxa, including *Ulmuslaciniata* (Trautv.) Mayr were floristic target species of degree III or above. Six taxa, including *Oenotherabiennis* L., were invasive alien plants and five taxa were introduced disturbing plants, out of which three were WS species, including *Galinsogaquadriradiata* Ruiz & Pav., one was an SS species, *Euphorbiamaculata* L. and one was an MS species, *Tagetesminuta* L.

On the algific talus slope in Dongmak-ri, Yeoncheon-gun (A-C-1), one taxon, AspleniumtrichomanesL.subsp.quadrivalens D.E. Mey., was a CR species of rare plants designated by the Korea Forest Service; two taxa, *Rosakoreana* Kom. and *Forsythiasaxatilis* (Nakai) Nakai, were EN species and three taxa, including *Adiantumpedatum* L. were VU species. One taxon, *Athyriumspinulosum* (Maxim.) Milde, was a VU species of the Red list and two taxa, *Rosakoreana* Kom. and *Forsythiasaxatilis* (Nakai) Nakai., were NT species. Thirteen taxa, including Asarummandshuricum(Maxim.)M. Kim & S. Sovar.seoulense (Nakai) M. Kim & S. So were endemic plants of the Korean Peninsula or endemic species. Twenty-eight taxa, including *Callicarpadichotoma* (Lour.) Raeusch. ex K. Koch were floristic target species of degree III or above. Five taxa were invasive alien plants, out of which two were WS species, including *Erechtiteshieraciifolius* (L.) Raf. ex DC. and *Bidensfrondosa* L. and three were MS species, including *Barbareavulgaris* W.T. Aiton. Two taxa, *Sicyosangulatus* L. and *Ambrosiatrifida* L., were introduced disturbing plants. On the algific talus slope in Unchi-ri, Jeongseon-gun (A-C-2), two taxa, *Cystopterisfragilis* (L.) Bernh. and *Aconitumcoreanum* (H. Lév.) Rapaics, were CR species of rare plants designated by the Korea Forest Service; two taxa, *Aconitumcoreanum* (H. Lév.) Rapaics and *Spiraeachartacea* Nakai, were VU species of the Red list and one taxon, *Cystopterisfragilis* (L.) Bernh., was an NT species. Ten taxa, including *Paulowniacoreana* Uyeki were endemic plants of the Korean Peninsula or endemic species. Thirty taxa, including *Ribesmandshuricum* (Maxim.) Kom. were floristic target species of degree III or above. Thirty-four taxa, including *Rumexacetosella* L. were alien plants and twenty-five taxa were invasive alien plants, out of which thirteen were WS species, including *Veronicapersica* Poir., four were SS species, including *Carduuscrispus* L., two were CS species, *Chenopodiumalbum* L. and *Euphorbiahypericifolia* L., three were MS species, including *Ipomoeanil* (L.) Roth and three were PS species, including *Anthriscuscaucalis* M.Bieb. Two taxa, *Rumexacetosella* L. and *Ambrosiaartemisiifolia* L., were introduced disturbing plants. On the algific talus slope in Jwapo-ri, Jinahn-gun (A-C-3), one taxon, *Taxuscuspidata* Siebold & Zucc., was a VU species of rare plants designated by the Korea Forest Service and one taxon, *Prunus×yedoensis* Matsum., was an EN species of the Red list. Four taxa, including *Broussonetia×hanjiana* M. Kim were endemic plants of the Korean Peninsula. Six taxa, including *Woodsiamacrochlaena* Mett. ex Kuhn were floristic target species of degree III or above. Twenty-four taxa, including *Violasororia* Willd. were alien plants and twenty taxa were invasive alien plants, out of which eleven were WS species, including *Phytolaccaamericana* L.; three were SS species, including *Sonchusasper* (L.) Hill, four were MS species, including *Rumexobtusifolius* L. and two were PS species, *Lamiumpurpureum* L. and *Violasororia* Willd. On the algific talus slope in Binggye-ri, Uiseong-gun (A-C-4), six taxa were rare plants designated by the Korea Forest Service, out of which one was a CR species, *Cystopterisfragilis* (L.) Bernh., two were EN species, *Astilboidestabularis* (Hemsl.) Engl. and *Dracocephalumargunense* Fisch. ex Link and three were VU species, including *Tylophorafloribunda* Miq. One taxon, *Spiraeachartacea* Nakai, was a VU species of the Red list and two taxa, *Cystopterisfragilis* (L.) Bernh. and *Astilboidestabularis* (Hemsl.) Engl., were NT species. Eight taxa, including *Artemisiaangustissima* Nakai were endemic plants of the Korean Peninsula or endemic species. Twenty-eight taxa, including *Laporteacuspidata* (Wedd.) Friis were floristic target species of degree III or above. Thirty-two taxa, including *Solidagoaltissima* L. were alien plants. Twenty-one taxa were invasive alien plants out of which ten were WS species, including *Erigeronannuus* (L.) Pers., four were SS species, including *Poapratensis* L., five were CS species, including *Medicagosativa* L., one was an MS species: *Tagetesminuta* L., and one was a PS species: *Solidagoaltissima* L. Two taxa: *Ambrosiaartemisiifolia* L. and *Solidagoaltissima* L., were introduced disturbing plants.

On the algific talus slope in Changchon-ri, Hongcheon-gun (A-D-1), two taxa, *Micranthesoctopetala* (Nakai) Y.I. Kim & Y.D. Kim and *Oplopanaxelatus* (Nakai) Nakai, were EN species of rare plants designated by the Korea Forest Service and three taxa, including *Taxuscuspidata* Siebold & Zucc. were VU species. One taxon, *Piceajezoensis* (Siebold & Zucc.) Carrière, was an EN species of the Red list, two taxa, *Gymnocarpiumdryopteris* (L.) Newman and *Oplopanaxelatus* (Nakai) Nakai, were VU species and two taxa, *Lycopodiumannotinum* L. and Thujakoraiensis Nakai, were NT species. One taxon, *Micranthesoctopetala* (Nakai) Y.I. Kim & Y.D. Kim, was an endemic plant of the Korean Peninsula. Nineteen taxa, including *Lycopodiumobscurum* L. and *Enemionraddeanum* Regel were floristic target species of degree III or above. No alien plant was detected. On the algific talus slope in Neunggang-ri, Jecheon-si (A-D-2), one taxon, *Paeoniajaponica* (Makino) Miyabe & Takeda, was a VU species of rare plants designated by the Korea Forest Service and an LC species of the Red list. Eleven taxa, including *Angelicareflexa* B.Y. Lee were endemic plants of the Korean Peninsula or endemic species. Sixteen taxa, including *Athyriumiseanum* Rosenst. were floristic target species of degree III or above. Four taxa were invasive alien plants out of which *Chenopodiumficifolium* Sm. was a WS species, *Barbareavulgaris* W.T. Aiton was an MS species and two were PS species, including *Anthriscuscaucalis* M.Bieb. and *Ageratinaaltissima* (L.) R.M. King & H. Rob. One taxon, *Ageratinaaltissima* (L.) R.M. King & H. Rob., was an introduced disturbing plant. On the algific talus slope in Goobyeong-ri, Boeun-gun (A-D-3), two taxa, *Actaeabifida* (Nakai) J.Compton and *Paeoniajaponica* (Makino) Miyabe & Takeda, were VU species of rare plants designated by the Korea Forest Service and one taxon, *Spiraeachartacea* Nakai, was a VU species of the Red list. Twelve taxa, including *Hemerocallishakuunensis* Nakai were endemic plants of the Korean Peninsula or endemic species. Ten taxa, including *Hostaclausa* Nakai were floristic target species of degree III or above; no alien plant was detected. On the algific talus slope in Samyang-ri, Milyang-si (A-D-4), one taxon, *Deutziapaniculata* Nakai, was an EN species of rare plants designated by the Korea Forest Service and an NT species of the Red list. Five taxa, including *Stewartiakoreana* Nakai ex Rehder were endemic plants of the Korean Peninsula or endemic species. Five taxa, including Melampyrumsetaceum(Maxim. ex Palib.)Nakaivar.nakaianum (Tuyama) T. Yamaz. were floristic target species of degree III or above; no alien plant was detected. It is notable that Samyang-ri exhibited the lowest number of plants among the 25 sites of algific talus slope.

On the algific talus slope in Seonheul-ri, Jeju-si (A-V-1), three taxa were rare plants designated by the Korea Forest Service out of which *Cyrtosiaseptentrionalis* (Rchb.f.) Garay was a CR species and two were VU species, *Illiciumanisatum* L. and *Calanthediscolor* Lindl. Two taxa, *Cyrtosiaseptentrionalis* (Rchb.f.) Garay and *Goodyerahenryi* Rolfe, were NT species of the Red list. One taxon, *Sasaquelpaertensis* Nakai, was an endemic plant of the Korean Peninsula. Twenty-four taxa, including *Kadsurajaponica* (L.) Dunal and *Strobilanthesoliganthus* Miq. were floristic target species of degree III or above. One taxon, *Zingibermioga* (Thunb.) Roscoe, was a PS species of invasive alien plants. On the algific talus slope in Gwandong-ri, Haenam-gun (A-O-1), one taxon, *Cymbidiumgoeringii* (Rchb.f.) Rchb.f., was a Red list species. Three taxa, including *Hostaminor* (Baker) Nakai were endemic plants of the Korean Peninsula. Four taxa, including Rhaphiolepisindica(L.)Lindl. ex Kervar.umbellata (Thunb. ex Murray) H. Ohashi were floristic target species of degree III or above; no alien plant was detected. On the algific talus slope in Chooseong-ri, Hamyang-gun (A-O-2), one taxon, *Aconitumaustrokoreense* Koidz., was a VU species of rare plants designated by the Korea Forest Service and an NT species of the Red list. Four taxa, including ThalictrumactaeifoliumSiebold & Zucc.var.brevistylum Nakai were endemic plants of the Korean Peninsula. Four taxa, including *Dicentraspectabilis* (L.) Lem. were floristic target species of degree III or above; no alien plant was detected.

## Discussion

The vascular flora at 25 sites of algific talus slopes was investigated as a specific area of forest biodiversity. The area of each site was approximately 0.25 km^2^, which accounted for approximately 0.0004% of the total forest area of 62,860 km^2^ in South Korea (Korea Forest Service 2023). Despite the relative narrowness of the area, the overall vascular flora across the 25 algific talus slopes comprised 1,052 taxa of 125 families, 486 genera, 947 species, 23 subsp., 75 var. and 7 f., thereby accounting for approximately 22.27% of all 4,724 taxa of known vascular plants in South Korea. Moreover, there were 55 and 52 detected taxa of rare plants as designated by the Korea Forest Service and Red list species, respectively. This accounted for approximately 10.09% of the total 545 taxa of rare plants and 9.58% of the total 543 taxa of Red list species. On the algific talus slope in Bangnae-ri, Hongcheon-gun (A-T-2), the area of the natural habitat of *Vacciniumvitis-idaea* L. is rapidly decreasing due to the spread of *Muhlenbergiahakonensis* (Hack. ex Matsum.) Makino, thereby implicating the need for developing ex-situ conservation measures. There were 54 (12.53%) and 44 (11.70%) detected taxa of endemic plants of the Korean Peninsula and endemic species, respectively. On the algific talus slope in Yeotan-ri, Jeongseon-gun (A-T-4), *Forsythiasaxatilis* (Nakai) Nakai was found with phenotypic features similar to *Forsythiaovata* Nakai, thereby demanding taxonomic reinterpretation through genetic analyses. Further, 317 taxa of floristic target species were found and out of which 163 taxa of Degree III–V should be subjected to long-term monitoring plans and in-situ and ex-situ conservation measures. Moreover, long-term measures for ecological conservation should be developed via selecting plant species vulnerable to climate change on the 25 algific talus slope sites. There were 181 detected taxa of northern lineage plants of the Korean Peninsula; 60 taxa out of the 300 species threatened by climate change were also detected. The floristic inventory for northern lineage plants of the Korean Peninsula according to Gantsetseg et al. (2020) presents 616 species that are difficult to define as northern lineage plants. This appears to be because the inventory focuses solely on the shared species between South Korea and Russia so that the pattern of distribution for each taxon on the Korean Peninsula deviates to induce errors. Hence, a new floristic inventory should be produced for northern lineage plants based on the findings of this study; further studies are encouraged. For limestone area plants, 32 taxa (31.37%), including three taxa of CIP (21.43%), six taxa of SCP (20.00%) and 23 taxa of CCP (39.66%) were found. Seventy-five taxa of alien plants were detected, which account for 12.12% of the total 619 taxa of alien plants. However, care should be taken as most of the 25 algific talus slope sites are close to roads with high levels of accessibility and increasing number of tourists, which may consequently propel the continuously increasing spread of alien plants. In particular, tendency of alien plants to spread to areas surrounding the core areas of algific talus slope sites implies the need for suitable management measures. Systematic and continuous development of measures is necessary to ensure integrated conservation and management of the 25 algific talus slope sites. Currently, however, only very few regions with an algific talus slope, such as Bangnae-ri, Hongcheon-gun (A-T-2), are designated as reserve areas. Therefore, the FGRR and OECMs should be applied to a greater scope of regions for systematic management (Lee et al. 2022b). An area of OECMs is not a reserve area but a geographically defined area, which is managed in ways to achieve positive and continuous long-term results for the in-situ conservation of biodiversity and regarding the relevant ecosystem functions, services and cultural, socioeconomic and other regional values (Convention on Biological Diversity 2018). In South Korea, seven regions, including algific talus slopes and buffer areas of the Korea National Arboretum have been selected for OECMs and the results verified the effectiveness of OECMs as a means to extend the quantitative scope of reserve areas (Hong et al. 2017). Thus, algific talus slopes are thought to become a representative set of regions for OECMs. Furthermore, the day and night temperatures distinctly vary in regions such as the algific talus slope in Yeotan-ri and functions such as the cooling effect should be thoroughly analyzed through micrometeorological studies in order to provide pre-emptive responses against climate change.

## Supplementary Material

BF9E9B3B-A09D-5EA6-9CD6-11F8BAA3A40A 10.3897/BDJ.11.e113952.suppl1 Supplementary material 1 Location of algific talus slopes in South Korea Data type MS word File: oo_889104.docx https://binary.pensoft.net/file/889104 Lee, J.W., H. G. Yun, T. Y. Hwang, K. M. Kim, S. H. Jung and J. B. An

EFBC67BF-AAA5-5424-92AF-9A003F2D706D 10.3897/BDJ.11.e113952.suppl2 Supplementary material 2 Flora of algific talus slopes in South Korea Data type MS word File: oo_889105.docx https://binary.pensoft.net/file/889105 Lee, J.W., H. G. Yun, T. Y. Hwang, K. M. Kim, S. H. Jung and J. B. An

3D710FC3-5CD1-5FBF-B93F-8D822EDC3EB8 10.3897/BDJ.11.e113952.suppl3 Supplementary material 3 Total list of vascular plants of algific talus slope of South Korea Data type MS word File: oo_889106.docx https://binary.pensoft.net/file/889106 Lee, J.W., H. G. Yun, T. Y. Hwang, K. M. Kim, S. H. Jung and J. B. An

8750267C-DDF0-5456-9D4B-BEF384ACE0EC 10.3897/BDJ.11.e113952.suppl4 Supplementary material 4 List of rare plants and Red-list species in the algific talus slopes in South Korea Data type MS word File: oo_889108.docx https://binary.pensoft.net/file/889108 Lee, J.W., H. G. Yun, T. Y. Hwang, K. M. Kim, S. H. Jung and J. B. An

026C320D-0DB2-57E0-A6A2-1B92C01FE989 10.3897/BDJ.11.e113952.suppl5 Supplementary material 5 List of endemic plants on the Korean peninsula in the algific talus slopes of South Korea Data type MS word File: oo_889109.docx https://binary.pensoft.net/file/889109 Lee, J.W., H. G. Yun, T. Y. Hwang, K. M. Kim, S. H. Jung and J. B. An

5D84B4F7-FB73-5BC2-B8EE-C94379E1A861 10.3897/BDJ.11.e113952.suppl6 Supplementary material 6 List of the Ⅴ–Ⅰ degree taxa of Korean floristic target species on the algific talus slope in South Korea Data type MS word File: oo_889110.docx https://binary.pensoft.net/file/889110 Lee, J.W., H. G. Yun, T. Y. Hwang, K. M. Kim, S. H. Jung and J. B. An

F494DCCF-B378-591E-898C-525F46A26E01 10.3897/BDJ.11.e113952.suppl7 Supplementary material 7 List of the northern lineage plants on the Korean Peninsula and 300 species threatened by climate change in the algific talus slopes of South Korea Data type MS word File: oo_889111.docx https://binary.pensoft.net/file/889111 Lee, J.W., H. G. Yun, T. Y. Hwang, K. M. Kim, S. H. Jung and J. B. An

905A4BE3-7427-506D-A4B0-C7EE50A614E9 10.3897/BDJ.11.e113952.suppl8 Supplementary material 8 List of calciphilous plants in the algific talus slopes of South Korea Data type MS word File: oo_889113.docx https://binary.pensoft.net/file/889113 Lee, J.W., H. G. Yun, T. Y. Hwang, K. M. Kim, S. H. Jung and J. B. An

0B82D6B7-DD66-5EDF-AEF2-460DAD31DFAD 10.3897/BDJ.11.e113952.suppl9 Supplementary material 9 List of alien plants in the algific talus slopes of South Korea Data type MS word File: oo_889114.docx https://binary.pensoft.net/file/889114 Lee, J.W., H. G. Yun, T. Y. Hwang, K. M. Kim, S. H. Jung and J. B. An

7FD7F65B-B964-5ECC-833F-EE80CC20FB66 10.3897/BDJ.11.e113952.suppl10 Supplementary material 10 Flora of algific talus slopes by type in South Korea Data type MS word File: oo_889115.docx https://binary.pensoft.net/file/889115 Lee, J.W., H. G. Yun, T. Y. Hwang, K. M. Kim, S. H. Jung and J. B. An

CD42B063-5ACC-5BB1-A650-03DD876E11F4 10.3897/BDJ.11.e113952.suppl11 Supplementary material 11 Flora of algific talus slopes by region in South Korea Data type MS word File: oo_889116.docx https://binary.pensoft.net/file/889116 Lee, J.W., H. G. Yun, T. Y. Hwang, K. M. Kim, S. H. Jung and J. B. An

## References

[B10449634] An JB, Lee JW (2023). Occurrences of vascular plant on algific talus slopes in South Korea.

[B10375859] Boo Kyung-On, Kwon Won-Tae, Baek Hee-Jeong (2006). Change of extreme events of temperature and precipitation over Korea using regional projection of future climate change. Geophysical Research Letters.

[B10375868] Chung Gyu Young, Chang Kae Sun, Chung Jae-Min, Choi Hyeok Jae, Paik Weon-Ki, Hyun Jin-Oh (2017). A checklist of endemic plants on the Korean Peninsula. Korean Journal of Plant Taxonomy.

[B10377637] Diversity Convention on Biological (2018). Protected areas and other effective area-based conservation measures: The subsidiary body on scientific, technical and technological advice. https://www.cbd.int/doc/c/9b1f/759a/dfcee171bd46b06cc91f6a0d/sbstta-22-l-02-en.pdf.

[B10375893] Gantsetseg A, Jung SY, Cho WB, Han EK (2020). Definition and species list of northern lineage plants on the Korean Peninsula. The Society of Korean Herbal Medicine Information.

[B10375909] Hong JP, Shim YJ, Heo HY (2017). Identifying other effective area-based conservation measures for expanding national protected areas. The Korea Society of Environmental Restoration Technology.

[B10375954] Iokawa Y, Ishizawa S (2003). Vascular plants of wind-hole areas in Japan. Journal of Phytogeography and Taxonomy.

[B10375963] Kang ES, Lee SR, Oh SH, Kim DK, Jung SY, Son DC (2020). Comprehensive review about alien plants in Korea. Korean Journal of Plant Taxonomy.

[B10375993] Kim JM, Yim YJ, Jeon US (2000). Naturalized plant of Korea.

[B10376010] Kim Jin-Seok, Chung Jae-Min, Kim Jung-Hyun, Lee Woong, Lee Byoung-Yoon, Pak Jae-Hong (2016). Floristic study and conservation management strategies of algific talus slopes on the Korean peninsula. Korean Journal of Plant Taxonomy.

[B10375974] Kim JH, Nam GH, Lee SB, Shin SK, Kim JS (2021). A checklist of vascular plants in limestone areas on the Korean Peninsula. Korean Journal of Plant Taxonomy.

[B10376021] Kim SY, Lee SH (2011). The Impact of climate changes on highland agriculture region in Taeback mountainous. KU Climate Research Institute.

[B10376030] Kong WS, Lee SG, Yoon KH, Park HN (2011). Environmental characteristics of Wind-Hole and phytogeographical values. Journal of Environmental Impact Assessment.

[B10376040] Kong WS, Yoon KH, Kim IT, Lee YM (2012). Spatial distributional characteristics of wind-hole and governance strategy. Korean Society of Environmental Impact Assessment.

[B10376049] Kong WS, Kim GO, Lee SG, Park HN, Kim HH, Kim DB (2017). Vegetation and landscape characteristics at the peaks of Mts. Seorak, Jiri and Halla. Journal of Climate Change Research.

[B10376069] Service Korea Forest (2023). Annual report on forestry and forestry trends in 2021. https://www.forest.go.kr/kfsweb/cop/bbs/selectBoardArticle.do?bbsId=BBSMSTR_1008&mn=NKFS_06_09_05&nttId=3166932.

[B10376078] Envrionment Korea Ministry of Act on wildlife protection and management. https://www.law.go.kr/.

[B10376095] Arboretum Korea National (2008). Illustrated Pteridophytes of Korea.

[B10376121] Arboretum Korea National (2008). Rare plants data book in Korea.

[B10376155] Arboretum Korea National (2010). 300 Target plants adaptable to climate change in the Korean Peninsula.

[B10376163] Arboretum Korea National (2011). Illustrated Grasses of Korea.

[B10376180] Arboretum Korea National (2012). Illustrated Conifers of Korea.

[B10376188] Arboretum Korea National (2013). Air holes in Korea.

[B10376206] Arboretum Korea National (2016). Illustrated Cyperaceae of Korea.

[B10376311] Arboretum Korea National (2019). Checklist of alien plants in Korea.

[B10376319] Arboretum Korea National (2019). Illustrated Juncaceae, Eriocaulaceae, Typhaceae of Korea.

[B10376327] Arboretum Korea National (2021). National Red List of vascular plant in Korea.

[B10376335] Arboretum Korea National (2022). Checklist of vascular plants in Korea.

[B10376351] Arboretum Korea National List of National Standard Plants. http://www.nature.go.kr/kpni/index.do.

[B10376368] Lee JW, Yun HG, Kim DH (2022). A study on the flora and its naturalized plants of Mt. Teomo · Hyeolgu (Incheon, Ganghwa-gun) in the Western Part of DMZ, Korea. Korean Journal of Environment and Ecology.

[B10376389] Lee JW, Yun HG, Hwang TY, An JB (2022). Floristic study of algific talus slope (Yeotan-ri, Jeongseon-gun) in a specific area of Forest Biodiversity. Korean Journal of Plant Resources.

[B10376432] Lee TB (2014). Coloured flora of Korea, Vol. 1.

[B10376440] Lee TB (2014). Coloured flora of Korea, Vol. 2.

[B10376457] Locke H, Rockstrom J, Bakker P, Bapna M, Conservation IUCN World (2021). A Nature-Positive World: The Global Goal for Nature. A nature-positive world: The global goal for nature.

[B10376510] Melchior H (1964). Engler's syllabus der pflanzenfamilien mit besonderer berücksichtigung der nutzpflanzen nebst einer übersicht über die florenreiche und florengebiete der erde.

[B10376518] Resources National Institute of Biological (2013). Endemic species of Korea: Plantae.

[B10376539] Resources National Institute of Biological (2020). The inventory of endemic species on the Korean Peninsula.

[B10376547] Resources National Institute of Biological (2021). Red data book of Republic of Korea (Vascular plants).

[B10376555] Ecology National Institute of (2018). Floristic target species in Korea.

[B10376563] Numata M, Kotaki O (1975). Naturalized plants.

[B10376571] Park CW (2017). A study on the characteristics of warm wind hole zone of talus slope in Mt. Mudeung National Park. Journal of the Association of Korean Geographers.

[B10391702] The Royal Botanic Garden Kew Plants of the World Online. https://powo.science.kew.org/.

[B10376598] Nations United, Nations United (1992). Convention on Biological Diversity. Convention on Biological Diversity.

