# Supplementary material for: Floristic inventory and distribution characteristics of algific talus slopes in a specific area of forest biodiversity in South Korea
Source: Biodivers Data J. 2023 Dec 18;11:e113952. doi: 10.3897/BDJ.11.e113952 (PMC10838045; doi:10.3897/BDJ.11.e113952)
Supplement: Supplementary material 1 — Location of algific talus slopes in South Korea [file bdj-11-e113952-s001.docx]

1. Location of algific talus slopes in South Korea

| ID | Type | Location | GPS | Altitude  (m) | Ownership | Accessibility |
| --- | --- | --- | --- | --- | --- | --- |
| A-T-1 | Talus | Gyeonggi, Pocheon | 127°16′146″E, 38°00′817″N | 150–250 | Public | Good |
| A-T-2 |  | Gangwon, Hongcheon | 128°16′150″E, 37°48′288″N | 510–825 | Private | Good |
| A-T-3 |  | Gangwon, Pyeongchang | 128°35´305″E, 37°35´031″N | 890–920 | Private | Bad |
| A-T-4 |  | Gangwon, Jeongseon | 128°43′370″E, 37°21′940″N | 350–430 | Private | Good |
| A-T-5 |  | Gangwon, Jeongseon | 128°41´089″E, 37°27´101″N | 400–450 | National | Good |
| A-T-6 |  | Gangwon, Hwacheon | 127°35´698″E, 38°12´453″N | 350–450 | National | Good |
| A-T-7 |  | Gangwon, Inje | 128°06´658″E, 37°59´516″N | 170–300 | Public | Good |
| A-T-8 |  | Chungbuk, Boeun | 127°48´044″E, 36°28´463″N | 200–300 | Private | Good |
| A-T-9 |  | Chungbuk, Danyang | 128°16´888″E, 36°54´926″N | 400–450 | National | Good |
| A-T-10 |  | Jeonbuk, Jeongeup | 127°35´698″E, 38°12´453″N | 300–350 | National | Normal |
| A-T-11 |  | Gyeongbuk, Yeongcheon | 128°57´620″E, 36°09´695″N | 550–600 | Public | Bad |
| A-T-12 |  | Gyeongbuk, Gunwi | 128°46´503″E, 36°07´532″N | 100–150 | National | Good |
| A-T-13 |  | Gyeongbuk, Cheongsong | 129°13′428″E, 36°18′955″N | 240–330 | Private | Good |
| A-T-14 |  | Gyeongnam, Miryang | 128°59′030″E, 35°34′480″N | 400–700 | Private | Normal |
| A-C-1 | Cave | Gyeonggi, Yeoncheon | 127°06′630″E, 38°04′990″N | 70–350 | Private | Good |
| A-C-2 |  | Gangwon, Jeongseon | 128°37′120″E, 37°15′832″N | 320–400 | National | Good |
| A-C-3 |  | Jeonbuk, Jinan | 127°17′743″E, 35°43′803″N | 260–380 | National | Good |
| A-C-4 |  | Gyeongbuk, Uiseong | 128°45′327″E, 36°13′885″N | 140–180 | National | Good |
| A-D-1 | Dent | Gangwon, Hongcheon | 128°28´439″E, 37°44´211″N | 1,300–1,400 | National | Bad |
| A-D-2 |  | Chungbuk, Jecheon | 128°14′702″E, 36°59′447″N | 680–750 | National | Bad |
| A-D-3 |  | Chungbuk, Boeun | 128°41´089″E, 37°27´101″N | 825–870 | Public | Bad |
| A-D-4 |  | Gyeongnam, Miryang | 128°56´623″E, 35°37´123″N | 600–650 | Private | Bad |
| A-V-1 | Vertical cave | Jeju, Jeju | 126°43′237″E, 33°27´120″N | 350–450 | National | Bad |
| A-O-1 | Others | Jeonnam, Haenam | 126°27´646″E, 34°28´585″N | 160–170 | Private | Normal |
| A-O-2 |  | Gyeongnam, Hamyang | 127°48´044″E, 36°28´463″N | 670–700 | Private | Bad |
