# Supplementary material for: Floristic inventory and distribution characteristics of algific talus slopes in a specific area of forest biodiversity in South Korea
Source: Biodivers Data J. 2023 Dec 18;11:e113952. doi: 10.3897/BDJ.11.e113952 (PMC10838045; doi:10.3897/BDJ.11.e113952)
Supplement: Supplementary material 2 — Flora of algific talus slopes in South Korea [file bdj-11-e113952-s002.docx]

2. Flora of algific talus slopes in South Korea

| **Taxa** | **Families** | **Genera** | **Species** | **subsp.** | **var.** | **f.** | **Total** | **Ratio (%)** |
| --- | --- | --- | --- | --- | --- | --- | --- | --- |
| Pteridophyta | 16 | 35 | 74 | 1 | 2 | 0 | 77 | 7.3 |
| Gymnospermae | 5 | 9 | 14 | 0 | 0 | 0 | 14 | 1.3 |
| Angiospermae | 104 | 442 | 859 | 22 | 73 | 7 | 961 | 91.3 |
| Dicotyledoneae | 92 | 352 | 682 | 22 | 62 | 5 | 771 | 73.3 |
| Monocotyledoneae | 12 | 90 | 177 | 0 | 11 | 2 | 190 | 18.1 |
| Total | 125 | 486 | 947 | 23 | 75 | 7 | 1,052 | 100 |
