# Supplementary material for: Floristic inventory and distribution characteristics of algific talus slopes in a specific area of forest biodiversity in South Korea
Source: Biodivers Data J. 2023 Dec 18;11:e113952. doi: 10.3897/BDJ.11.e113952 (PMC10838045; doi:10.3897/BDJ.11.e113952)
Supplement: Supplementary material 3 — Total list of vascular plants of algific talus slope of South Korea [file bdj-11-e113952-s003.docx]

3. Total list of vascular plants of algific talus slope of South Korea

| Family name | Scientific and Korean name | In. | Ex. | A | B | C | D | E | F |
| --- | --- | --- | --- | --- | --- | --- | --- | --- | --- |
| Selaginellaceae | *Selaginella rossii* (Baker) Warb. 구실사리 |  | ○ |  |  |  |  |  |  |
| Equisetaceae | *Equisetum arvense* L. 쇠뜨기 | ○ | ○ |  |  |  |  |  |  |
| Dennstaedtiaceae | *Dennstaedtia wilfordii* (T. Moore) Christ 황고사리 |  | ○ |  |  |  |  |  |  |
| Dennstaedtiaceae | *Pteridium aquilinum* (L.) Kuhn var. *latiusculum* (Desv.) Underw. ex A. Heller 고사리 |  | ○ |  |  |  |  |  |  |
| Cystopteridaceae | *Gymnocarpium dryopteris* (L.) Newman 토끼고사리 | ○ | ○ | DD |  | ○ | Ⅲ |  |  |
| Cystopteridaceae | *Gymnocarpium jessoense* (Koidz.) Koidz. 산토끼고사리 | ○ | ○ |  |  |  |  |  | SCP |
| Pteridaceae | *Cheilanthes argentea* (S.G. Gmel.) Kunze 부싯깃고사리 |  | ○ |  |  |  | Ⅰ |  |  |
| Aspleniaceae | *Asplenium ruprechtii* Sa. Kurata 거미고사리 |  | ○ |  |  | ○ |  |  |  |
| Woodsiaceae | *Woodsia manchuriensis* Hook. 만주우드풀 | ○ | ○ |  |  |  |  |  |  |
| Woodsiaceae | *Woodsia polystichoides* D.C. Eaton 우드풀 |  |  |  |  |  |  |  |  |
| Athyriaceae | *Deparia pycnosora* (Christ) M. Kato 털고사리 |  | ○ |  |  |  |  |  |  |
| Dryopteridaceae | *Dryopteris crassirhizoma* Nakai 관중 |  | ○ |  |  | ○ |  |  |  |
| Pinaceae | *Larix kaempferi* (Lamb.) Carrière 일본잎갈나무 | ○ |  |  |  |  |  | ○ |  |
| Pinaceae | *Pinus densiflora* Siebold & Zucc. 소나무 |  | ○ |  |  | ○ |  |  |  |
| Juglandaceae | *Juglans mandshurica* Maxim. 가래나무 |  | ○ |  |  |  | Ⅰ |  |  |
| Salicaceae | *Populus tremula* L. var. *davidiana* (Dode) C.K. Schneid. 사시나무 | ○ |  |  |  |  |  |  |  |
| Salicaceae | *Salix caprea* L. 호랑버들 | ○ | ○ |  |  |  |  |  |  |
| Salicaceae | *Salix gracilistyla* Miq. 갯버들 |  | ○ |  |  | ○ |  |  |  |
| Salicaceae | *Salix koriyanagi* Kimura ex Goerz 키버들 |  | ○ |  | ○ |  |  |  |  |
| Salicaceae | *Salix pierotii* Miq. 버드나무 |  | ○ |  |  |  |  |  |  |
| Betulaceae | *Betula chinensis* Maxim. 개박달나무 |  | ○ |  |  |  | Ⅲ |  |  |
| Betulaceae | *Betula costata* Trautv. 거제수나무 |  | ○ |  |  | ○ | Ⅲ |  |  |
| Betulaceae | *Betula davurica* Pall. 물박달나무 |  | ○ |  |  | ○ | Ⅲ |  |  |
| Betulaceae | *Betula schmidtii* Regel 박달나무 |  | ○ |  |  | ○ | Ⅲ |  |  |
| Betulaceae | *Carpinus cordata* Blume 까치박달 |  | ○ |  |  |  |  |  |  |
| Betulaceae | *Carpinus turczaninovii* Hance 소사나무 |  | ○ |  |  |  | Ⅰ |  |  |
| Betulaceae | *Corylus heterophylla* Fisch. ex Trautv. 개암나무 | ○ | ○ |  |  |  |  |  |  |
| Betulaceae | *Corylus sieboldiana* Blume 참개암나무 | ○ | ○ |  |  | ○ |  |  |  |
| Fagaceae | *Quercus aliena* Blume 갈참나무 |  | ○ |  |  |  |  |  |  |
| Fagaceae | *Quercus dentata* Thunb. 떡갈나무 |  | ○ |  |  |  |  |  |  |
| Fagaceae | *Quercus mongolica* Fisch. ex Ledeb. 신갈나무 |  | ○ |  |  |  |  |  |  |
| Fagaceae | *Quercus variabilis* Blume 굴참나무 |  | ○ |  |  |  |  |  |  |
| Ulmaceae | *Celtis aurantiaca* Nakai 산팽나무 |  | ○ |  |  |  |  |  |  |
| Ulmaceae | *Ulmus davidiana* Planch. ex DC. var. *japonica* (Rehder) Nakai 느릅나무 | ○ | ○ |  |  |  | Ⅰ |  |  |
| Ulmaceae | *Ulmus macrocarpa* Hance 왕느릅나무 | ○ | ○ |  |  | ○ | Ⅳ |  | CCP |
| Moraceae | *Broussonetia × hanjiana* M.Kim 닥나무 |  | ○ |  | ○ |  |  |  |  |
| Moraceae | *Fatoua villosa* (Thunb.) Nakai 뽕모시풀 |  | ○ |  |  |  |  |  |  |
| Moraceae | *Morus bombycis* Koidz. 산뽕나무 |  | ○ |  |  |  |  |  |  |
| Cannabaceae | *Humulus scandens* (Lour.) Merr. 환삼덩굴 |  | ○ |  |  |  |  |  |  |
| Urticaceae | *Boehmeria japonica* (L.f.) Miq. 왜모시풀 |  | ○ |  |  |  |  |  |  |
| Urticaceae | *Boehmeria paraspicata* Nakai 풀거북꼬리 |  |  |  |  |  |  |  |  |
| Urticaceae | *Boehmeria platanifolia* (Maxim.) Franch & Sav. ex C.H.Wright 개모시풀 |  | ○ |  |  |  |  |  |  |
| Polygonaceae | *Persicaria senticosa* (Meisn.) H.Gross ex Nakai 며느리밑씻개 |  | ○ |  |  |  |  |  |  |
| Caryophyllaceae | *Dianthus chinensis* L. 패랭이꽃 |  | ○ |  |  |  |  |  |  |
| Caryophyllaceae | *Silene baccifera* (L.) Roth 덩굴별꽃 |  | ○ |  |  |  | Ⅰ |  |  |
| Caryophyllaceae | *Stellaria aquatica* (L.) Scop. 쇠별꽃 |  | ○ |  |  |  |  |  |  |
| Amaranthaceae | *Amaranthus retroflexus* L. 털비름 |  | ○ |  |  |  |  | ○ |  |
| Magnoliaceae | *Magnolia sieboldii* K.Koch 함박꽃나무 |  | ○ |  |  |  | Ⅱ |  |  |
| Schisandraceae | *Schisandra chinensis* (Turcz.) Baill. 오미자 | ○ | ○ |  |  | ○ | Ⅱ |  |  |
| Lauraceae | *Lindera obtusiloba* Blume 생강나무 | ○ | ○ |  |  |  |  |  |  |
| Ranunculaceae | *Aconitum barbatum* Patrin ex Pers. 노랑투구꽃 |  |  |  |  |  |  |  |  |
| Ranunculaceae | *Aconitum jaluense* Kom. 투구꽃 |  | ○ |  |  |  | Ⅰ |  |  |
| Ranunculaceae | *Aconitum pseudolaeve* Nakai 진범 |  | ○ |  | ○ |  |  |  |  |
| Ranunculaceae | *Actaea bifida* (Nakai) J.Compton 세잎승마 |  | ○ | VU | ○ |  | Ⅳ |  | CCP |
| Ranunculaceae | *Actaea dahurica* (Turcz. ex Fisch. & C.A.Mey.) Franch. 눈빛승마 |  | ○ |  |  |  |  |  |  |
| Ranunculaceae | *Anemone reflexa* Steph. ex Willd. 회리바람꽃 |  | ○ |  |  | ○ | Ⅳ |  |  |
| Ranunculaceae | *Clematis apiifolia* DC. 사위질빵 |  | ○ |  |  |  |  |  |  |
| Ranunculaceae | *Clematis brachyura* Maxim. 외대으아리 |  | ○ |  | ○ |  | Ⅲ |  |  |
| Ranunculaceae | *Clematis brevicaudata* DC. 좀사위질빵 | ○ | ○ | DD |  |  |  |  | SCP |
| Ranunculaceae | *Clematis fusca* Turcz. var. *violacea* Maxim. 종덩굴 |  | ○ |  |  |  | Ⅲ |  |  |
| Ranunculaceae | *Clematis serratifolia* Rehder 개버무리 |  | ○ |  |  | ○ | Ⅳ |  |  |
| Ranunculaceae | Clematis terniflora DC. var. *mandshuric*a (Rupr.) Ohwi 으아리 |  | ○ |  |  |  |  |  |  |
| Ranunculaceae | *Clematis trichotoma* Nakai 할미밀망 |  | ○ |  | ○ |  |  |  |  |
| Ranunculaceae | *Clematis urticifolia* Nakai ex Kitag. 병조희풀 | ○ | ○ |  | ○ |  | Ⅲ |  |  |
| Ranunculaceae | *Hepatica asiatica* Nakai 노루귀 |  | ○ |  |  |  | Ⅰ |  |  |
| Ranunculaceae | *Ranunculus japonicus* Thunb. 미나리아재비 |  | ○ |  |  |  |  |  |  |
| Ranunculaceae | *Ranunculus tachiroei* Franch. & Sav. 개구리미나리 |  | ○ |  |  | ○ |  |  |  |
| Ranunculaceae | *Semiaquilegia adoxoides* (DC.) Makino 개구리발톱 |  | ○ |  |  |  | Ⅰ |  |  |
| Ranunculaceae | *Thalictrum ichangense* Lecoy. ex Oliv. 꼭지연잎꿩의다리 | ○ | ○ |  |  |  | Ⅳ |  | CCP |
| Ranunculaceae | *Thalictrum minus* (Pamp.) Pamp. var*. hypoleucum* (Siebold & Zucc.) Miq. 좀꿩의다리 | ○ | ○ |  |  |  |  |  |  |
| Ranunculaceae | *Thalictrum tuberiferum* Maxim. 산꿩의다리 |  | ○ |  |  |  |  |  |  |
| Berberidaceae | *Berberis koreana* Palib. 매자나무 | ○ | ○ |  | ○ |  | Ⅳ |  |  |
| Menispermaceae | *Menispermum dauricum* DC. 새모래덩굴 |  | ○ |  |  | ○ |  |  |  |
| Chloranthaceae | *Chloranthus japonicus* Siebold 홀아비꽃대 |  | ○ |  |  | ○ | Ⅰ |  |  |
| Aristolochiaceae | *Aristolochia manshuriensis* Kom. 등칡 |  | ○ | LC |  | ○ | Ⅱ |  |  |
| Aristolochiaceae | *Asarum mandshuricum* (Maxim.) M.Kim & S. So var. *seoulense* (Nakai) M. Kim & S. So 서울족도리풀 |  | ○ |  | ○ |  |  |  |  |
| Aristolochiaceae | *Asarum sieboldii* Miq. 족도리풀 |  | ○ |  |  |  |  |  |  |
| Paeoniaceae | *Paeonia japonica* (Makino) Miyabe & Takeda 백작약 | ○ |  | VU |  |  | Ⅱ |  |  |
| Paeoniaceae | *Paeonia obovata* Maxim. 산작약 | ○ |  | CR |  | ○ | Ⅴ |  | CCP |
| Actinidiaceae | *Actinidia arguta* (Siebold & Zucc.) Planch. ex Miq. 다래 | ○ |  |  |  |  |  |  |  |
| Actinidiaceae | *Actinidia kolomikta* (Maxim. & Rupr.) Maxim. 쥐다래 |  | ○ |  |  |  | Ⅲ |  |  |
| Actinidiaceae | *Actinidia polygama* (Siebold & Zucc.) Planch. ex Maxim. 개다래 | ○ | ○ |  |  |  |  |  |  |
| Clusiaceae | *Hypericum ascyron* L. 물레나물 |  | ○ |  |  | ○ |  |  |  |
| Papaveraceae | *Corydalis speciosa* Maxim. 산괴불주머니 |  | ○ |  |  | ○ |  |  |  |
| Brassicaceae | *Arabis hirsuta* (L.) Scop. 털장대 |  | ○ |  |  |  |  |  |  |
| Brassicaceae | *Barbarea orthoceras* Ledeb. 나도냉이 |  | ○ |  |  | ○ |  |  |  |
| Brassicaceae | *Barbarea vulgaris* W.T.Aiton 유럽나도냉이 |  | ○ |  |  |  |  | ○ |  |
| Brassicaceae | *Capsella bursa-pastoris* (L.) Medik. 냉이 |  | ○ |  |  |  |  |  |  |
| Brassicaceae | *Cardamine impatiens* L. 싸리냉이 |  | ○ |  |  |  |  |  |  |
| Brassicaceae | *Cardamine leucantha* (Tausch) O.E.Schulz 미나리냉이 |  | ○ |  |  |  |  |  |  |
| Brassicaceae | *Draba nemorosa* L. 꽃다지 |  | ○ |  |  |  |  |  |  |
| Brassicaceae | *Thlaspi arvense* L. 말냉이 |  | ○ |  |  |  |  | ○ |  |
| Brassicaceae | *Turritis glabra* L. 장대나물 |  | ○ |  |  |  |  |  |  |
| Crassulaceae | *Phedimus aizoon* (L.) 't Hart 가는기린초 | ○ |  |  |  | ○ |  |  |  |
| Crassulaceae | *Phedimus kamtschaticus* (Fisch. & C.A.Mey.) 't Hart 기린초 | ○ |  |  |  |  |  |  |  |
| Saxifragaceae | *Astilbe chinensis* (Maxim.) Franch. & Sav. 노루오줌 |  | ○ |  |  |  |  |  |  |
| Saxifragaceae | *Mukdenia rossii* (Oliv.) Koidz. 돌단풍 | ○ | ○ |  |  |  | Ⅱ |  |  |
| Saxifragaceae | *Rodgersia podophylla* A.Gray 도깨비부채 |  | ○ | LC |  |  | Ⅳ |  |  |
| Hydrangeaceae | *Deutzia glabrata* Kom. 물참대 | ○ | ○ |  |  | ○ | Ⅰ |  |  |
| Hydrangeaceae | *Deutzia parviflora* Bunge 말발도리 |  | ○ |  |  | ○ | Ⅰ |  |  |
| Hydrangeaceae | *Philadelphus tenuifolius* Rupr. & Maxim. 얇은잎고광나무 | ○ | ○ |  |  | ○ |  |  |  |
| Parnassiaceae | *Parnassia palustris* L. 물매화 |  | ○ |  |  | ○ |  |  |  |
| Grossulariaceae | *Ribes mandshuricum* (Maxim.) Kom. 까치밥나무 |  | ○ |  |  | ○ | Ⅲ |  |  |
| Grossulariaceae | *Ribes maximowiczianum* Kom. 명자순 | ○ |  |  |  | ○ | Ⅲ |  |  |
| Rosaceae | *Aruncus dioicus* (Walter) Fernald 눈개승마 |  | ○ |  |  |  | Ⅲ |  |  |
| Rosaceae | *Crataegus pinnatifida* Bunge 산사나무 |  | ○ |  |  |  |  |  |  |
| Rosaceae | *Duchesnea indica* (Andrews) Teschem. 뱀딸기 |  | ○ |  |  |  |  |  |  |
| Rosaceae | *Malus baccata* (L.) Borkh. 야광나무 |  | ○ |  |  | ○ | Ⅰ |  |  |
| Rosaceae | *Malus mandshurica* (Maxim.) Kom. ex Skvortsov 털야광나무 | ○ |  |  |  |  | Ⅰ |  |  |
| Rosaceae | *Potentilla chinensis* Ser. 딱지꽃 | ○ |  |  |  | ○ |  |  |  |
| Rosaceae | *Potentilla dickinsii* Franch. & Sav. 돌양지꽃 |  | ○ |  |  |  | Ⅱ |  |  |
| Rosaceae | *Potentilla freyniana* Bornm. 세잎양지꽃 | ○ | ○ |  |  |  |  |  |  |
| Rosaceae | *Prunus choreiana* Nakai ex H.T.Im 복사앵도나무 | ○ |  | EN | ○ |  |  |  | CIP |
| Rosaceae | *Prunus padus* L. 귀룽나무 |  | ○ |  |  |  |  |  |  |
| Rosaceae | *Prunus persica* (L.) Batsch. 복사나무 |  | ○ |  |  |  |  | ○ |  |
| Rosaceae | *Prunus serrulata* Lindl. var. pubescens (Makino) Nakai 잔털벚나무 |  | ○ |  |  |  |  |  |  |
| Rosaceae | *Pyrus ussuriensis* Maxim. ex Rupr. 산돌배 |  | ○ |  |  | ○ |  |  |  |
| Rosaceae | *Rubus crataegifolius* Bunge 산딸기 | ○ | ○ |  |  | ○ |  |  |  |
| Rosaceae | *Rubus pungens* Cambess. 줄딸기 |  | ○ |  |  |  |  |  |  |
| Rosaceae | *Sanguisorba officinalis* L. 오이풀 |  | ○ |  |  |  |  |  |  |
| Rosaceae | *Sorbaria sorbifolia* (L.) A. Braun var. *stellipila* Maxim. 쉬땅나무 |  | ○ |  |  | ○ | Ⅲ |  |  |
| Rosaceae | *Spiraea blumei* G. Don 산조팝나무 | ○ | ○ |  |  |  | Ⅰ |  |  |
| Rosaceae | *Spiraea chartacea* Nakai 아구장나무 |  | ○ |  |  | ○ | Ⅰ |  |  |
| Rosaceae | *Spiraea chinensis* Maxim. 당조팝나무 | ○ | ○ |  |  |  | Ⅲ |  | CCP |
| Rosaceae | *Spiraea prunifolia* Siebold & Zucc. f. *simpliciflora* Nakai 조팝나무 | ○ | ○ |  |  |  |  |  |  |
| Rosaceae | *Spiraea salicifolia* L. 꼬리조팝나무 |  | ○ |  |  | ○ | Ⅱ |  |  |
| Rosaceae | *Spiraea trichocarpa* Nakai 갈기조팝나무 |  | ○ |  |  |  | Ⅳ |  | CCP |
| Rosaceae | *Stephanandra incisa* (Thunb.) Zabel 국수나무 |  | ○ |  |  |  |  |  |  |
| Fabaceae | *Amorpha fruticosa* L. 족제비싸리 |  | ○ |  |  |  |  | ○ |  |
| Fabaceae | *Amphicarpaea bracteata* (L.) Fernald subsp. *edgeworthii* (Benth.) H. Ohashi 새콩 |  | ○ |  |  |  |  |  |  |
| Fabaceae | *Astragalus penduliflorus* Lam. var. *dahuricu*s (DC.) X.Y. Zhu 황기 |  | ○ |  |  |  |  |  | SCP |
| Fabaceae | *Caragana fruticosa* (Pall.) Besser 참골담초 | ○ |  |  |  |  | Ⅳ |  | SCP |
| Fabaceae | *Kummerowia striata* (Thunb.) Schindl. 매듭풀 |  | ○ |  |  |  |  |  |  |
| Fabaceae | *Lathyrus davidii* Hance 활량나물 |  | ○ |  |  |  |  |  |  |
| Fabaceae | *Lespedeza bicolor* Turcz. 싸리 | ○ | ○ |  |  |  |  |  |  |
| Fabaceae | *Lespedeza cuneata* (Dum.Cours.) G.Don 비수리 |  |  |  |  |  |  |  |  |
| Fabaceae | *Lespedeza cyrtobotrya* Miq. 참싸리 |  |  |  |  |  |  |  |  |
| Fabaceae | *Lespedeza maximowiczii* C.K.Schneid. 조록싸리 | ○ | ○ |  |  |  |  |  |  |
| Fabaceae | *Maackia amurensis* Rupr. 다릅나무 | ○ |  |  |  | ○ |  |  |  |
| Fabaceae | *Medicago lupulina* L. 잔개자리 |  | ○ |  |  |  |  | ○ |  |
| Fabaceae | *Melilotus albus* Medik. 흰전동싸리 |  | ○ |  |  |  |  | ○ |  |
| Fabaceae | *Pueraria lobata* (Willd.) Ohwi 칡 |  | ○ |  |  |  |  |  |  |
| Fabaceae | *Robinia pseudoacacia* L. 아까시나무 |  | ○ |  |  |  |  | ○ |  |
| Fabaceae | *Sophora flavescens* Aiton 고삼 |  | ○ |  |  |  |  |  |  |
| Fabaceae | *Trifolium repens* L. 토끼풀 |  | ○ |  |  |  |  | ○ |  |
| Fabaceae | *Vicia amurensis* Oett. 벌완두 |  | ○ |  |  | ○ |  |  |  |
| Fabaceae | *Vicia unijuga* A.Braun 나비나물 | ○ | ○ |  |  | ○ |  |  |  |
| Oxalidaceae | *Oxalis corniculata* L. 괭이밥 |  | ○ |  |  |  |  | ○ |  |
| Oxalidaceae | *Oxalis stricta* L. 선괭이밥 |  | ○ |  |  |  |  |  |  |
| Geraniaceae | *Geranium koreanum* Kom. 둥근이질풀 |  | ○ |  | ○ |  | Ⅱ |  |  |
| Euphorbiaceae | *Euphorbia sieboldiana* Morren & Decne. 개감수 |  | ○ |  |  |  |  |  |  |
| Euphorbiaceae | *Securinega suffruticosa* (Pall.) Rehder 광대싸리 | ○ | ○ |  |  |  |  |  |  |
| Rutaceae | *Zanthoxylum schinifolium* Siebold & Zucc. 산초나무 | ○ | ○ |  |  |  |  |  |  |
| Simaroubaceae | *Picrasma quassioides* (D. Don) Benn. 소태나무 | ○ | ○ |  |  |  |  |  |  |
| Anacardiaceae | *Rhus chinensis* Mill. 붉나무 |  | ○ |  |  |  |  |  |  |
| Anacardiaceae | *Toxicodendron trichocarpum* (Miq.) Kuntze 개옻나무 |  | ○ |  |  |  |  |  |  |
| Aceraceae | *Acer pictum* Thunb. var. *mono* (Maxim.) Maxim. ex Franch. 고로쇠나무 | ○ | ○ |  |  |  |  |  |  |
| Aceraceae | *Acer pseudosieboldianum* (Pax) Kom. 당단풍나무 |  | ○ |  |  |  |  |  |  |
| Aceraceae | *Acer tataricum* L. subsp. *ginnala* (Maxim.) Wesm. 신나무 | ○ | ○ |  |  |  |  |  |  |
| Aceraceae | *Acer triflorum* Kom. 복자기 | ○ | ○ |  |  |  | Ⅲ |  |  |
| Balsaminaceae | *Impatiens textorii* Miq. 물봉선 |  | ○ |  |  |  |  |  |  |
| Celastraceae | *Euonymus alatus* (Thunb.) Siebold 화살나무 |  | ○ |  |  |  |  |  |  |
| Celastraceae | *Euonymus alatus* (Thunb.) Siebold f. *ciliato-dentatus* (Franch. & Sav.) Hiyama 회잎나무 |  | ○ |  |  |  |  |  |  |
| Celastraceae | *Euonymus pauciflorus* Maxim. 회목나무 | ○ | ○ |  |  |  | Ⅱ |  |  |
| Staphyleaceae | *Staphylea bumalda* DC. 고추나무 | ○ | ○ |  |  |  |  |  |  |
| Buxaceae | *Buxus sinica* (Rehder & E.H. Wilson) M. Cheng var. *insularis* (Nakai) M. Cheng 회양목 |  | ○ |  |  |  | Ⅰ |  | CCP |
| Rhamnaceae | *Hovenia dulcis* Thunb. 헛개나무 |  | ○ |  |  |  | Ⅰ |  |  |
| Rhamnaceae | *Rhamnus yoshinoi* Makino 짝자래나무 | ○ | ○ |  |  |  |  |  |  |
| Vitaceae | *Ampelopsis glandulosa* (Wall.) Momiy var. brevipedunculata (Maxim.) Momiy 개머루 | ○ | ○ |  |  |  |  |  |  |
| Vitaceae | *Parthenocissus tricuspidata* (Siebold & Zucc.) Planch. 담쟁이덩굴 | ○ | ○ |  |  |  |  |  |  |
| Vitaceae | *Vitis amurensis* Rupr. 왕머루 | ○ | ○ |  |  | ○ |  |  |  |
| Vitaceae | *Vitis coignetiae* Pulliat ex Planch. 머루 |  | ○ |  |  |  | Ⅲ |  |  |
| Tiliaceae | *Corchoropsis tomentosa* (Thunb.) Makino 수까치깨 |  |  |  |  |  |  |  |  |
| Tiliaceae | *Tilia amurensis* Rupr. 피나무 |  | ○ |  |  | ○ | Ⅱ |  |  |
| Tiliaceae | *Tilia mandshurica* Rupr. & Maxim. 찰피나무 | ○ | ○ |  |  | ○ | Ⅱ |  |  |
| Violaceae | *Viola acuminata* Ledeb. 졸방제비꽃 |  | ○ |  |  | ○ |  |  |  |
| Violaceae | *Viola albida* Palib. var. *chaerophylloide*s (Regel) F. Maek. ex H. Hara 남산제비꽃 |  | ○ |  |  |  |  |  |  |
| Violaceae | *Viola arcuata* Blume 콩제비꽃 |  | ○ |  |  |  |  |  |  |
| Violaceae | *Viola collina* Besser 둥근털제비꽃 |  | ○ |  |  |  |  |  |  |
| Violaceae | *Viola keiskei* Miq. 잔털제비꽃 |  | ○ |  |  |  |  |  |  |
| Violaceae | *Viola mandshurica* W. Becker 제비꽃 |  | ○ |  |  | ○ |  |  |  |
| Violaceae | *Viola orientalis* (Maxim.) W. Becker 노랑제비꽃 |  | ○ |  |  | ○ | Ⅱ |  |  |
| Violaceae | *Viola phalacrocarpa* Maxim. 털제비꽃 |  | ○ |  |  |  |  |  |  |
| Violaceae | *Viola selkirkii* Pursh ex Goldie 뫼제비꽃 |  | ○ |  |  | ○ |  |  |  |
| Violaceae | *Viola tenuicornis* W. Becker 자주알록제비꽃 |  | ○ |  |  |  |  |  |  |
| Violaceae | *Viola variegata* Fisch. ex Link 알록제비꽃 |  | ○ |  |  | ○ |  |  |  |
| Elaeagnaceae | *Elaeagnus umbellata* Thunb. 보리수나무 | ○ | ○ |  |  |  |  |  |  |
| Onagraceae | *Circaea mollis* Siebold & Zucc. 털이슬 |  | ○ |  |  |  |  |  |  |
| Onagraceae | *Oenothera biennis* L. 달맞이꽃 |  | ○ |  |  |  |  | ○ |  |
| Alangiaceae | *Alangium platanifolium* (Siebold & Zucc.) Harms var. *trilobum* (Miq.) Ohwi 박쥐나무 | ○ | ○ |  |  |  |  |  |  |
| Cornaceae | *Cornus controversa* Hemsl. 층층나무 |  | ○ |  |  |  |  |  |  |
| Cornaceae | *Cornus walteri* Wangerin 말채나무 |  |  |  |  |  |  |  |  |
| Araliaceae | *Kalopanax septemlobus* (Thunb.) Koidz. 음나무 | ○ | ○ |  |  |  |  |  |  |
| Apiaceae | *Angelica amurensis* Schischk. 지리강활 |  | ○ |  |  |  |  |  |  |
| Apiaceae | *Angelica dahurica* (Fisch. ex Hoffm.) Benth. & Hook.f. ex Franch. & Sav. 구릿대 |  | ○ |  |  |  |  |  |  |
| Apiaceae | *Angelica gigas* Nakai 참당귀 |  | ○ |  |  |  |  |  |  |
| Apiaceae | *Angelica polymorpha* Maxim. 궁궁이 |  | ○ |  |  |  |  |  |  |
| Apiaceae | *Angelica reflexa* B.Y. Lee 강활 |  | ○ |  | ○ |  |  |  |  |
| Apiaceae | *Anthriscus sylvestris* (L.) Hoffm. 전호 |  | ○ |  |  |  |  |  |  |
| Apiaceae | *Oenanthe javanica* DC. 미나리 |  | ○ |  |  |  |  |  |  |
| Apiaceae | *Peucedanum terebinthaceum* (Fisch. ex Trevir.) Fisch. ex Turcz. 기름나물 |  | ○ |  |  | ○ |  |  |  |
| Apiaceae | *Pimpinella brachycarpa* (Kom.) Nakai 참나물 |  | ○ |  |  |  |  |  |  |
| Apiaceae | *Torilis japonica* (Houtt.) DC. 사상자 |  | ○ |  |  |  |  |  |  |
| Ericaceae | *Rhododendron mucronulatum* Turcz. 진달래 |  | ○ |  |  | ○ |  |  |  |
| Ericaceae | *Rhododendron mucronulatum* Turcz. var. *ciliatum* Nakai 털진달래 |  | ○ |  |  |  |  |  |  |
| Primulaceae | *Lysimachia clethroides* Duby 큰까치수염 | ○ |  |  |  |  |  |  |  |
| Styracaceae | *Styrax obassia* Siebold & Zucc. 쪽동백나무 |  | ○ |  |  |  |  |  |  |
| Symplocaceae | *Symplocos sawafutagi* Nagam. 노린재나무 |  | ○ |  |  |  |  |  |  |
| Oleaceae | *Forsythia saxatilis* (Nakai) Nakai 산개나리 | ○ |  | EN | ○ |  | Ⅳ |  | CCP |
| Oleaceae | *Fraxinus rhynchophylla* Hance 물푸레나무 | ○ | ○ |  |  |  |  |  |  |
| Oleaceae | *Fraxinus sieboldiana* Blume 쇠물푸레나무 | ○ | ○ |  |  |  |  |  |  |
| Oleaceae | *Syringa fauriei* H.Lév. 버들개회나무 | ○ | ○ |  | ○ |  | Ⅳ |  |  |
| Oleaceae | *Syringa pubescens* Turcz. subsp. *patula* (Palib.) M.C.Chang & X.L.Chen 털개회나무 | ○ | ○ |  |  |  | Ⅰ |  |  |
| Oleaceae | *Syringa reticulata* (Blume) H.Hara 개회나무 |  | ○ |  |  | ○ | Ⅲ |  |  |
| Oleaceae | *Syringa villosa* Vahl subsp. *wolfii* (C.K.Schneid.) Y.Chen & D.Y.Hong 꽃개회나무 | ○ |  | LC |  | ○ | Ⅳ |  |  |
| Apocynaceae | *Metaplexis japonica* (Thunb.) Makino 박주가리 |  | ○ |  |  |  |  |  |  |
| Rubiaceae | *Asperula lasiantha* Nakai 갈퀴아재비 |  | ○ |  | ○ |  | Ⅴ |  |  |
| Rubiaceae | *Galium gracilens* (A.Gray) Makino 좀네잎갈퀴 |  | ○ |  |  |  |  |  |  |
| Rubiaceae | *Galium maximowiczii* (Kom.) Pobed. 개갈퀴 |  | ○ |  |  |  |  |  |  |
| Rubiaceae | *Galium pogonanthum* Franch. & Sav. 산갈퀴 |  | ○ |  |  |  |  |  |  |
| Rubiaceae | *Galium spurium* L. 갈퀴덩굴 |  | ○ |  |  |  |  |  |  |
| Rubiaceae | *Galium tricornutum* Dandy 민둥갈퀴덩굴 | ○ | ○ |  |  |  |  | ○ |  |
| Rubiaceae | *Galium trifloriforme* Kom. 개선갈퀴 |  |  |  |  |  |  |  |  |
| Rubiaceae | *Rubia argyi* (H.Lév. & Vaniot) H.Hara ex Lauener 꼭두서니 |  | ○ |  |  |  |  |  |  |
| Rubiaceae | *Rubia cordifolia* L. 갈퀴꼭두서니 | ○ | ○ |  |  |  |  |  |  |
| Convolvulaceae | *Calystegia pubescens* Lindl. 메꽃 |  |  |  |  |  |  |  |  |
| Boraginaceae | *Brachybotrys paridiformis* Maxim. ex Oliv. 당개지치 | ○ | ○ |  |  | ○ | Ⅲ |  |  |
| Boraginaceae | *Lithospermum erythrorhizon* Siebold & Zucc. 지치 |  | ○ | LC |  | ○ |  |  | CCP |
| Boraginaceae | *Trigonotis peduncularis* (Trevis.) Benth. ex Baker & S. Moore 꽃마리 |  | ○ |  |  |  |  |  |  |
| Verbenaceae | *Callicarpa japonica* Thunb. 작살나무 |  | ○ |  |  |  |  |  |  |
| Lamiaceae | *Clinopodium chinense* (Benth.) Kuntze var. *shibetchens*e (H. Lév.) Koidz. 산층층이 |  | ○ |  |  |  |  |  |  |
| Lamiaceae | *Isodon inflexus* (Thunb.) Kudô 산박하 |  | ○ |  |  |  |  |  |  |
| Lamiaceae | *Isodon serra* (Maxim.) Kudô 자주방아풀 |  |  |  |  |  |  |  |  |
| Lamiaceae | *Leonurus japonicus* Houtt. 익모초 |  | ○ |  |  |  |  |  |  |
| Lamiaceae | *Meehania urticifolia* (Miq.) Makino 벌깨덩굴 | ○ |  |  |  |  |  |  |  |
| Lamiaceae | *Prunella vulgaris* L. subsp. *asiatica* (Nakai) H. Hara 꿀풀 |  | ○ |  |  |  |  |  |  |
| Lamiaceae | *Scutellaria indica* L. 골무꽃 |  |  |  |  |  |  |  |  |
| Phrymaceae | *Phryma leptostachya* L. var. *oblongifolia* (Koidz.) Honda 파리풀 |  | ○ |  |  |  |  |  |  |
| Plantaginaceae | *Plantago asiatica* L. 질경이 |  |  |  |  |  |  |  |  |
| Caprifoliaceae | *Lonicera praeflorens* Batalin 올괴불나무 | ○ | ○ |  |  | ○ |  |  |  |
| Caprifoliaceae | *Sambucus racemosa* L. subsp. *kamtschatica* (E.Wolf) Hultén 지렁쿠나무 |  | ○ |  |  |  |  |  |  |
| Caprifoliaceae | *Sambucus williamsii* Hance 딱총나무 |  | ○ |  |  | ○ |  |  |  |
| Caprifoliaceae | *Viburnum carlesii* Hemsl. 분꽃나무 | ○ | ○ |  |  |  |  |  |  |
| Caprifoliaceae | *Viburnum opulus* L. var. *calvescens* (Rehder) H. Hara 백당나무 |  | ○ |  |  |  | Ⅰ |  |  |
| Caprifoliaceae | *Weigela florida* (Bunge) A. DC. 붉은병꽃나무 | ○ | ○ |  |  |  | Ⅱ |  |  |
| Caprifoliaceae | *Weigela subsessilis* (Nakai) L.H. Bailey 병꽃나무 | ○ | ○ |  | ○ |  |  |  |  |
| Caprifoliaceae | *Zabelia biflora* (Turcz.) Makino 털댕강나무 | ○ | ○ |  |  |  | Ⅲ |  | CCP |
| Valerianaceae | *Patrinia rupestris* (Pall.) Juss. 돌마타리 |  | ○ |  |  | ○ | Ⅳ |  | CCP |
| Valerianaceae | *Patrinia scabiosifolia* Fisch. ex Trevir. 마타리 |  | ○ |  |  |  |  |  |  |
| Valerianaceae | *Patrinia villosa* (Thunb.) Juss. 뚝갈 |  |  |  |  |  |  |  |  |
| Valerianaceae | *Valeriana fauriei* Briq. 쥐오줌풀 | ○ |  |  |  |  |  |  |  |
| Campanulaceae | *Adenophora triphylla* (Thunb.) A. DC. var. *japonica* (Regel) H. Hara 잔대 |  |  |  |  |  |  |  |  |
| Campanulaceae | *Campanula punctata* Lam. 초롱꽃 |  | ○ |  |  | ○ | Ⅰ |  |  |
| Campanulaceae | *Codonopsis lanceolata* (Siebold & Zucc.) Benth. & Hook.f. ex Trautv. 더덕 |  | ○ |  |  |  |  |  |  |
| Asteraceae | *Achillea alpina* L. 톱풀 | ○ | ○ |  |  |  | Ⅱ |  |  |
| Asteraceae | *Ambrosia artemisiifolia* L. 돼지풀 |  | ○ |  |  |  |  | ○ |  |
| Asteraceae | *Artemisia capillaris* Thunb. 사철쑥 |  |  |  |  |  |  |  |  |
| Asteraceae | *Artemisia codonocephala* Diels 참쑥 |  | ○ |  |  |  |  |  |  |
| Asteraceae | *Artemisia indica* Willd. 쑥 |  | ○ |  |  |  |  |  |  |
| Asteraceae | *Artemisia keiskeana* Miq. 맑은대쑥 |  | ○ |  |  |  |  |  |  |
| Asteraceae | *Artemisia rubripes* Nakai 덤불쑥 |  | ○ |  |  | ○ | Ⅰ |  |  |
| Asteraceae | *Artemisia sacrorum* Ledeb. var. *iwayomogi* (Kitam.) M.S. Park & G.Y. Chung 더위지기 |  | ○ |  |  |  |  |  |  |
| Asteraceae | *Artemisia selengensis* Turcz. ex Besser 물쑥 |  | ○ |  |  |  |  |  |  |
| Asteraceae | *Artemisia stolonifera* (Maxim.) Kom. 넓은잎외잎쑥 |  |  |  |  |  |  |  |  |
| Asteraceae | *Aster ageratoides* Turcz. 까실쑥부쟁이 |  | ○ |  |  |  |  |  |  |
| Asteraceae | *Aster maackii* Regel 좀개미취 |  | ○ |  |  | ○ | Ⅲ |  | CCP |
| Asteraceae | *Aster scaber* Thunb. 참취 | ○ | ○ |  |  |  |  |  |  |
| Asteraceae | *Aster tataricus* L.f. 개미취 | ○ | ○ |  |  |  |  |  |  |
| Asteraceae | *Atractylodes ovata* (Thunb.) DC. 삽주 |  | ○ |  |  |  |  |  |  |
| Asteraceae | *Bidens frondosa* L. 미국가막사리 |  | ○ |  |  |  |  | ○ |  |
| Asteraceae | *Carduus crispus* L. 지느러미엉겅퀴 |  | ○ |  |  |  |  | ○ |  |
| Asteraceae | *Chrysanthemum boreale* (Makino) Makino 산국 |  | ○ |  |  |  |  |  |  |
| Asteraceae | *Cirsium japonicum* Fisch. ex DC. var. *maackii* (Maxim.) Matsum. 엉겅퀴 |  | ○ |  |  |  |  |  |  |
| Asteraceae | *Cirsium setidens* (Dunn) Nakai 고려엉겅퀴 | ○ | ○ |  | ○ |  | Ⅰ |  |  |
| Asteraceae | *Crepidiastrum denticulatum* (Houtt.) J.H. Pak & Kawano 이고들빼기 |  | ○ |  |  |  |  |  |  |
| Asteraceae | *Crepidiastrum sonchifolium* (Bunge) J.H. Pak & Kawano 고들빼기 |  | ○ |  |  |  |  |  |  |
| Asteraceae | *Dendranthema oreastrum* (Hance) Y. Ling 바위구절초 |  | ○ |  |  |  |  |  |  |
| Asteraceae | *Dendranthema zawadskii* (Herbich) Tzvelev 산구절초 | ○ | ○ |  |  | ○ |  |  |  |
| Asteraceae | *Erigeron annuus* (L.) Pers. 개망초 |  | ○ |  |  |  |  | ○ |  |
| Asteraceae | *Erigeron philadelphicus* L. 봄망초 |  | ○ |  |  |  |  | ○ |  |
| Asteraceae | *Eupatorium japonicum* Thunb. 등골나물 |  |  |  |  |  |  |  |  |
| Asteraceae | *Eupatorium tripartitum* (Makino) Murata & H.Koyama 향등골나물 |  |  |  |  |  |  |  |  |
| Asteraceae | *Hemisteptia lyrata* (Bunge) Fisch. & C.A.Mey 지칭개 |  | ○ |  |  |  |  |  |  |
| Asteraceae | *Lactuca indica* L. 왕고들빼기 |  | ○ |  |  |  |  |  |  |
| Asteraceae | *Lactuca triangulata* Maxim. 두메고들빼기 |  | ○ |  |  |  |  |  |  |
| Asteraceae | *Saussurea pulchella* (Fisch.) Fisch. ex Colla 각시취 |  | ○ |  |  | ○ |  |  |  |
| Asteraceae | *Stemmacantha uniflora* (L.) Dittrich 뻐꾹채 |  | ○ |  |  |  | Ⅰ |  |  |
| Asteraceae | *Symphyotrichum pilosum* (Willd.) G.L.Nesom 미국쑥부쟁이 |  | ○ |  |  |  |  | ○ |  |
| Asteraceae | *Syneilesis palmata* (Thunb.) Maxim. 우산나물 |  | ○ |  |  |  |  |  |  |
| Asteraceae | *Taraxacum officinale* F.H.Wigg. 서양민들레 | ○ | ○ |  |  |  |  | ○ |  |
| Liliaceae | *Convallaria keiskei* Miq. 은방울꽃 |  | ○ |  |  |  |  |  |  |
| Liliaceae | *Disporum smilacinum* A.Gray 애기나리 |  | ○ |  |  | ○ |  |  |  |
| Liliaceae | *Disporum uniflorum* Baker 윤판나물 |  | ○ |  |  |  |  |  |  |
| Liliaceae | *Hemerocallis hakuunensis* Nakai 백운산원추리 |  | ○ |  | ○ |  |  |  |  |
| Liliaceae | *Lilium amabile* Palib. 털중나리 |  | ○ |  |  |  |  |  |  |
| Liliaceae | *Paris verticillata* M. Bieb. 삿갓나물 |  | ○ |  |  | ○ |  |  |  |
| Liliaceae | *Polygonatum involucratum* (Franch. & Sav.) Maxim. 용둥굴레 | ○ | ○ |  |  | ○ |  |  |  |
| Liliaceae | *Polygonatum* *odoratum* (Mill.) Druce var. *pluriflorum* (Miq.) Ohwi 둥굴레 | ○ | ○ |  |  |  |  |  |  |
| Liliaceae | *Smilax china* L. 청미래덩굴 |  | ○ |  |  |  |  |  |  |
| Liliaceae | *Smilax nipponica* Miq. 선밀나물 |  | ○ |  |  |  |  |  |  |
| Liliaceae | *Smilax sieboldii* Miq. 청가시덩굴 |  | ○ |  |  |  |  |  |  |
| Liliaceae | *Veratrum maackii* Regel var. *japonicum* (Baker) Shimizu 여로 |  | ○ |  |  |  | Ⅲ |  |  |
| Liliaceae | *Veratrum oxysepalum* Turcz. 박새 |  | ○ |  |  | ○ | Ⅰ |  |  |
| Dioscoreaceae | *Dioscorea nipponica* Makino 부채마 |  | ○ |  |  |  |  |  |  |
| Dioscoreaceae | *Dioscorea quinquelobata* Thunb. 단풍마 |  | ○ |  |  |  |  |  |  |
| Poaceae | *Achnatherum pekinense* (Hance) Ohwi 나래새 |  |  |  |  |  |  |  |  |
| Poaceae | *Calamagrostis arundinacea* (L.) Roth 실새풀 |  | ○ |  |  |  |  |  |  |
| Poaceae | *Diarrhena fauriei* (Hack.) Ohwi 광릉용수염 |  | ○ |  |  | ○ |  |  |  |
| Poaceae | *Diarrhena mandshurica* Maxim. 껍질용수염 |  | ○ |  |  | ○ |  |  |  |
| Poaceae | *Elymus ciliaris* (Trin. ex Bunge) Tzvelev 속털개밀 |  | ○ |  |  |  |  |  |  |
| Poaceae | *Melica nutans* L. 왕쌀새 | ○ | ○ |  |  | ○ | Ⅱ |  |  |
| Poaceae | *Milium effusum* L. 나도겨이삭 |  | ○ |  |  |  |  |  |  |
| Poaceae | *Miscanthus sinensis* Andersson var. *purpurascens* (Andersson) Matsum. 억새 |  |  |  |  |  |  |  |  |
| Poaceae | *Phaenosperma globosum* Munro ex Benth. 산기장 |  | ○ |  |  |  | Ⅰ |  |  |
| Poaceae | *Phragmites australis* (Cav.) Trin. ex Steud. 갈대 |  | ○ |  |  |  |  |  |  |
| Poaceae | *Phragmites japonicus* Steud. 달뿌리풀 |  |  |  |  |  |  |  |  |
| Poaceae | *Poa viridula* Palib. 청포아풀 |  | ○ |  |  |  |  |  |  |
| Poaceae | *Spodiopogon cotulifer* (Thunb.) Hack. 기름새 |  | ○ |  |  |  |  |  |  |
| Poaceae | *Spodiopogon sibiricus* Trin. 큰기름새 |  | ○ |  |  | ○ |  |  |  |
| Araceae | *Arisaema amurense* Maxim. 둥근잎천남성 |  | ○ |  |  |  |  |  |  |
| Cyperaceae | *Carex breviculmis* R.Br. 청사초 |  | ○ |  |  |  |  |  |  |
| Cyperaceae | *Carex forficula* Franch. & Sav. 산뚝사초 |  |  |  |  |  |  |  |  |
| Cyperaceae | *Carex humilis* Leyss. var. *nana* (H. Lév. & Vaniot) Ohwi 가는잎그늘사초 | ○ | ○ |  |  |  |  |  |  |
| Cyperaceae | *Carex lanceolata* Boott 그늘사초 | ○ | ○ |  |  |  |  |  |  |
| Cyperaceae | *Carex mira* Kük. 꼬랑사초 | ○ | ○ |  |  |  |  |  |  |
| Cyperaceae | *Carex pediformis* C.A. Mey. 넓은잎그늘사초 | ○ |  |  |  | ○ |  |  |  |
| Cyperaceae | *Carex pediformis* C.A. Mey. var. *pedunculata* Maxim. 왕그늘사초 |  | ○ |  |  |  | Ⅱ |  |  |
| Cyperaceae | *Carex polyschoena* H. Lév. & Vaniot 가지청사초 | ○ | ○ |  |  |  |  |  |  |
| Cyperaceae | *Carex siderosticta* Hance 대사초 |  | ○ |  |  |  |  |  |  |
| Cyperaceae | *Carex ussuriensis* Kom. 싸라기사초 | ○ | ○ |  |  | ○ |  |  | SCP |
| Cyperaceae | *Trichophorum polygamum* D.C. Son & K.S. Chang 동강고랭이 |  | ○ |  | ○ |  | Ⅳ |  | CIP |

* In.: Internal, Ex.: External, A: Rare plants (CR: Critically endangered, EN: Endangered, VU: Vulnerable, LC: Least concern), B: Korean endemic plants, C: Northern lineage plants on Korean Peninsula, D: Floristic target plants, E: Alien plants, F: Calciphilous plants (CIP: Calciphilous Indicator Plant, SCP: Superlative Calciphilous Plant, CCP: Comparative Calciphilous Plants)
