# Supplementary material for: Floristic inventory and distribution characteristics of algific talus slopes in a specific area of forest biodiversity in South Korea
Source: Biodivers Data J. 2023 Dec 18;11:e113952. doi: 10.3897/BDJ.11.e113952 (PMC10838045; doi:10.3897/BDJ.11.e113952)
Supplement: Supplementary material 4 — List of rare plants and Red-list species in the algific talus slopes in South Korea [file bdj-11-e113952-s004.docx]

4. List of rare plants and Red-list species in the algific talus slopes in South Korea

| Family | Scientific name / Korean name | KNA | NIBR | Fre. |
| --- | --- | --- | --- | --- |
| Cystopteridaceae | *Cystopteris fragilis* (L.) Bernh. | CR | NT | 2 |
| Aspleniaceae | *Asplenium trichomanes* L. subsp. *quadrivalens* D.E. Mey. | CR | LC | 1 |
| Ranunculaceae | *Aconitum coreanum* (H. Lév.) Rapaics ★ | CR | VU | 1 |
| Paeoniaceae | *Paeonia obovata* Maxim. ★ | CR | EN | 1 |
| Ericaceae | *Vaccinium vitis-idaea* L. | CR | EN | 1 |
| Orchidaceae | *Cyrtosia septentrionalis* (Rchb.f.) Garay ★ | CR | NT | 1 |
| Orchidaceae | *Goodyera repens* (L.) R.Br. | CR | VU | 1 |
| Saxifragaceae | *Astilboides tabularis* (Hemsl.) Engl. ★ | EN | NT | 2 |
| Saxifragaceae | *Micranthes octopetala* (Nakai) Y.I. Kim & Y.D. Kim | EN |  | 1 |
| Hydrangeaceae | *Deutzia paniculata* Nakai | EN | NT | 2 |
| Rosaceae | *Prunus choreiana* Nakai ex H.T. Im | EN | NT | 2 |
| Rosaceae | *Rosa koreana* Kom. | EN | NT | 3 |
| Fabaceae | *Sophora koreensis* Nakai | EN | NT | 1 |
| Araliaceae | *Oplopanax elatus* (Nakai) Nakai | EN | VU | 2 |
| Oleaceae | *Forsythia saxatilis* (Nakai) Nakai | EN | NT | 2 |
| Lamiaceae | *Dracocephalum argunense* Fisch. ex Link | EN |  | 1 |
| Caprifoliaceae | *Zabelia tyaihyonii* (Nakai) Hisauti & H. Hara | EN | VU | 1 |
| Rosaceae | *Prunus × yedoensis* Matsum. |  | EN | 1 |
| Pteridaceae | *Adiantum pedatum* L. | VU |  | 2 |
| Athyriaceae | *Athyrium spinulosum* (Maxim.) Milde | VU | VU | 1 |
| Pinaceae | *Picea jezoensis* (Siebold & Zucc.) Carrière | VU | EN | 1 |
| Cupressaceae | *Thuja koraiensis* Nakai | VU | NT | 1 |
| Taxaceae | *Taxus cuspidata* Siebold & Zucc. | VU |  | 2 |
| Illiciaceae | *Illicium anisatum* L. | VU | LC | 1 |
| Ranunculaceae | *Aconitum austrokoreense* Koidz. ★ | VU | NT | 1 |
| Ranunculaceae | *Actaea bifida* (Nakai) J. Compton | VU |  | 3 |
| Paeoniaceae | *Paeonia japonica* (Makino) Miyabe & Takeda | VU | LC | 9 |
| Rhamnaceae | *Berchemia berchemiifolia* (Makino) Koidz. | VU | LC | 2 |
| Apocynaceae | *Tylophora floribunda* Miq. | VU | LC | 1 |
| Asteraceae | *Scorzonera albicaulis* Bunge | VU |  | 1 |
| Liliaceae | *Lilium callosum* Siebold & Zucc. | VU | LC | 1 |
| Orchidaceae | *Calanthe discolor* Lindl. | VU | LC | 1 |
| Orchidaceae | *Gastrodia elata* Blume | VU | LC | 1 |
| Rosaceae | *Spiraea chartacea* Nakai |  | VU | 5 |
| Ranunculaceae | *Aconitum barbatum* Patrin ex Pers. |  | NT | 1 |
| Paeoniaceae | *Paeonia lactiflora* Pall. |  | NT | 2 |
| Orchidaceae | *Goodyera henryi* Rolfe |  | NT | 1 |
| Lycopodiaceae | *Lycopodium annotinum* L. | LC | NT | 1 |
| Selaginellaceae | *Selaginella helvetica* (L.) Spring | LC | LC | 1 |
| Ulmaceae | *Celtis choseniana* Nakai | LC | DD | 1 |
| Ranunculaceae | *Eranthis stellata* Maxim. | LC |  | 1 |
| Aristolochiaceae | *Aristolochia contorta* Bunge | LC |  | 3 |
| Aristolochiaceae | *Aristolochia manshuriensis* Kom. | LC |  | 4 |
| Aristolochiaceae | *Asarum maculatum* Nakai | LC | LC | 1 |
| Saxifragaceae | *Rodgersia podophylla* A. Gray | LC | LC | 5 |
| Rosaceae | *Exochorda serratifolia* S. Moore | LC | LC | 1 |
| Rosaceae | *Potentilla discolor* Bunge | LC | LC | 1 |
| Violaceae | *Viola albida* Palib. | LC |  | 3 |
| Ericaceae | *Rhododendron brachycarpum* D. Don ex G. Don | LC | LC | 1 |
| Oleaceae | *Syringa villosa* Vahl subsp. *wolfii* (C.K. Schneid.) Y. Chen & D.Y. Hong | LC |  | 3 |
| Gentianaceae | *Gentiana triflora* Pall. var. *japonica* (Kusn.) H. Hara | LC |  | 2 |
| Boraginaceae | *Lithospermum erythrorhizon* Siebold & Zucc. | LC | LC | 2 |
| Asteraceae | *Tephroseris flammea* (Turcz. ex DC.) Holub | LC |  | 1 |
| Liliaceae | *Lilium distichum* Nakai ex Kamib. | LC | LC | 1 |
| Liliaceae | *Trillium camschatcense* Ker Gawl. | LC | LC | 1 |
| Araceae | *Arisaema heterophyllum* Blume | LC |  | 4 |
| Orchidaceae | *Goodyera schlechtendaliana* Rchb.f. | LC | LC | 1 |
| Polypodiaceae | *Polypodium sibiricum* Sipliv. |  | LC | 3 |
| Ranunculaceae | *Thalictrum ichangense* Lecoy. ex Oliv. |  | LC | 1 |
| Rosaceae | *Prunus sibirica* L. |  | LC | 1 |
| Oleaceae | *Fraxinus chiisanensis* Nakai |  | LC | 1 |
| Oleaceae | *Syringa reticulata* (Blume) H. Hara |  | LC | 9 |
| Potamogetonaceae | *Potamogeton oxyphyllus* Miq. |  | LC | 1 |
| Orchidaceae | *Cymbidium goeringii* (Rchb.f.) Rchb.f. |  | LC | 1 |
| Cystopteridaceae | *Gymnocarpium dryopteris* (L.) Newman | DD | VU | 2 |
| Araliaceae | *Eleutherococcus divaricatus* (Siebold & Zucc.) S.Y. Hu var. *chiisanensis* (Nakai) C.H. Kim & B.-Y. Sun | DD | LC | 1 |
| Scrophulariaceae | *Pseudolysimachion pyrethrinum* (Nakai) T. Yamaz. | DD |  | 1 |

*KNA: Korea National Arboretum (2008), NIBR: National Institute of Biological Resources (2021). Degree (CR: Critically Endangered, EN: Endangered species, VU: Vulnerable, NT: Near Threatened, LC: Least Concerned, DD: Data Deficient.), Fre.: Frequency, ★: endangered wildlife Ⅱ.
