# Supplementary material for: Floristic inventory and distribution characteristics of algific talus slopes in a specific area of forest biodiversity in South Korea
Source: Biodivers Data J. 2023 Dec 18;11:e113952. doi: 10.3897/BDJ.11.e113952 (PMC10838045; doi:10.3897/BDJ.11.e113952)
Supplement: Supplementary material 5 — List of endemic plants on the Korean peninsula in the algific talus slopes of South Korea [file bdj-11-e113952-s005.docx]

5. List of endemic plants og the Korean peninsula in the algific talus slopes of South Korea.

| Family | Scientific name / Korean name | KNA | NIBR | Fre. |
| --- | --- | --- | --- | --- |
| Salicaceae | *Populus × tomentiglandulosa* T.B. Lee | ○ |  | 2 |
| Salicaceae | *Salix koriyanagi* Kimura ex Goerz | ○ | ○ | 3 |
| Ulmaceae | *Celtis choseniana* Nakai | ○ | ○ | 1 |
| Moraceae | *Broussonetia × hanjiana* M. Kim | ○ |  | 2 |
| Caryophyllaceae | *Pseudostellaria coreana* (Nakai) Ohwi | ○ |  | 1 |
| Ranunculaceae | *Aconitum austrokoreense* Koidz. | ○ | ○ | 1 |
| Ranunculaceae | *Aconitum pseudolaeve* Nakai | ○ | ○ | 3 |
| Ranunculaceae | *Actaea bifida* (Nakai) J. Compton | ○ | ○ | 3 |
| Ranunculaceae | *Clematis brachyura* Maxim. | ○ | ○ | 3 |
| Ranunculaceae | *Clematis trichotoma* Nakai | ○ | ○ | 9 |
| Ranunculaceae | *Clematis urticifolia* Nakai ex Kitag. | ○ |  | 9 |
| Ranunculaceae | *Thalictrum actaeifolium* Siebold & Zucc. var. *brevistylum* Nakai | ○ | ○ | 4 |
| Berberidaceae | *Berberis koreana* Palib. | ○ | ○ | 2 |
| Aristolochiaceae | *Asarum chungbuensis* (C.S. Yook & J.G. Kim) B.U. Oh | ○ | ○ | 3 |
| Aristolochiaceae | *Asarum maculatum* Nakai | ○ | ○ | 1 |
| Aristolochiaceae | *Asarum mandshuricum* (Maxim.) M.Kim & S. So var. *seoulense* (Nakai) M. Kim & S.So | ○ |  | 6 |
| Aristolochiaceae | *Asarum misandrum* B.U. Oh & J.G. Kim | ○ |  | 1 |
| Theaceae | *Stewartia koreana* Nakai ex Rehder | ○ | ○ | 3 |
| Papaveraceae | *Corydalis alata* B.U. Oh & W.R. Lee | ○ | ○ | 1 |
| Papaveraceae | *Corydalis maculata* B.U. Oh & Y.S. Kim | ○ | ○ | 2 |
| Papaveraceae | *Corydalis namdoensis* B.U. Oh & J.G. Kim | ○ | ○ | 1 |
| Saxifragaceae | *Micranthes octopetala* (Nakai) Y.I. Kim & Y.D. Kim | ○ |  | 1 |
| Hydrangeaceae | *Deutzia paniculata* Nakai | ○ | ○ | 2 |
| Rosaceae | *Prunus choreiana* Nakai ex H.T. Im | ○ |  | 2 |
| Fabaceae | *Indigofera grandiflora* B.H. Choi & S.K. Cho | ○ | ○ | 1 |
| Fabaceae | *Lespedeza maritima* Nakai | ○ | ○ | 1 |
| Fabaceae | *Sophora koreensis* Nakai | ○ | ○ | 1 |
| Fabaceae | *Vicia chosenensis* Ohwi | ○ | ○ | 2 |
| Geraniaceae | *Geranium koreanum* Kom. | ○ | ○ | 2 |
| Araliaceae | *Eleutherococcus divaricatus* (Siebold & Zucc.) S.Y. Hu var. *chiisanensis* (Nakai) C.H. Kim & B.-Y. Sun | ○ |  | 1 |
| Apiaceae | *Angelica reflexa* B.Y. Lee | ○ | ○ | 4 |
| Apiaceae | *Sillaphyton podagraria* (H. Boissieu) Pimenov | ○ | ○ | 1 |
| Ericaceae | *Vaccinium hirtum* Thunb. var. *koreanum* (Nakai) Kitam. |  | ○ | 7 |
| Ericaceae | *Rhododendron yedoense* Maxim. f. *poukhanense* (H. Lév.) Sugim. ex T. Yamaz. | ○ |  | 4 |
| Oleaceae | *Forsythia saxatilis* (Nakai) Nakai | ○ | ○ | 2 |
| Oleaceae | *Fraxinus chiisanensis* Nakai | ○ | ○ | 1 |
| Oleaceae | *Syringa fauriei* H. Lév. | ○ |  | 1 |
| Rubiaceae | *Asperula lasiantha* Nakai | ○ | ○ | 5 |
| Scrophulariaceae | *Melampyrum setaceum* (Maxim. ex Palib.) Nakai var. *nakaianum* (Tuyama) T. Yamaz. | ○ | ○ | 2 |
| Scrophulariaceae | *Paulownia coreana* Uyeki | ○ | ○ | 2 |
| Scrophulariaceae | *Pseudolysimachion pyrethrinum* (Nakai) T. Yamaz. | ○ |  | 1 |
| Caprifoliaceae | *Lonicera subsessilis* Rehder | ○ | ○ | 6 |
| Caprifoliaceae | *Weigela subsessilis* (Nakai) L.H. Bailey | ○ | ○ | 11 |
| Caprifoliaceae | *Zabelia tyaihyonii* (Nakai) Hisauti & H. Hara | ○ |  | 1 |
| Asteraceae | *Artemisia angustissima* Nakai |  | ○ | 1 |
| Asteraceae | *Aster koraiensis* Nakai | ○ | ○ | 1 |
| Asteraceae | *Cirsium setidens* (Dunn) Nakai | ○ | ○ | 2 |
| Asteraceae | *Saussurea macrolepis* (Nakai) Kitam. | ○ | ○ | 2 |
| Asteraceae | *Saussurea seoulensis* Nakai | ○ | ○ | 1 |
| Liliaceae | *Heloniopsis koreana* Fuse, N.S. Lee & M.N..Tamura | ○ | ○ | 3 |
| Liliaceae | *Hemerocallis hakuunensis* Nakai | ○ | ○ | 7 |
| Liliaceae | *Hosta minor* (Baker) Nakai | ○ | ○ | 2 |
| Liliaceae | *Lilium amabile* Palib. |  | ○ | 5 |
| Poaceae | *Sasa quelpaertensis* Nakai | ○ | ○ | 1 |
| Araceae | *Arisaema thunbergii* Blume |  | ○ | 1 |
| Cyperaceae | *Carex fusanensis* Ohwi | ○ |  | 3 |
| Cyperaceae | *Carex okamotoi* Ohwi | ○ | ○ | 5 |
| Cyperaceae | *Trichophorum polygamum* D.C. Son & K.S. Chang | ○ | ○ | 1 |

*KNA: Korea National Arboretum (2022), NIBR: National Institute of Biological Resources (2020). Fre.: Frequency.
