# Supplementary material for: Floristic inventory and distribution characteristics of algific talus slopes in a specific area of forest biodiversity in South Korea
Source: Biodivers Data J. 2023 Dec 18;11:e113952. doi: 10.3897/BDJ.11.e113952 (PMC10838045; doi:10.3897/BDJ.11.e113952)
Supplement: Supplementary material 6 — List of the Ⅴ–Ⅰ degree taxa of Korean floristic target species on the algific talus slope in South Korea [file bdj-11-e113952-s006.docx]

6. List of the Ⅴ–Ⅰ degree taxa of Korean floristic target species on the algific talus slope in South Korea.

| **Degree** | **Family name** | **Scientific name / Korean name** | **IUCN** | **Fre.** |
| --- | --- | --- | --- | --- |
| Ⅴ | Cupressaceae | *Thuja koraiensis* Nakai | NT | 1 |
|  | Ranunculaceae | *Aconitum austrokoreense* Koidz. | NT | 1 |
|  | Ranunculaceae | *Aconitum coreanum* (H. Lév.) Rapaics | VU | 1 |
|  | Paeoniaceae | *Paeonia obovata* Maxim. | EN | 1 |
|  | Saxifragaceae | *Astilboides tabularis* (Hemsl.) Engl. | NT | 2 |
|  | Fabaceae | *Sophora koreensis* Nakai | NT | 1 |
|  | Araliaceae | *Oplopanax elatus* (Nakai) Nakai | VU | 2 |
|  | Ericaceae | *Vaccinium vitis-idaea* L. | EN | 1 |
|  | Rubiaceae | *Asperula lasiantha* Nakai | · | 5 |
|  | Asteraceae | *Tephroseris flammea* (Turcz. ex DC.) Holub | · | 1 |
|  | Orchidaceae | *Cyrtosia septentrionalis* (Rchb.f.) Garay | NT | 1 |
|  | Orchidaceae | *Goodyera repens* (L.) R.Br. | VU | 1 |
| Ⅳ | Lycopodiaceae | *Lycopodium annotinum* L. | NT | 1 |
|  | Dennstaedtiaceae | *Microlepia strigosa* (Thunb.) C.Presl |  | 1 |
|  | Cystopteridaceae | *Cystopteris fragilis* (L.) Bernh. | NT | 2 |
|  | Aspleniaceae | *Asplenium pekinense* Hance |  | 1 |
|  | Aspleniaceae | *Asplenium trichomanes* L. subsp. *quadrivalens* D.E. Mey. | LC | 1 |
|  | Woodsiaceae | *Woodsia macrochlaena* Mett. ex Kuhn |  | 3 |
|  | Athyriaceae | *Athyrium iseanum* Rosenst. |  | 1 |
|  | Polypodiaceae | *Polypodium sibiricum* Sipliv. | LC | 3 |
|  | Ulmaceae | *Ulmus macrocarpa* Hance |  | 5 |
|  | Moraceae | *Morus mongolica* (Bureau) C.K. Schneid. |  | 1 |
|  | Urticaceae | *Laportea cuspidata* (Wedd.) Friis |  | 2 |
|  | Ranunculaceae | *Actaea bifida* (Nakai) J. Compton |  | 3 |
|  | Ranunculaceae | *Anemone reflexa* Steph. ex Willd. |  | 3 |
|  | Ranunculaceae | *Clematis serratifolia* Rehder |  | 1 |
|  | Ranunculaceae | *Thalictrum ichangense* Lecoy. ex Oliv. | LC | 1 |
|  | Berberidaceae | *Berberis koreana* Palib. |  | 2 |
|  | Papaveraceae | *Corydalis alata* B.U. Oh & W.R.Lee |  | 1 |
|  | Crassulaceae | *Phedimus middendorffianus* (Maxim.) 't Hart |  | 1 |
|  | Saxifragaceae | *Micranthes octopetala* (Nakai) Y.I. Kim & Y.D. Kim |  | 1 |
|  | Saxifragaceae | *Rodgersia podophylla* A. Gray | LC | 5 |
|  | Hydrangeaceae | *Deutzia grandiflora* Bunge var. *baroniana* (Diels) Rehder |  | 4 |
|  | Hydrangeaceae | *Deutzia paniculata* Nakai | NT | 2 |
|  | Hydrangeaceae | *Schizophragma hydrangeoides* Siebold & Zucc. |  | 1 |
|  | Rosaceae | *Prunus buergeriana* Miq. |  | 1 |
|  | Rosaceae | *Prunus × yedoensis* Matsum. | EN | 1 |
|  | Rosaceae | *Rosa davurica* Pall. |  | 1 |
|  | Rosaceae | *Rosa koreana* Kom. | NT | 3 |
|  | Rosaceae | *Spiraea trichocarpa* Nakai |  | 2 |
|  | Fabaceae | *Caragana fruticosa* (Pall.) Besser |  | 2 |
|  | Fabaceae | *Wisteria floribunda* (Willd.) DC. |  | 1 |
|  | Aceraceae | *Acer tegmentosum* Maxim. |  | 1 |
|  | Rhamnaceae | *Berchemia berchemiifolia* (Makino) Koidz. | LC | 2 |
|  | Rhamnaceae | *Rhamnus davurica* Pall. |  | 3 |
|  | Rhamnaceae | *Ziziphus jujuba* Mill. |  | 1 |
|  | Apiaceae | *Sillaphyton podagraria* (H. Boissieu) Pimenov |  | 1 |
|  | Oleaceae | *Forsythia saxatilis* (Nakai) Nakai | NT | 2 |
|  | Oleaceae | *Syringa fauriei* H. Lév. |  | 1 |
|  | Oleaceae | *Syringa villosa* Vahl subsp. *wolfii* (C.K. Schneid.) Y. Chen & D.Y.Hong |  | 3 |
|  | Scrophulariaceae | *Mazus stachydifolius* (Turcz.) Maxim. |  | 2 |
|  | Acanthaceae | *Strobilanthes oliganthus* Miq. |  | 1 |
|  | Caprifoliaceae | *Lonicera chrysantha* Turcz. ex Ledeb. |  | 1 |
|  | Valerianaceae | *Patrinia rupestris* (Pall.) Juss. |  | 2 |
|  | Campanulaceae | *Peracarpa carnosa* (Wall.) Hook. f. & Thomson |  | 1 |
|  | Asteraceae | *Rhynchospermum verticillatum* Reinw. |  | 1 |
|  | Liliaceae | *Trillium camschatcense* Ker Gawl. | LC | 1 |
|  | Poaceae | *Festuca rubra* L. |  | 1 |
|  | Poaceae | *Poa nemoralis* L. |  | 2 |
|  | Cyperaceae | *Trichophorum polygamum* D.C. Son & K.S. Chang |  | 1 |
| Ⅲ | Lycopodiaceae | *Lycopodium obscurum* L. |  | 1 |
|  | Selaginellaceae | *Selaginella tamariscina* (P. Beauv.) Spring |  | 3 |
|  | Cystopteridaceae | *Gymnocarpium dryopteris* (L.) Newman | VU | 2 |
|  | Pteridaceae | *Coniogramme japonica* (Thunb.) Diels |  | 1 |
|  | Athyriaceae | *Diplazium sibiricum* (Turcz. ex Kunze) Sa. Kurata |  | 2 |
|  | Dryopteridaceae | *Arachniodes standishii* (T. Moore) Ohwi |  | 1 |
|  | Dryopteridaceae | *Dryopteris fragrans* (L.) Schott |  | 9 |
|  | Polypodiaceae | *Lepisorus onoei* (Franch. & Sav.) Ching |  | 1 |
|  | Pinaceae | *Abies nephrolepis* (Trautv. ex Maxim.) Maxim. |  | 2 |
|  | Pinaceae | *Picea jezoensis* (Siebold & Zucc.) Carrière | EN | 1 |
|  | Salicaceae | *Salix rorida* Laksch. |  | 1 |
|  | Salicaceae | *Salix xerophila* Flod. |  | 1 |
|  | Betulaceae | *Betula chinensis* Maxim. |  | 5 |
|  | Betulaceae | *Betula costata* Trautv. |  | 2 |
|  | Betulaceae | *Betula davurica* Pall. |  | 10 |
|  | Betulaceae | *Betula schmidtii* Regel |  | 11 |
|  | Ulmaceae | *Celtis koraiensis* Nakai |  | 5 |
|  | Ulmaceae | *Ulmus laciniata* (Trautv.) Mayr |  | 3 |
|  | Moraceae | *Ficus erecta* Thunb. |  | 1 |
|  | Urticaceae | *Urtica angustifolia* Fisch. ex Hornem. |  | 7 |
|  | Urticaceae | *Urtica thunbergiana* Siebold & Zucc. |  | 3 |
|  | Schisandraceae | *Kadsura japonica* (L.) Dunal |  | 1 |
|  | Illiciaceae | *Illicium anisatum* L. | LC | 1 |
|  | Lauraceae | *Actinodaphne lancifolia* (Blume) Meisn. |  | 1 |
|  | Lauraceae | *Machilus japonica* Siebold & Zucc |  | 1 |
|  | Lauraceae | *Neolitsea aciculata* (Blume) Koidz. |  | 1 |
|  | Ranunculaceae | *Actaea asiatica* H. Hara |  | 6 |
|  | Ranunculaceae | *Clematis brachyura* Maxim. |  | 3 |
|  | Ranunculaceae | *Clematis fusca* Turcz. |  | 1 |
|  | Ranunculaceae | *Clematis fusca* Turcz. var. *violacea* Maxim. |  | 2 |
|  | Ranunculaceae | *Clematis urticifolia* Nakai ex Kitag. |  | 9 |
|  | Ranunculaceae | *Enemion raddeanum* Regel |  | 1 |
|  | Ranunculaceae | *Eranthis stellata* Maxim. |  | 1 |
|  | Lardizabalaceae | *Stauntonia hexaphylla* (Thunb.) Decne. |  | 1 |
|  | Aristolochiaceae | *Asarum maculatum* Nakai | LC | 1 |
|  | Aristolochiaceae | *Asarum misandrum* B.U. Oh & J.G. Kim |  | 1 |
|  | Actinidiaceae | *Actinidia kolomikta* (Maxim. & Rupr.) Maxim. |  | 8 |
|  | Theaceae | *Stewartia koreana* Nakai ex Rehder |  | 3 |
|  | Papaveraceae | *Corydalis maculata* B.U. Oh & Y.S. Kim |  | 2 |
|  | Papaveraceae | *Corydalis namdoensis* B.U. Oh & J.G. Kim |  | 1 |
|  | Papaveraceae | *Dicentra spectabilis* (L.) Lem. |  | 1 |
|  | Brassicaceae | *Cardamine komarovii* Nakai |  | 1 |
|  | Brassicaceae | *Catolobus pendulus* (L.) Al-Shehbaz |  | 2 |
|  | Hydrangeaceae | *Hydrangea petiolaris* Siebold & Zucc. |  | 1 |
|  | Hydrangeaceae | *Philadelphus schrenkii* Rupr. |  | 11 |
|  | Grossulariaceae | *Ribes mandshuricum* (Maxim.) Kom. |  | 6 |
|  | Grossulariaceae | *Ribes maximowiczianum* Kom. |  | 2 |
|  | Rosaceae | *Aruncus dioicus* (Walter) Fernald |  | 2 |
|  | Rosaceae | *Exochorda serratifolia* S. Moore | LC | 1 |
|  | Rosaceae | *Potentilla cryptotaeniae* Maxim. |  | 1 |
|  | Rosaceae | *Prunus mandshurica* (Maxim.) Koehne |  | 3 |
|  | Rosaceae | *Prunus sargentii* Rehder |  | 7 |
|  | Rosaceae | *Rhaphiolepis indica* (L.) Lindl. ex Ker var. *umbellata* (Thunb. ex Murray) H. Ohashi |  | 1 |
|  | Rosaceae | *Sanguisorba hakusanensis* Makino |  | 1 |
|  | Rosaceae | *Sorbaria sorbifolia* (L.) A. Braun var. *stellipila* Maxim. |  | 9 |
|  | Rosaceae | *Spiraea chamaedryfolia* L. |  | 7 |
|  | Rosaceae | *Spiraea chinensis* Maxim. |  | 6 |
|  | Rosaceae | *Spiraea fritschiana* C.K. Schneid. |  | 1 |
|  | Fabaceae | *Indigofera pseudotinctoria* Matsum. |  | 2 |
|  | Fabaceae | *Lespedeza juncea* (L.f.) Pers. |  | 1 |
|  | Fabaceae | *Vicia chosenensis* Ohwi |  | 2 |
|  | Oxalidaceae | *Oxalis acetosella* L. |  | 1 |
|  | Euphorbiaceae | *Mercurialis leiocarpa* Siebold & Zucc. |  | 1 |
|  | Rutaceae | *Poncirus trifoliata* (L.) Raf. |  | 1 |
|  | Aceraceae | *Acer barbinerve* Maxim. |  | 2 |
|  | Aceraceae | *Acer komarovii* Pojark. |  | 2 |
|  | Aceraceae | *Acer palmatum* Thunb. |  | 4 |
|  | Aceraceae | *Acer triflorum* Kom. |  | 6 |
|  | Aceraceae | *Acer ukurunduense* Trautv. & C.A. Mey. |  | 3 |
|  | Rhamnaceae | *Rhamnus ussuriensis* J.J. Vassil. |  | 3 |
|  | Vitaceae | *Vitis coignetiae* Pulliat ex Planch. |  | 3 |
|  | Tiliaceae | *Corchoropsis tomentosa* (Thunb.) Makino var. *psilocarpa* (Harms & Loes. ex Gilg & Loes.) C.Y. Wu & Y. Tang |  | 2 |
|  | Violaceae | *Viola ovato*-oblonga (Miq.) Makino |  | 1 |
|  | Violaceae | *Viola violacea* Makino |  | 1 |
|  | Cornaceae | *Aucuba japonica* Thunb. |  | 1 |
|  | Araliaceae | *Eleutherococcus divaricatus* (Siebold & Zucc.) S.Y. Hu var. *chiisanensis* (Nakai) C.H. Kim & B.-Y. Sun | LC | 1 |
|  | Apiaceae | *Cnidium monnieri* (L.) Cusson |  | 1 |
|  | Ericaceae | *Rhododendron brachycarpum* D. Don ex G. Don | LC | 1 |
|  | Ericaceae | *Vaccinium bracteatum* Thunb. |  | 1 |
|  | Ericaceae | *Vaccinium hirtum* Thunb. var. *koreanum* (Nakai) Kitam. |  | 7 |
|  | Oleaceae | *Fraxinus chiisanensis* Nakai | LC | 1 |
|  | Oleaceae | *Ligustrum ovalifolium* Hassk. |  | 1 |
|  | Oleaceae | *Syringa reticulata* (Blume) H. Hara | LC | 9 |
|  | Rubiaceae | *Galium odoratum* (L.) Scop. |  | 2 |
|  | Boraginaceae | *Brachybotrys paridiformis* Maxim. ex Oliv. |  | 4 |
|  | Verbenaceae | *Callicarpa dichotoma* (Lour.) Raeusch. ex K. Koch |  | 2 |
|  | Lamiaceae | *Dracocephalum argunense* Fisch. ex Link |  | 1 |
|  | Scrophulariaceae | *Melampyrum setaceum* (Maxim. ex Palib.) Nakai var. *nakaianum* (Tuyama) T. Yamaz. |  | 2 |
|  | Scrophulariaceae | *Pseudolysimachion dauricum* (Steven) Holub |  | 1 |
|  | Caprifoliaceae | *Zabelia biflora* (Turcz.) Makino |  | 2 |
|  | Asteraceae | *Aster maackii* Regel |  | 1 |
|  | Liliaceae | *Asparagus cochinchinensis* (Lour.) Merr. |  | 1 |
|  | Liliaceae | *Hosta clausa* Nakai |  | 1 |
|  | Liliaceae | *Lilium callosum* Siebold & Zucc. | LC | 1 |
|  | Liliaceae | *Lilium distichum* Nakai ex Kamib. | LC | 1 |
|  | Liliaceae | *Veratrum maackii* Regel var. *japonicum* (Baker) Shimizu |  | 3 |
|  | Poaceae | *Brachyelytrum japonicum* (Hack.) Matsum. ex Honda |  | 1 |
|  | Poaceae | *Melica scabrosa* Trin. |  | 1 |
|  | Araceae | *Arisaema thunbergii* Blume |  | 1 |
|  | Cyperaceae | *Carex filipes* Franch. & Sav. |  | 1 |
|  | Orchidaceae | *Calanthe discolor* Lindl. | LC | 1 |
|  | Orchidaceae | *Cephalanthera falcata* (Thunb.) Blume |  | 1 |
|  | Orchidaceae | *Goodyera henryi* Rolfe | NT | 1 |
| Ⅱ | Lycopodiaceae | *Huperzia miyoshiana* (Makino) Ching |  | 2 |
|  | Selaginellaceae | *Selaginella helvetica* (L.) Spring | LC | 1 |
|  | Osmundaceae | *Osmunda cinnamomea* L. |  | 2 |
|  | Hymenophyllaceae | *Crepidomanes minutum* (Blume) K. Iwats. |  | 3 |
|  | Pteridaceae | *Adiantum pedatum* L. |  | 2 |
|  | Pteridaceae | *Coniogramme intermedia* Hieron. |  | 1 |
|  | Thelypteridaceae | *Phegopteris connectilis* (Michx.) D.Watt |  | 1 |
|  | Woodsiaceae | *Woodsia subcordata* Turcz. |  | 2 |
|  | Dryopteridaceae | *Dryopteris expansa* (C. Presl) Fraser-Jenk. & Jermy |  | 5 |
|  | Dryopteridaceae | *Polystichum braunii* (Spenn.) Fée |  | 5 |
|  | Polypodiaceae | *Pleurosoriopsis makinoi* (Maxim. ex Makino) Fomin |  | 1 |
|  | Polypodiaceae | *Pyrrosia petiolosa* (Christ) Ching |  | 1 |
|  | Pinaceae | *Pinus koraiensis* Siebold & Zucc. |  | 9 |
|  | Taxaceae | *Taxus cuspidata* Siebold & Zucc. |  | 2 |
|  | Betulaceae | *Alnus japonica* (Thunb.) Steud. |  | 2 |
|  | Betulaceae | *Betula ermanii* Cham. |  | 2 |
|  | Caryophyllaceae | *Lychnis cognata* Maxim. |  | 2 |
|  | Magnoliaceae | *Magnolia sieboldii* K. Koch |  | 13 |
|  | Schisandraceae | *Schisandra chinensis* (Turcz.) Baill. |  | 16 |
|  | Ranunculaceae | *Caltha palustris* L. |  | 1 |
|  | Berberidaceae | *Berberis amurensis* Rupr. |  | 2 |
|  | Aristolochiaceae | *Aristolochia manshuriensis* Kom. |  | 4 |
|  | Paeoniaceae | *Paeonia japonica* (Makino) Miyabe & Takeda | LC | 9 |
|  | Papaveraceae | *Hylomecon vernalis* Maxim. |  | 1 |
|  | Crassulaceae | *Hylotelephium viviparum* (Maxim.) H. Ohba |  | 3 |
|  | Saxifragaceae | *Mukdenia rossii* (Oliv.) Koidz. |  | 5 |
|  | Rosaceae | *Potentilla dickinsii* Franch. & Sav. |  | 6 |
|  | Rosaceae | *Rosa acicularis* Lindl. |  | 3 |
|  | Rosaceae | *Sorbus commixta* Hedl. |  | 5 |
|  | Rosaceae | *Spiraea salicifolia* L. |  | 3 |
|  | Geraniaceae | *Geranium koreanum* Kom. |  | 2 |
|  | Rutaceae | *Phellodendron amurense* Rupr. |  | 5 |
|  | Celastraceae | *Euonymus macropterus* Rupr. |  | 7 |
|  | Celastraceae | *Euonymus pauciflorus* Maxim. |  | 8 |
|  | Celastraceae | *Tripterygium regelii* Sprague & Takeda |  | 9 |
|  | Tiliaceae | *Tilia amurensis* Rupr. |  | 14 |
|  | Tiliaceae | *Tilia mandshurica* Rupr. & Maxim. |  | 6 |
|  | Tiliaceae | *Tilia taquetii* C.K. Schneid. |  | 2 |
|  | Violaceae | *Viola orientalis* (Maxim.) W. Becker |  | 5 |
|  | Violaceae | *Viola tokubuchiana* Makino var. *takedana* (Makino) F. Maek. |  | 1 |
|  | Onagraceae | *Circaea alpina* L. |  | 1 |
|  | Onagraceae | *Circaea lutetiana* L. subsp. *quadrisulcata* (Maxim.) Asch. & Magnus |  | 2 |
|  | Apiaceae | *Bupleurum longeradiatum* Turcz. |  | 1 |
|  | Primulaceae | *Primula jesoana* Miq. |  | 1 |
|  | Rubiaceae | *Rubia chinensis* Regel & Maack |  | 4 |
|  | Caprifoliaceae | *Lonicera subsessilis* Rehder |  | 6 |
|  | Caprifoliaceae | *Lonicera vidalii* Franch. & Sav. |  | 1 |
|  | Caprifoliaceae | *Weigela florida* (Bunge) A. DC. |  | 13 |
|  | Asteraceae | *Achillea alpina* L. |  | 2 |
|  | Asteraceae | *Ligularia fischeri* (Ledeb.) Turcz. |  | 2 |
|  | Liliaceae | *Heloniopsis koreana* Fuse, N.S. Lee & M.N. Tamura |  | 3 |
|  | Juncaceae | *Luzula multiflora* (Ehrh.) Lej. |  | 1 |
|  | Poaceae | *Melica nutans* L. |  | 9 |
|  | Poaceae | *Muhlenbergia hakonensis* (Hack. ex Matsum.) Makino |  | 2 |
|  | Poaceae | *Poa matsumurae* Hack. |  | 1 |
|  | Cyperaceae | *Carex onoei* Franch. & Sav. |  | 2 |
|  | Cyperaceae | *Carex pediformis* C.A. Mey. var. *pedunculata* Maxim. |  | 1 |
|  | Orchidaceae | *Gastrodia elata* Blume | LC | 1 |
| Ⅰ | Pteridaceae | *Cheilanthes argentea* (S.G. Gmel.) Kunze |  | 2 |
|  | Onocleaceae | *Onoclea interrupta* (Maxim.) Ching & P.C. Chiu |  | 2 |
|  | Dryopteridaceae | *Dryopteris erythrosora* (D.C. Eaton) Kuntze |  | 1 |
|  | Dryopteridaceae | *Dryopteris gymnophylla* (Baker) C.Chr. |  | 1 |
|  | Dryopteridaceae | *Polystichum ovato-paleaceum* (Kodama) Sa. Kurata var. *coraiense* (Christ) Sa. Kurata |  | 2 |
|  | Dryopteridaceae | *Polystichum polyblepharum* (Roem. ex Kunze) C. Presl |  | 1 |
|  | Polypodiaceae | *Lemmaphyllum microphyllum* C. Presl |  | 2 |
|  | Polypodiaceae | *Lepisorus thunbergianus* (Kaulf.) Ching |  | 3 |
|  | Cephalotaxaceae | *Cephalotaxus harringtonia* (Knight ex J. Forbes) K. Koch |  | 1 |
|  | Juglandaceae | *Juglans mandshurica* Maxim. |  | 8 |
|  | Betulaceae | *Carpinus tschonoskii* Maxim. |  | 2 |
|  | Betulaceae | *Carpinus turczaninovii* Hance |  | 3 |
|  | Ulmaceae | *Celtis biondii* Pamp. |  | 2 |
|  | Ulmaceae | *Celtis choseniana* Nakai | DD | 1 |
|  | Ulmaceae | *Hemiptelea davidii* (Hance) Planch. |  | 4 |
|  | Ulmaceae | *Ulmus davidiana* Planch. ex DC. var*. japonica* (Rehder) Nakai |  | 19 |
|  | Ulmaceae | *Ulmus parvifolia* Jacq. |  | 3 |
|  | Urticaceae | *Nanocnide japonica* Blume |  | 3 |
|  | Urticaceae | *Pilea japonica* (Maxim.) Hand.-Mazz. |  | 7 |
|  | Caryophyllaceae | *Silene baccifera* (L.) Roth |  | 3 |
|  | Lauraceae | *Lindera erythrocarpa* Makino |  | 8 |
|  | Lauraceae | *Lindera glauca* (Siebold & Zucc.) Blume |  | 4 |
|  | Lauraceae | *Neolitsea sericea* (Blume) Koidz. |  | 1 |
|  | Ranunculaceae | *Aconitum jaluense* Kom. |  | 4 |
|  | Ranunculaceae | *Aconitum longecassidatum* Nakai |  | 1 |
|  | Ranunculaceae | *Clematis patens* C. Morren & Decne. |  | 7 |
|  | Ranunculaceae | *Hepatica asiatica* Nakai |  | 6 |
|  | Ranunculaceae | *Semiaquilegia adoxoides* (DC.) Makino |  | 2 |
|  | Chloranthaceae | *Chloranthus japonicus* Siebold |  | 4 |
|  | Aristolochiaceae | *Aristolochia contorta* Bunge |  | 3 |
|  | Theaceae | *Camellia japonica* L. |  | 2 |
|  | Theaceae | *Eurya japonica* Thunb. |  | 2 |
|  | Papaveraceae | *Corydalis incisa* (Thunb.) Pers. |  | 1 |
|  | Saxifragaceae | *Chrysosplenium japonicum* (Maxim.) Makino |  | 1 |
|  | Hydrangeaceae | *Deutzia glabrata* Kom. |  | 12 |
|  | Hydrangeaceae | *Deutzia parviflora* Bunge |  | 16 |
|  | Hydrangeaceae | *Deutzia uniflora* Shirai |  | 9 |
|  | Rosaceae | *Filipendula glaberrima* Nakai |  | 1 |
|  | Rosaceae | *Malus baccata* (L.) Borkh. |  | 8 |
|  | Rosaceae | *Malus mandshurica* (Maxim.) Kom. ex Skvortsov |  | 1 |
|  | Rosaceae | *Pourthiaea villosa* (Thunb.) Decne. |  | 4 |
|  | Rosaceae | *Pyrus calleryana* Decne. var. *fauriei* (C.K. Schneid.) Rehder |  | 2 |
|  | Rosaceae | *Rosa lucieae* Franch. & Rochebr. ex Crép. |  | 2 |
|  | Rosaceae | *Rubus corchorifolius* L.f. |  | 2 |
|  | Rosaceae | *Spiraea blumei* G. Don |  | 7 |
|  | Rosaceae | *Spiraea chartacea* Nakai | VU | 5 |
|  | Fabaceae | *Lespedeza maritima* Nakai |  | 1 |
|  | Euphorbiaceae | *Mallotus japonicus* (L.f.) Müll.Arg. |  | 2 |
|  | Euphorbiaceae | *Neoshirakia japonica* (Siebold & Zucc.) Esser |  | 2 |
|  | Rutaceae | *Dictamnus dasycarpus* Turcz. |  | 3 |
|  | Rutaceae | *Orixa japonica* Thunb. |  | 1 |
|  | Anacardiaceae | *Toxicodendron sylvestre* (Siebold & Zucc.) Kuntze |  | 2 |
|  | Sabiaceae | *Meliosma pinnata* (Roxb.) Maxim. var. *oldhamii* (Miq. ex Maxim.) Beusekom |  | 1 |
|  | Balsaminaceae | *Impatiens noli-tangere* L. |  | 6 |
|  | Aquifoliaceae | *Ilex macropoda* Miq. |  | 5 |
|  | Celastraceae | *Celastrus stephanotiifolius* (Makino) Makino |  | 2 |
|  | Celastraceae | *Euonymus fortunei* (Turcz.) Hand.-Mazz. |  | 1 |
|  | Celastraceae | *Euonymus sachalinensis* (F. Schmidt) Maxim. |  | 4 |
|  | Staphyleaceae | *Euscaphis japonica* (Thunb.) Kanitz |  | 1 |
|  | Buxaceae | *Buxus sinica* (Rehder & E.H. Wilson) M. Cheng var. *insularis* (Nakai) M.Cheng |  | 3 |
|  | Rhamnaceae | *Hovenia dulcis* Thunb. |  | 3 |
|  | Tiliaceae | *Grewia biloba* G. Don |  | 1 |
|  | Elaeagnaceae | *Elaeagnus macrophylla* Thunb. |  | 2 |
|  | Onagraceae | *Circaea cordata* Royle |  | 1 |
|  | Araliaceae | *Eleutherococcus sessiliflorus* (Rupr. & Maxim.) S.Y. Hu |  | 6 |
|  | Araliaceae | *Hedera rhombea* (Miq.) Siebold & Zucc. ex Bean |  | 1 |
|  | Ericaceae | *Vaccinium oldhamii* Miq. |  | 1 |
|  | Myrsinaceae | *Ardisia japonica* (Thunb.) Blume |  | 1 |
|  | Oleaceae | *Fraxinus mandshurica* Rupr. |  | 4 |
|  | Oleaceae | *Syringa pubescens* Turcz. subsp. *patula* (Palib.) M.C. Chang & X.L.Chen |  | 9 |
|  | Apocynaceae | *Cynanchum ascyrifolium* (Franch. & Sav.) Matsum. |  | 1 |
|  | Apocynaceae | *Tylophora floribunda* Miq. | LC | 1 |
|  | Rubiaceae | *Paederia foetida* L. |  | 8 |
|  | Caprifoliaceae | *Lonicera maackii* (Rupr.) Maxim. |  | 6 |
|  | Caprifoliaceae | *Viburnum opulus* L. var. *calvescens* (Rehder) H. Hara |  | 6 |
|  | Campanulaceae | *Campanula punctata* Lam. |  | 5 |
|  | Asteraceae | *Artemisia rubripes* Nakai |  | 4 |
|  | Asteraceae | *Carpesium macrocephalum* Franch. & Sav. |  | 1 |
|  | Asteraceae | *Cirsium pendulum* Fisch. ex DC. |  | 1 |
|  | Asteraceae | *Cirsium setidens* (Dunn) Nakai |  | 2 |
|  | Asteraceae | *Saussurea odontolepis* (Herder) Sch.Bip. ex Maxim. |  | 1 |
|  | Asteraceae | *Stemmacantha uniflora* (L.) Dittrich |  | 3 |
|  | Liliaceae | *Asparagus oligoclonos* Maxim. |  | 3 |
|  | Liliaceae | *Hosta capitata* (Koidz.) Nakai |  | 2 |
|  | Liliaceae | *Hosta minor* (Baker) Nakai |  | 2 |
|  | Liliaceae | *Ophiopogon japonicus* (Thunb.) Ker Gawl. |  | 1 |
|  | Liliaceae | *Veratrum oxysepalum* Turcz. |  | 1 |
|  | Poaceae | *Hystrix longe-aristata* (Hack.) Honda |  | 1 |
|  | Poaceae | *Phaenosperma globosum* Munro ex Benth. |  | 3 |
|  | Araceae | *Arisaema heterophyllum* Blume |  | 4 |
|  | Araceae | *Arisaema ringens* (Thunb.) Schott |  | 1 |
|  | Cyperaceae | *Carex lenta* D. Don |  | 1 |
|  | Cyperaceae | *Carex okamotoi* Ohwi |  | 5 |
|  | Cyperaceae | *Carex tristachya* Thunb. |  | 1 |
|  | Orchidaceae | *Cymbidium goeringii* (Rchb.f.) Rchb.f. | LC | 1 |
|  | Orchidaceae | *Liparis suzumushi* Tsusumi, T. Yukawa & M. Kato |  | 1 |

Degree of IUCN (CR: Critically Endangered, EN: Endangered, VU: Vulnerable, NT: Near Threatened, LC: Least Concerned, DD: Data Deficient.), Fre.: Frequency
