# Supplementary material for: Floristic inventory and distribution characteristics of algific talus slopes in a specific area of forest biodiversity in South Korea
Source: Biodivers Data J. 2023 Dec 18;11:e113952. doi: 10.3897/BDJ.11.e113952 (PMC10838045; doi:10.3897/BDJ.11.e113952)
Supplement: Supplementary material 7 — List of the northern lineage plants on the Korean Peninsula and 300 species threatened by climate change in the algific talus slopes of South Korea [file bdj-11-e113952-s007.docx]

Table 7. List of the northern lineage plants on the Korean Peninsula and 300 species threatened by climate change in the algific talus slopes of South Korea.

| **Family name** | **Scientific name / Korean name** | **NLP** | **300** | **Fre.** |
| --- | --- | --- | --- | --- |
| Crassulaceae | *Phedimus aizoon* (L.) 't Hart | ○ |  | 2 |
| Urticaceae | *Urtica angustifolia* Fisch. ex Hornem. | ○ |  | 7 |
| Pinaceae | *Picea jezoensis* (Siebold & Zucc.) Carrière | ○ | N | 1 |
| Pteridaceae | *Coniogramme japonica* (Thunb.) Diels |  | S | 1 |
| Rosaceae | *Exochorda serratifolia* S. Moore | ○ |  | 1 |
| Caprifoliaceae | *Lonicera chrysantha* Turcz. ex Ledeb. | ○ | N | 1 |
| Asteraceae | *Saussurea pulchella* (Fisch.) Fisch. ex Colla | ○ |  | 2 |
| Rosaceae | *Spiraea trichocarpa* Nakai |  | N | 2 |
| Rhamnaceae | *Rhamnus davurica* Pall. | ○ |  | 3 |
| Fabaceae | *Vicia amoena* Fisch. ex Ser. | ○ |  | 3 |
| Ranunculaceae | *Ranunculus tachiroei* Franch. & Sav. | ○ |  | 1 |
| Ranunculaceae | *Clematis serratifolia* Rehder | ○ | N | 1 |
| Saxifragaceae | *Astilboides tabularis* (Hemsl.) Engl. |  | N | 2 |
| Rosaceae | *Prunus mandshurica* (Maxim.) Koehne | ○ |  | 3 |
| Apiaceae | *Bupleurum longeradiatum* Turcz. | ○ |  | 1 |
| Poaceae | *Beckmannia syzigachne* (Steud.) Fernald | ○ |  | 1 |
| Oleaceae | *Syringa reticulata* (Blume) H. Hara | ○ |  | 9 |
| Salicaceae | *Salix gracilistyla* Miq. | ○ |  | 2 |
| Aspleniaceae | *Asplenium ruprechtii* Sa. Kurata | ○ |  | 10 |
| Betulaceae | *Betula costata* Trautv. | ○ |  | 2 |
| Ranunculaceae | *Clematis fusca* Turcz. | ○ | N | 1 |
| Cyperaceae | *Carex accrescens* Ohwi | ○ |  | 1 |
| Clusiaceae | *Hypericum erectum* Thunb. | ○ |  | 3 |
| Gentianaceae | *Gentiana triflora* Pall. var. *japonica* (Kusn.) H. Hara | ○ | N | 2 |
| Dryopteridaceae | *Dryopteris crassirhizoma* Nakai | ○ |  | 18 |
| Poaceae | *Diarrhena fauriei* (Hack.) Ohwi | ○ |  | 5 |
| Cyperaceae | *Carex neurocarpa* Maxim. | ○ |  | 2 |
| Caprifoliaceae | *Lonicera maackii* (Rupr.) Maxim. | ○ |  | 6 |
| Apiaceae | *Peucedanum terebinthaceum* (Fisch. ex Trevir.) Fisch. ex Turcz. | ○ |  | 8 |
| Grossulariaceae | *Ribes mandshuricum* (Maxim.) Kom. | ○ |  | 6 |
| Poaceae | *Diarrhena mandshurica* Maxim. | ○ |  | 4 |
| Grossulariaceae | *Ribes komarovii* Pojark. | ○ | N | 1 |
| Rosaceae | *Spiraea salicifolia* L. | ○ | N | 3 |
| Oleaceae | *Syringa villosa* Vahl subsp. *wolfii* (C.K. Schneid.) Y. Chen & D.Y. Hong | ○ |  | 3 |
| Scrophulariaceae | *Melampyrum roseum* Maxim. | ○ |  | 13 |
| Ranunculaceae | *Thalictrum aquilegiifolium* L. var. *sibiricum* Regel & Tiling | ○ |  | 2 |
| Brassicaceae | *Barbarea orthoceras* Ledeb. | ○ |  | 4 |
| Urticaceae | *Nanocnide japonica* Blume |  | S | 3 |
| Ranunculaceae | *Enemion raddeanum* Regel | ○ |  | 2 |
| Scrophulariaceae | *Phtheirospermum japonicum* (Thunb.) Kanitz | ○ |  | 3 |
| Celastraceae | *Euonymus macropterus* Rupr. | ○ |  | 7 |
| Fabaceae | *Vicia unijuga* A. Braun | ○ |  | 6 |
| Ulmaceae | *Ulmus laciniata* (Trautv.) Mayr | ○ |  | 3 |
| Schisandraceae | *Kadsura japonica* (L.) Dunal |  | S | 1 |
| Ranunculaceae | *Eranthis stellata* Maxim. | ○ | N | 1 |
| Cyperaceae | *Carex pediformis* C.A. Mey. | ○ |  | 2 |
| Violaceae | *Viola orientalis* (Maxim.) W. Becker | ○ |  | 5 |
| Ranunculaceae | *Aconitum barbatum* Patrin ex Pers. |  | N | 1 |
| Ericaceae | *Pyrola japonica* Klenze ex Alef. | ○ |  | 12 |
| Papaveraceae | *Corydalis ochotensis* Turcz. | ○ |  | 3 |
| Cupressaceae | *Thuja koraiensis* Nakai |  | N | 1 |
| Brassicaceae | *Catolobus pendulus* (L.) Al-Shehbaz | ○ |  | 2 |
| Brassicaceae | *Cardamine komarovii* Nakai |  | N | 1 |
| Fabaceae | *Maackia amurensis* Rupr. | ○ |  | 13 |
| Rosaceae | *Rhaphiolepis indica* (L.) Lindl. ex Ker var. *umbellata* (Thunb. ex Murray) H. Ohashi |  | S | 1 |
| Boraginaceae | *Brachybotrys paridiformis* Maxim. ex Oliv. | ○ | N | 4 |
| Asteraceae | *Artemisia rubripes* Nakai | ○ | N | 4 |
| Saxifragaceae | *Rodgersia podophylla* A. Gray |  | N | 5 |
| Araliaceae | *Aralia cordata* Thunb. var. *continentalis* (Kitag.) Y.C. Chu | ○ |  | 4 |
| Saxifragaceae | *Mukdenia rossii* (Oliv.) Koidz. |  | N | 5 |
| Valerianaceae | *Patrinia rupestris* (Pall.) Juss. | ○ | N | 2 |
| Dennstaedtiaceae | *Microlepia strigosa* (Thunb.) C.Presl |  | S | 1 |
| Ranunculaceae | *Caltha palustris* L. | ○ |  | 1 |
| Caryophyllaceae | *Lychnis cognata* Maxim. | ○ |  | 2 |
| Oleaceae | *Fraxinus mandshurica* Rupr. | ○ |  | 4 |
| Aristolochiaceae | *Aristolochia manshuriensis* Kom. | ○ | N | 4 |
| Rosaceae | *Potentilla chinensis* Ser. | ○ |  | 3 |
| Caprifoliaceae | *Sambucus williamsii* Hance | ○ |  | 16 |
| Araliaceae | *Oplopanax elatus* (Nakai) Nakai | ○ | N | 2 |
| Ericaceae | *Rhododendron brachycarpum* D. Don ex G. Don | ○ |  | 1 |
| Aristolochiaceae | *Asarum mandshuricum* (Maxim.) M.Kim & S. So | ○ |  | 1 |
| Liliaceae | *Lilium distichum* Nakai ex Kamib. | ○ |  | 1 |
| Hydrangeaceae | *Deutzia parviflora* Bunge | ○ |  | 16 |
| Berberidaceae | *Berberis amurensis* Rupr. | ○ |  | 2 |
| Grossulariaceae | *Ribes maximowiczianum* Kom. | ○ |  | 2 |
| Ericaceae | *Vaccinium bracteatum* Thunb. |  | S | 1 |
| Violaceae | *Viola selkirkii* Pursh ex Goldie | ○ |  | 5 |
| Clusiaceae | *Hypericum ascyron* L. | ○ |  | 8 |
| Parnassiaceae | *Parnassia palustris* L. | ○ |  | 1 |
| Betulaceae | *Betula davurica* Pall. | ○ |  | 10 |
| Rosaceae | *Potentilla cryptotaeniae* Maxim. | ○ |  | 1 |
| Betulaceae | *Alnus incana* (L.) Moench subsp. *hirsuta* (Turcz. ex Spach) Á. Löve & D.Löve | ○ |  | 5 |
| Hydrangeaceae | *Deutzia glabrata* Kom. | ○ |  | 12 |
| Cyperaceae | *Carex onoei* Franch. & Sav. | ○ |  | 2 |
| Betulaceae | *Betula schmidtii* Regel | ○ |  | 11 |
| Liliaceae | *Veratrum oxysepalum* Turcz. | ○ |  | 1 |
| Acanthaceae | *Strobilanthes oliganthus* Miq. |  | S | 1 |
| Ranunculaceae | *Aconitum coreanum* (H. Lév.) Rapaics |  | N | 1 |
| Rutaceae | *Dictamnus dasycarpus* Turcz. | ○ |  | 3 |
| Fabaceae | *Vicia amurensis* Oett. | ○ |  | 4 |
| Aceraceae | *Acer triflorum* Kom. |  | N | 6 |
| Aceraceae | *Acer ukurunduense* Trautv. & C.A. Mey. |  | N | 3 |
| Brassicaceae | *Erysimum amurense* Kitag. | ○ |  | 1 |
| Salicaceae | *Salix rorida* Laksch. | ○ |  | 1 |
| Pinaceae | *Abies nephrolepis* (Trautv. ex Maxim.) Maxim. | ○ | N | 2 |
| Iridaceae | *Iris sanguinea* Donn ex Hornem. | ○ |  | 4 |
| Asteraceae | *Saussurea odontolepis* (Herder) Sch.Bip. ex Maxim. | ○ |  | 1 |
| Asteraceae | *Artemisia lancea* Vaniot | ○ |  | 2 |
| Betulaceae | *Betula ermanii* Cham. | ○ |  | 2 |
| Anacardiaceae | *Toxicodendron sylvestre* (Siebold & Zucc.) Kuntze |  | S | 2 |
| Aceraceae | *Acer tegmentosum* Maxim. | ○ | N | 1 |
| Cyperaceae | *Carex leiorhyncha* C.A. Mey. | ○ |  | 1 |
| Papaveraceae | *Corydalis speciosa* Maxim. | ○ |  | 15 |
| Asteraceae | *Dendranthema zawadskii* (Herbich) Tzvelev | ○ |  | 7 |
| Rosaceae | *Pyrus ussuriensis* Maxim. ex Rupr. | ○ |  | 4 |
| Rosaceae | *Rubus crataegifolius* Bunge | ○ |  | 17 |
| Rosaceae | *Prunus sargentii* Rehder | ○ |  | 7 |
| Liliaceae | *Allium thunbergii* G. Don | ○ |  | 6 |
| Cyperaceae | *Carex heterolepis* Bunge | ○ |  | 2 |
| Asteraceae | *Tephroseris flammea* (Turcz. ex DC.) Holub | ○ |  | 1 |
| Woodsiaceae | *Woodsia subcordata* Turcz. | ○ |  | 2 |
| Paeoniaceae | *Paeonia obovata* Maxim. | ○ |  | 1 |
| Rosaceae | *Agrimonia coreana* Nakai | ○ |  | 3 |
| Euphorbiaceae | *Mercurialis leiocarpa* Siebold & Zucc. |  | S | 1 |
| Liliaceae | *Paris verticillata* M. Bieb. | ○ |  | 6 |
| Crassulaceae | *Hylotelephium viviparum* (Maxim.) H.Ohba | ○ |  | 3 |
| Lauraceae | *Neolitsea aciculata* (Blume) Koidz. |  | S | 1 |
| Menispermaceae | *Menispermum dauricum* DC. | ○ |  | 9 |
| Rosaceae | *Rosa davurica* Pall. | ○ |  | 1 |
| Geraniaceae | *Geranium krameri* Franch. & Sav. | ○ |  | 1 |
| Lauraceae | *Machilus japonica* Siebold & Zucc |  | S | 1 |
| Pinaceae | *Pinus densiflora* Siebold & Zucc. | ○ |  | 15 |
| Asteraceae | *Tephroseris kirilowii* (Turcz. ex DC.) Holub | ○ |  | 1 |
| Rosaceae | *Potentilla discolor* Bunge | ○ |  | 1 |
| Scrophulariaceae | *Pedicularis resupinata* L. | ○ |  | 6 |
| Lamiaceae | *Leonurus macranthus* Maxim. | ○ |  | 3 |
| Asteraceae | *Scorzonera albicaulis* Bunge | ○ |  | 1 |
| Rosaceae | *Rubus corchorifolius* L.f. |  | S | 2 |
| Asteraceae | *Synurus deltoides* (Aiton) Nakai | ○ |  | 3 |
| Rosaceae | *Sorbaria sorbifolia* (L.) A. Braun var. *stellipila* Maxim. | ○ |  | 9 |
| Aceraceae | *Acer komarovii* Pojark. | ○ |  | 2 |
| Rosaceae | *Prunus sibirica* L. | ○ |  | 1 |
| Cyperaceae | *Carex ussuriensis* Kom. | ○ |  | 2 |
| Rosaceae | *Spiraea chartacea* Nakai | ○ |  | 5 |
| Violaceae | *Viola variegata* Fisch. ex Link | ○ |  | 9 |
| Cyperaceae | *Carex laevissima* Nakai | ○ |  | 1 |
| Saxifragaceae | *Chrysosplenium flagelliferum* F. Schmidt | ○ |  | 2 |
| Liliaceae | *Disporum smilacinum* A. Gray | ○ |  | 11 |
| Urticaceae | *Urtica laetevirens* Maxim. | ○ |  | 2 |
| Rosaceae | *Malus baccata* (L.) Borkh. | ○ | N | 8 |
| Hydrangeaceae | *Philadelphus tenuifolius* Rupr. & Maxim. | ○ |  | 20 |
| Rosaceae | *Potentilla fragarioides* L. var. *major* Maxim. | ○ |  | 11 |
| Liliaceae | *Erythronium japonicum* Decne. | ○ |  | 2 |
| Asteraceae | *Carpesium macrocephalum* Franch. & Sav. | ○ | N | 1 |
| Fabaceae | *Vicia venosa* (Link) Maxim. | ○ |  | 1 |
| Liliaceae | *Trillium camschatcense* Ker Gawl. | ○ | N | 1 |
| Campanulaceae | *Asyneuma japonicum* (Miq.) Briq. | ○ |  | 5 |
| Euphorbiaceae | *Mallotus japonicus* (L.f.) Müll.Arg. |  | S | 2 |
| Araliaceae | *Eleutherococcus sessiliflorus* (Rupr. & Maxim.) S.Y. Hu | ○ |  | 6 |
| Lamiaceae | *Isodon excisus* (Maxim.) Kudô | ○ |  | 4 |
| Schisandraceae | *Schisandra chinensis* (Turcz.) Baill. | ○ |  | 16 |
| Caprifoliaceae | *Lonicera praeflorens* Batalin | ○ |  | 16 |
| Ulmaceae | *Ulmus macrocarpa* Hance | ○ |  | 5 |
| Vitaceae | *Vitis amurensis* Rupr. | ○ |  | 13 |
| Poaceae | *Melica nutans* L. | ○ |  | 9 |
| Gentianaceae | *Gentiana scabra* Bunge | ○ |  | 2 |
| Liliaceae | *Polygonatum involucratum* (Franch. & Sav.) Maxim. | ○ |  | 6 |
| Lamiaceae | *Dracocephalum argunense* Fisch. ex Link | ○ |  | 1 |
| Ericaceae | *Vaccinium vitis-idaea* L. | ○ |  | 1 |
| Lauraceae | *Actinodaphne lancifolia* (Blume) Meisn. |  | S | 1 |
| Orchidaceae | *Cyrtosia septentrionalis* (Rchb.f.) Garay |  | S | 1 |
| Rosaceae | *Potentilla nivea* L. | ○ |  | 1 |
| Athyriaceae | *Cornopteris crenulato-serrulata* (Makino) Nakai | ○ |  | 1 |
| Rosaceae | *Rosa acicularis* Lindl. |  | N | 3 |
| Rosaceae | *Spiraea chamaedryfolia* L. | ○ |  | 7 |
| Betulaceae | *Betula pendula* Roth | ○ |  | 2 |
| Papaveraceae | *Corydalis incisa* (Thunb.) Pers. |  | S | 1 |
| Paeoniaceae | *Paeonia lactiflora* Pall. | ○ |  | 2 |
| Pinaceae | *Pinus koraiensis* Siebold & Zucc. | ○ |  | 9 |
| Pinaceae | *Abies holophylla* Maxim. | ○ | N | 2 |
| Ranunculaceae | *Ranunculus chinensis* Bunge | ○ |  | 1 |
| Violaceae | *Viola mandshurica* W. Becker | ○ |  | 5 |
| Papaveraceae | *Corydalis turtschaninovii* Besser |  | N | 1 |
| Violaceae | *Viola acuminata* Ledeb. | ○ |  | 19 |
| Asteraceae | *Aster maackii* Regel | ○ |  | 1 |
| Polypodiaceae | *Polypodium sibiricum* Sipliv. |  | N | 3 |
| Taxaceae | *Taxus cuspidata* Siebold & Zucc. | ○ |  | 2 |
| Dryopteridaceae | *Dryopteris fragrans* (L.) Schott | ○ | N | 9 |
| Boraginaceae | *Lithospermum erythrorhizon* Siebold & Zucc. | ○ |  | 2 |
| Ericaceae | *Rhododendron mucronulatum* Turcz. | ○ |  | 21 |
| Tiliaceae | *Tilia mandshurica* Rupr. & Maxim. | ○ |  | 6 |
| Rhamnaceae | *Rhamnus ussuriensis* J.J. Vassil. | ○ |  | 3 |
| Betulaceae | *Corylus sieboldiana* Blume | ○ |  | 9 |
| Lauraceae | *Neolitsea sericea* (Blume) Koidz. |  | S | 1 |
| Asteraceae | *Artemisia codonocephala* Diels |  | N | 3 |
| Rosaceae | *Spiraea fritschiana* C.K. Schneid. | ○ |  | 1 |
| Orchidaceae | *Gastrodia elata* Blume | ○ |  | 1 |
| Moraceae | *Ficus erecta* Thunb. |  | S | 1 |
| Ericaceae | *Rhododendron schlippenbachii* Maxim. | ○ |  | 11 |
| Chenopodiaceae | *Chenopodium bryoniifolium* Bunge | ○ |  | 1 |
| Juncaceae | *Juncus papillosus* Franch. & Sav. | ○ |  | 1 |
| Aceraceae | *Acer barbinerve* Maxim. | ○ |  | 2 |
| Campanulaceae | *Campanula punctata* Lam. | ○ |  | 5 |
| Asteraceae | *Rhynchospermum verticillatum* Reinw. |  | S | 1 |
| Oxalidaceae | *Oxalis obtriangulata* Maxim. | ○ |  | 1 |
| Gentianaceae | *Gentiana zollingeri* Fawc. | ○ |  | 4 |
| Poaceae | *Spodiopogon sibiricus* Trin. | ○ |  | 16 |
| Rubiaceae | *Rubia chinensis* Regel & Maack | ○ |  | 4 |
| Liliaceae | *Disporum viridescens* (Maxim.) Nakai | ○ |  | 2 |
| Primulaceae | *Primula jesoana* Miq. | ○ |  | 1 |
| Asteraceae | *Cirsium pendulum* Fisch. ex DC. | ○ | N | 1 |
| Rubiaceae | *Galium dahuricum* Turcz. ex Ledeb. | ○ |  | 4 |
| Araceae | *Arisaema ringens* (Thunb.) Schott |  | S | 1 |
| Rosaceae | *Filipendula glaberrima* Nakai | ○ |  | 1 |
| Caprifoliaceae | *Zabelia biflora* (Turcz.) Makino |  | N | 2 |
| Cystopteridaceae | *Gymnocarpium dryopteris* (L.) Newman | ○ |  | 2 |
| Liliaceae | *Polygonatum inflatum* Kom. | ○ |  | 4 |
| Dryopteridaceae | *Dryopteris expansa* (C. Presl) Fraser-Jenk. & Jermy | ○ |  | 5 |
| Tiliaceae | *Tilia amurensis* Rupr. | ○ |  | 14 |
| Papaveraceae | *Hylomecon vernalis* Maxim. |  | N | 1 |
| Chloranthaceae | *Chloranthus japonicus* Siebold | ○ |  | 4 |
| Campanulaceae | *Peracarpa carnosa* (Wall.) Hook.f. & Thomson |  | S | 1 |
| Lamiaceae | *Scutellaria baicalensis* Georgi | ○ |  | 1 |
| Rutaceae | *Phellodendron amurense* Rupr. | ○ |  | 5 |
| Celastraceae | *Euonymus sachalinensis* (F. Schmidt) Maxim. | ○ |  | 4 |
| Ranunculaceae | *Anemone reflexa* Steph. ex Willd. | ○ | N | 3 |
| Rosaceae | *Rosa koreana* Kom. | ○ | N | 3 |
| Violaceae | *Viola hirtipes* S. Moore | ○ |  | 3 |

※ NLP: Northern lineage plant. 300: 300 target plants adaptable to climate change in the Korean Peninsula (N: Northern Lineage plant, S: Southern Lineage plant), Fre.: Frequency
