# Supplementary material for: Floristic inventory and distribution characteristics of algific talus slopes in a specific area of forest biodiversity in South Korea
Source: Biodivers Data J. 2023 Dec 18;11:e113952. doi: 10.3897/BDJ.11.e113952 (PMC10838045; doi:10.3897/BDJ.11.e113952)
Supplement: Supplementary material 8 — List of calciphilous plants in the algific talus slopes of South Korea [file bdj-11-e113952-s008.docx]

9. List of alien plants in the algific talus slopes of South Korea.

| **Family name** | **Scientific name / Korean name** | **L-f** | **Orig.** | **Type** | **Degree** | **IDP** | **Fre.** |
| --- | --- | --- | --- | --- | --- | --- | --- |
|  |  |  |  |  |  |  |  |
| Ginkgoaceae | *Ginkgo biloba* L. | Tr. | As | Arc. | - |  | 2 |
| Pinaceae | *Larix kaempferi* (Lamb.) Carrière | Tr. | As | PIP (CAP) | - |  | 6 |
| Pinaceae | *Pinus rigida* Mill. | Tr. | nA | PIP (CAP) | - |  | 2 |
| Moraceae | *Morus alba* L. | Tr. | As | Arc. | - |  | 6 |
| Cannabaceae | *Humulus scandens* (Lour.) Merr. | 1 | · | · | - | ○ | 9 |
| Polygonaceae | *Fagopyrum esculentum* Moench | 1 | As | Arc. | - |  | 1 |
| Polygonaceae | *Rumex acetosella* L. | Pe. | As, Eu | IAP (NP) | WS | ○ | 2 |
| Polygonaceae | *Rumex crispus* L. | Pe. | Af, As, Eu | IAP (NP) | WS |  | 4 |
| Polygonaceae | *Rumex obtusifolius* L. | Pe. | Af, As, Eu | IAP (NP) | MS |  | 1 |
| Phytolaccaceae | *Phytolacca americana* L. | Pe. | nA, sA | IAP (NP) | WS |  | 2 |
| Caryophyllaceae | *Cerastium glomeratum* Thuill. | 2 | Af, Eu | IAP (NP) | MS |  | 2 |
| Caryophyllaceae | *Stellaria media* (L.) Vill. | 1 | Af, As, Eu | IAP (NP) | WS |  | 2 |
| Chenopodiaceae | *Chenopodium album* L. | 1 | As, Eu | IAP (NP) | CS |  | 2 |
| Chenopodiaceae | *Chenopodium ficifolium* Sm. | 1 | As, Eu | IAP (NP) | WS |  | 4 |
| Amaranthaceae | *Amaranthus blitum* L. subsp. *oleraceus* (L.) Costea | 1 | Eu | Arc. | - |  | 1 |
| Amaranthaceae | *Amaranthus patulus* Bertol. | 1 | nA, sA | IAP (NP) | CS |  | 2 |
| Amaranthaceae | *Amaranthus retroflexus* L. | 1 | nA | PIP (UN) | - |  | 2 |
| Brassicaceae | *Barbarea vulgaris* W.T.Aiton | Pe. | As, Eu | IAP (NP) | MS |  | 5 |
| Brassicaceae | *Lepidium apetalum* Willd. | 2 | As, Eu | PIP (UN) | - |  | 2 |
| Brassicaceae | *Thlaspi arvense* L. | 2 | As, Eu | Arc. | - |  | 1 |
| Rosaceae | *Prunus persica* (L.) Batsch | Tr. | As | Arc. | - |  | 3 |
| Rosaceae | *Prunus tomentosa* Thunb. | Tr. | As | PIP (CAP) | - |  | 1 |
| Fabaceae | *Amorpha fruticosa* L. | Tr. | nA | IAP (NP) | WS |  | 2 |
| Fabaceae | *Medicago lupulina* L. | 2 | Af, As, Eu | IAP (NP) | MS |  | 1 |
| Fabaceae | *Medicago sativa* L. | Pe. | Eu | IAP (NP) | CS |  | 1 |
| Fabaceae | *Melilotus albus* Medik. | 2 | Af, As, Eu | IAP (NP) | MS |  | 1 |
| Fabaceae | *Robinia pseudoacacia* L. | Tr. | nA | IAP (NP) | WS |  | 6 |
| Fabaceae | *Styphnolobium japonicum* (L.) Schott | Tr. | As | PIP (CAP) | - |  | 1 |
| Fabaceae | *Trifolium dubium* Sibth. | Pe. | Eu | IAP (NP) | PS |  | 1 |
| Fabaceae | *Trifolium pratense* L. | Pe. | Af, As, Eu | IAP (NP) | SS |  | 4 |
| Fabaceae | *Trifolium repens* L. | Pe. | Af | IAP (NP) | WS |  | 5 |
| Fabaceae | *Wisteria floribunda* (Willd.) DC. | Pe. | As | Arc. | - |  | 1 |
| Oxalidaceae | *Oxalis corniculata* L. | Pe. | nA | Arc. | - |  | 7 |
| Euphorbiaceae | *Euphorbia hypericifolia* L. | 1 | nA, sA | IAP (NP) | CS |  | 3 |
| Euphorbiaceae | *Euphorbia maculata* L. | 1 | nA, sA | IAP (NP) | SS |  | 3 |
| Simaroubaceae | *Ailanthus altissima* (Mill.) Swingle | Tr. | As | Arc. | - |  | 3 |
| Violaceae | *Viola sororia* Willd. | Pe. | nA | IAP (NP) | PS |  | 1 |
| Cucurbitaceae | *Sicyos angulatus* L. | 1 | nA, Oc | IAP (NP) | MS | ○ | 1 |
| Onagraceae | *Oenothera biennis* L. | 2 | nA | IAP (NP) | WS |  | 11 |
| Apiaceae | *Anthriscus caucalis* M. Bieb. | 1 | Af, Eu | IAP (NP) | PS |  | 2 |
| Rubiaceae | *Galium tricornutum* Dandy | 1 | As, Eu | PIP (UN) | - |  | 3 |
| Convolvulaceae | *Ipomoea nil* (L.) Roth | 1 | sA | IAP (NP) | MS |  | 1 |
| Convolvulaceae | *Quamoclit coccinea* (L.) Moench | 1 | nA, sA | IAP (NP) | CS |  | 1 |
| Lamiaceae | *Lamium purpureum* L. | 2 | As, Eu | IAP (NP) | PS |  | 1 |
| Lamiaceae | *Scutellaria baicalensis* Georgi | Pe. | As | PIP (UN) | - |  | 1 |
| Solanaceae | *Physalis alkekengi* L. | Pe. | As, Eu | PIP (CAP) | - |  | 1 |
| Solanaceae | *Solanum nigrum* L. | Pe. | Af, Eu | Arc. | - |  | 5 |
| Scrophulariaceae | *Veronica anagallis-aquatica* L. | Pe. | As, Eu | IAP (CAP) | CS |  | 1 |
| Scrophulariaceae | *Veronica arvensis* L. | 1 | Af, As, Eu | IAP (NP) | WS |  | 1 |
| Scrophulariaceae | *Veronica persica* Poir. | 2 | As | IAP (NP) | WS |  | 2 |
| Asteraceae | *Ageratina altissima* (L.) R.M. King & H.Rob. | Pe. | nA | IAP (NP) | PS | ○ | 1 |
| Asteraceae | *Ambrosia artemisiifolia* L. | 1 | nA | IAP (NP) | WS | ○ | 6 |
| Asteraceae | *Ambrosia trifida* L. | 1 | nA | IAP (NP) | MS | ○ | 3 |
| Asteraceae | *Bidens frondosa* L. | 1 | nA | IAP (NP) | WS |  | 7 |
| Asteraceae | *Carduus crispus* L. | 2 | As, Eu | IAP (NP) | SS |  | 7 |
| Asteraceae | *Conyza canadensis* (L.) Cronquist | 2 | nA, sA | IAP (NP) | WS |  | 5 |
| Asteraceae | *Eclipta thermalis* Bunge | 1 | nA, sA | Arc. | - |  | 3 |
| Asteraceae | *Erechtites hieraciifolius* (L.) Raf. ex DC. | 1 | nA, sA | IAP (NP) | WS |  | 4 |
| Asteraceae | *Erigeron annuus* (L.) Pers. | 2 | nA | IAP (NP) | WS |  | 13 |
| Asteraceae | *Erigeron philadelphicus* L. | Pe. | nA | IAP (NP) | PS |  | 3 |
| Asteraceae | *Erigeron strigosus* Muhl. ex Willd. | 2 | nA | IAP (NP) | MS |  | 1 |
| Asteraceae | *Galinsoga quadriradiata* Ruiz & Pav. | 1 | nA, sA | IAP (NP) | WS |  | 7 |
| Asteraceae | *Senecio vulgaris* L. | 1 | As, Eu | IAP (NP) | SS |  | 3 |
| Asteraceae | *Solidago altissima* L. | Pe. | nA | IAP (NP) | PS | ○ | 1 |
| Asteraceae | *Solidago gigantea* Aiton | Pe. | nA | IAP (NP) | CS |  | 1 |
| Asteraceae | *Sonchus asper* (L.) Hill | 1 | Af, As, Eu | IAP (NP) | SS |  | 1 |
| Asteraceae | *Sonchus oleraceus* L. | 1 | As, Eu | IAP (NP) | SS |  | 1 |
| Asteraceae | *Symphyotrichum pilosum* (Willd.) G.L. Nesom | Pe. | nA | IAP (NP) | SS | ○ | 4 |
| Asteraceae | *Tagetes minuta* L. | 1 | sA | IAP (NP) | MS |  | 3 |
| Asteraceae | *Taraxacum officinale* F.H. Wigg. | Pe. | Eu | IAP (NP) | WS |  | 9 |
| Liliaceae | *Allium tuberosum* Rottler ex Spreng. | Pe. | As | PIP (CAP) | - |  | 1 |
| Poaceae | *Dactylis glomerata* L. | Pe. | As, Eu | IAP (NP) | WS |  | 2 |
| Poaceae | *Festuca arundinacea* Schreb. | Pe. | Af, As, Eu | IAP (NP) | SS |  | 2 |
| Poaceae | *Poa pratensis* L. | Pe. | nA, As, Eu | IAP (NP) | SS |  | 2 |
| Poaceae | *Vulpia myuros* (L.) C.C. Gmel. | 1 | Af, As, Eu | IAP (NP) | MS |  | 1 |
| Zingiberaceae | *Zingiber mioga* (Thunb.) Roscoe | Pe. | As | IAP (NP) | PS |  | 1 |

* L-f: Life form (Tr.: Tree, 1: Annual, 2: Biennial, Pre.: Perennial), Orig.: Origins (Af: Africa, As: Asia, Eu: Europe, nA: North America), Type [Arc.: Archaeophyte, PIP: Potentially invasive plant (CAP: Concerned alien plant, UN: Uncertain plant), IAP: Invasive alien plant (CAP: Casual alien plant, NP: Naturalized plant)], Degree (WS: Widespread, SS: Serious spread, CS: Concerned spread, MS: Minor spread, PS: Potential spread), IDP: Introduced disturbing plant, Fre.: Frequency
