# Supplementary material for: Floristic inventory and distribution characteristics of algific talus slopes in a specific area of forest biodiversity in South Korea
Source: Biodivers Data J. 2023 Dec 18;11:e113952. doi: 10.3897/BDJ.11.e113952 (PMC10838045; doi:10.3897/BDJ.11.e113952)
Supplement: Supplementary material 10 — Flora of algific talus slopes by type in South Korea [file bdj-11-e113952-s010.docx]

10. Flora of algific talus slopes by type in South Korea

| **Type** | **Families** | **Genera** | **Species** | **subbsp.** | **var.** | **f.** | **Total** | **Ratio (%)** |
| --- | --- | --- | --- | --- | --- | --- | --- | --- |
| Talus | 114 | 394 | 722 | 18 | 59 | 5 | 804 | 76.43 |
| Cave | 107 | 361 | 623 | 18 | 53 | 7 | 701 | 66.63 |
| Dent | 82 | 173 | 274 | 5 | 25 | 2 | 306 | 29.09 |
| Vertical cave | 44 | 68 | 77 | 1 | 2 | 0 | 80 | 7.60 |
| Others | 72 | 129 | 152 | 5 | 14 | 2 | 173 | 16.44 |
| **Total** | **125** | **486** | **947** | **23** | **75** | **7** | **1,052** | **100** |
