# Supplementary material for: Floristic inventory and distribution characteristics of algific talus slopes in a specific area of forest biodiversity in South Korea
Source: Biodivers Data J. 2023 Dec 18;11:e113952. doi: 10.3897/BDJ.11.e113952 (PMC10838045; doi:10.3897/BDJ.11.e113952)
Supplement: Supplementary material 11 — Flora of algific talus slopes by region in South Korea [file bdj-11-e113952-s011.docx]

11. Flora of algific talus slopes by region in South Korea

| **ID/Study site** | **Families** | **Genera** | **Species.** | **subsp.** | **var.** | **f.** | **Total** |
| --- | --- | --- | --- | --- | --- | --- | --- |
| A-T-1/Pocheon | 59 | 107 | 117 | 2 | 14 | 0 | 133 |
| A-T-2/Hongcheon | 76 | 176 | 267 | 5 | 23 | 3 | 298 |
| A-T-3/Pyeongchang | 71 | 162 | 220 | 8 | 20 | 2 | 250 |
| A-T-4/Jeongseon | 84 | 203 | 295 | 6 | 28 | 2 | 331 |
| A-T-5/Jeongseon | 65 | 122 | 125 | 8 | 11 | 1 | 145 |
| A-T-6/Hwacheon | 49 | 71 | 72 | 0 | 8 | 0 | 80 |
| A-T-7/Inje | 86 | 204 | 241 | 11 | 26 | 3 | 281 |
| A-T-8/Boeun | 52 | 84 | 91 | 1 | 11 | 0 | 103 |
| A-T-9/Danyang | 52 | 88 | 98 | 0 | 6 | 1 | 105 |
| A-T-10/Jeongeup | 38 | 52 | 55 | 1 | 6 | 0 | 62 |
| A-T-11/Yeongcheon | 37 | 54 | 61 | 2 | 5 | 0 | 68 |
| A-T-12/Gunwi | 48 | 84 | 83 | 4 | 11 | 1 | 99 |
| A-T-13/Cheongsong | 80 | 207 | 260 | 12 | 24 | 2 | 298 |
| A-T-14/Miryang | 72 | 150 | 189 | 7 | 14 | 3 | 213 |
| A-C-1/Yeoncheon | 77 | 173 | 231 | 5 | 24 | 5 | 265 |
| A-C-2/Jeongseon | 87 | 256 | 377 | 13 | 36 | 5 | 431 |
| A-C-3/Jinan | 63 | 139 | 167 | 5 | 12 | 2 | 186 |
| A-C-4/Uiseong | 81 | 232 | 326 | 9 | 24 | 2 | 361 |
| A-D-1/Hongcheon | 31 | 46 | 53 | 0 | 0 | 0 | 53 |
| A-D-2/Jecheon | 65 | 122 | 168 | 3 | 19 | 2 | 192 |
| A-D-3/Boeun | 58 | 106 | 154 | 3 | 15 | 2 | 174 |
| A-D-4/Miryang | 25 | 30 | 31 | 1 | 3 | 0 | 35 |
| A-V-1/Jeju | 44 | 68 | 77 | 1 | 2 | 0 | 80 |
| A-O-1/Haenam | 45 | 78 | 78 | 2 | 11 | 2 | 93 |
| A-O-2/Hamyang | 54 | 79 | 88 | 3 | 6 | 0 | 97 |
